# Comprehensive Modular Synthesis of Ganglioside Glycans and Evaluation of their Binding Affinities to Siglec‐7 and Siglec‐9

**DOI:** 10.1002/advs.202412815

**Published:** 2024-11-18

**Authors:** Avijit K. Adak, Hsin‐Kai Tseng, Shu‐Yen Chang, Yu‐Ching Chiang, Ke‐Hong Lyu, Yun‐Sheng Lee, Wen Lu, Wen‐Hua Kuo, Takashi Angata, Chun‐Cheng Lin

**Affiliations:** ^1^ Department of Chemistry National Tsing Hua University 101 Section 2, Kuang Fu Road Hsinchu 30013 Taiwan; ^2^ Institute of Biological Chemistry Academia Sinica Taipei 11529 Taiwan; ^3^ Department of Medicinal and Applied Chemistry Kaohsiung Medical University Kaohsiung 80708 Taiwan

**Keywords:** carbohydrates, chemoenzymatic synthesis, gangliosides, glycan microarray, Siglec

## Abstract

In the present work, bacterial glycosyltransferases are utilized to construct ganglioside glycans in a convergent approach via a sugar‒nucleotide regeneration system and one‐pot multienzyme reactions. Starting from β‐lactoside enables the diversification of both the glycan moieties and the linkages in the lower α‐arm and upper β‐arm. Overall, a comprehensive panel of 24 natural a‐series (GM3, GM2, GM1a, GD1a, GT1a, and fucosyl‐GM1), b‐series (GD3, GD2, GD1b, GT1b, and GQ1b), c‐series (GT3, GT2, GT1c, GQ1c, and GP1c), α‐series (GM1α, GD1aα, and GT1aα), and o‐series (GA2, GA1, GM1b, GalNAc‐GM1b, and GD1c) ganglioside glycans are prepared, which are suitable for biological studies and further applications. Moreover, a microarray is constructed with these synthesized ganglioside glycans to investigate their binding specificity with recombinant Fc‐fused Siglec‐7 and Siglec‐9, which are immune checkpoint‐like glycan recognition proteins on natural killer cells. The microarray binding results reveal that GD3 and GT1aα are specific ligands for Siglec‐7 and Siglec‐9, respectively, and this discovery can lead to the identification of appropriate ligands for investigating the roles of these Siglecs in immunomodulation.

## Graphical abstract

The modular enzymatic synthesis of a library of 24 ganglioside glycans is successfully achieved by using bacterial glycosyltransferases in a reprogrammed synthetic route. Glycan microarray analysis reveals that these synthetic ganglioside glycans, featuring various spatial arrangements of sialic acids, are specifically bound to sialic acid‐binding immunoglobulin‐type lectins (Siglec‐7 and Siglec‐9).

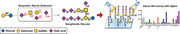

## 1 Introduction

Glycosphingolipids (GSLs) are abundant in lipid rafts, which are specialized microdomains in the cell membrane crucial for signal transduction.^[^ ^1^ ^]^ Gangliosides are a subclass of GSLs that contain sialic acid (Sia) and consist of an extracellular acidic sialylated glycan headgroup attached to a double‐tailed hydrophobic ceramide (Cer) moiety.^[^ ^2^ ^]^ Particularly rich in the mammalian brain, gangliosides such as GM1a, GD1a, GD1b, and GT1b play key roles in neural functions.^[^ ^2^, ^3^ ^]^ These gangliosides feature specific structures, such as a GM1a core with an additional α(2,3)‐Sia attached to the upper β‐arm, α(2,8)‐Sia positioned adjacent to the Sia on the lower α‐arm, or an internal α(2,6)‐Sia linked to the GalNAc residue. They also include more complex variants, such as the a‐, b‐, c‐, and α‐series gangliosides like GT1a, GT1aα, GQ1b, and GQ1c. Certain gangliosides, such as GM3, GM2, GD2, and GD3, although typically expressed at low levels in normal tissues, are overexpressed in tumors,^[^ ^4^ ^]^ with the prominent tumor‐associated gangliosides GD2 and GD3 being promising targets for cancer immunotherapy.^[^ ^5^, ^6^ ^]^ Gangliosides have diverse functions, including promoting signal transduction, interacting with adhesion proteins, and the development of the brain, with implications in neurodegenerative diseases, neuroplasticity, and storage disorders.^[^ ^7^ ^,^ ^8^ ^]^

Sialic acid‐binding immunoglobulin‐like lectins (Siglecs) are transmembrane receptors that modulate immunity by binding Neu5Ac‐bearing glycans.^[^ ^9^ ^]^ Each Siglec has distinct ligand binding preferences with endogenous sialylated glycans and a unique pattern of expression.^[^ ^10^ ^]^ Siglec‐7, a potential cancer immunotherapy target expressed strongly on most natural killer (NK) cells,^[^ ^11^ ^]^ prefers the Neu5Acα(2,8)Neu5Ac‐disialyl structures found on b‐series gangliosides.^[^ ^12^, ^13^ ^]^ Siglec‐9, which is closely related to Siglec‐7, is expressed on early NK cells, suggesting that it is a potential target for antitumor immunotherapy.^[^ ^14^, ^15^ ^]^ To elucidate the biological interactions at the molecular level, the ligand preferences of these Siglecs were consequently studied.^[^ ^16^ ^]^ However, their detailed glycan recognition specificities, especially those concerning Neu5Ac linkages and Neu5Acα(2,3)Galβ1,3 extension of the GalNAcβ1,4 branch in the context of natural ganglioside glycans, have not been fully established, partly owing to limitations in accessing these complex glycans.^[^ ^17^ ^]^ To address this issue, it has been suggested that a systematic chemoenzymatic strategy to produce well‐defined ganglioside glycans be designed. Total synthesis is often favored for generating gangliosides due to complexities and heterogeneities in both the glycan and Cer portions.^[^ ^18^ ^]^ Unlike chemical glycosylation reactions, enzymatic glycosylation reactions offer precise regio‐ and stereoselectivity, improving synthetic efficiency.^[^ ^19^ ^]^ For example, Blixt et al. synthesized various ganglioside glycans (GD3, GT3, GM2, GD2, GT2, GM1, and GD1a) with a 2‐azidoethyl aglycone via specific bacterial glycosyltransferases (GTs).^[^ ^20^ ^]^ We^[^ ^21^, ^22^ ^]^ and Chen et al.^[^ ^23^ ^]^ developed a sequential one‐pot reaction (SOPME) or one‐pot multienzyme (OPME) system to simplify the synthetic process without the need to purify sugar nucleotide donors. While earlier efforts focused on the synthesis of the glycan moieties of a‐ and b‐series gangliosides, recent work has involved incorporating the Cer moiety.^[^ ^24^ ^]^ The human ST, ST3GAL II, efficiently installs the upper arm α(2,3)Neu5Ac‐linkage after expression in *Escherichia coli*.^[^ ^25^ ^]^ Moreover, Boons^[^ ^26^ ^]^ applied α2,3‐ST, PmST1, from *Pasteurella multocida* ^[^ ^27^ ^]^ for α(2,3) sialylation of ganglioside derivatives.

Recently, we utilized GTs, especially STs, for the chemoenzymatic synthesis of the DSGb5 glycan moiety via the SOPME strategy. In SOPME, the required enzymes and materials are sequentially added to the same reaction flask after the previous enzymatic reaction has been completed.^[^ ^28^ ^]^ Additionally, a sugar nucleotide regeneration system (SNRS), which regenerates sugar‒nucleotide donors after glycosidic bond formation,^[^ ^29^, ^30^, ^31^, ^32^ ^]^ was implemented to construct sialylated or fucosylated oligo‐LacNAc (Galβ(1,4)GlcNAc) and several members of the globo series of glycolipids were synthesized in high yields via these strategies.^[^ ^33^, ^34^ ^]^

**Figure 1:**
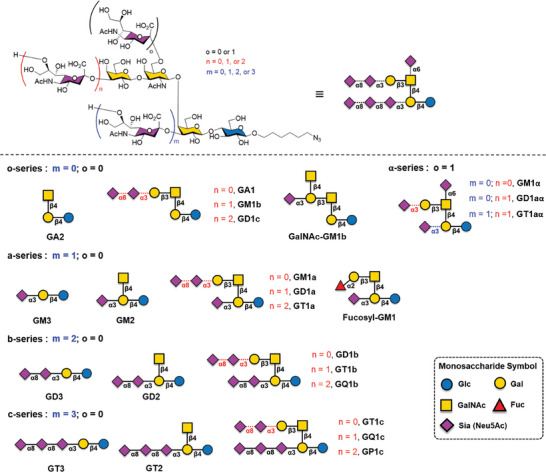
The general chemical structure of a ganglioside glycan (top panel) and structures of major o‐, a‐, b‐, c‐, and α‐series ganglioside glycans (bottom panel) synthesized and evaluated for their binding specificity with Siglec‐7‐Fc and Siglec‐9‐Fc in this study.

Herein, we present the successful chemoenzymatic synthesis of diverse ganglioside glycans by extending spacer‐modified β‐Lac via the SNRS and SOPME methods. Initially, we synthesized the core glycans GM3, GD3, and GT3 and subsequently elongated the cores using robust GTs, resulting in the synthesis of 24 natural ganglioside glycans categorized into five distinct series: a, b, c, o, and α (**Figure** **1**). This strategic approach enables the controlled diversification of glycan moieties and linkages in both the upper β‐arm and lower α‐arm of the ganglioside glycans, covering nearly all the enzymatically accessible α2,3‐, α2,6‐, and α2,8‐sialyl linkages. Additionally, upon sialidase treatment and GT extension, we successfully transformed GM2 and GM1a into GA2 and GA1, respectively, and further extension to GM1α and GD1aα in the α‐series of gangliosides, and GalNAc‐GM1b within the o‐series. We evaluated the binding properties of these ganglioside glycans by constructing a glycan microarray using the synthesized glycans and probed it with recombinant human Siglec‐7‐Fc and Siglec‐9‐Fc. Our results demonstrated the preferential binding of Siglec‐7‐Fc and Siglec‐9‐Fc to certain ganglioside glycans, highlighting their specificity for distinct sialic acid linkages and glycan structures within the ganglioside molecules.

## 2 Results and Discussion

**Scheme 1:**
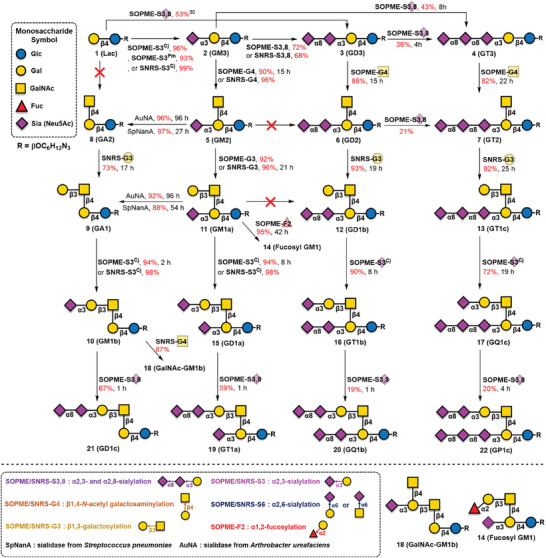
Synthesis of ganglioside glycans by the SNRS or SOPME systems coupled with glycosyltransferases. See supporting information for reaction system abbreviation and details conditions.

To facilitate the production of natural ganglioside glycans, we initiated the chemoenzymatic synthesis of **2** (**GM3**, **Scheme** **1**) via a well‐established process.^[^ ^20^, ^30^, ^35^ ^]^ As shown in Scheme 1, the process first employed NmCSS, a CMP‐sialic acid synthetase from *Neisseria meningitidis*,^[^ ^36^ ^]^ to fully generate CMP‐Sia in the presence of cytidine 5′‐triphosphate (CTP) and Neu5Ac. This was followed by the addition of lactoside **1** (Galβ(1,4)Glc‐βOR, R = (CH_2_)_6_N_3_, **Lac**)^[^ ^37^ ^]^ and the α(2,3)ST, either PmST1 or CjCst‐I from *C. jejuni* ^[^ ^38^ ^]^ (a typical SOPME protocol). Both enzymes, PmST1 and CjCst‐I (the reaction conditions are denoted as SOPME‐S3^Pm^ and SOPME‐S3^Cj^, respectively), efficiently produced GM3 trisaccharide in 93% and 96% yield, respectively (Scheme 1). In a parallel approach, α(2,3)‐sialylation of **1** in the SNRS with CjCst‐I (the reaction condition was denoted as SNRS‐S3^Cj^) led to the formation of **2** in nearly quantitative yield (99%) on a 444 mg scale.

For the synthesis of glycans **3** (**GD3**) and **4** (**GT3**), **1** and **2** were used as acceptors and were sialylated by the α2,3/8‐ST CjCst‐II.^[^ ^39^ ^]^ In a SOPME with CjCst‐II (denoted as SOPME‐S3,8), **1** was directly converted to **3** (**GD3**) in 53% yield by using 10 equiv. of Neu5Ac. To improve the yield, **2** was used as an acceptor along with 0.8 equiv. of Neu5Ac in SOPME‐S3,8 in 72% yield of **3** and 6% yield of **4** (**GT3**). However, altering the molar ratio of Neu5Ac to 1 equiv. led to decreased production of **3** (66%). Alternatively, a comparable yield (68%) was achieved when α(2,8)‐sialylation was carried out in the SNRS with CjCst‐II (denoted as SNRS‐S3,8). Despite the successful preparation of **3**, the enzymatic synthesis of **4** using CjCst‐II proved to be more challenging due to its promiscuous activity toward the sialosides.

To investigate the optimal conditions for the synthesis of **4** (**GT3**), **2** (**GM3**) was used as the acceptor under SOPME‐S3,8 with different reaction conditions (Neu5Ac 1.0, 1.5, 1.8, 2.0, 2.3, and 2.5 equiv.; reaction times of 1.5, 3, 5, 7.5, and 26 h). The highest yield of **4** (**GT3**) (43%) was obtained after 8 h with 2.5 equiv. of Neu5Ac, along with the formation of **3** (**GD3**) (39%) and a trace amount of oligo‐sialylated compound **GQ3** (6%). The remaining acceptor **2** (10%) in the reaction mixture indicated that, at higher molar ratios of Neu5Ac, **3** could compete with **2** to serve as an acceptor.^[^ ^20^ ^]^ Therefore, SOPME‐S3,8 was applied to **3** to yield **4** with 1 equiv. of Neu5Ac for 4 h of reaction. However, under these conditions, the yield of **4** decreased slightly to 36%, accompanied by the hydrolysis product **2** (10%), and **GQ3** (5%) and the recovery of **3** (49%). These results revealed the superior acceptor properties of **2** over those of **1** and **3** for CjCst‐II.

Once **2**, **3**, and **4** were obtained, attempts were made to perform β(1,4)‐GalNAcylation via the SOPME with CjCgtA, a β(1,4)‐*N*‐acetylgalactosaminyltransferase from *C. jejuni OH4384* ^[^ ^40^ ^]^ (denoted SOPME‐G4, Scheme 1). In the SOPME‐G4 process, BlNahK (an *N*‐acetylhexosamine‐1‐kinase from *Bifidobacterium longum*)^[^ ^41^ ^]^ and AGX1 (a UDP‐GalNAc pyrophosphorylase from *Homo sapiens*)^[^ ^42^ ^]^ were used to generate the corresponding GalNAc‐1‐phosphate and UDP‐GalNAc, respectively, while CjCgtA was used to construct β(1,4) glycosidic bonds. The results confirmed the successful installation of the GalNAc moiety to yield **5** (**GM2**) (90%), **6** (**GD2**) (88%), and **7** (**GT2**) (82%). Notably, the replacement of AGX1 with GlmU, an *N*‐acetylglucosamine 1‐phosphate uridyltransferase from *E. coli*,^[^ ^43^ ^]^ resulted in a slower reaction rate. Compound **5** was also obtained by the SNRS with CjCgtA (denoted as SNRS‐G4) in 96% yield. Notably, both **GM3** and **GD3** generally showed quantitative conversion within 15 h by thin‐layer chromatography (TLC), whereas **GT3** required a higher molar ratio of GalNAc (2 equiv.) and a relatively longer time (22 h) for full conversion. In addition, we found that compound **5** (**GM2**) could not serve as an acceptor for CjCst‐II to produce **6** (**GD2**), whereas **6** (**GD2**) could be further α(2,8)‐sialylated by CjCst‐II to form **7** (**GT2**) in low yield (21%). This suggests that the presence of the adjacent β(1,4)GalNAc‐linked motif in **5** might impart steric hindrance, blocking the α(2,8)‐sialylation activity of CjCst‐II.

The synthesis of **8** (**GA2**), **9** (**GA1**), and **10** (**GM1b**) faced challenges because CjCgtA requires the presence of α(2,3)Neu5Ac at the terminal Gal residue to assemble a β(1,4)GalNAc.^[^ ^44^ ^]^ Such specificity prevents the extension of compound **1** using this enzyme. To overcome this obstacle, a strategy involving removing Neu5Ac from **5** (**GM2**) via a sialidase was applied. The neuraminidase (NA) from *Arthrobacter ureafaciens* (AuNA) or the sialidase from *Streptococcus pneumoniae* (SpNanA)^[^ ^45^ ^]^ could be used to successfully remove the Neu5Ac residue from **5**, leading to the formation of **8** in 96% (AuNA for 96 h) and 97% (SpNanA for 27 h) yields. Importantly, the Neu5Ac component of **5** could also be removed under a formic acid solution (1 m) at 80 °C for 2 h, producing **8** in 49% yield. Having **8** in hand allowed further elaboration with GTs to produce **9** and **10** (see below). Compound **5** was transformed into **11** (**GM1a**) by forming a β(1,3)‐Gal glycosidic bond in 92% yield via incubation with 1.05 equiv. of Gal in a SOPME containing CjCgtB, a β(1,3)‐galactosyltransferase from *C. jejuni*,^[^ ^46^ ^]^ UDP‐sugar pyrophosphorylase (AtUSP),^[^ ^47^ ^]^ and *Meiothermus taiwanensis* galactokinase (MtGalK),^[^ ^48^ ^]^ (denoted as SOPME‐G3). Notably, CjCgtB can recognize both Gal and GalNAc as the acceptor, with a preference for that latter.^[^ ^46^ ^]^ Thus, controlling the amount of UDP‐Gal was critically important for suppressing di‐galactosylation during CjCgtB‐catalyzed glycosylation. Notably, LgtD from *Haemophilus influenzae* strain Rd,^[^ ^49^ ^]^ which has β(1,3)‐galactosyltransferase activity, could not recognize **5** (**GM2**) as the acceptor. To simplify the synthetic protocol, an effective SNRS with CjCgtB (denoted as SNRS‐G3) was then performed on **5** (**GM2**), **6** (**GD2**), and **7** (**GT2**) to give **11** (**GM1a**), **12** (**GD1b**), and **13** (**GT1c**), respectively, in excellent yields of 96%, 93%, and 92%, respectively. However, the catalytic activity of CjCgtB was found to be affected by Neu5Ac; as the amount of Neu5Ac on the molecule increases, the reaction rate decreases, and 19–25 h is usually required for completion of the reaction. We noted that **8** (**GA2**) was a good acceptor for CjCgtB in the SNRS protocol, but **9** (**GA1**) was provided in relatively lower yield (73%) due to the loss of product during purification. However, **9** (**GA1**) could also be obtained from **11** (**GM1a**) by desialylation via sialidases. The exposure of **11** to AuNA for 96 h gave **9** in 92% yield, whereas shorter reaction times (54 h) were required when using SpNanA with a yield of 88%. Acidolysis of **11** using formic acid solution led to the formation of **9** in 63% yield. The results of enzymatic hydrolysis revealed that the activity of SpNanA decreased as the number of sugars in the upper β‐arm increased, possibly because of steric hindrance. Moreover, **11** could easily be transformed into **14** (**fucosyl‐GM1**) in 95% yield by the SOPME system via FutC (an α(1,2)‐fucosyltransferase from *Helicobacter pylori*),^[^ ^50^ ^]^ and BfFKP (a bifunctional enzyme from *Bacteroides fragilis* with fucokinase/GDP‐fucose pyrophosphorylase activities),^[^ ^51^ ^]^ (denoted as SOPME‐F2).

To efficiently install α(2,3)‐Neu5Ac at the upper arm, three different α(2,3)STs, PmST1, CjCst‐I, and NgST,^[^ ^52^ ^]^ were investigated for their ability to assemble Neu5Ac on the nonreducing end of Gal in **11** (**GM1a**) (the upper β‐arm). Synthesis was performed via the SOPME‐S3 protocol (but using different α(2,3)STs) with 5 equiv. of Neu5Ac for a duration of 6–47 h. Overall, **15** (**GD1a**) was produced in various yields, depending on the ST used. CjCst‐I yielded **15** in 80% yield after 6 h, whereas PmST1 produced only 13% yield of the desired product in 47 h. In contrast, no product was detected after 19 h of reaction with NgST. Notably, extending the CjCst‐I‐catalyzed sialylation reaction time to 8 h significantly increased the yield to 94%. Similar reaction conditions were then applied for the synthesis of **10** (**GM1b**), **16** (**GT1b**), and **17** (**GQ1c**) from **9** (**GA1**), **12** (**GD1b**), and **13** (**GT1c**), respectively. CjCst‐I showed high efficiency with **9** (**GA1**) as the acceptor (by SOPME‐S3^Cj^), resulting in 94% yield of **10** (**GM1b**) after 2 h of incubation.

Similarly, **16** (**GT1b**) was obtained in 90% yield after an 8‐h reaction, whereas **17** (**GQ1c**) was obtained in 72% yield after a 19‐h reaction. Overall, the sialylation efficiency was influenced by the amount of Neu5Ac present on the acceptor, with lower Neu5Ac content resulting in the shorter completion time. Additionally, we found that the syntheses of **10** (**GM1b**) and **15** (**GD1a**) using SNRS‐S3^Cj^ were highly efficient, achieving excellent yields from **9** (**GA1**) (98%) and **11** (**GM1a**) (98%), respectively. A surprising finding was the ability of **10** (**GM1b**) to serve as an acceptor for GalNAcylation by CjCgtA via SNRS‐G4 resulting in 87% yield of **18** (**GalNAc‐GM1b**), which was isolated from patients with Guillain–Barre syndrome.^[^ ^53^ ^]^ Structural verification was performed via 2D NMR spectroscopy (Figure S1, Supporting Information).

As previously reported, CjCst‐II was unable to recognize the α(2,3)‐Neu5Ac moiety on **5** (**GM2**). To clarify the spatial preference of CjCst‐II for either the upper or lower arm, **8‐OMe‐GD1a** (see Figure S2, Supporting Information) was synthesized from **GM1a** via SOPME‐S3^Cj^ with Neu5Ac8Me^[^ ^54^ ^]^ as the donor precursor. When **8‐OMe‐GD1a** was used as an acceptor in the SOPME‐S3,8 catalytic module, no product was observed by TLC (Figure S2, Supporting Information). This suggested that CjCst‐II prefers the α2,3‐Neu5Ac moiety on the upper β‐arm over the lower α‐arm. These limitations were attributed to the steric hindrance imposed by GalNAc and Galβ(1,3)GalNAc. Although CjCst‐II has been used in the assembly of α(2,8)‐glycosidic bonds on the upper β‐arm of GD1a‐like molecules^[^ ^26^ ^]^ and monosialosides serve as better acceptors than disialosides,^[^ ^55^ ^]^ our attempts at CjCst‐II‐catalyzed sialylation proved elaborate because of its hydrolysis activity. Moreover, to obtain a reasonable yield of the desired product featuring Neu5Ac‐α2,8‐Neu5Ac‐α2,3‐ moiety at the upper arm, the reaction necessitated the use of high concentrations of CjCst‐II within a shorter reaction time (1 h). Starting from **15** (**GD1a**), we afforded **19** (**GT1a**) in a moderate yield (59%). However, **20** (**GQ1b**), synthesized from **16** (**GT1b**), was isolated with a 19% yield due to the purification challenges from further sialylated products and the recovery of ≈ 40% of **16**. Notably, a small amount (≈10% yield) of **17** (**GQ1c**) was generated, indicating that the upper Neu5Ac acceptor might be more favorable than the bottom disialic acid acceptor due to reduced steric hindrance. Consequently, the transformation of **10** (**GM1b**) to **21** (**GD1c**) proceeded in higher yield (67%) with recovery of 29% of the starting material. Similarly, the synthesis of **22** (**GP1c**) presented considerable challenges due to the intrinsic activity of CjCst‐II and the high polarity of **GP1c**, which made monitoring the reaction progress difficult. SOPME‐S3,8 was applied on **17** (**GQ1c**) and prolonged the reaction time exceeding 10 h led to the formation of hydrolysis products, resulting in the loss of Neu5Ac. Following a 4‐h reaction period, the mixture contained at least four sialosides bearing three to six sialic acids, including **17** (**GQ1c**) and **22** (**GP1c**) (putative products) as the major components, after DEAE separation (Figure S3, Supporting Information). Based on previous studies indicating that mono‐Neu5Ac is a better acceptor than di‐Neu5Ac for CjCst‐II, the pentasaccharide was designed as the targeted **GP1c** (in a yield of 20%). To validate the structure of **GP1c**, a comparison of the ^13^C NMR spectra of the synthesized ganglioside glycans was conducted (see below). Notably, **GP1c** is labile and gradually undergoes hydrolysis during both the separation process and the NMR analysis.

**Scheme 2:**
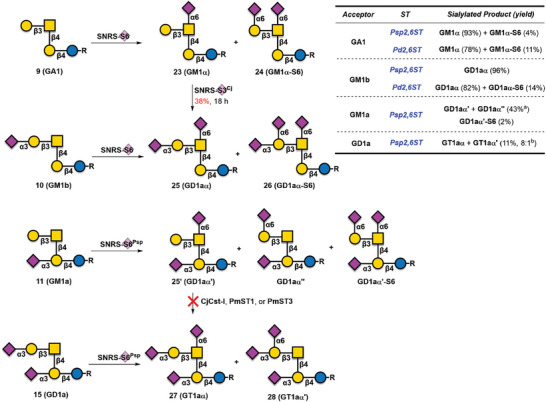
Evaluation of bacterial ST‐catalyzed α2,6‐sialylation of ganglioside glycans for the synthesis of **23** (**GM1**α), **25** (**GD1aα**), and **27** (**GT1aα**). ^a^Isolated yield from the mixture of **GD1aα’** and **GD1aα’’** (the isolated yield of **GD1aα’** was 2%). ^b^Isolated yield from the mixture of regioisomers (ratio of 8:1) (isolated yield of **GT1aα** was 7%).

To achieve α‐series ganglioside glycans, as shown in **Scheme** **2**, synthetic **9** (**GA1**) served as the acceptor substrate for SNRS α(2,6)‐sialylation (SNRS‐S6) using α2,6‐STs from *Photobacterium sp*. (Psp2,6ST)^[^ ^56^ ^]^ or *Photobacterium damselae* (Pd2,6ST),^[^ ^57^ ^]^ leading to the synthesis of **23** (**GM1α**). In the presence of 1.1 equiv. of Neu5Ac as the donor precursor, both enzymes produced monosialylated **GM1α** in yields of 93% and 78%, respectively. A small amount of oversialylation occurred at the C6 hydroxyl group of the terminal Gal, yielding the disialylated product **24** (**GM1α‐S6**) in 4% and 11% yields, respectively. To confirm that sialylation occurred at C6 of the internal GalNAc, a detailed 2D NMR technique was applied (Figure S4, Supporting Information) to determine the structure.

Next (Scheme 2), **23** (**GM1α**) was utilized as an acceptor for the synthesis of **25** (**GD1aα**). We found that employing SNRS‐S3^Cj^ sialylation to the terminal Gal of **23** (**GM1α**) produced only **25** (**GD1aα**) in 38% yield, indicating that **23** (**GM1α**) was not a good acceptor for CjCst‐I. However, the successful assembly of Neu5Acα2,3 Gal glycosidic bonds on **GM1α** by CjCst‐I further confirmed the original location of α(2,6)‐Neu5Ac at the internal GalNAc site. It is known that Neu5Acα2,6 Gal cannot serve as the acceptor for CjCst‐I‐catalyzed α2,3‐sialylation.^[^ ^28^ ^]^ To further confirm the structure of **GD1aα** and increase its yield, **10** (**GM1b**) was used as the starting material in SNRS‐S6. Previous observations indicated that Psp2,6ST was sensitive to α(2,3)‐Neu5Ac on the Galβ(1,3)GalNAc acceptor and incorporated Neu5Ac at the C6 position of the internal GalNAc.^[^ ^28^ ^]^ As expected, **GD1aα** was obtained in 96% yield from this reaction. Intriguingly, Pd2,6ST‐catalyzed sialylation, similar to the reaction catalyzed by Psp2,6ST, produced **GD1aα** in 82% yield. However, the trisialylated byproduct (**26** (**GD1aα‐S6**)) was also obtained in 14% yield. The structure of **25** (**GD1aα**) was confirmed by 2D NMR spectroscopy. In addition, we found that both Psp2,6ST and Pd2,6ST showed poor regioselectivity between the Gal and GalNAc residues of **15** (**GD1a**), forming these regio‐isomers in an ≈ 8:1 ratio in favor of the desired **27** (**GT1aα**) (by Psp2,6ST). When **11** (**GM1a**) was used as an acceptor for Psp2,6ST‐catalyzed sialylation, two disialylated products (**GD1aα’** and **GD1aα’’** as the major products) and a trisialylated product (**GD1aα’‐S6** as the minor product) were produced in lower yields. These results indicated that the presence of Neu5Ac at lower arm disrupted the catalytic efficiency and selectivity of Psp2,6ST. Notably, sialylation of Neu5Acα(2,3)Galβ(1,3)GalNAcβ(1,3)Galα(1,4)Lac (SSEA‐4) by the same enzymes resulted in only 24% Neu5Ac at the internal GalNAc, whereas Pd2,6ST resulted in Neu5Ac being placed at the nonreducing end of Gal in almost quantitative yield.^[^ ^28^ ^]^ However, when Pd2,6ST was used for sialylation of the disaccharide Galβ(1,3)GalNAcβ‐linker, nearly equal amounts of monosialylation at Gal (32%) and GalNAc (34%) were observed.^[^ ^58^ ^]^ These results revealed that the structure of the acceptor could significantly affect the regioselectivity of Pd2,6ST and Psp2,6ST. Alternatively, attempts were made to use CjCst‐I‐, PmST1‐, or PmST3^[^ ^59^ ^]^‐catalyzed sialylation to assemble Neu5Ac on the nonreducing Gal of **GD1aα’**, but these efforts proved futile. Compared with **GM1α**, **GD1aα’** with an additional Neu5Ac on the lower α‐arm inhibited the catalytic activity of the α(2,3)STs tested.

**Figure 2:**
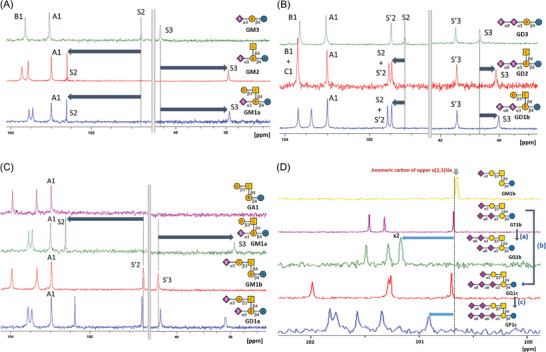
Comparison of the ^13^C NMR data of: A) **GM3**, **GM2**, and **GM1a**; B) **GD3**, **GD2**, and **GD1b**; C) **GA1**, **GM1a**, **GM1b**, and **GD1a**; and D) the anomeric carbons in the sialic acid residues of **GM1b**, **GT1b**, **GQ1b**, **GQ1c**, and **GP1c**. A, B, C, D, S, and S’ represent Glc, Gal, GalNAc, Gal, α3Neu5Ac at lower arm, and α3Neu5Ac at upper arm (or α8Neu5Ac) moiety, respectively from reducing end. The number indicates the position of the carbon atom. (a) Additional sialic acid attached to upper α(2,3)sia moiety with α2,8‐sialyl linkage (from **GT1b** to **GQ1b**) would shift the anomeric carbon of upper α(2,3)sia into the downfield region. (b) Additional sialic acid attached to lower α(2,8)sia moiety with α2,8‐sialyl linkage (from **GT1b** to **GQ1c**) would not shift the anomeric carbon of upper α(2,3)sia into the downfield region. (c) ^13^C NMR spectra of **GQ1c** in comparison with that of **GP1c**, original anomeric carbon of upper α(2,3)sia was shifted to the downfield region, agreeing with the results observed from (a) and (b) and confirming the sialyl linkages of **GP1c**.

The previous study of chemical shifts of GM3, GM2, and GM1a indicated that the presence of β(1,4)GalNAc induced a downfield shift in C2 and an upfield shift in C3‐Neu5Ac of ≈1.8 and 2.7 ppm, respectively.^[^ ^60^ ^]^ In addition, the carbon at position 2 of the newly formed α(2,8)Neu5Ac residue exhibited a greater downfield shift than that of α(2,3)Neu5Ac.^[^ ^55^ ^]^ The variations in the ^13^C chemical shifts of GM3, GM2, GM1a, GM1b, GD3, GD2, GD1b, and GD1a are consistent with the reported tendencies, as shown in **Figure** **2**A–C. The conversion of **16** (**GT1b**) to **20** (**GQ1b**) was associated with a downfield shift in the upper carbon C2 of α(2,3)Neu5Ac (Figure 2D). A similar downfield shift was also observed for **20** (**GQ1b**) vs **17** (**GQ1c**). When the chemical shift of **22** (**GP1c**) was compared with that of **GQ1c**, **GP1c** exhibited a downfield shift of the upper α(2,3)Neu5Ac anomeric carbon. This confirmed the formation of a new α(2,8) glycosidic bond on the upper branch of **GQ1c**, indicating that the synthesized compound was in fact **GP1c**.

**Figure 3:**
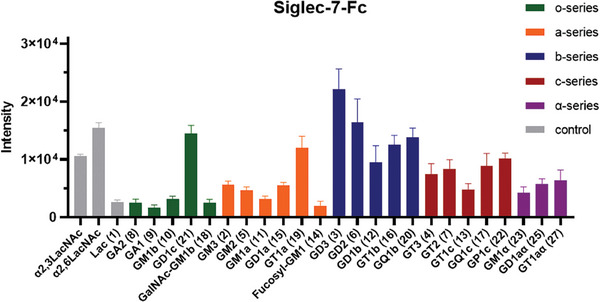
Binding of human Siglec‐7‐Fc to the ganglioside glycan microarray. Binding signals were quantified by fluorescence intensity, as shown in the bar graphs in the panels, which represent the means ± SDs. b‐Series glycans that contain the GD3 motif presented higher binding affinities than other glycans with similar numbers of Neu5Ac residues.

**Figure 4:**
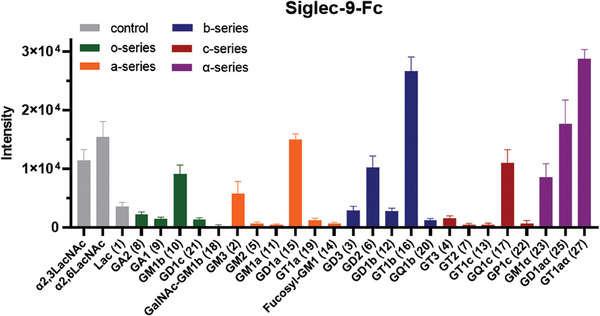
Binding of human Siglec‐9‐Fc to the ganglioside glycan microarray. Binding signals were quantified by fluorescence intensity, as shown in the bar graphs in the panels, which represent the means ± SDs.

To probe the binding affinities toward synthetic ganglioside glycans, human recombinant Siglec‐7‐Fc and Siglec‐9‐Fc fusion proteins were investigated.^[^ ^61^ ^]^ Strong interactions between human Siglecs and sialylated glycans play crucial roles in immune regulation.^[^ ^62^ ^]^ Siglec‐7 and Siglec‐9 are found on natural killer cells and have garnered significant attention since they were recently revealed as immune checkpoints.^[^ ^63^, ^64^ ^]^ In this study, the synthesized ganglioside glycans were used to construct a glycan microarray, which was then used to investigate the interactions between the glycans and Siglecs. By exploiting the existing azidohexyl spacer on the synthesized glycans, a microarray was constructed by direct printing on an alkynylated silver‐coated cuprous oxide nanoparticle (Cu_2_O@Ag) glass slide through a Cu(I)‐free click reaction.^[^ ^65^ ^]^ The microarrays were exposed to a premix solution containing recombinant human Fc‐fused Siglec‐7 or Siglec‐9 (Siglec‐7‐Fc or Siglec‐9‐Fc, 5 µg mL^−1^) and an Fc‐ specific anti‐human antibody labeled with Cy3 (5 µg mL^−1^) to visualize the binding signals (**Figures** **3** and **4**).

Compared with the α2,3‐ and α2,6‐sialosides linked to terminal Gal in poly‐LacNAc, Siglec‐7‐Fc has superior binding affinity toward α2,8α2,3‐sialosides.^[^ ^30^, ^31^ ^]^ This α2,8‐sialyl linkage is also found extensively in b‐series gangliosides, c‐series gangliosides, GD1c, and GT1a. Although many studies have reported analyses of the binding of Siglec‐7‐Fc with different natural and artificial glycan ligands,^[^ ^28^, ^66^, ^67^, ^68^, ^69^ ^]^ a comprehensive investigation of ganglioside glycans has not been performed. The binding preference of Siglec‐7‐Fc toward our synthetic ganglioside glycans is shown in Figure 3. Surprisingly, GD3 exhibited the strongest binding affinity with Siglec‐7‐Fc among all the ganglioside glycans, whereas increasing the number of Neu5Ac residues resulted in a decrease in the binding affinity (**3** vs **4**; **6** vs **7**; and **12** vs **13**). In general, the glycans with α2,8α2,3 disialo moieties (**21**, **19**, **6**, **12**, **16**, and **20**) presented relatively high binding affinities. In general, b‐series gangliosides presented higher affinities than the glycans in the other series did, and in comparison with **3**, the weaker affinities of the b‐series gangliosides resulted from the presence of a sugar in the upper arm. The monosialo‐gangliosides presented relatively low binding affinities with Siglec‐7‐Fc in the order of **2** > **5** > **23** > **11** ≈ **10** > **18** > **14**. Clearly, the glycan extension in the upper arm spatially shields α2,3‐sialylation in the lower arm. Additionally, **23**, which contains an inner α(2,6)‐sialic acid, produced a stronger binding signal than **10** and **11** but a weaker signal than **2** and **5**. The fact that Neu5Acα(2,6)GalNAc can enhance the binding affinity was consistent with a previous report.^[^ ^67^ ^]^ The addition of one Neu5Ac, e.g., disialyl glycan **15**, restored affinity (**15** > **11** ≈ **10**). This observation also held true for **25** versus **23**. For disialoglycans, disialosides containing Neu5Acα(2,8)Neu5Ac (**3** > **6** > **21** > **12**) showed higher affinities than did glycans with two separated mono‐Neu5Ac residues (**25** and **15**). Moreover, the glycans with the Neu5Acα(2,8)Neu5Ac motif in the lower arm (**3** and **6**) displayed better affinities than the glycan with this motif in the upper arm (**21**). However, **21** was preferred over **12** because of the steric hindrance caused by the sugar in the upper arm. For the trisialylated glycans, **19** and **16** displayed comparable affinities with Siglec‐7 and higher affinities than the linear trisialosides **4**, **7**, and **13**. **GT3** exhibited dramatically lower binding affinity than **GD3**, indicating that the extra α2,8Neu5Ac group diminishes the affinity (also **7** vs **6** and **13** vs **12**). Among the hypersialylated (more than three sialic acids) glycans, **20**, bearing two sets of Neu5Acα(2,8)Neu5Ac motifs, presented slightly greater binding affinities than the other glycans but lower binding affinities than the glycans with one Neu5Acα(2,8)Neu5Ac motif, such as **3**, **6** and **21**. A multivalent effect was not expected to be observed the interaction between **GQ1b**/**GP1c** and Siglec‐7‐Fc. These results also revealed that the binding affinities of glycans with linear trisialic acid motifs were lower than those of their Neu5Acα(2,8)Neu5Ac parents. Overall, **3** presented the highest binding affinity with Siglec‐7‐Fc among the tested ganglioside glycans.

Siglec‐9 has high sequence similarity with siglec‐7 but displays different specificities, preferring terminal α2,3‐ or α2,6‐sialic acid moieties.^[^ ^16^, ^67^, ^69^ ^]^ Recent glycan microarray binding studies revealed that Siglec‐9 trended toward sialyl Lewis X, along with an additional preference for 6‐sulfated GlcNAc (sialyl 6‐sulfo Lewis X).^[^ ^69^ ^]^ However, little information is available regarding the binding specificity between Siglec‐9 and gangliosides due to the absence of a complete ganglioside glycan library. The results with Siglec‐9‐Fc and our synthetic glycans of gangliosides are depicted in Figure 4. In contrast to the binding signals observed with Siglec‐7‐Fc, Siglec‐9‐Fc exhibited more selective binding. Surprisingly, **27** displayed superior binding signals with Siglec‐9‐Fc. **GT1aα** contains the Neu5Acα2,3Galβ1,3(Neu5Acα2,6)GalNAc motif, which can mimic sialyl 6‐sulfo Lewis X because of the negative charges (carboxylic acid and sulfate groups) located in a similar region. Intriguingly, a clear binding preference was observed in favor of multiple separate sialic acids, and the addition of extra Neu5Ac into the upper arm decreased the binding affinity. Moreover, the results revealed that α2,3Nue5Ac in the upper arm was more crucial than that in the lower arm (**10** vs **11**; **15** vs **11**; **16** vs **12**; and **17** vs **13**). Increasing the presence of Neu5Ac in the lower arm resulted in increased binding affinity (**10** < **15** < **16**) but reduced affinity with tri‐Nue5Ac (**17**). Among the evaluated ganglioside glycans, **GT1b** and **GT1aα** presented the highest binding affinities with Siglec‐9‐Fc.

## 3 Conclusion

In summary, this work presents a highly versatile and efficient enzymatic synthetic approach for generating a broad spectrum of natural ganglioside glycans in different series with various molecular structures. The synthesis involves elongating a simple Lac‐βO(CH_2_)_6_N_3_ substrate through the sequential action of GTs via an SNRS or SOPME. Subsequent chemical or enzymatic removal of the sialoside moieties from **5** (**GM2**) or **11** (**GM1a**) yielded asialoganglioside glycans **8** (**GA2**) and **9** (**GA1**), respectively. Further enzymatic extension led to the creation of an extensive array of ganglioside glycans. In the course of this study, Li and co‐workers successfully generated an extensive library of ganglioside glycans through chemoenzymatic synthesis.^[^ ^70^ ^]^ However, the enzymology of the bacterial GTs with respect to ganglioside glycan structures had not been thoroughly examined. Notably, the discovery that the bacterial α2,6‐ST (Pd2,6ST or Psp2,6ST) can regioselectively add α(2,6)Neu5Ac to internal GalNAc residue, thereby producing α‐series ganglioside glycans, presents a more cost‐effective alternative for large‐scale synthesis compared to using the expensive mammalian sialyltransferase (ST6GalNAc5) and time consuming chemoenzymatic synthesis. The synthesized compounds were employed in microarray format to assess their binding specificity with Siglec‐7 and Siglec‐9, offering insights into their potential applications in biological studies and therapeutic development. The microarray binding results revealed that the specific ligands for Siglec‐7‐Fc and Sigelec‐9‐Fc are **3** (**GD3**) and **27** (**GT1aα**), respectively. This finding may provide suitable ligands for studies of the roles of these Siglecs in immunomodulation. In addition, the ability to systematically synthesize such a diverse collection of ganglioside glycans is invaluable for studying their roles in glycobiology.

## Conflict of Interest

The authors declare no conflict of interest.

## Supporting information



Supporting Information

## Data Availability

The data that support the findings of this study are available in the supplementary material of this article.
