# Supplementary material for: Comprehensive Modular Synthesis of Ganglioside Glycans and Evaluation of their Binding Affinities to Siglec‐7 and Siglec‐9
Source: Adv Sci (Weinh). 2024 Nov 18;12(2):2412815. doi: 10.1002/advs.202412815 (PMC11727393; doi:10.1002/advs.202412815)
Supplement: Supplementary file 1 — Supporting Information [file ADVS-12-2412815-s001.pdf]

ADVANCED SCIENCE
Open Access

Supporting Information

for *Adv. Sci.*, DOI 10.1002/advs.202412815

Comprehensive Modular Synthesis of Ganglioside Glycans and Evaluation of their Binding Affinities to Siglec-7 and Siglec-9

*Avijit K. Adak, Hsin-Kai Tseng, Shu-Yen Chang, Yu-Ching Chiang, Ke-Hong Lyu, Yun-Sheng Lee, Wen Lu, Wen-Hua Kuo, Takashi Angata and Chun-Cheng Lin**

Supporting information

Comprehensive Modular Synthesis of Ganglioside Glycans and Evaluation of Their Binding Affinities to Siglec-7 and Sigelc-9

Avijit K. Adak,[a,†] Hsin-Kai Tseng,[a,†] Shu-Yen Chang,[a] Yu-Ching Chiang,[a] Ke-Hong Lyu,[a] Yun-Sheng Lee,[a] Wen Lu,[a] Wen-Hua Kuo,[a] Takashi Angata,[b] Chun-Cheng Lin [a,c,]*

[a]Department of Chemistry, National Tsing Hua University, Hsinchu 30013, Taiwan

[b]Institute of Biological Chemistry, Academia Sinica, Taipei 11529, Taiwan

[c]Department of Medicinal and Applied Chemistry, Kaohsiung Medical University, Kaohsiung 80708, Taiwan

*E-Mail: cclin66@mx.nthu.edu.tw

Table of Contents

Materials and method 3

Cloning and Overexpression of Enzyme 3

Figure S1. 5

Figure S2. 6

Figure S3. 7

Figure S4. 8

General Procedure 8

Synthetic procedures and characterization of new compounds 12

Reference 35

NMR Spectra 37

Materials and method

All chemicals were purchased as reagent grade and used without further purification. Matrixes of bacterial cultural media were purchased from BD Bioscience, Neogen, and Sigma Aldrich. Chemical competent *E. coli* BL21(DE3) was purchased from Yeastern Biotechnology (Taipei, Taiwan). Chitin beads used in IMPACT™ system and amylose beads used in MBP system were purchased from New England Biolabs. His-tag purification resin was purchased from Roche. *N*-Acetylneuraminic acid (Sialic acid) and neuraminidase from *Arthrobacter ureafaciens* were purchased from Nacalai Tesque, Inc. (Kyoto, JAPAN). Phosphoenolpyruvic acid (PEP) was purchased from Alfa Aesar. Pyruvate kinase (PK) from rabbit muscle was purchased from Calzyme. Size exclusion column chromatography was performed by polyacrylamide gel (Bio-Gel P-2) with deionized H_2O. Reactions were monitored with analytical thin layer chromatography (TLC) on Merck silica gel 60 F_{254} plates (0.25 mm) and visualized under UV (254 nm) and staining with *p*-anisaldehyde. C18 reverse-phase silica column (Sep-Pak Vac C18 cartridge 20 c.c./5 g 37-55 µm, Waters) and C18 reverse-phase silica gel (LiChroprep® RP-18 (40-63 µm), Merck) were used to perform reverse-phase column chromatography with methanol, and deionized H_2O. DEAE anion-exchange resin (GE Healthcare Life Sciences) was used to perform anion-exchange chromatography with $NaCl_{(aq)}$, and deionized H_2O. ^1H and ^{13}C NMR spectra were recorded by Bruker AV-400, AV-600, AV-850, Varian-Unity INOVA-500, or VNMRS-700 NMR spectrometers, respectively. Chemical shift (δ) was reported in ppm as units and coupling constant (J) was reported in hertz (Hz) as unit. D_2O (4.79 ppm at 298 K) was used as internal standards in ^1H NMR spectra and CD_3OD (49.0 ppm) was used as internal standards in ^{13}C NMR spectra. ^1H NMR spectra were reported in the order of chemical shift, multiplicity, coupling constant, and number of protons. 2D NMR (COSY, HSQC, HMBC, or HSQC-TOCSY) experiments were used to assist assignment of the products. Multiplicities were reported using the following abbreviations: s = singlet, d = doublet, t = triplet, q = quartet, m = multiplet. High-resolution mass spectra were obtained by ESI-TOF mass spectrometry. Protein molecular weight standards was purchased from Thermo Fisher Scientific (Waltham, MA, USA). The protein concentrations were determined with the Bradford Protein Assay (Bio-Rad) and Pierce™ BCA protein assay kit (Thermo Fisher Scientific) using bovine serum albumin as the standard. Protein purification devices were using centrifugal filter devices (Vivaspin® Turbo 15 10 kDa MWCO, Satorius).

Cloning and Overexpression of Enzyme

Enzyme Resources. BlNahK (*N*-acetylhexosamine 1-kinase from *Bifidobacterium longum*),[1] EcGlmU (*N*-acetylglucosamine 1-phosphate uridyltransferase from *Escherichia coli*),[2] AGX1 (recombinant human UDP-GalNAc pyrophosphorylase),[3] CjCgtA (β1,4-*N*-galactosaminyltransferase from *Campylobacter jejuni* OH4384),[4] MtGalK (galactokinase from *Meiothermus taiwanensis* sp. nov. WR-220),[5] AtUSP (uridine diphosphate-sugar pyrophosphorylase from *Arabidopsis thaliana*),[6] CjCgtB (β1,3-galactosyltransferase from *Campylobacter jejuni* OH4384),[4] HiLgtD (β1,3-*N*-galactosaminyltransferase/

galactosyltransferase from *Haemophilus influenzae*),[7] IP (inorganic pyrophosphatase from *E. coli* MG1655 ATCC 700926),[8] BfFKP (fucokinase/L-fucose-1-P-guanylyltransferase from *B. fragilis* NCTC 9343),[9] FutC (α1,2-fucosyltransferase from *H. pylori* ATCC 26695),[7] NmCSS (CMP-sialic acid synthetase from *Neisseria meningitidis*),[10] PmST1 M144D (*N*-terminal amino acid 2-25 truncated α2,3-sialyltransferase 1 M144D mutant from *Pasteurella multocida*),[11] CjCst-I (α2,3-sialyltransferase from *Campylobacter jejuni*),[12] PmST3 (*C*-terminal 35 amino acids truncated *Pm1174* encoding α2,3-sialyltransferase from *Pasteurella multocida*),[13] Psp2,6ST (α2,6-sialyltransferase from *Photobacterium sp. JH-ISH-224*),[14] Pd2,6ST (α2,6-sialyltransferase from *Photobacterium damsela*),[1] CMK (cytidine monophosphate kinase from *E. coli* MG1655 ATCC 700926),[8] SpNanA (neuraminidase from *Streptococcus pneumoniae* R6)[15] were cloned, overexpressed, and purified as previously reported procedures. The plasmids pCWmalE-thrombin respectively containing the *cgtA* and *cgtB* gene were generously provided by Prof. Stephen G. Withers.

Protein overexpression and purification for *E. coli* overexpression system. The protein overexpression procedure is similarly as previous report.[1] Positive plasmid was selected and subsequently transformed into *E. coli* BL21(DE3) chemical competent cells. The *E. coli* BL21 (DE3) harboring the recombinant plasmid was grown in LB or TB rich medium containing ampicillin (100 μg/mL) or kanamycin (50 μg/mL) at 37 °C until the OD_{600} reached 0.5-0.8. Protein expression was then induced by adding 0.1-0.5 mM of IPTG followed by incubation at 16-20 °C for 16-24 h with vigorous shaking at 200 rpm in a shaking incubator (Firstek S300R). The bacterial cells were harvested by centrifugation at 4 °C and 5000 x g for 15 min. The enzyme purifications followed similar procedures as reported previously for each respective enzyme.

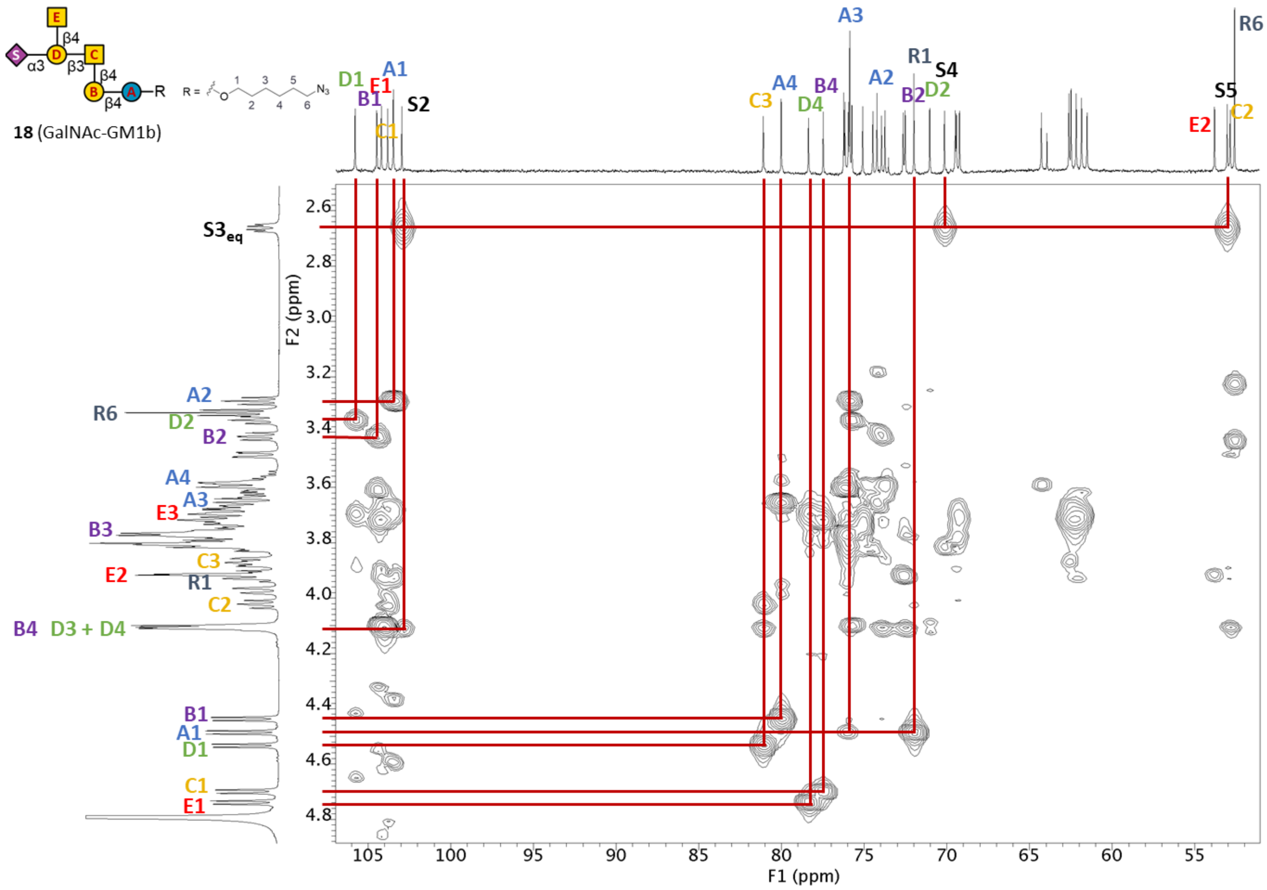

Figure S1. ^{1}H-^{13}C HMBC analysis of **GalNAc-GM1b**. Glycans A, B, C, D, E, and S represent Glc, Gal, GalNAc, Gal, GalNAc, and α3Neu5Ac, respectively from reducing end. The number indicates the position of the carbon or proton atom. Cross signals for the anomeric protons and glycosidic linkages adjacent carbons were observed (B1/A4, C1/B4, D1/C3, and E1/D4). The cross signal for the anomeric carbon (S2) and C-3 proton of Gal (D3) was observed (S2/D3). These were in line with the structure of **GalNAc-GM1b**.

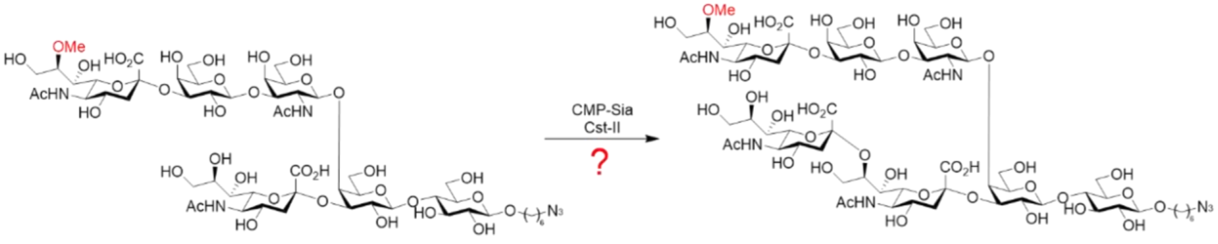

8-OMe-GD1a

1	GD1a + 1 eq. CMP-Sia
2	co-spot of lane 1 and lane 3
3	GD1a α-2,8-sialylation
4	8-OMe-GD1a + CMP-Sia
5	Co-spot of lane 4, 6, 7, 8
6	8-OMe-GD1a reacted with 1 eq. CMP-Sia
7	8-OMe-GD1a reacted with 5 eq. CMP-Sia
8	8-OMe-GD1a reacted with 10 eq. CMP-Sia

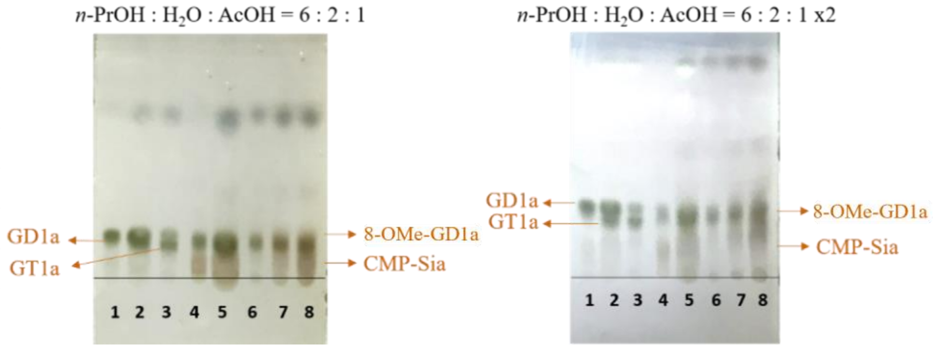

Figure S2. 8-OMe-GD1a was attempted to be catalyzed by using CjCst-II-catalyzed α2,8-sialylation. After incubation for 3 h, the reaction was monitored by TLC analysis (*n*-PrOH/ H_2O/ AcOH = 6/2/1 (v/v/v) or *n*-PrOH/ H_2O/ AcOH = 6/2/1 (v/v/v), run twice; stained with *p*-anisaldehyde)). For lane 1-3, **GD1a** was successfully transformed into **GT1a** catalyzed by CjCst-II using 1.0 equivalent of CMP-sia as donor. For lane 4-8, **8-OMe-GD1a** was not served as a suitable acceptor for CjCst-II using 1.0 to 10.0 equivalent of CMP-sia as donor, suggesting that CjCst-II cannot elongate the α2,3-Neu5Ac moiety at lower α arm.

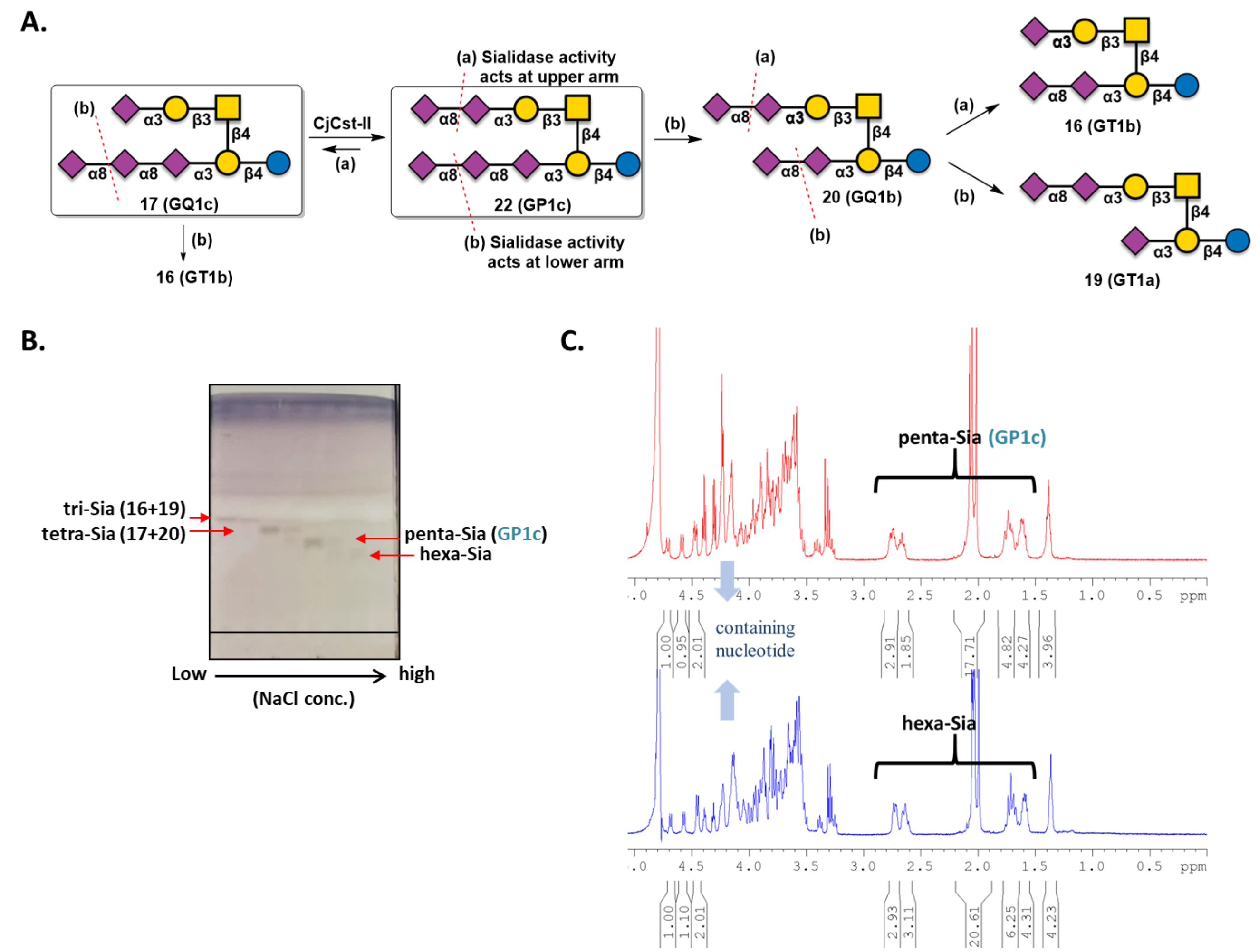

Figure S3. (A) Proposed sialylated products after CjCst-II-catalyzed α2,8-sialylation on **GQ1c**. Sialic acid residues were cleaved from upper arm (pathway (a)) or lower arm (pathway (b)) by its intrinsic sialidase activity to give tetra-sialylated products (**17** and **20**) and tri-sialylated products (**16** and **19**). (B) Purification of sialylated prdoucts by anion exchange chromatography (using DEAE Sepharose) was monitored by TLC (*n*-PrOH/ H_2O/ 25%NH_4OH = 6/2/1 (v/v/v), stained with *p*-anisaldehyde). (C) Parital ^1H NMR (500 MHz, D_2O) spectrum of penta-sialylated product and hexa-sialylated product. The integrals of H3 signals approximately at 2.8-2.6 ppm (for equatorial protons of sialic acids), along with signals arising from H3 protons at around 1.8-1.6 ppm (for axial protons of sialic acids) manifested that the numbers of sialic acids on the two sialosides were five and six, respectively.

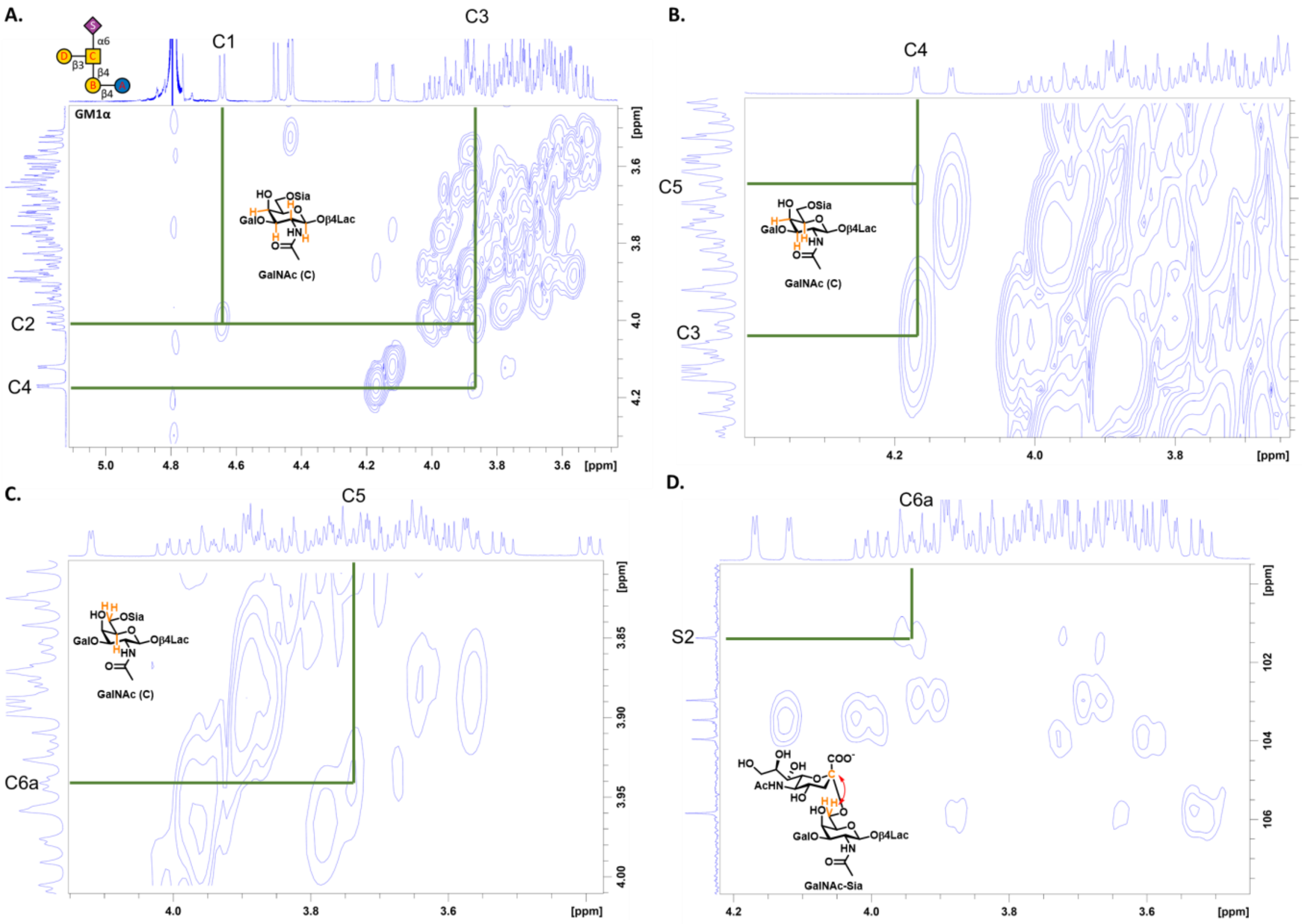

Figure S4. Two-dimensional NMR analysis of **GM1α**. Glycans A, B, C, D, and S represent Glc, Gal, GalNAc, Gal, and α6Neu5Ac, respectively from reducing end. The number indicates the position of the carbon or proton atom. (A) Cross signals were identified for the protons on GalNAc residue (C1/C2, C2/C3, and C3/C4) by using ^{1}H-^{1}H COSY spectra. (B) Cross signals were identified for H3 and H5 protons within H4 proton on GalNAc residue (C3/C4 and C4/C5) by using ^{1}H-^{1}H COSY spectra. (C) Cross signal was identified for H5 within one of H6 protons on GalNAc residue (C5/C6a) by using ^{1}H-^{1}H COSY spectra. (D) ^{1}H-^{13}C HMBC analysis of **GM1α**. A cross signal for the anomeric carbon of sialic acid (S2) and one of the H6 protons of GalNAc (C6a) was observed (S2/C6a). This suggests that the glycosidic linkage is a 2→6 (GalNAc) linkage.

General Procedure

General Procedure for Sequential One Pot Multi-enzymatic α-2,3-Sialylation (SOPME-S3)

SOPME-S3^{Cj} and **SOPME-S3^{Pm}** (using CjCst-I and PmST1 M144D as α-2,3-ST, respectively); Taking the synthesis of **2 (GM3)** as an example:

A HEPES buffer (100 mM, pH 8.5) solution containing Neu5Ac (40 mM), CTP (50 mM), and $MgCl_2$ (50 mM) was incubated with agitation at 450 rpm at 37 °C in the presence of CMP-sialic acid synthetase CSS

(0.1 mg/mL) and inorganic pyrophosphatase IP (0.1 mg/mL) for 3 h. When the completion of the reaction as indicated by the disappearance of Neu5Ac and the formation of CMP-Neu5Ac on thin-layer chromatography (TLC) (*n*-PrOH/ H_2O/AcOH = 6/ 2/ 1 (v/ v/ v), $R_f = 0.10$), 20 mM of **1** (**Lac**) was added to the reaction and the pH value was adjusted to 8.5 by adding 2N NaOH. The reaction was incubated at 37 °C in the presence of α2,3-sialyltransferase CjCst-I (0.2 mg/mL) or PmST1 M144D (0.3 mg/mL).

General Procedure for Enzymatic α-2,3-Sialylation by Sugar Nucleotide Regeneration System (SNRS-S3)

<u>**SNRS-S3^{Cj}** (CjCst-I as α-2,3-ST)**;** Taking the synthesis of **2 (GM3)** as an example:</u>

The pH of a buffer solution (100 mM Tris-HCl) containing 20 mM of **1** (**Lac**), 25 mM of Sia, 0.25 mM of ATP, 0.625 mM of CTP, 55 mM of PEP, 20 mM of $MgCl_2$ was adjusted to 8.2 by adding 2N $NaOH_{(aq)}$. To the above solution were added 0.1 mg/mL of NmCSS, 0.1 mg/mL of CMK, 0.04 mg/ml of IP, 3.3 U/mL of PK, and 0.1 mg/mL of CjCst-I. The solution was incubated at 27 °C. More enzymes were added if necessary. The reaction progression was monitored by TLC analysis and stained with *p*-anisaldehyde stain.

General Procedure for Sequential One Pot Multi-enzymatic α-2,3-/α-2,8-Sialylation (SOPME-S3,8)

<u>Taking the synthesis of **3 (GD3)** as an example:</u>

A HEPES buffer (50 mM, pH 8.0) solution containing Neu5Ac (6.4 mM), CTP (8 mM) and $MgCl_2$ (20 mM) was incubated with agitation at 450 rpm at 37 °C in the presence of CMP-sialic acid synthetase CSS (0.1 mg/mL) and inorganic pyrophosphatase IP (0.1 mg/mL) for 3 h. When the completion of the reaction as indicated by the disappearance of Neu5Ac and the formation of CMP-Neu5Ac on thin-layer chromatography (TLC) (*n*-PrOH/ H_2O/AcOH = 6/ 2/ 1 (v/ v/ v), $R_f = 0.10$), 8 mM of **2** (**GM3**) was added to the reaction and the pH value was adjusted to 8.5 by adding 2N NaOH. The reaction was incubated at 37 °C in the presence of α2,3/8-sialyltransferase CjCst-II (0.1 mg/mL).

General Procedure for Enzymatic α-2,3-/α-2,8-Sialylation by Sugar Nucleotide Regeneration System (SNRS-S3,8)

<u>Taking the synthesis of **3 (GD3)** as an example:</u>

The pH of a buffer solution (100 mM Tris-HCl) containing 5 mM of **2** (**GM3**), 5 mM of Neu5Ac, 0.05 mM of ATP, 0.5 mM of CTP, 12 mM of PEP, and 10 mM of $MgCl_2$ was adjusted to 8.5 by adding 2N $NaOH_{(aq)}$. To the above solution were added 0.1 mg/mL of NmCSS, 0.1 mg/mL of CMK, 0.1 mg/ml of IP, 3.3 U/mL of PK, and 0.1 mg/mL of CjCst-II. The solution was incubated at 37 °C. More enzymes were added if necessary. The reaction progression was monitored by TLC analysis and stained with *p*-anisaldehyde stain.

General Procedure for Sequential One Pot Multi-enzymatic β-1,4-*N*-acetylgalactosaminylation (SOPME-G4)

<u>Taking the synthesis of **5** (**GM2**) as an example:</u>

A HEPES buffer (50 mM, pH 8.0) solution containing GalNAc (10 mM), ATP (16 mM), UTP (16 mM),

and $MgCl_2$ (20 mM) was incubated with agitation at 450 rpm at 37 °C in the presence of BlNahK (0.1 mg/mL) for 5 h. When the completion of the reaction as indicated by the disappearance of GalNAc and the formation of GalNAc-1-P on TLC (*n*-PrOH/ H_2O/AcOH = 6/ 2/ 1 (v/ v/ v), R_f = 0.30), the reaction mixture was adjusted to 7.5 by adding 2N NaOH. The reaction was incubated with agitation at 450 rpm at 37 °C in the presence of AGX1 (0.1 mg/mL) and IP (0.1 mg/mL) for 2 h. After the formation of UDP-GalNAc (*n*-BuOH/ H_2O / AcOH = 2/ 1/ 1 (v/ v/ v), R_f = 0.20), **2** (**GM3**) (8 mM) was added to the reaction and then the pH value was adjusted to 7.5 by adding 2N NaOH. The reaction was incubated with agitation at 450 rpm at 37 °C in the presence of CjCgtA (0.2 mg/mL) and more enzymes were added if necessary.

General Procedure for Enzymatic β-1,4-*N*-acetylgalactosaminylation by Sugar Nucleotide Regeneration System (SNRS-G4)

<u>Taking the synthesis of **5** (**GM2**) as an example:</u>

The pH of a buffer solution (100 mM Tris-HCl) containing 15 mM of **2** (**GM3**), 20 mM of GalNAc, 0.2 mM of ATP, 0.5 mM of UTP, 44 mM of PEP, 20 mM of $MgCl_2$ was adjusted to 8.0 by adding 2N $NaOH_{(aq)}$. To the above solution were added 0.1 mg/mL of BlNahK, 0.01 mg/mL of AGX1, 0.08 mg/ml of IP, 2.6 U/mL of PK, and 0.2 mg/mL of CjCgtA. The solution was incubated at 27 °C. More enzymes were added if necessary. The reaction progression was monitored by TLC analysis and stained with *p*-anisaldehyde stain.

General Procedure for Sequential One Pot Multi-enzymatic β-1,3-galactosaminylation (SOPME-G3)

<u>Taking the synthesis of **11** (**GM1a**) as an example:</u>

A Tris-HCl buffer (100 mM, pH 9.0) solution containing Gal (10 mM), ATP (15 mM), and $MgCl_2$ (40 mM) was incubated with agitation at 450 rpm at 37 °C in the presence of MtGalK (0.1 mg/mL) for 4 h. When the completion of the reaction as indicated by the disappearance of Gal and the formation of Gal-1-P on TLC (*n*-PrOH/ H_2O/AcOH = 6/ 2/ 1 (v/ v/ v), R_f = 0.30), the reaction mixture was adjusted to 7.5 by adding 2N NaOH. The reaction was incubated with agitation at 450 rpm at 37 °C in the presence of AtUSP (1 mg/mL). After the formation of UDP-Gal (*n*-BuOH/ H_2O / AcOH = 2/ 1/ 1 (v/ v/ v), R_f = 0.20) reached 50%, **5** (**GM2**) (10 mM) was added to the reaction and the pH value was adjusted to 7.5 by adding 2N NaOH. The reaction was incubated with agitation at 450 rpm at 37 °C in the presence of CjCgtB (0.2 mg/mL) and more enzymes were added if necessary.

General Procedure for Enzymatic β-1,3-galactosaminylation by Sugar Nucleotide Regeneration System (SNRS-G3)

<u>Taking the synthesis of **11** (**GM1a**) as an example:</u>

The pH of a buffer solution (50 mM HEPES) containing 15 mM of **5** (**GM2**), 16 mM of Gal, 0.16 mM of

ATP, 0.4 mM of UTP, 32 mM of PEP, 10 mM of $MgCl_2$ was adjusted to 8.0 by adding 2N $NaOH_{(aq)}$. To the above solution were added 0.3 mg/mL of MtGalK 0.3 mg/mL of AtUSP, 0.08 mg/ml of IP, 3.8 U/mL of PK, and 0.3 mg/mL of CjCgtB. The solution was incubated at 27 °C. More enzymes were added if necessary. The reaction progression was monitored by TLC analysis and stained with *p*-anisaldehyde stain.

General Procedure for Enzymatic α-2,6-Sialylation by Sugar Nucleotide Regeneration System (SNRS-S6)

SNRS-S6^{Pd} and **SNRS-S6^{Psp}** (Pd2,6ST and Psp2,6ST as α-2,6-ST, respectively); Taking the synthesis of **23 (GM1*α*)** as an example:

The pH of a buffer solution (100 mM Tris-HCl) containing 10 mM of **9** (**GA1**), 11 mM of Sia, 0.11 mM of ATP, 0.28 mM of CTP, 22 mM of PEP, 20 mM of $MgCl_2$ was adjusted to 8.5 (for Pd2,6ST) or 8.2 (for Psp2,6ST) by adding 2N $NaOH_{(aq)}$. To the above solution were added 10 mM of acceptor, 0.3 mg/mL of NmCSS, 0.1 mg/mL of CMK, 0.1 mg/ml of IP, 13 U/mL of PK, and 0.3 mg/mL of Pd2,6ST or Psp2,6ST. The solution was incubated at 27 °C. More enzymes were added if necessary. The reaction progression was monitored by TLC analysis and stained with *p*-anisaldehyde stain.

General Purification Procedure 1

If there is no regioisomer formed in enzymatic glycosylation, the reaction mixture containing sialylated products is suitable for this purification procedure. The enzymatic reaction was quenched by addition of the same reaction volume of EtOH. The reaction solution was centrifuged (10,000 x g, 10 min) to remove enzymes and insoluble precipitates. The supernatant was collected, filtered (0.45-μm PVDF filter; Millipore), and then concentrated. The resulting residue was purified by a C18 reverse-phase silica column (Sep-Pak Vac C18 cartridge 5 g, 55-105 μm, Waters) using a solution followed by stepwise elution of 0-100% MeOH in H_2O as eluent (increase 10% MeOH per step, 20 mL eluent for each step). The resulting residue was purified by anion exchange chromatography (DEAE Sepharose Fast Flow resin, GE Healthcare). The column was washed with water (10 mL) followed by the elution with a gradient of aqueous sodium chloride (10-200 mM, increase 10 mM for each gradient and 10 mL for each gradient). The fractions containing product were pooled, concentrated, and then purified by size-exclusion column using H_2O as eluent (BioGel P2 gel packed in a column 1.5 cm x 50 cm or 1.5 cm x 100 cm, Biorad).

General Purification Procedure 2

If there are regio-isomers formed in enzymatic sialylation, the reaction mixture is suitable for this purification procedure. Follow the same procedure as described in general purification procedure 1, but the residues after C-18 reverse-phase silica column were pooled and purified by Xbridge® BEH Amide OBD™ Prep Column (10 x 250 mm, 130Å, 5μm) with a flow rate of 1.7 mL/min. HPLC purification was monitored by ELSD (evaporative light scattering detector), and fractions were analyzed by TLC. After pooled and concentrated the solution containing product, further desalting procedure was applied by a

size-exclusion chromatography (Bio Gel P-2 gel packed in column, 1.5 cm x 50 cm, Bio-Rad) with distilled water as eluent.

HPLC mobile phase condition for **27** (**GT1aα**) and **28** (**GT1aα'**):

Using an elution of 62% acetonitrile in water containing 100 mM ammonium formate (pH = 3.45) for 30 mins. Retention times for **27** and **28** were at 22.36 min and 21.00 min respectively.

Synthetic procedures and characterization of new compounds

Compounds **1**, **2**, **3**, and **4** were reported previously.[16]

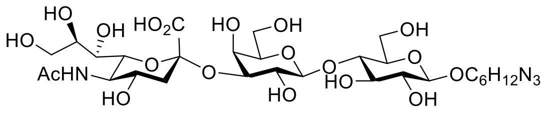

2

Compound 2 (GM3).

Using SOPME system:

1 (**Lac**) (1.0 equiv.) and Neu5Ac (2.0 equiv.) were used as acceptor and donor precursor respectively by following the general enzymatic synthetic procedure **SOPME-S3^{Cj}** or **SOPME-S3^{Pm}**. After being shaken for 4 h (CjCst-I) or 2 h (PmST1 M144D), the reaction was purified by following general purification procedure 1 (without anion exchange chromatography). After purification, the product was lyophilized to give **2** (**GM3**) in 96% (160 mg) or 93% (13 mg).

Using SNRS system:

1 (**Lac**) (1.0 equiv.) and Neu5Ac (1.25 equiv.) were used as acceptor and donor precursor respectively by following the general enzymatic synthetic procedure **SNRS-S3^{Cj}**. After being shaken for 29 h, the reaction was purified by following general purification procedure 1 (without anion exchange chromatography). After purification, the product was lyophilized to give **2** (**GM3**) in 99% (444 mg). R_f = 0.55 (*n*-PrOH/ H_2O/AcOH = 6/ 2/ 1 (v/ v/ v)).

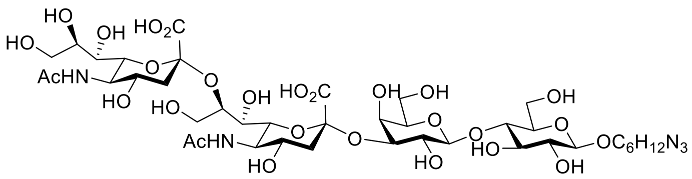

3

Compound 3 (GD3).

Using SOPME system:

2 (**GM3**) (1.0 equiv.) and Neu5Ac (0.8 equiv.) were used as acceptor and donor precursor respectively by following the general enzymatic synthetic procedure **SOPME-S3,8**. After being shaken for 3 h, the reaction was purified by following general purification procedure 1. After purification, the product was lyophilized to give **3** (**GD3**) in 72% (89% based on equivalent of Neu5Ac) (37 mg) and **4** (**GT3**) in 6% (4 mg).

Using SNRS system:

2 (**GM3**) (1.0 equiv.) and Neu5Ac (1.0 equiv.) were used as acceptor and donor precursor respectively by following the general enzymatic synthetic procedure **SNRS-S3,8**. After being shaken for 10 h, the reaction was purified by following general purification procedure 1. After purification, the product was lyophilized to give **3** (**GD3**) in 68% (9.4 mg). R_f= 0.38 (*n*-PrOH/ H_2O/AcOH = 6/ 2/ 1 (v/ v/ v)).

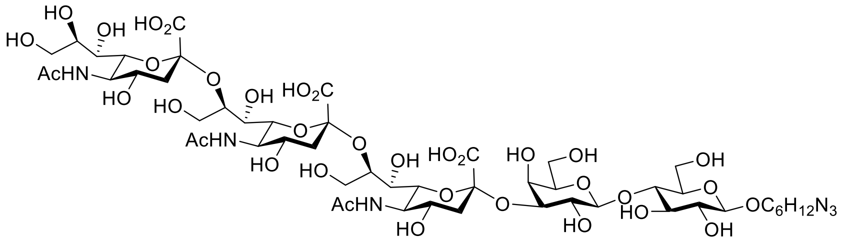

4

Compound 4 (GT3).

Synthesis from **2** (**GM3**):

2 (**GM3**) (1.0 equiv.) and Neu5Ac (2.5 equiv.) were used as acceptor and donor precursor respectively by following the general enzymatic synthetic procedure **SOPME-S3,8**. After being shaken for 8 h, the reaction was purified by following general purification procedure 1. After purification, the product was lyophilized to give **4** (**GT3**) in 43% (17 mg), **3** (**GD3**) in 39% (12 mg), and **GQ3** in 6% (3 mg).

Synthesis from **3** (**GD3**):

3 (**GD3**) (1.0 equiv.) and Neu5Ac (1.0 equiv.) were used as acceptor and donor precursor respectively by following the general enzymatic synthetic procedure **SOPME-S3,8**. After being shaken for 4 h, the reaction was purified by following general purification procedure 1. After purification, the product was lyophilized to give **4** (**GT3**) in 36% (26 mg), **2** (**GD3**) in 49% (28 mg), and **GQ3** in 5% (5 mg). R_f = 0.26 (*n*-PrOH/ H_2O/AcOH = 6/ 2/ 1 (v/ v/ v)).

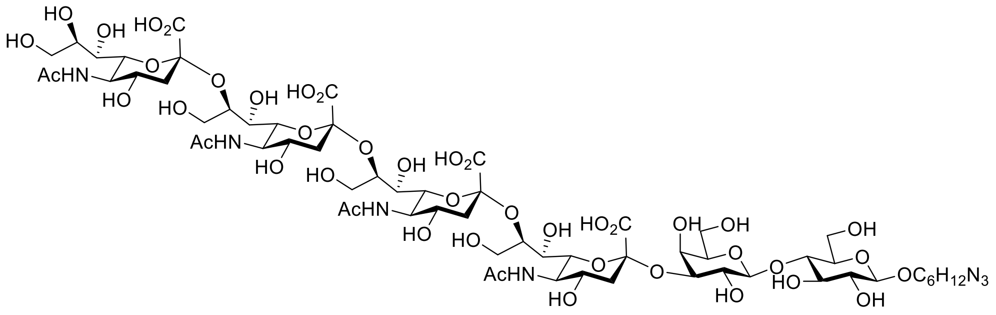

GQ3

GQ3. R$_f$ = 0.14 (*n*-PrOH/ H_2O/AcOH = 6/ 2/ 1 (v/ v/ v)); ^{1}H NMR (500 MHz, D_2O) δ 4.49 (d, J = 7.9 Hz, 1H), 4.46 (d, J = 8.0 Hz, 1H), 4.17-4.04 (m, 7H), 3.98 (d, J = 11.2 Hz, 1H), 3.94-3.91 (m, 2H), 3.90-3.87 (m, 4H), 3.84-3.51 (m, 29H), 3.32-3.27 (m, 3H), 2.75 (dd, J = 4.4, 12.3 Hz, 1H), 2.68-2.65 (m, 3H), 2.06 (s, 6H), 2.05 (s, 3H), 2.01 (s, 3H), 1.75-1.69 (m, 4H), 1.65-1.57 (m, 4H), 1.38 (brs, 4H); ^{13}C NMR (125 MHz, D_2O) δ 176.0, 175.92, 175.90 (2C), 174.50, 174.48, 174.2, 174.1, 103.6, 103.0, 102.0, 101.9, 101.3, 101.1, 79.4, 79.0, 78.8, 78.7, 76.5, 76.2, 75.8, 75.3, 74.7, 74.5, 74.2, 73.8, 73.6, 72.7, 71.5, 70.4, 70.3, 70.2, 69.9, 69.5, 69.2 (2C), 69.1, 69.0, 68.5, 63.5, 62.4, 62.3, 62.2, 62.0, 61.0, 53.4, 53.35, 53.32, 52.7, 52.1, 41.4, 41.0 (2C), 40.6, 29.5, 28.8, 26.6, 25.6, 23.4 (2C), 23.3, 23.0; HRMS (ESI-TOF): m/z of $C_{62}H_{100}N_7O_{43}^-$ [M-H]$^-$: calculated 1630.5854, found 1630.5857.

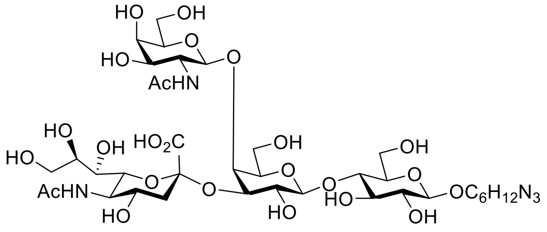

5

Compound 5 (GM2).

Using SOPME system:

2 (**GM3**) (1.0 equiv.) and GalNAc (1.25 equiv.) were used as acceptor and donor precursor respectively by following the general enzymatic synthetic procedure **SOPME-G4**. After being shaken for 14.5 h, the reaction was purified by following general purification procedure 1 (without anion exchange chromatography). After purification, the product was lyophilized to give **5** (**GM2**) in 90% (45 mg).

Using SNRS system:

2 (**GM3**) (1.0 equiv.) and GalNAc (1.33 equiv.) were used as acceptor and donor precursor respectively by following the general enzymatic synthetic procedure **SNRS-G4**. After being shaken for 38 h, the reaction was purified by following general purification procedure 1 (without anion exchange chromatography). After purification, the product was lyophilized to give **5** (**GM2**) in 96% (259 mg). R$_f$ = 0.37 (*n*-PrOH/ H_2O/AcOH = 6/ 2/ 1 (v/ v/ v)); ^{1}H NMR (500 MHz, D_2O) δ 4.73 (d, J = 8.5 Hz, 1H), 4.52

(d, J = 7.9 Hz, 1H), 4.47 (d, J = 8.0 Hz, 1H), 4.15-4.11 (m, 2H), 3.97 (d, J = 12.1 Hz, 1H), 3.94-3.85 (m, 4H), 3.83-3.76 (m, 5H), 3.75-3.68 (m, 6H), 3.65- 3.57 (m, 6H), 3.47 (d, J = 10.0 Hz, 1H), 3.36-3.26 (m, 4H), 2.65 (dd, J = 4.3, 12.2 Hz, 1H), 2.02 (s, 3H), 2.01 (s, 3H), 1.92 (dd, J = 12.2, 12.2 Hz, 1H), 1.65-1.59 (m, 4H), 1.39 (brs, 4H); ^{13}C NMR (125 MHz, D_2O) δ176.0, 175.8, 175.0, 103.7, 103.6, 103.0, 102.6, 79.6, 78.1, 75.7 (2C), 75.4, 75.3, 75.0, 74.0, 73.7, 73.2, 72.2, 71.5, 71.0, 69.6, 69.0, 68.7, 63.8, 62.1, 61.5, 61.1, 53.3, 52.6, 52.1, 37.9, 29.5, 28.8, 26.6, 25.6, 23.6, 23.0.; HRMS (ESI-TOF) : m/z of $C_{37}H_{62}N_5O_{24}^-$ $[M-H]^-$: calculated 960.3785, found 960.3794.

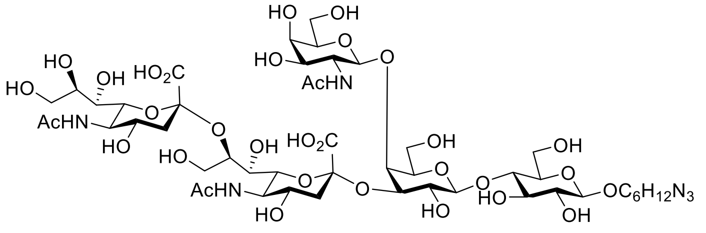

6

Compound 6 (GD2). 3 (**GD3**) (1.0 equiv.) and GalNAc (1.1 equiv.) were used as acceptor and donor precursor respectively by following the general enzymatic synthetic procedure **SOPME-G4**. After being shaken for 15 h, the reaction was purified by following general purification procedure 1. After purification, the product was lyophilized to give **6** (**GD2**) in 88% (35 mg). R_f = 0.22 (n-PrOH/ H_2O/AcOH = 6/ 2/ 1 (v/ v/ v)); ^1H NMR (500 MHz, D_2O) δ 4.69 (d, J = 8.4 Hz, 1H), 4.50 (d, J = 7.9 Hz, 1H), 4.47 (d, J = 8.1 Hz, 1H), 4.18-4.10 (m, 3H), 4.03-3.97 (m, 2H), 3.94-3.90 (m, 3H), 3.88-3.86 (m, 3H), 3.84-3.71 (m, 10H), 3.70-3.64 (m, 6H), 3.63-3.55 (m, 6H), 3.39 (t, J = 8.9 Hz, 1H), 3.33-3.26 (m, 3H), 2.76 (dd, J = 4.3, 12.2 Hz, 1H), 2.67 (dd, J = 4.2, 12.2 Hz, 1H), 2.07 (s, 3H), 2.04 (s, 3H), 2.02 (s, 3H), 1.77 (dd, J = 12.2, 12.2 Hz, 1H), 1.73 (dd, J = 12.2, 12.2 Hz, 1H), 1.65-1.60 (m, 4H), 1.39 (brs, 4H); ^{13}C NMR (125 MHz, D_2O) δ175.9, 175.85, 175.77, 174.34, 174.29, 103.7 (2C), 103.0, 101.53, 101.46, 79.3 (2C), 76.9, 75.7, 75.5, 75.4, 75.3, 75.2, 74.7, 73.8, 73.6, 72.7, 71.8, 71.4, 70.7, 70.2, 69.4, 69.1 (2C), 68.7, 63.5, 62.4, 61.9, 61.6, 61.0, 53.4, 53.3, 52.7, 52.1, 41.4, 40.1, 29.5, 28.8, 26.6, 25.6, 23.5, 23.3, 23.0.; HRMS (ESI-TOF) : m/z of $C_{48}H_{79}N_6O_{32}^-$$[M-H]^-$: calculated 1251.4739, found 1251.4737.

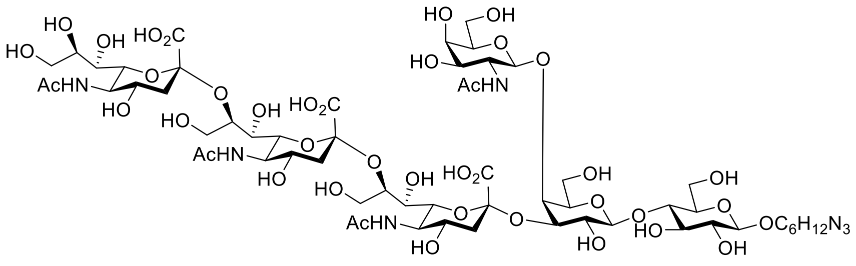

7

Compound 7 (GT2). 4 (**GT3**) (1.0 equiv.) and GalNAc (2.0 equiv.) were used as acceptor and donor precursor respectively by following the general enzymatic synthetic procedure **SOPME-G4**. After being

shaken for 22 h, the reaction was purified by following general purification procedure 1. After purification, the product was lyophilized to give **6** (**GD2**) in 82% (25 mg). R_f = 0.16 (*n*-PrOH/ H_2O/AcOH = 6/ 2/ 1 (v/ v/ v)); ^1H NMR (500 MHz, D_2O) δ 4.69 (d, J = 8.4 Hz, 1H), 4.49 (d, J = 8.2 Hz, 1H), 4.47 (d, J = 8.1 Hz, 1H), 4.18-4.13 (m, 4H), 4.06-3.97 (m, 120 3H), 3.93-3.85 (m, 6H), 3.84-3.72 (m, 11H), 3.70-3.61 (m, 10H), 3.59-3.54 (m, 5H), 3.38 (t, J = 8.9 Hz, 1H), 3.34-3.26 (m, 3H), 2.75 (dd, J = 4.3, 12.2 Hz, 1H), 2.68-2.63 (m, 2H), 2.07 (s, 3H), 2.05 (s, 3H), 2.03 (s, 3H), 2.02 (s, 3H), 1.78 (dd, J = 12.1, 12.1 Hz, 1H), 1.73 (dd, J = 12.0, 12.0 Hz, 1H), 1.69 (dd, J = 12.2, 12.2 Hz, 1H), 1.64-1.58 (m, 4H), 1.39 (brs, 4H); ^{13}C NMR (125 MHz, D_2O) δ175.9 (2C), 175.83, 175.76, 174.5, 174.3, 174.0, 103.7 (2C), 103.0, 102.0, 101.6, 101.3, 79.4 (2C), 78.7, 77.1, 75.7, 75.44, 75.40, 75.35, 75.2, 74.7, 74.3, 73.8, 73.6, 72.7, 71.9, 71.4, 70.7, 70.4, 70.1, 69.5, 69.3, 69.2, 69.1, 68.7, 63.6, 62.5, 62.3, 61.9, 61.6, 61.0, 53.43, 53.39, 53.3, 52.7, 52.1, 41.4, 41.1, 40.0, 29.5, 28.8, 26.6, 25.6, 23.5, 23.4, 23.3, 23.0.; HRMS (ESI-TOF) : m/z of $C_{59}H_{96}N_7O_{40}^-$ $[M-H]^-$: calculated 1542.5693, found 1542.5704.

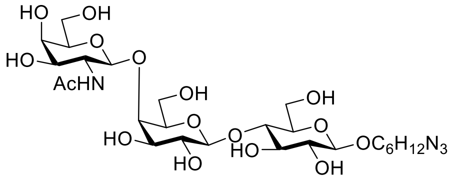

8

Compound 8 (GA2).

<u>Chemical removal of sialic acid from **GM2** under acidic condition:</u>

5 (**GM2**) (10 mg, 0.0104 mmol, 50 mM) was dissolved in 1M formic acid (200 uL) under 80 °C for 2 h, indicating the completion of the reaction by the disappearance of **5** (**GM2**) on TLC (*n*-PrOH/ H_2O/AcOH = 6/ 2/ 1 (v/ v/ v)), the formic acid was removed by high-vacuum evaporator. The resulting residue was purified by following general purification procedure 1 (without anion exchange chromatography). After purification, the product was lyophilized to give **8** (**GA2**) in 49% (3 mg).

<u>Enzymatic removal of sialic acid from **GM2** using AuNA:</u>

To a 1.5 mL PBS buffer solution (pH 6.0) containing **5** (**GM2**) (15 mg, 0.0156 mmol, 10 mM) were added neuraminidase (0.9 U, from Arthrobacter ureafaciens). The solution was incubated with agitation at 350 rpm at 37 °C for 96 h, indicating the completion of the reaction by the disappearance of **5** (**GM2**) and the formation of **8** (**GA2**) on TLC (*n*-PrOH/ H_2O/AcOH = 6/ 2/ 1 (v/ v/ v)), the reaction was quenched and purified by following general purification procedure 1 (without anion exchange chromatography). After purification, the product was lyophilized to give **8** (**GA2**) in 96% (10 mg).

<u>Enzymatic removal of sialic acid from **GM2** using SpNanA:</u>

To a sodium acetate solution (100 mM, pH 6.0, 2.3 mL) containing **5** (**GM2**) (18 mg, 0.0182 mmol, 8 mM) was added sialidase SpNanA (0.8 mg/mL). The reaction mixture was incubated at 37 °C with agitation at 450 rpm for 27 h, indicating the completion of the reaction by the disappearance of **5** (**GM2**) and the formation of **8** (**GA2**) on TLC (*n*-PrOH/ H_2O/AcOH = 6/ 2/ 1 (v/ v/ v)), the reaction was quenched

and purified by following general purification procedure 1 (without anion exchange chromatography). After purification, the product was lyophilized to give **8** (**GA2**) in 97% (12 mg). R_f = 0.65 (*n*-PrOH/ H_2O/AcOH = 6/ 2/ 1 (v/ v/ v)); ^{1}H NMR (500 MHz, D_2O) δ 4.60 (d, J = 8.4 Hz, 1H), 4.46 (d, J = 8.0 Hz, 1H), 4.41 (d, J = 7.8 Hz, 1H), 4.06 (br, 1H), 3.96-3.85 (m, 4H), 3.81-3.69 (m, 8H), 3.67-3.56 (m, 5H), 3.38 (t, J = 8.9 Hz, 1H), 3.31-3.24 (m, 3H), 2.03 (s, 3H), 1.63-1.58 (m, 4H), 1.37 (brs, 4H); ^{13}C NMR (125 MHz, D_2O) δ 175.9, 104.0, 103.7, 103.0, 79.5, 77.1, 75.8, 75.7, 75.4, 75.3, 73.8, 73.4, 72.04, 71.99, 71.5, 68.8, 62.0, 61.6, 61.0, 53.6, 52.1, 29.6, 28.9, 26.6, 25.6, 23.4.; HRMS (ESI-TOF) : m/z of $C_{26}H_{46}N_4O_{16}Na^+$[M+Na]$^+$: calculated 693.2806, found 693.2815.

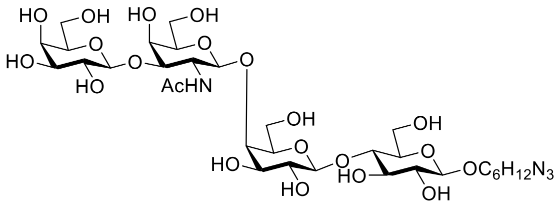

9

Compound 9 (GA1).

<u>Synthesis from **GA2**:</u>

8 (**GA2**) (1.0 equiv.) and Gal (1.1 equiv.) were used as acceptor and donor precursor respectively by following the general enzymatic synthetic procedure **SNRS-G3**. After being shaken for 23 h, the reaction was quenched by following general purification procedure 1. The resulting residue was purified by C18 reverse-phase chromatography and eluted by a gradient from 100% H_2O to 30% methanol in H_2O. Fractions containing **9** (**GA1**) and double galactosylated **8** (**GA2**) were collected and further separated by size-exclusion chromatography (TOYOPEARL HW-40 F-grade packed in column, 1.5 cm x 120 cm, Tosoh Bioscience) with distilled H_2O as eluent. After purification, the product was lyophilized to give **9** (**GA1**) in 73% (12 mg).

<u>Chemical removal of sialic acid from **GM1a** under an acidic condition:</u>

11 (**GM1a**) (10 mg, 0.0089 mmol, 50 mM) was dissolved in 1M formic acid (0.86 mL) under 80 °C for 2 h, indicating the completion of the reaction by the disappearance of **11** (**GM1a**) on TLC (*n*-PrOH/ H_2O/AcOH = 6/ 2/ 1 (v/ v/ v)), the formic acid was removed by high-vacuum evaporator. The resulting residue was purified by following general purification procedure 1 (without anion exchange chromatography). After purification, the product was lyophilized to give **9** (**GA1**) in 63% (5 mg).

<u>Enzymatic removal of sialic acid from **GM1a** using AuNA:</u>

To a 1.5 mL PBS buffer solution (pH 6.0) containing **11** (**GM1a**) (20 mg, 0.0178 mmol, 10 mM) were added neuraminidase (0.9 U, from Arthrobacter ureafaciens). The solution was incubated with agitation at 350 rpm at 37 °C for 96 h, indicating the completion of the reaction by the disappearance of **11** (**GM1a**) and the formation of **9** (**GA1**) on TLC (*n*-PrOH/ H_2O/AcOH = 6/ 2/ 1 (v/ v/ v)), the reaction was quenched

and purified by following general purification procedure 1 (without anion exchange chromatography). After purification, the product was lyophilized to give **9** (**GA1**) in 92% (15 mg).

<u>Enzymatic removal of sialic acid from **GM1a** using SpNanA:</u>

To a sodium acetate solution (100 mM, pH 6.0, 4.8 mL) containing **11** (**GM1a**) (43 mg, 0.0382 mmol, 8 mM) and CMP (0.1 mM)[69,76] was added sialidase SpNanA (0.8 mg/mL). The reaction mixture was incubated at 37 °C with agitation at 450 rpm for 54 h, indicating the completion of the reaction by the disappearance of **5** (**GM2**) and the formation of **8** (**GA2**) on TLC (*n*-PrOH/ H_2O/AcOH = 6/ 2/ 1 (v/ v/ v)), the reaction was quenched and purified by following general purification procedure 1 (without anion exchange chromatography). After purification, the product was lyophilized to give **9** (**GA1**) in 88% (28 mg). R_f = 0.54 (*n*-PrOH/ H_2O/AcOH = 6/ 2/ 1 (v/ v/ v)); ^{1}H NMR (500 MHz, D_2O) δ 4.68 (d, J = 8.5 Hz, 1H), 4.47 (d, J = 8.0 Hz, 1H), 4.44 (d, J = 7.7 Hz, 1H), 4.43 (d, J = 7.8 Hz, 1H), 4.15 (d, J = 2.9 Hz, 1H), 4.10 (d, J = 2.4 Hz, 1H), 4.01 (dd, J = 8.6, 10.5 Hz, 1H), 3.97-3.86 (m, 4H), 3.83-3.71 (m, 9H), 3.70-3.56 (m, 7H), 3.52 (dd, J = 7.9, 9.7 Hz, 1H), 3.40 (dd, J = 8.0, 9.8 Hz, 1H), 3.32 (t, J = 6.9 Hz, 2H), 3.28 (t, J = 8.6 Hz, 1H), 2.03 (s, 3H), 1.64-1.59 (m, 4H), 1.40-1.39 (m, 4H); ^{13}C NMR (125 MHz, D_2O) δ175.9, 105.8, 103.9, 103.3, 103.0, 80.7, 79.5, 77.0, 76.0, 75.7, 75.5, 75.4 (2C), 73.7, 73.43, 73.40, 72.0, 71.6, 71.5, 69.5, 69.0, 62.0 (2C), 61.7, 61.0, 52.5, 52.1, 29.6, 28.8, 26.6, 25.6, 23.4.; HRMS (ESI-TOF) : m/z of $C_{32}H_{56}N_4O_{21}Na^+$[M+Na]$^+$: calculated 855.3335, found 855.3367.

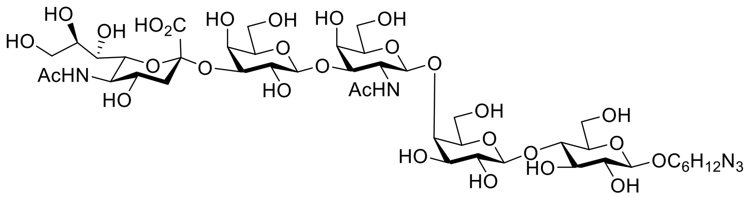

10

Compound 10 (GM1b).

<u>Using SOPME system:</u>

9 (**GA1**) (1.0 equiv.) and Neu5Ac (2.0 equiv.) were used as acceptor and donor precursor respectively by following the general enzymatic synthetic procedure **SOPME-S3**$^{\mathbf{Cj}}$. After being shaken for 2 h, the reaction was purified by following general purification procedure 1 (without anion exchange chromatography). After purification, the product was lyophilized to give **10** (**GM1b**) in 94% (35 mg).

<u>Using SNRS system:</u>

9 (**GA1**) (1.0 equiv.) and Neu5Ac (1.2 equiv.) were used as acceptor and donor precursor respectively by following the general enzymatic synthetic procedure **SNRS-S3**$^{\mathbf{Cj}}$. After being shaken for 4 h, the reaction

was purified by following general purification procedure 1 (without anion exchange chromatography). After purification, the product was lyophilized to give **10** (**GM1b**) in 98% (15 mg). R_f = 0.34 (*n*-PrOH/ H_2O/AcOH = 6/ 2/ 1 (v/ v/ v)); ^{1}H NMR (500 MHz, D_2O) δ 4.70 (d, J = 8.5 Hz, 1H), 4.52 (d, J = 7.8 Hz, 1H), 4.48 (d, J = 8.0 Hz, 1H), 4.44 (d, J = 7.8 Hz, 1H), 4.15 (br, 1H), 4.11 (br, 1H), 4.07 (dd, J = 2.5, 9.9 Hz, 1H), 4.04-3.96 (m, 2H), 3.93- 3.58 (m, 25H), 3.55 (t, J = 9.5 Hz, 1H), 3.41 (t, J = 9.5 Hz, 1H), 3.33 (t, J = 6.9 Hz, 1H), 3.28 (t, J = 8.6 Hz, 1H), 2.75 (dd, J = 4.5, 12.2 Hz, 1H), 2.04 (s, 3H), 2.03 (s, 3H), 1.79 (dd, J = 12.2, 12.2 Hz, 1H), 1.65-1.60 (m, 4H), 1.40 (brs, 4H); ^{13}C NMR (125 MHz, D_2O) δ175.9, 175.8, 174.9, 105.5, 104.0, 103.3, 103.0, 100.6, 80.9, 79.5, 77.0, 76.5, 75.7 (2C), 75.5, 75.4 (2C), 73.8, 73.7, 73.5, 72.8, 72.0, 71.5, 70.0, 69.4, 69.0, 68.9, 68.3, 63.5, 62.0 (2C), 61.7, 61.0, 52.6, 52.3, 52.1, 40.7, 29.5, 28.8, 26.6, 25.6, 23.4, 23.0.; HRMS (ESI-TOF) : m/z of $C_{43}H_{72}N_5O_{29}^-$[M-H]$^-$: calculated 1122.4313, found 1122.4331.

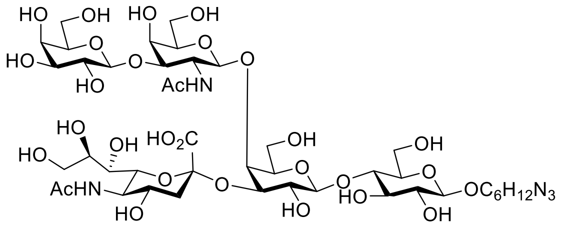

11

Compound 11 (GM1a).

Using SOPME system:

5 (**GM2**) (1.0 equiv.) and Gal (1.0 equiv.) were used as acceptor and donor precursor respectively by following the general enzymatic synthetic procedure **SOPME-G3**. After being shaken for 23 h, the reaction was purified by following general purification procedure 1 (without anion exchange chromatography). After purification, the product was lyophilized to give **11** (**GM1a**) in 92% (50 mg).

Using SNRS system:

5 (**GM2**) (1.0 equiv.) and Gal (1.07 equiv.) were used as acceptor and donor precursor respectively by following the general enzymatic synthetic procedure **SNRS-G3**. After being shaken for 21 h, the reaction was purified by following general purification procedure 1 (without anion exchange chromatography). After purification, the product was lyophilized to give **11** (**GM1a**) in 96% (225 mg). R_f = 0.30 (*n*-PrOH/ H_2O/AcOH = 6/ 2/ 1 (v/ v/ v)); ^{1}H NMR (500 MHz, D_2O) δ 4.75 (d, J = 8.6 Hz, 1H), 4.51 (d, J = 7.2 Hz, 1H), 4.50 (d, J = 7.2 Hz, 1H), 4.46 (d, J = 8.0 Hz, 1H), 4.13-4.11 (m, 3H), 4.03-3.94 (m, 2H), 3.92-3.80 (m, 4H), 3.79-3.64 (m, 16H), 3.62- 3.56 (m, 6H), 3.51-3.45 (m, 2H), 3.35-3.25 (m, 4H), 2.63 (dd, J = 4.4, 12.1 Hz, 1H), 2.01 (s, 3H), 1.98 (s, 3H), 1.90 (dd, J = 12.1, 12.1 Hz, 1H), 1.63- 1.58 (m, 4H), 1.37 (brs,

4H); ^{13}C NMR (125 MHz, D2O) δ175.9, 175.7, 175.0, 105.7, 103.5, 103.4, 103.0, 102.6, 81.3, 79.6, 78.1, 75.8, 75.7, 75.4, 75.3 (2C), 75.0, 74.0, 73.7, 73.4, 73.2, 71.6, 71.4, 71.0, 69.6, 69.5, 69.0, 68.8, 63.8, 62.1, 61.9, 61.5, 61.1, 52.6, 52.12, 52.08, 37.8, 29.6, 28.9, 26.6, 25.6, 23.5, 23.0.; HRMS (ESI-TOF) : m/z of $C_{43}H_{72}N_5O_{29}^-$[M-H]$^-$: calculated 1122.4313, found 1122.4309.

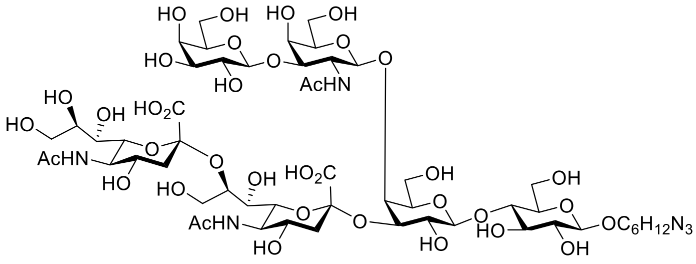

12

Compound 12 (GD1b). 6 (**GD2**) (1.0 equiv.) and Gal (1.0 equiv.) were used as acceptor and donor precursor respectively by following the general enzymatic synthetic procedure **SNRS-G3**. After being shaken for 19 h, the reaction was purified by following general purification procedure 1. After purification, the product was lyophilized to give **12** (**GD1b**) in 93% (21 mg). R_f = 0.18 (*n*-PrOH/ H_2O/AcOH = 6/ 2/ 1 (v/ v/ v)); ^{1}H NMR (500 MHz, D_2O) δ 4.74 (d, *J* = 8.5 Hz, 1H), 4.51 (d, J = 7.6 Hz, 1H), 4.50 (d, J = 7.7 Hz, 1H), 4.47 (d, J = 8.0 Hz, 1H), 4.18-4.15 (m, 3H), 4.10-4.05 (m, 2H), 4.02-3.97 (m, 2H), 3.94-3.78 (m, 10H), 3.76- 3.56 (m, 21H), 3.51 (dd, *J* = 8.4, 9.3 Hz, 1H), 3.39 (dd, *J* = 8.5, 9.2 Hz, 1H), 3.34-3.26 (m, 3H), 2.75 (dd, *J* = 4.3, 12.4 Hz, 1H), 2.66 (dd, *J* = 4.3, 12.4 Hz, 1H), 2.06 (s, 3H), 2.02 (s, 6H), 1.78 (dd, *J* = 12.4, 12.4 Hz, 1H), 1.72 (dd, *J* = 12.4, 12.4 Hz, 1H), 1.64-1.59 (m, 4H), 1.39 (brs, 4H); ^{13}C NMR (125 MHz, D_2O) δ175.93, 175.88, 175.8, 174.3 (2C), 105.6, 103.7, 103.4, 103.0, 101.6, 101.5, 80.8, 79.3, 79.2, 76.9, 75.9, 75.7, 75.5, 75.3, 75.2, 75.1, 74.7, 73.7, 73.6, 73.4, 72.7, 71.6, 71.5, 70.7, 70.1, 69.6, 69.4, 69.1 (2C), 68.8, 63.5, 62.4, 61.9, 61.8, 61.6, 61.0, 53.3, 52.7, 52.3, 52.1, 41.4, 40.0, 29.5, 28.8, 26.6, 25.6, 23.5, 23.3, 23.0.; HRMS (ESI-TOF) : m/z of $C_{54}H_{89}N_6O_{37}^-$[M-H]$^-$: calculated 1413.5267, found 1413.5256.

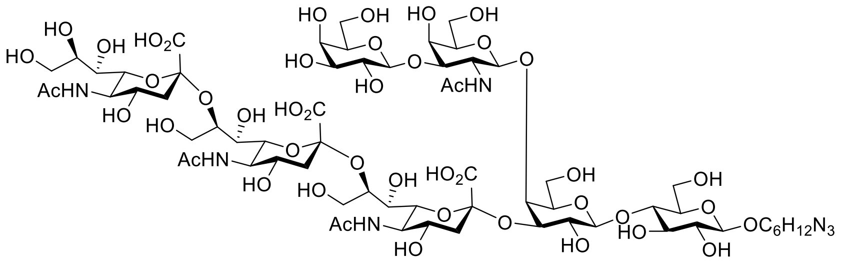

13

Compound 13 (GT1c). 7 (**GT2**) (1.0 equiv.) and Gal (1.0 equiv.) were used as acceptor and donor precursor respectively by following the general enzymatic synthetic procedure **SNRS-G3**. After being shaken for 25 h, the reaction was purified by following general purification procedure 1. After purification, the product was lyophilized to give **13** (**GT1c**) in 92% (24 mg). R_f= 0.14 (*n*-PrOH/ H_2O/AcOH = 6/ 2/ 1 (v/ v/ v)); ^1H NMR (500 MHz, D_2O) δ 4.73 (d, J = 8.5 Hz, 1H), 4.50 (d, J = 8.2 Hz, 1H), 4.48 (d, J = 8.3 Hz, 1H), 4.46 (d, J = 8.1 Hz, 1H), 4.15-4.12 (m, 5H), 4.06-4.01 (m, 2H), 3.99-3.94 (m, 2H), 3.92-3.86 (m, 6H), 3.83- 3.76 (m, 6H), 3.75-3.68 (m, 8H), 3.67-3.61 (m, 8H), 3.60-3.53 (m, 7H), 3.49 (dd, J = 8.1, 9.7 Hz, 1H), 3.37 (dd, J = 8.4, 9.4 Hz, 1H), 3.32-3.25 (m, 3H), 2.74 (dd, J = 4.3, 12.2 Hz, 1H), 2.67-2.62 (m, 2H), 2.06 (s, 3H), 2.04 (s, 3H), 2.00 (s, 6H), 1.78 (dd, J = 12.2, 12.2 Hz, 1H), 1.75-1.65 (m, 2H), 1.63-1.57 (m, 4H), 1.37 (brs, 4H); ^{13}C NMR (125 MHz, D_2O) δ175.90, 175.87 (2C), 175.8, 174.5, 174.4, 174.0, 105.6, 103.7, 103.4, 103.0, 101.9, 101.7, 101.3, 80.9, 79.4, 79.3, 78.7, 77.2, 75.9, 75.7, 75.4, 75.3, 75.2, 75.1, 74.8, 74.3, 73.7, 73.6, 73.4, 72.7, 71.6, 71.4, 70.7, 70.4, 69.9, 69.6, 69.5, 69.3, 69.2, 69.1, 68.8, 63.6, 62.5, 62.3, 61.9, 61.8, 61.6, 61.0, 53.4, 53.3, 52.7, 52.3, 52.1, 41.4, 41.1, 39.8, 29.5, 28.8, 26.6, 25.6, 23.5, 23.4, 23.3, 23.0.; HRMS (ESI-TOF) : m/z of $C_{65}H_{106}N_7O_{45}^-$[M-H]$^-$: calculated 1704.6221, found 1704.6190.

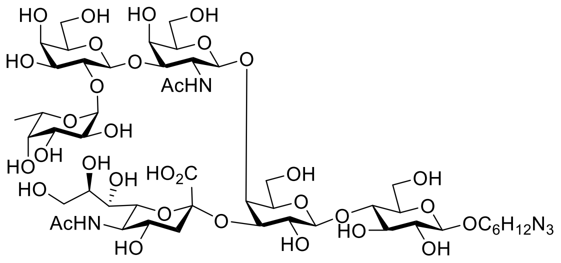

14

Compound 14 (Fucosyl GM1). The pH of a Tris-HCl buffer (100 mM, pH 8.5) (10.5 mL) containing Fuc (7.5 mM), ATP (10 mM), GTP (10 mM), $MgCl_2$ (20 mM) and $MnCl_2$ (20 mM) was adjusted to 7.5 by adding 2N NaOH. Then, FKP (final concentration = 0.5 mg/mL) was added to the above solution. The resulting mixture was incubated with agitation at 450 rpm for 3 h at 37 °C. The formation of GDP-Fuc was monitored by TLC analysis (*n*-PrOH/ H_2O/ AcOH = 6/ 2/ 1 (v/ v/ v), R_f= 0.13). After the completion of the reaction as indicated by the disappearance of Fuc on TLC, **11** (**GM1a**) (11 mg, 0.0089 mmol, 5 mM) was added to the reaction mixture and its pH value was adjusted to 7.5 by adding 2N NaOH. FutC (final concentration = 0.2 mg/mL) was added and the reaction solution was incubated with agitation at 450 rpm at 37 °C. The progress of the reaction was monitored by TLC (*n*-PrOH/ H_2O/ AcOH = 6/ 2/ 1 (v/ v/ v), R_f = 0.32). More enzymes were added if necessary. When TLC indicated the completion of the reaction after 42 h, the reaction was purified by following general purification procedure 1 (without anion exchange chromatography). After purification, the product was lyophilized to give **14** (**Fucosyl GM1a**)

in 95% (12 mg). R_f = 0.32 (*n*-PrOH/ H_2O/ AcOH = 6/ 2/ 1 (v/ v/ v)); ^{1}H NMR (500 MHz, D_2O) δ 5.24 (d, *J* = 4.0 Hz, 1H), 4.69 (d, *J* = 7.7 Hz, 1H), 4.62 (d, *J* = 7.2 Hz, 1H), 4.52 (d, *J* = 7.9 Hz, 1H), 4.49 (d, *J* = 8.0 Hz, 1H), 4.22 (q, *J* = 6.5 Hz, 1H), 4.12 (dd, *J* = 2.5, 10.0 Hz, 1H), 4.08 (dd, *J* = 2.3, 11.8 Hz, 2H), 4.00-3.89 (m, 5H), 3.86-3.52 (m, 29H), 3.38-3.34 (m, 1H), 3.33 (t, *J* = 6.8 Hz, 2H), 3.29 (t, *J* = 8.5 Hz, 1H), 2.68 (dd, *J* = 4.6, 12.3 Hz, 1H), 2.03 (s, 6H), 1.91 (t, *J* = 12.3 Hz, 1H), 1.67-1.59 (m, 4H), 1.42-1.39 (m, 4H), 1.20 (d, *J* = 6.5 Hz, 3H); ^{13}C NMR (100 MHz, D_2O) δ 175.0, 174.2, 173.9, 103.1, 102.5, 102.1, 101.9, 101.3, 99.1, 78.5, 76.9, 76.4, 75.8, 74.8, 74.7, 74.4, 74.2, 74.0, 73.6, 73.0, 72.8, 72.2, 71.8, 70.5, 69.9, 69.5, 69.1, 68.6, 68.4, 68.0, 66.7, 62.8, 61.0, 60.8, 60.4, 60.1, 51.6, 51.4, 51.1 (2C), 37.3, 37.2, 28.5, 27.8, 25.6, 24.5, 22.7, 22.0, 15.3; HRMS (ESI-TOF) : m/z of $C_{49}H_{82}N_5O_{33}$[M-H]$^-$: calculated 1268.4892, found 1268.4887.

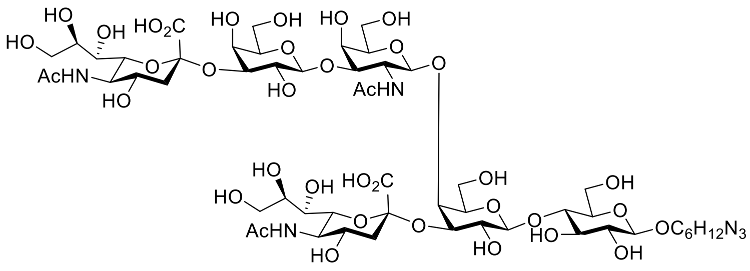

15

Compound 15 (GD1a).

Using SOPME system:

11 (**GM1a**) (1.0 equiv.) and Neu5Ac (2.0 equiv.) were used as acceptor and donor precursor respectively by following the general enzymatic synthetic procedure **SOPME-S3**[Cj]. After being shaken for 8 h, the reaction was purified by following general purification procedure 1. After purification, the product was lyophilized to give **15** (**GD1a**) in 94% (18 mg).

Using SNRS system:

11 (**GM1a**) (1.0 equiv.) and Neu5Ac (1.2 equiv.) were used as acceptor and donor precursor respectively by following the general enzymatic synthetic procedure **SNRS-S3**[Cj]. After being shaken for 19 h, the reaction was purified by following general purification procedure 1. After purification, the product was lyophilized to give **15** (**GD1a**) in 98% (62 mg). R_f = 0.16 (*n*-PrOH/ H_2O/AcOH = 6/ 2/ 1 (v/ v/ v)); ^{1}H NMR (500 MHz, D_2O) δ 4.75 (d, *J* = 8.6 Hz, 1H), 4.59 (d, *J* = 7.8 Hz, 1H), 4.51 (d, *J* = 7.9 Hz, 1H), 4.47 (d, *J* = 8.1 Hz, 1H), 4.14-4.10 (m, 3H), 4.07 (dd, *J* = 2.9, 9.9 Hz, 1H), 4.01 (dd, *J* = 8.8, 10.6 Hz, 1H), 3.96 (d, *J* = 11.3 Hz, 1H), 3.93 (d, *J* = 2.6 Hz, 1H), 3.91-3.52 (m, 32H), 3.48 (dd, *J* = 1.7, 10.0 Hz, 1H), 3.35 (dd, *J* = 8.1, 9.2 Hz, 1H), 3.31 (t, *J* = 6.9 Hz, 2H), 3.27 (t, *J* = 8.6 Hz, 1H), 2.73 (dd, *J* = 4.6, 12.3 Hz, 1H), 2.66 (dd, *J* = 4.6, 12.3 Hz, 1H), 2.01 (s, 6H), 1.99 (s, 3H), 1.90 (dd, *J* = 12.1, 12.1 Hz, 1H), 1.78 (dd, *J* = 12.2, 12.2 Hz, 1H), 1.65-1.57 (m, 4H), 1.38 (brs, 4H); ^{13}C NMR (125 MHz, D_2O) δ 176.0, 175.9, 175.6, 175.0 (2C), 105.4, 103.6, 103.5, 103.0, 102.4, 100.7, 81.4, 79.6, 77.8, 76.4, 75.7, 75.6, 75.4, 75.33,

75.26, 75.0, 74.0, 73.7 (2C), 73.2, 72.8, 71.5, 70.9, 70.1, 69.6, 69.4, 69.1, 69.0, 68.7, 68.4, 63.8, 63.5, 62.1, 61.9, 61.5, 61.1, 52.63, 52.55, 52.1, 52.0, 40.6, 38.2, 29.5, 28.8, 26.6, 25.6, 23.6, 23.0 (2C); HRMS (ESI-TOF) : m/z of $C_{54}H_{89}N_6O_{37}^{-}$[M-H]$^{-}$: calculated 1413.5267, found 1413.5248.

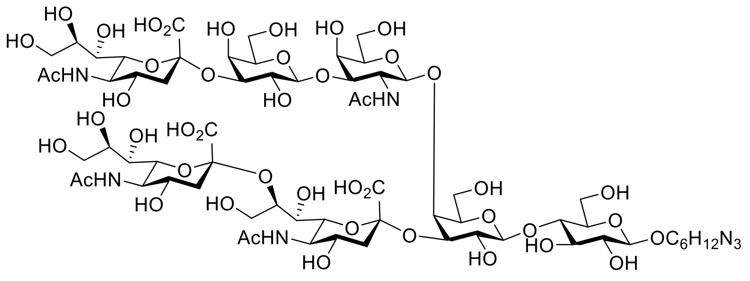

16

Compound 16 (GT1b). 12 (**GD1b**) (1.0 equiv.) and Neu5Ac (2.0 equiv.) were used as acceptor and donor precursor respectively by following the general enzymatic synthetic procedure **SOPME-S3**[Cj]. After being shaken for 8 h, the reaction was purified by following general purification procedure 1. After purification, the product was lyophilized to give **16** (**GT1b**) in 90% (29 mg). R_f = 0.09 (*n*-PrOH/ H_2O/AcOH = 6/ 2/ 1 (v/ v/ v)); ^{1}H NMR (700 MHz, D_2O) δ 4.75 (d, J = 8.5 Hz, 1H), 4.60 (d, J = 7.8 Hz, 1H), 4.51 (d, J = 7.9 Hz, 1H), 4.49 (d, J = 8.1 Hz, 1H), 4.20-4.16 (m, 3H), 4.13 (brs, 1H), 4.10 (dd, J = 2.8, 9.9 Hz, 1H), 4.06 (br, 1H), 4.03- 3.99 (m, 2H), 3.95 (d, J = 2.3 Hz, 1H), 3.94-3.82 (m, 12H), 3.81-3.59 (m, 24H), 3.56 (t, J = 9.3 Hz, 1H), 3.42 (t, J = 9.4 Hz, 1H), 3.33 (t, J = 6.9 Hz, 2H), 3.29 (t, J = 8.5 Hz, 1H), 2.79-3.75 (m, 2H), 2.72 (dd, J = 4.1, 12.3 Hz, 1H), 2.08 (s, 3H), 2.05 (s, 3H), 2.04 (s, 6H), 1.80 (dd, J = 12.3, 12.3 Hz, 1H), 1.77 (dd, J = 12.0, 12.0 Hz, 1H), 1.74 (dd, J = 12.1, 12.1 Hz, 1H), 1.67-1.61 (m, 4H), 1.41-1.40 (m, 4H); ^{13}C NMR (175 MHz, D_2O) δ175.93, 175.90 (2C), 175.7, 174.9, 174.4, 174.2, 105.3, 103.7, 103.4, 103.0, 101.5, 133 101.3, 100.7, 81.0, 79.3, 79.2, 76.5 (2C), 75.7, 75.64, 75.59, 75.3 (2C), 75.1, 74.7, 73.7 (2C), 73.6, 72.8, 72.7, 71.5, 70.6, 70.3, 70.1, 69.5, 69.4, 69.1, 69.0 (2C), 68.6, 68.4, 63.5, 63.4, 62.5, 61.89, 61.87, 61.6, 61.0, 53.3, 52.7, 52.6, 52.12, 52.09, 41.4, 40.6, 40.4, 29.5, 28.8, 26.6, 25.5, 23.5, 23.3, 23.00, 22.99; HRMS (ESI-TOF) : m/z of $C_{65}H_{105}N_7O_{45}^{2-}$[M-2H]$^{2-}$: calculated 1703.6143, found 1703.6060.

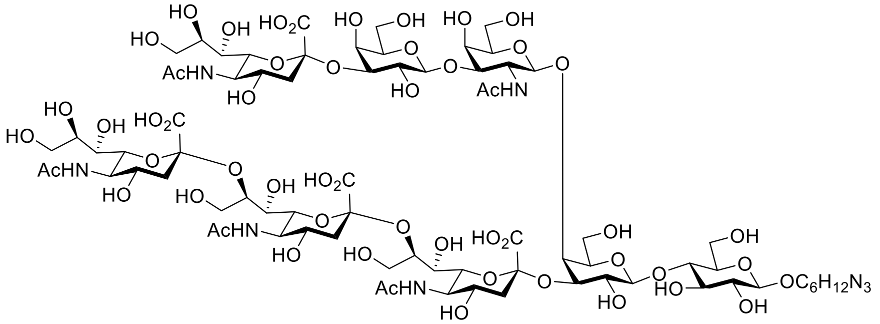

17

Compound 17 (GQ1c). 13 (**GT1c**) (1.0 equiv.) and Neu5Ac (2.0 equiv.) were used as acceptor and donor precursor respectively by following the general enzymatic synthetic procedure **SOPME-S3[Cj]**. After being shaken for 19 h, the reaction was purified by following general purification procedure 1. After purification, the product was lyophilized to give **17** (**GQ1c**) in 72% (19 mg). R_f = 0.18 (*n*-PrOH/ H_2O/AcOH = 6/ 2/ 1 (v/ v/ v), run twice); ^{1}H NMR (500 MHz, D_2O) δ 4.73 (d, *J* = 8.4 Hz, 1H), 4.59 (d, *J* = 7.8 Hz, 1H), 4.49 (d, *J* = 7.5 Hz, 1H), 4.47 (d, *J* = 7.6 Hz, 1H), 4.17- 4.12 (m, 5H), 4.09-3.98 (m, 5H), 3.94-3.56 (m, 41H), 3.54 (t, *J* = 9.4 Hz, 1H), 3.40 (t, *J* = 9.4 Hz, 1H), 3.33-3.26 (m, 3H), 2.77-2.66 (m, 4H), 2.08 (s, 3H), 2.06 (s, 3H), 2.03 (s, 3H), 2.02 (s, 6H), 1.81-1.72 (m, 3H), 1.70 (dd, *J* = 12.1, 12.1 Hz, 1H), 1.64-1.60 (m, 4H), 1.39 (brs, 4H); ^{13}C NMR (125 MHz, D_2O) δ175.92, 175.87 (3C), 175.7, 174.9, 174.5, 174.2, 174.0, 105.3, 103.7, 103.4, 103.0, 102.0, 101.28, 101.26, 100.7, 81.0, 79.4, 79.2, 78.7, 76.5, 76.4, 75.8, 75.7, 75.3 (2C), 75.1, 74.8, 74.2, 73.7 (2C), 73.6, 72.8, 72.7, 71.5, 70.6, 70.3, 70.1 (2C), 70.0, 69.5, 69.4, 69.2 (2C), 69.1, 69.0, 68.6, 68.4, 63.5, 63.4, 62.5, 62.3, 61.88, 61.85, 61.6, 61.0, 53.4, 53.3, 52.7, 52.6, 52.13, 52.08, 41.4, 41.1, 40.6, 40.4, 29.5, 28.8, 26.6, 25.6, 23.5, 23.4, 23.3, 23.0 (2C); HRMS (ESI-TOF): m/z of $C_{76}H_{120}N_8Na_5O_{53}^+$[M-4H+5Na]$^+$: calculated 2107.6429, found 2107.6458.

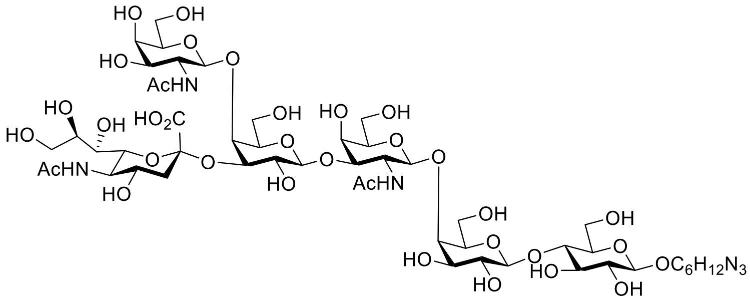

18

Compound 18 (GalNAc-GM1b). 10 (**GM1b**) (1.0 equiv.) and GalNAc (1.2 equiv.) were used as acceptor and donor precursor respectively by following the general enzymatic synthetic procedure **SOPME-G4**. After being shaken for 1 h, the reaction was purified by following general purification procedure 1 (without anion exchange chromatography). After purification, the product was lyophilized to give 18 (**GalNAc-GM1b**) in 87% (13 mg). R_f = 0.23 (*n*-PrOH/ H_2O/AcOH = 6/ 2/ 1 (v/ v/ v)); ^{1}H NMR (700 MHz, D_2O) δ 4.74 (d, *J* = 8.5 Hz, 1H), 4.70 (d, *J* = 8.5 Hz, 1H), 4.53 (d, *J* = 7.9 Hz, 1H), 4.48 (d, *J* = 8.1 Hz, 1H), 4.43 (d, *J* = 7.8 Hz, 1H), 4.11-4.10 (m, 4H), 4.02 (dd, *J* = 8.8, 10.8 Hz, 1H), 3.97 (d, *J* = 11.2 Hz, 1H), 3.94-3.90 (m, 3H), 3.88-3.85 (m, 2H), 3.83-3.56 (m, 25H), 3.48 (dd, *J* = 1.6, 10.3 Hz, 1H), 3.41 (dd, *J* = 7.8, 9.7 Hz, 1H), 3.35 (dd, *J* = 7.9, 9.3 Hz, 1H), 3.33 (t, *J* = 6.9 Hz, 2H), 3.28 (dd, *J* = 8.1, 9.2 Hz, 1H), 2.66 (dd, *J* = 4.6, 12.1 Hz, 1H), 2.04 (s, 3H), 2.03 (s, 3H), 2.02 (s, 3H), 1.91 (dd, *J* = 12.1, 12.1 Hz,

1H), 1.65-1.60 (m, 4H), 1.41-1.40 (m, 4H); ^{13}C NMR (175 MHz, D_2O) δ176.0, 175.9, 175.8, 175.1, 105.3, 104.0, 103.7, 103.3, 103.0, 102.4, 80.6, 79.5, 77.9, 77.0, 75.7, 75.6, 75.44, 75.36 (2C), 75.2, 74.6, 74.0, 73.7, 73.4, 73.2, 72.1, 72.0, 71.5, 70.5, 69.6, 69.0, 68.9, 68.7, 63.8, 62.1, 62.0, 61.7, 61.3, 61.0, 53.3, 52.5, 52.4, 52.1, 38.1, 29.5, 28.8, 26.6, 25.6, 23.6, 23.4, 23.0; HRMS (ESI-TOF): m/z of $C_{51}H_{86}N_6Na_1O_{34}^+$ $[M+Na]^+$: calculated 1349.5083, found 1349.5098.

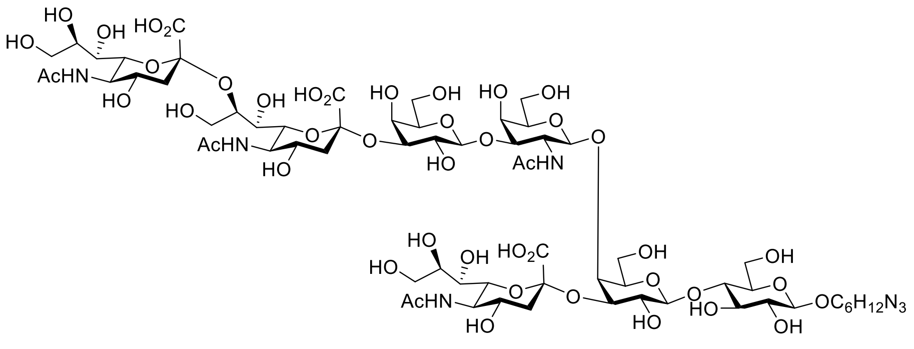

19

Compound 19 (GT1a). 15 (GD1a) (1.0 equiv.) and Neu5Ac (1.1 equiv.) were used as acceptor and donor precursor respectively by following the general enzymatic synthetic procedure **SOPME-S3,8**. After being shaken for 1 h, the reaction was purified by following general purification procedure 1. After purification, the product was lyophilized to give **19** (**GT1a**) in 59% yield (7 mg). R_f = 0.32 (*n*-PrOH/ H_2O/AcOH = 6/ 2/ 1 (v/ v/ v), run twice); ^{1}H NMR (500 MHz, D_2O) δ 4.72 (d, J = 8.5 Hz, 1H), 4.59 (d, J = 7.9 Hz, 1H), 4.49 (d, J = 7.9 Hz, 1H), 4.46 (d, J = 8.1 Hz, 1H), 4.15-4.10 (m, 4H), 4.08 (d, J = 2.3 Hz, 1H), 4.06 (dd, J = 2.6, 10.2 Hz, 1H), 4.01-3.94 (m, 3H), 3.91-3.87 (m, 3H), 3.85-3.55 (m, 33H), 3.53-3.47 (m, 2H), 3.35 (t, J = 8.6 Hz, 1H), 3.29 (t, J = 6.9 Hz, 2H), 3.25 (t, J = 8.5 Hz, 1H), 2.75 (dd, J = 4.5, 12.3 Hz, 1H), 2.68-2.62 (m, 2H), 2.03 (s, 3H), 2.00 (s, 9H), 1.88 (dd, J = 12.3, 12.3 Hz, 1H), 1.72 (dd, J = 12.3, 12.3 Hz, 1H), 1.71 (dd, J = 12.3, 12.3 Hz, 1H), 1.62-1.57 (m, 4H), 1.37 (brs, 4H); ^{13}C NMR (125 MHz, D_2O) δ 176.0, 175.93, 175.90, 175.8, 175.0, 174.6, 174.4, 105.4, 103.54, 103.48, 103.0, 102.2, 101.3, 101.1, 81.2, 79.51, 79.47, 77.5, 76.2, 75.7, 75.5, 75.4, 75.3, 75.2, 75.1, 75.0, 74.0, 73.7, 73.6, 73.1, 72.7, 71.5, 70.9, 70.4, 70.1, 69.6, 69.5, 69.1, 69.0, 68.7, 68.2, 63.8, 63.5, 62.7, 62.1, 62.0, 61.5, 61.0, 53.2, 52.8, 52.6, 52.09, 52.04, 41.5, 40.6, 38.4, 29.5, 28.8, 26.6, 25.6, 23.6, 23.3, 23.0 (2C); HRMS (ESI-TOF) : m/z of $C_{64}H_{106}N_7O_{45}^-$$[M-H]^-$: calculated 1704.6221, found 1704.6200.

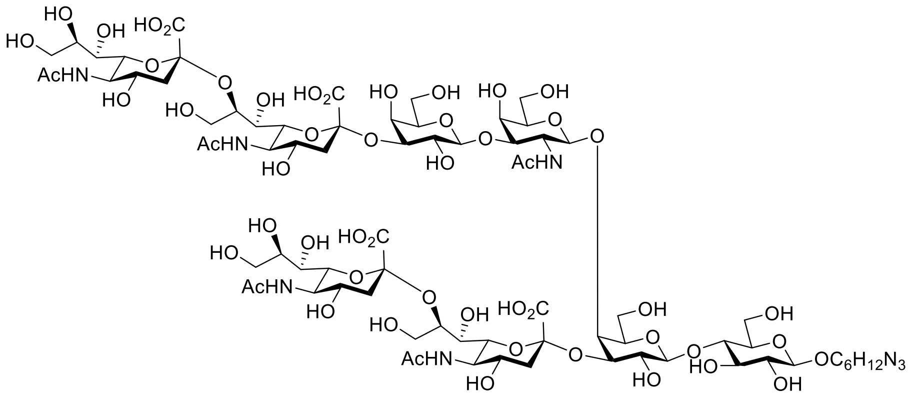

20

Compound 20 (GQ1b). 16 (**GT1b**) (1.0 equiv.) and Neu5Ac (0.95 equiv.) were used as acceptor and donor precursor respectively by following the general enzymatic synthetic procedure **SOPME-S3,8**. After being shaken for 1 h, the reaction was purified by following general purification procedure 1. After purification, the product was lyophilized to give **20** (**GQ1b**) in 19% (20% based on equivalent of Neu5Ac) (6 mg). R_f = 0.09 (*n*-PrOH/ H_2O/AcOH = 6/ 2/ 1 (v/ v/ v)); ^{1}H NMR (500 MHz, D_2O) δ 4.72 (d, J = 8.3 Hz, 1H), 4.59 (d, J = 7.8 Hz, 1H), 4.49 (d, J = 7.8 Hz, 1H), 4.47 (d, J = 7.8 Hz, 1H), 4.19-4.14 (m, 5H), 4.11-4.04 (m, 3H), 4.01-3.96 (m, 3H), 3.93-3.89 (m, 5H), 3.86-3.80 (m, 8H), 3.77-3.52 (m, 28H), 3.41 (t, J = 8.7 Hz, 1H), 3.31 (t, J = 6.9 Hz, 2H), 3.27 (t, J = 8.3 Hz, 1H), 2.77- 2.71 (m, 3H), 2.65 (dd, J = 4.5, 12.2 Hz, 1H), 2.06 (s, 3H), 2.054 (s, 3H), 2.051 (s, 3H), 2.02 (s, 6H), 1.76-1.70 (m, 4H), 1.64-1.59 (m, 4H), 1.39 (brs, 4H); ^{13}C NMR (125 MHz, D_2O) δ 175.9 (4C), 175.8, 174.7, 174.40, 174.36, 174.2, 105.3, 103.7, 103.5, 103.0, 101.5, 101.3, 101.2 (2C), 80.8, 79.5, 79.3, 79.2, 76.3, 76.2, 75.8, 75.7, 75.6, 75.3 (2C), 75.14, 75.06, 74.7, 73.8, 73.6 (2C), 72.71, 72.69, 71.5, 70.5, 70.4, 70.3, 70.1, 69.48, 69.46, 69.11, 69.08, 69.00, 68.97, 68.6, 68.3, 63.5 (2C), 62.6, 62.5, 62.0, 61.9, 61.6, 60.9, 53.3, 53.2, 52.74, 52.72, 52.14, 52.10, 41.5 (2C), 40.6 (2C), 29.5, 28.8, 26.6, 25.6, 23.5, 23.31, 23.27, 23.0 (2C); HRMS (ESI-TOF): m/z of $C_{76}H_{120}N_8Na_5O_{53}{}^+$[M-4H+5Na]$^+$: calculated 2107.6429, found 2107.6491.

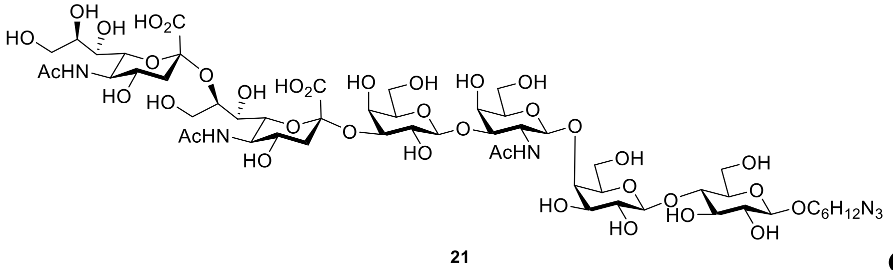

21

Compound 21 (GD1c). 10 (**GM1b**) (1.0 equiv.) and Neu5Ac (0.95 equiv.) were used as acceptor and donor precursor respectively by

following the general enzymatic synthetic procedure **SOPME-S3,8**. After being shaken for 1 h, the reaction was purified by following general purification procedure 1. After purification, the product was lyophilized to give **21** (**GD1c**) in 67% (71% based on equivalent of Neu5Ac) (20 mg). R_f = 0.27 (*n*-PrOH/ H_2O/AcOH = 6/ 2/ 1 (v/ v/ v)); ^{1}H NMR (500 MHz, D_2O) δ 4.67 (d, J = 8.5 Hz, 1H), 4.48 (d, J = 7.9 Hz, 1H), 4.46 (d, J = 7.9 Hz, 1H), 4.41 (d, J = 7.9 Hz, 1H), 4.17 (dd, J = 3.5, 12.1 Hz, 1H), 4.13-4.12 (m, 2H), 4.08 (d, J = 2.3 Hz, 1H), 4.04 (dd, J = 2.7, 10.0 Hz, 1H), 4.00 (dd, J = 8.8 Hz, 1H), 3.95 (d, J = 11.8 Hz, 1H), 3.92-3.50 (m, 34H), 3.39 (dd, J = 8.3, 9.5 Hz, 1H), 3.30 (t, J = 6.9 Hz, 2H), 3.26 (t, J = 8.6 Hz, 1H), 2.74 (dd, J = 4.5, 12.2 Hz, 1H), 2.65 (dd, J = 4.4, 12.2 Hz, 1H), 2.04 (s, 6H), 2.01 (s, 3H), 1.70 (dd, J = 12.2, 12.2 Hz, 2H), 1.63-1.58 (m, 4H), 1.37 (brs, 4H); ^{13}C NMR (125 MHz, D_2O) δ176.0, 175.92, 175.88, 174.6, 174.3, 105.7, 103.9, 103.3, 102.9, 101.4, 100.9, 81.0, 79.5, 79.4, 76.9, 76.2, 75.73, 75.70, 75.5, 75.3 (2C), 75.0, 73.7, 73.6, 73.5, 72.7, 72.0, 71.5, 70.39, 70.37, 69.9, 69.4, 69.1, 68.9, 68.2, 63.5, 62.6, 62.0 (2C), 61.7, 61.0, 53.2, 52.7, 52.3, 52.1, 41.4, 40.8, 29.5, 28.8, 26.6, 25.5, 23.5, 23.3, 23.0; HRMS (ESI-TOF): m/z of $C_{54}H_{89}N_6O_{37}^-$[M-H]$^-$: calculated 1413.5267, found 1413.5257.

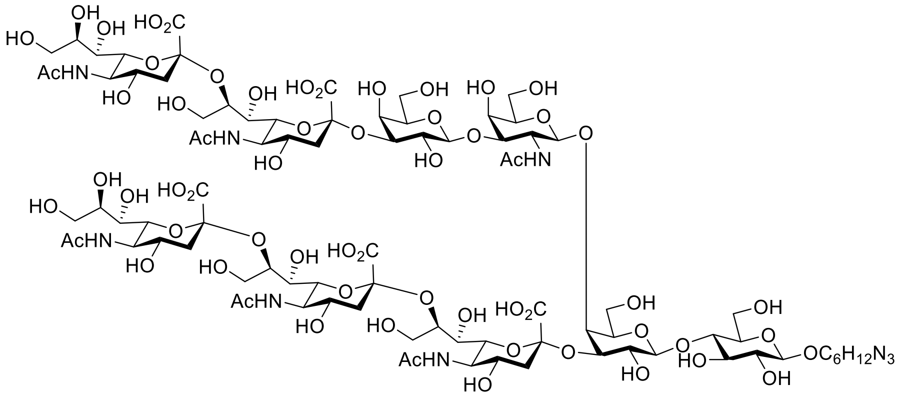

22

Compound 22 (GP1c). 17 (**GQ1c**) (1.0 equiv.) and Neu5Ac (2.0 equiv.) were used as acceptor and donor precursor respectively by following the general enzymatic synthetic procedure **SOPME-S3,8**. After being shaken for 4 h, the reaction was purified by following general purification procedure 1. Product-containing fractions were further purified by HPLC-HILIC as described general purification procedure 2 (using an elution of 60% acetonitrile in water containing 100 mM ammonium formate (pH = 3.45) to remove the impurity of hydrolyzed sugar donor. After purification, the product was lyophilized to give **22** (**GP1c**) in 20% (4.5 mg). R_f = 0.45 (*n*-PrOH/H_2O/25% NH_4OH = 7/2/1 (v/v/v)); ^{1}H NMR (850 MHz, D_2O) δ 4.74 (d, J = 8.4 Hz, 1H), 4.61 (d, J = 7.8 Hz, 1H), 4.51 (d, J = 8.1 Hz, 1H), 4.50 (d, J = 8.3 Hz,

1H), 4.19-4.06 (m, 10H), 4.03-3.99 (m, 3H), 3.96-3.81 (m, 15H), 3.80-3.59 (m, 30H), 3.56 (dd, J = 9.1, 8.5 Hz, 1H), 3.43 (t, J = 8.9 Hz, 1H), 3.34 (t, J = 6.9 Hz, 2H), 3.30 (t, J = 8.5 Hz, 1H), 2.80 (dd, J = 12.3, 4.2 Hz, 2H), 2.76 (dd, J = 12.0, 3.9 Hz, 1H), 2.72 (dd, J = 12.3, 4.1 Hz, 1H), 2.66 (dd, J = 12.2, 4.1 Hz, 1H), 2.09 (s, 3H), 2.08 (s, 3H), 2.07 (s, 6H), 2.05 (s, 6H), 1.84-1.74 (m, 5H), 1.66-1.62 (m, 4H), 1.42-1.41 (m, 4H); ^{13}C NMR (214 MHz, D_2O) δ 176.0, 175.9 (4C), 175.8, 174.1 (2C), 174.0, 173.9, 173.3, 105.3, 103.7, 103.4, 103.0, 101.82, 101.77, 101.6, 101.3, 100.9, 80.8, 79.3, 78.8, 78.7, 78.3, 76.4, 76.1, 75.8, 75.7, 75.6, 75.3 (2C), 75.1, 74.9, 74.8, 74.2, 73.8 (2C), 73.7, 72.7 (2C), 71.5, 70.6, 70.2, 69.7 (2C), 69.5, 69.4, 69.3, 69.14, 69.09, 69.0, 68.9 (2C), 68.7, 68.6, 63.6 (2C), 62.18, 62.15, 62.0, 61.9 (2C), 61.7, 61.0, 53.3, 53.2, 53.0, 52.7 (2C), 52.15, 52.10, 41.5, 41.4, 40.8, 40.6, 39.7, 29.5, 28.8, 26.6, 25.6, 23.5, 23.34, 23.26, 23.2, 23.0 (2C); HRMS (ESI-TOF): m/z of $C_{87}H_{134}N_9O_{59}^{3-}$ [M-3H-2H$_2$O]$^{3-}$: calculated 749.5926, found 749.5887.

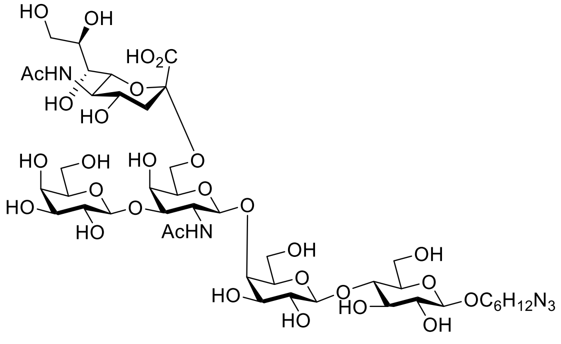

23

Compound 23 (GM1α). 9 (**GA1**) (1.0 equiv.) and Neu5Ac (1.1 equiv.) were used as acceptor and donor precursor respectively by following the general enzymatic synthetic procedure **SNRS-S6**. After being shaken for 6 h (Psp2,6ST) or 4 h (Pd2,6ST), the reaction was purified by following general purification procedure 1. After purification, the product was lyophilized to give **23** (**GM1α**) in 93% (8 mg for Psp2,6ST) or 78% (5 mg for Pd2,6ST) and **24** (**GM1α-S6**) in 4% (0.5 mg for Psp2,6ST) 11% (0.7 mg for Pd2,6ST) respectively. R_f of **23** (**GM1α**)= 0.26 (n-PrOH : H_2O : AcOH = 6 : 2 : 1); ^{1}H NMR (600 MHz, D_2O) δ 4.64 (d, J = 8.5 Hz, 1H), 4.48 (d, J = 8.1 Hz, 1H), 4.43 (d, J = 7.9 Hz, 1H), 4.43 (d, J = 7.8 Hz, 1H), 4.17 (d, J = 3.3 Hz, 1H), 4.12 (d, J = 2.8 Hz, 1H), 4.09 (dd, J = 3.1, 9.7 Hz, 1H), 4.01 (dd, J = 8.5, 10.7 Hz, 1H), 3.98-3.55 (m, 27H), 3.52 (dd, J = 7.7, 9.9 Hz, 1H), 3.39 (dd, J = 7.9, 10.0 Hz, 1H), 3.32 (t, J = 6.9 Hz, 2H), 3.28 (dd, J = 8.0, 9.1 Hz, 1H), 2.71 (dd, J = 4.7, 12.4 Hz, 1H), 2.03 (s, 3H), 2.02 (s, 3H), 1.65 (dd, J = 12.4, 12.4 Hz, 1H), 1.65-1.59 (m, 4H), 1.45-1.37 (brs, 4H); ^{13}C NMR (150 MHz, D_2O) δ

176.0, 175.9, 174.4, 105.8, 103.9, 103.5, 103.0, 101.4, 80.7, 79.5, 77.2, 76.0, 75.8, 75.4, 75.1, 73.9, 73.7, 73.54, 73.46, 73.3, 72.8, 72.0, 71.6, 71.5, 69.6, 69.4, 69.3, 69.0, 64.7, 63.6, 61.9, 61.6, 61.1, 52.8, 52.4, 52.1, 41.2, 29.5, 28.8, 26.6, 25.6, 23.4, 23.0; HRMS (ESI-TOF) : m/z of $C_{43}H_{72}N_5O_{29}^-$[M-H]$^-$: calculated 1122.4313, found 1122.4301.

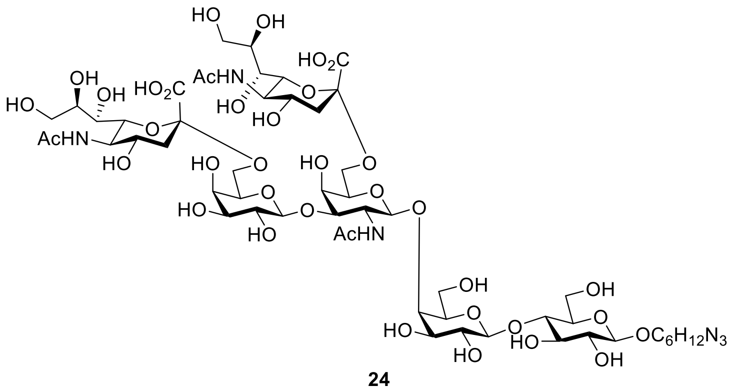

24

Compound 24 (GM1α-S6). 23 (**GM1α**) (1.0 equiv.) and Neu5Ac (1.2 equiv.) were used as acceptor and donor precursor respectively by following the general enzymatic synthetic procedure **SNRS-S6$^{\mathbf{Pd}}$**. After being shaken for 5 h, the reaction was purified by following general purification procedure 1. After purification, the product was lyophilized to give **24** (**GM1α-S6**) in 92% (5 mg). R_f = 0.26 (*n*-PrOH : H_2O : AcOH = 6 : 2 : 1). R_f = 0.14 (*n*-PrOH : H_2O : AcOH = 6 : 2 : 1); ^{1}H NMR (600 MHz, D_2O) δ 4.62 (d, J = 8.5 Hz, 1H), 4.48 (d, J = 8.1 Hz, 1H), 4.44 (d, J = 7.8 Hz, 1H), 4.44 (d, J = 7.9 Hz, 1H), 4.13 (d, J = 2.9 Hz, 1H), 4.12 (d, J = 3.1 Hz, 1H), 4.03 (dd, J = 8.5, 10.9 Hz, 1H), 4.01-3.96 (m, 2H), 3.94-3.57 (m, 31H), 3.56 (dd, J = 1.7, 9.2 Hz, 1H), 3.50 (dd, J = 7.8, 9.9 Hz, 1H), 3.39 (dd, J = 7.8, 9.8 Hz, 1H), 3.32 (t, J = 6.9 Hz, 2H), 3.29 (dd, J = 8.0, 9.1 Hz, 1H), 2.72 (dd, J = 4.7, 12.3 Hz, 1H), 2.71 (dd, J = 4.6, 12.4 Hz, 1H), 2.03 (s, 6H), 2.02 (s, 3H), 1.68 (dd, J = 12.3, 12.3 Hz, 1H), 1.67 (dd, J = 12.4, 12.4 Hz, 1H), 1.65-1.58 (m, 4H), 1.44-1.36 (brs, 4H); ^{13}C NMR (150 MHz, D_2O) δ 176.1, 175.9, 175.8, 174.4, 174.3, 105.4, 104.0, 103.5, 103.0, 79.9, 79.8, 77.2, 75.8, 75.4, 75.2, 74.2, 73.8, 73.7, 73.6, 73.4 (2C), 72.8, 72.7, 72.1, 71.6, 71.5, 69.6, 69.5, 69.4, 69.3, 69.2, 69.1, 65.1, 64.3, 63.8, 61.6, 61.2, 60.4, 53.0, 52.9, 52.5, 52.2, 41.2, 41.2, 29.9, 28.8, 26.6, 25.6, 23.5, 23.1 (2C); HRMS (ESI-TOF) : m/z of $C_{54}H_{89}N_6O_{37}^-$[M-H]$^-$: calculated 1413.5267, found 1413.5260.

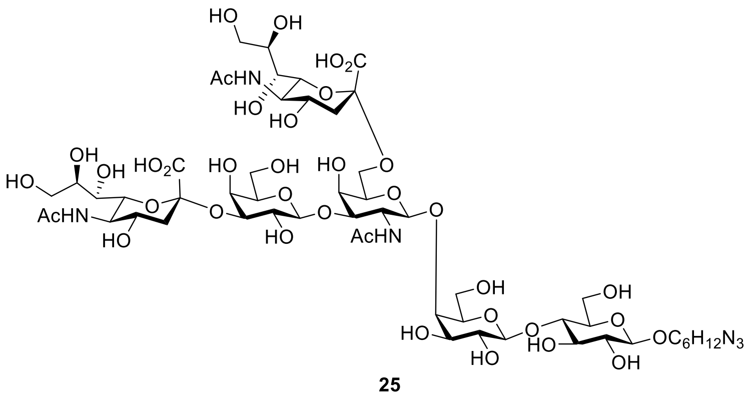

25

Compound 25 (GD1aα).

Synthesis from **23 (GM1α)**:

23 (**GM1α**) (1.0 equiv.) and Neu5Ac (1.2 equiv.) were used as acceptor and donor precursor respectively by following the general enzymatic synthetic procedure **SNRS-S3**$^{\mathbf{Cj}}$. After being shaken for 18 h, the reaction was purified by following general purification procedure 1. After purification, the product was lyophilized to give **25** (**GD1aα**) in 38% (3 mg).

Synthesis from **10 (GM1b)**:

10 (**GM1b**) (1.0 equiv.) and Neu5Ac (1.1 equiv.) were used as acceptor and donor precursor respectively by following the general enzymatic synthetic procedure **SNRS-S6**. After being shaken for 15 h (Psp2,6ST) or 18 h (Pd2,6ST), the reaction was purified by following general purification procedure 1. After purification, the product was lyophilized to give **25** (**GD1aα**) in 96% (4 mg for Psp2,6ST) or 82% (5 mg for Pd2,6ST) respectively and **26** (**GD1aα-S6**) in 14% (1.1 mg for Pd2,6ST) respectively. R_f of **25** (**GD1aα**) = 0.16 (*n*-PrOH : H_2O : AcOH = 6 : 2 : 1); ^{1}H NMR (600 MHz, D_2O) δ 4.66 (d, J = 8.6 Hz, 1H), 4.51 (d, J = 7.9 Hz, 1H), 4.49 (d, J = 8.0 Hz, 1H), 4.44 (d, J = 7.9 Hz, 1H), 4.17 (d, J = 3.0 Hz, 1H), 4.13 (d, J = 2.5 Hz, 1H), 4.08 (dd, J = 3.1, 9.7 Hz, 1H), 4.02 (dd, J = 8.6, 10.7 Hz, 1H), 4.00-3.56 (m, 33H), 3.55 (dd, J = 7.8 9.7 Hz, 1H), 3.41 (dd, J = 7.9, 9.8 Hz, 1H), 3.33 (t, J = 7.0 Hz, 2H), 3.29 (dd, J = 7.9, 9.3 Hz, 1H), 2.75 (dd, J = 4.6, 12.5 Hz, 1H), 2.71 (dd, J = 4.5, 12.4 Hz, 1H), 2.04 (s, 3H), 2.032 (s, 3H), 2.030 (s, 3H), 1.80 (dd, J = 12.4, 12.4 Hz, 1H), 1.67 (dd, J = 12.5, 12.5 Hz, 1H), 1.66-1.59 (m, 4H), 1.44-1.36 (brs, 4H); ^{13}C NMR (150 MHz, D_2O) δ 175.95, 175.90, 175.8, 174.9, 174.4, 105.6, 104.0, 103.5, 103.0, 101.4, 100.7, 80.8, 79.6, 77.2, 76.5, 75.8, 75.7, 75.4, 75.1, 73.9, 73.8, 73.7, 73.5, 73.3, 72.8 (2C), 72.0, 71.5, 70.0, 69.40, 69.36, 69.3, 69.1, 68.8, 68.4, 64.7 63.6, 63.5, 61.9, 61.6, 61.1, 52.8, 52.6, 52.2, 52.1, 41.2, 40.7, 29.6, 28.8, 26.6, 25.6, 23.4, 23.0 (2C); HRMS (ESI-TOF) : m/z of $C_{54}H_{89}N_6O_{37}^-$[M-H]$^-$: calculated 1413.5267, found 1413.5248.

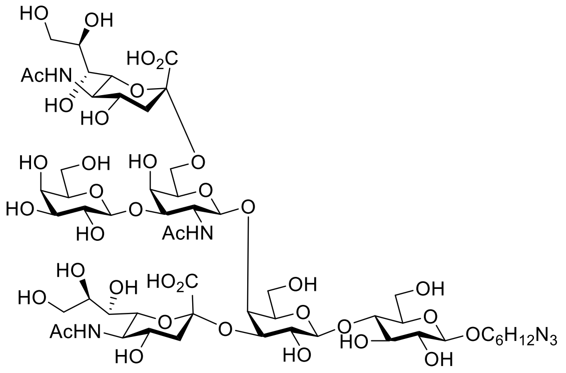

25'

Compound 25' (GD1aα'). 11 (**GM1a**) (197.6 mg, 0.1758 mmol, 1.0 equiv.) and Neu5Ac (1.33 equiv.) were used as acceptor and donor precursor respectively by following the general enzymatic synthetic procedure **SNRS-S6^{Psp}**. After being shaken for 23 h, the reaction was purified by following general purification procedure 1. After purification, the product was lyophilized to give a mixture of **GD1aα'** and **GD1aα''** (43%) and **GD1aα'-S6** (2%). The mixture was further purified by C18 reverse-phase chromatography (Sep-Pak C18 35 c.c. Vac Cartridge, 10 g sorbent, 55-105 μM, Waters) for many times and eluted by a gradient from 100% H_2O to 10% methanol in H_2O. Fractions containing **25'** (**GD1aα'**) were collected, concentrated, and further purified by size-exclusion chromatography (Bio Gel P-2 gel packed in column, 1.5 cm x 50 cm, Bio-Rad) with distilled water as eluent and then lyophilized to give **25'** (**GD1aα'**) in 2% as isolated yield (5 mg). R_f = 0.14 (*n*-PrOH : H_2O : AcOH = 6 : 2 : 1); ^{1}H NMR (600 MHz, D_2O) δ 4.73 (d, J = 8.7 Hz, 1H), 4.53 (d, J = 8.0 Hz, 1H), 4.52 (d, J = 7.9 Hz, 1H), 4.48 (d, J = 8.0 Hz, 1H), 4.18 (d, J = 3.2 Hz, 1H), 4.15 (dd, J = 3.0, 9.7 Hz, 1H), 4.12 (d, J = 3.1 Hz, 1H), 4.04 (dd, J = 8.5, 10.7 Hz, 1H), 3.99-3.74 (m, 17H), 3.73-3.53 (m, 15H), 3.51 (dd, J = 7.8, 9.9 Hz, 1H), 3.49 (dd, J = 2.1, 10.4 Hz, 1H), 3.34 (dd, J = 8.0, 9.7 Hz, 1H), 3.32 (t, J = 6.9 Hz, 2H), 3.28 (dd, J = 8.0, 9.1 Hz, 1H), 2.70 (dd, J = 4.5, 12.2 Hz, 1H), 2.64 (dd, J = 4.7, 12.6 Hz, 1H), 2.03 (s, 3H), 2.02 (s, 3H), 1.99 (s, 3H), 1.90 (dd, J = 12.2, 12.2 Hz, 1H), 1.66-1.59 (m, 5H), 1.43-1.36 (brs, 4H); ^{13}C NMR (150 MHz, D_2O) δ 176.0, 175.9, 175.7, 175.1, 174.3, 105.7, 103.6, 103.5, 103.0, 102.8, 101.3, 81.3, 79.7, 78.5, 75.9, 75.8, 75.5, 75.3, 74.8, 74.2, 73.9, 73.8, 73.6, 73.4, 74.3, 72.8, 71.7, 71.5, 71.0, 69.8, 69.64, 69.61, 69.4, 69.0, 68.7, 64.6, 63.7, 63.5, 61.9, 61.6, 61.2, 52.9, 52.6, 52.2, 52.0, 41.2, 37.7, 29.6, 28.8, 26.6, 25.6, 23.6, 23.1 (2C); HRMS (ESI-TOF) : m/z of $C_{54}H_{89}N_6O_{37}^-$[M-H]$^-$: calculated 1413.5267, found 1413.5255.

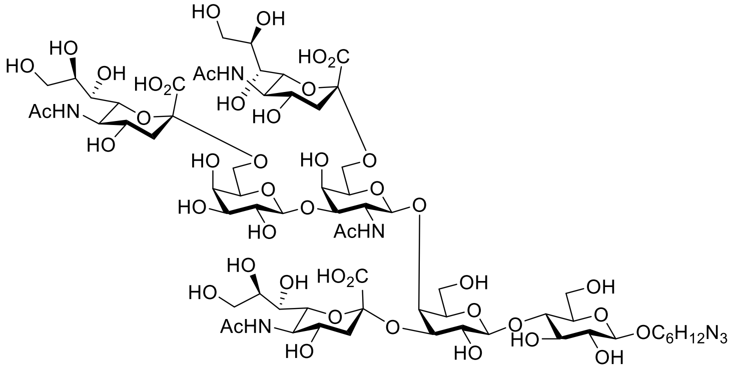

GD1aα'-S6

GD1aα'-S6. R_f = 0.1 (*n*-PrOH/ H_2O/ AcOH = 6/ 2/ 1 (v/ v/ v)); ^1H NMR (700 MHz, D_2O) δ 4.72 (d, J = 8.5 Hz, 1H), 4.54 (d, J = 7.8 Hz, 1H), 4.53 (d, J = 7.4 Hz, 1H), 4.49 (d, J = 8.0 Hz, 1H), 4.16-4.14 (m, 3H), 4.06-3.98 (m, 3H), 3.95-3.49 (m, 40H), 3.36 (t, J = 8.3 Hz, 1H), 3.33 (t, J = 6.9 Hz, 2H), 3.30 (t, J = 8.5 Hz, 1H), 2.74 (dd, J = 4.6, 12.2 Hz, 1H), 2.72 (dd, J = 4.3, 11.8 Hz, 1H), 2.66 (dd, J = 4.6, 12.5 Hz, 1H), 2.04 (s, 6H), 2.03 (s, 3H), 2.00 (s, 3H), 1.96 (t, J = 12.2 Hz, 1H), 1.69 (t, J = 12.2 Hz, 1H), 1.67-1.61 (m, 5H), 1.41-1.40 (m, 4H); ^{13}C NMR (175 MHz, D_2O) δ 174.9, 174.8, 174.7, 174.6, 174.0, 173.4, 173.3, 104.5, 102.4, 102.4, 101.9, 101.7, 100.4, 100.2, 80.0, 78.4, 77.1, 74.6, 74.3, 74.1, 73.5, 73.1, 72.88, 72.86, 72.7, 72.5, 72.3, 72.2, 72.0, 71.54, 71.52, 70.5, 70.4, 69.9, 68.7, 68.5, 68.4, 68.3, 68.1, 68.0, 67.8, 67.5, 64.1, 62.8, 62.6, 62.5, 62.3, 60.3, 60.0, 51.9, 51.7, 51.5, 51.02, 50.98, 40.14, 40.08, 36.6, 28.5, 27.8, 25.5, 24.5, 22.5, 22.0 (2C), 21.9; HRMS (ESI-TOF) : m/z of $C_{65}H_{106}N_7O_{45}^-$[M-H]$^-$: calculated 1704.6221, found 1704.6184.

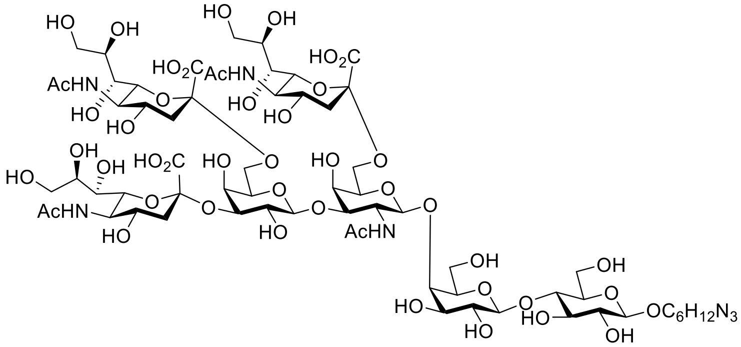

26

Compound 26 (GD1aα-S6). 25 (**GD1aα**) (1.0 equiv.) and Neu5Ac (1.2 equiv.) were used as acceptor and donor precursor respectively by following the general enzymatic synthetic procedure **SNRS-S6^{Pd}**. After being shaken for 13 h, the reaction was purified by following general purification procedure 1. After

purification, the product was lyophilized to give **26** (**GD1aα-S6**) in 77% (4 mg). R_f = 0.08 (*n*-PrOH : H_2O : AcOH = 6 : 2 : 1); ^{1}H NMR (600 MHz, D_2O) δ 4.62 (d, *J* = 8.4 Hz, 1H), 4.51 (d, *J* = 7.7 Hz, 1H), 4.48 (d, *J* = 8.1 Hz, 1H), 4.43 (d, *J* = 7.9 Hz, 1H), 4.13 (d, *J* = 2.5 Hz, 1H), 4.11 (d, *J* = 2.7 Hz, 1H), 4.07 (dd, *J* = 3.1, 10.0 Hz, 1H), 4.04 (dd, *J* = 8.7, 11.1 Hz, 1H), 4.01-3.54 (m, 40H), 3.52 (dd, *J* = 8.3, 9.6 Hz, 1H), 3.40 (dd, *J* = 8.1, 9.6 Hz, 1H), 3.32 (t, *J* = 6.9 Hz, 2H), 3.28 (dd, *J* = 8.1, 9.2 Hz, 1H), 2.74 (dd, *J* = 4.5, 12.2 Hz, 1H), 2.72 (dd, *J* = 4.7, 12.2 Hz, 1H), 2.71 (dd, *J* = 4.6, 12.1 Hz, 1H), 2.035 (s, 3H), 2.030 (s, 3H), 2.02 (s, 6H), 1.78 (dd, *J* = 12.1, 12.1 Hz, 1H), 1.68 (dd, *J* = 12.2, 12.2 Hz, 1H), 1.67 (dd, *J* = 12.2, 12.2 Hz, 1H), 1.65-1.58 (m, 4H), 1.42-1.37 (brs, 4H); ^{13}C NMR (150 MHz, D_2O) δ 176.0, 175.91, 175.85, 175.81, 174.79, 174.4 (2C), 105.1, 104.0, 103.6, 103.0, 101.6, 101.4, 100.8, 79.9, 79.6, 77.2, 76.4, 75.8, 75.4, 75.1, 74.1, 74.0, 73.8 (2C), 73.6, 73.5, 73.3, 72.8, 72.71, 72.65, 72.0, 71.5, 69.9, 69.5 (2C), 69.4, 69.3, 69.2, 69.1, 68.9, 68.3, 65.2, 64.2, 63.7 (2C), 63.5, 61.6, 61.1, 53.0, 52.8, 52.6, 52.3, 52.1, 41.2, 41.1, 40.6, 29.6, 28.9, 26.6, 25.6, 23.5, 23.0 (3C); HRMS (ESI-TOF) : m/z of $C_{65}H_{106}N_7O_{45}^-$[M-H]$^-$: calculated 1704.6221, found 1704.6196.

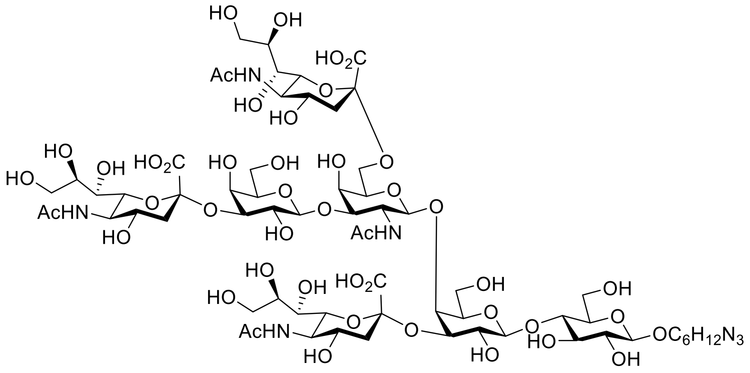

27

Compound 27 (GT1aα). 15 (**GD1a**) (1.0 equiv.) and Neu5Ac (1.2 equiv.) were used as acceptor and donor precursor respectively by following the general enzymatic synthetic procedure **SNRS-S6^{Psp}**. After being shaken for 28 h, the reaction was purified by following general purification procedure 2. After purification, the product was lyophilized to give **27** (**GT1aα**) in 7% (3 mg). R_f = 0.09 (*n*-PrOH : H_2O : AcOH = 6 : 2 : 1); ^{1}H NMR (600 MHz, D_2O) δ 4.72 (d, *J* = 8.5 Hz, 1H), 4.60 (d, *J* = 7.9 Hz, 1H), 4.53 (d, *J* = 7.9 Hz, 1H), 4.48 (d, *J* = 8.0 Hz, 1H), 4.18 (d, *J* = 3.0 Hz, 1H), 4.15 (dd, *J* = 2.9, 9.6 Hz, 1H), 4.12 (d, *J* = 2.8 Hz, 1H), 4.09 (dd, *J* = 3.1, 9.9 Hz, 1H), 4.04 (dd, *J* = 8.5, 10.9 Hz, 1H), 4.00-3.54 (m, 38H), 3.53 (dd, *J* = 8.0, 10.0 Hz, 1H), 3.50 (dd, *J* = 2.0, 10.3 Hz, 1H), 3.36 (dd, *J* = 8.0, 9.6 Hz, 1H), 3.33 (t, *J* = 6.9 Hz, 2H), 3.29 (dd, *J* = 8.6, 8.6 Hz, 1H), 2.75 (dd, *J* = 4.6, 12.4 Hz, 1H), 2.70 (dd, *J* = 4.7, 12.5 Hz, 1H),

2.66 (dd, J = 4.6, 12.5 Hz, 1H), 2.04 (s, 3H), 2.03 (s, 3H), 2.02 (s, 3H), 2.00 (s, 3H), 1.94 (dd, J = 12.4, 12.4 Hz, 1H), 1.80 (dd, J = 12.5, 12.5 Hz, 1H), 1.68-1.57 (m, 5H), 1.44-1.36 (brs, 4H); ^{13}C NMR (150 MHz, D_2O) δ 175.9 (2C), 175.8, 175.6, 175.04, 175.01, 174.4, 105.5, 103.6, 103.5, 103.0, 102.6, 101.2, 100.7, 81.4, 79.5, 78.2, 76.4, 75.7, 75.6, 75.4, 75.2, 74.7, 74.1, 73.8, 73.7 (2C), 73.4, 73.2, 72.7 (2C), 71.5, 71.0, 70.1, 69.8, 69.53, 69.48, 69.42, 69.1, 68.8, 68.6, 68.4, 64.7, 63.6, 63.5 (2C), 61.9, 61.5, 61.1, 52.8, 52.63, 52.56, 52.1, 51.9, 41.2, 40.6, 37.9, 29.5, 28.8, 26.6, 25.6, 23.6, 23.0 (3C); HRMS (ESI-TOF) : m/z of $C_{65}H_{107}N_7O_{45}^-$[M-H]$^-$: calculated 1704.6221, found 1704.6247.

Reference

1. Chien, W. T.; Liang, C. F.; Yu, C. C.; Lin, C. H.; Li, S. P.; Primadona, I.; Chen, Y. J.; Mong, K. K.; Lin, C. C., Sequential one-pot enzymatic synthesis of oligo-N-acetyllactosamine and its multi-sialylated extensions. *Chem Commun (Camb)* **2014,** *50* (43), 5786-9.
2. Guan, W.; Cai, L.; Fang, J.; Wu, B.; George Wang, P., Enzymatic synthesis of UDP-GlcNAc/UDP-GalNAc analogs using N-acetylglucosamine 1-phosphate uridyltransferase (GlmU). *Chem Commun (Camb)* **2009,** (45), 6976-8.
3. Guan, W.; Cai, L.; Wang, P. G., Highly efficient synthesis of UDP-GalNAc/GlcNAc analogues with promiscuous recombinant human UDP-GalNAc pyrophosphorylase AGX1. *Chemistry* **2010,** *16* (45), 13343-5.
4. Blixt, O.; Vasiliu, D.; Allin, K.; Jacobsen, N.; Warnock, D.; Razi, N.; Paulson, J. C.; Bernatchez, S.; Gilbert, M.; Wakarchuk, W., Chemoenzymatic synthesis of 2-azidoethyl-ganglio-oligosaccharides GD3, GT3, GM2, GD2, GT2, GM1, and GD1a. *Carbohydr Res* **2005,** *340* (12), 1963-72.
5. Li, S.-P.; Hsiao, W.-C.; Yu, C.-C.; Chien, W.-T.; Lin, H.-J.; Huang, L.-D.; Lin, C.-H.; Wu, W.-L.; Wu, S.-H.; Lin, C.-C., Characterization of Meiothermus taiwanensis Galactokinase and its Use in the One-Pot Enzymatic Synthesis of Uridine Diphosphate-Galactose and the Chemoenzymatic Synthesis of the Carbohydrate Antigen Stage Specific Embryonic Antigen-3. *Adv Synth Catal* **2014,** *356* (14-15), 3199-3213.
6. Litterer, L. A.; Schnurr, J. A.; Plaisance, K. L.; Storey, K. K.; Gronwald, J. W.; Somers, D. A., Characterization and expression of Arabidopsis UDP-sugar pyrophosphorylase. *Plant Physiol Biochem* **2006,** *44* (4), 171-80.
7. Chiang, P. Y.; Adak, A. K.; Liang, W. L.; Tsai, C. Y.; Tseng, H. K.; Cheng, J. Y.; Hwu, J. R.; Yu, A. L.; Hung, J. T.; Lin, C. C., Chemoenzymatic Synthesis of Globo-series Glycosphingolipids and Evaluation of Their Immunosuppressive Activities. *Chem-Asian J* **2022,** *17* (16).
8. Tsai, T.-I.; Lee, H.-Y.; Chang, S.-H.; Wang, C.-H.; Tu, Y.-C.; Lin, Y.-C.; Hwang, D.-R.; Wu, C.-Y.; Wong, C.-H., Effective Sugar Nucleotide Regeneration for the Large-Scale Enzymatic Synthesis of Globo H and SSEA4. *Journal of the American Chemical Society* **2013,** *135* (39), 14831-14839.
9. Fang, J.-L.; Tsai, T.-W.; Liang, C.-Y.; Li, J.-Y.; Yu, C.-C., Enzymatic Synthesis of Human Milk Fucosides α1,2-Fucosyl para-Lacto-N-Hexaose and its Isomeric Derivatives. *Adv Synth Catal* **2018,** *360* (17), 3213-3219.
10. Yu, C. C.; Lin, P. C.; Lin, C. C., Site-specific immobilization of CMP-sialic acid synthetase on magnetic nanoparticles and its use in the synthesis of CMP-sialic acid. *Chem Commun (Camb)* **2008,** (11), 1308-10.
11. Yu, C. C.; Kuo, Y. Y.; Liang, C. F.; Chien, W. T.; Wu, H. T.; Chang, T. C.; Jan, F. D.; Lin, C. C., Site-specific immobilization of enzymes on magnetic nanoparticles and their use in organic synthesis. *Bioconjug Chem* **2012,** *23* (4), 714-24.

12. Chiu, C. P.; Lairson, L. L.; Gilbert, M.; Wakarchuk, W. W.; Withers, S. G.; Strynadka, N. C., Structural analysis of the alpha-2,3-sialyltransferase Cst-I from Campylobacter jejuni in apo and substrate-analogue bound forms. *Biochemistry* **2007,** *46* (24), 7196-204.
13. Tseng, H. K.; Su, Y. Y.; Chang, T. W.; Liu, H. C.; Li, P. J.; Chiang, P. Y.; Lin, C. C., Acceptor-mediated regioselective enzyme catalyzed sialylation: chemoenzymatic synthesis of GAA-7 ganglioside glycan. *Chem Commun (Camb)* **2021,** *57* (28), 3468-3471.
14. Li, P. J.; Huang, S. Y.; Chiang, P. Y.; Fan, C. Y.; Guo, L. J.; Wu, D. Y.; Angata, T.; Lin, C. C., Chemoenzymatic Synthesis of DSGb5 and Sialylated Globo-series Glycans. *Angew Chem Int Ed Engl* **2019,** *58* (33), 11273-11278.
15. Tasnima, N.; Yu, H.; Li, Y.; Santra, A.; Chen, X., Chemoenzymatic synthesis of para-nitrophenol (pNP)-tagged alpha2-8-sialosides and high-throughput substrate specificity studies of alpha2-8-sialidases. *Org Biomol Chem* **2016,** *15* (1), 160-167.
16. Wu, H. R.; Anwar, M. T.; Fan, C. Y.; Low, P. Y.; Angata, T.; Lin, C. C., Expedient assembly of Oligo-LacNAcs by a sugar nucleotide regeneration system: Finding the role of tandem LacNAc and sialic acid position towards siglec binding. *Eur J Med Chem* **2019,** *180*, 627-636.

NMR Spectra

Current Data Parameters
NAME SYC-GM3-H
EXPNO 1
PROCNO 1

F2 - Processing parameters
SI 16384
SF 499.7849627 MHz
WDW EM
SSB 0
LB 0.30 Hz
GB 0
PC 1.00

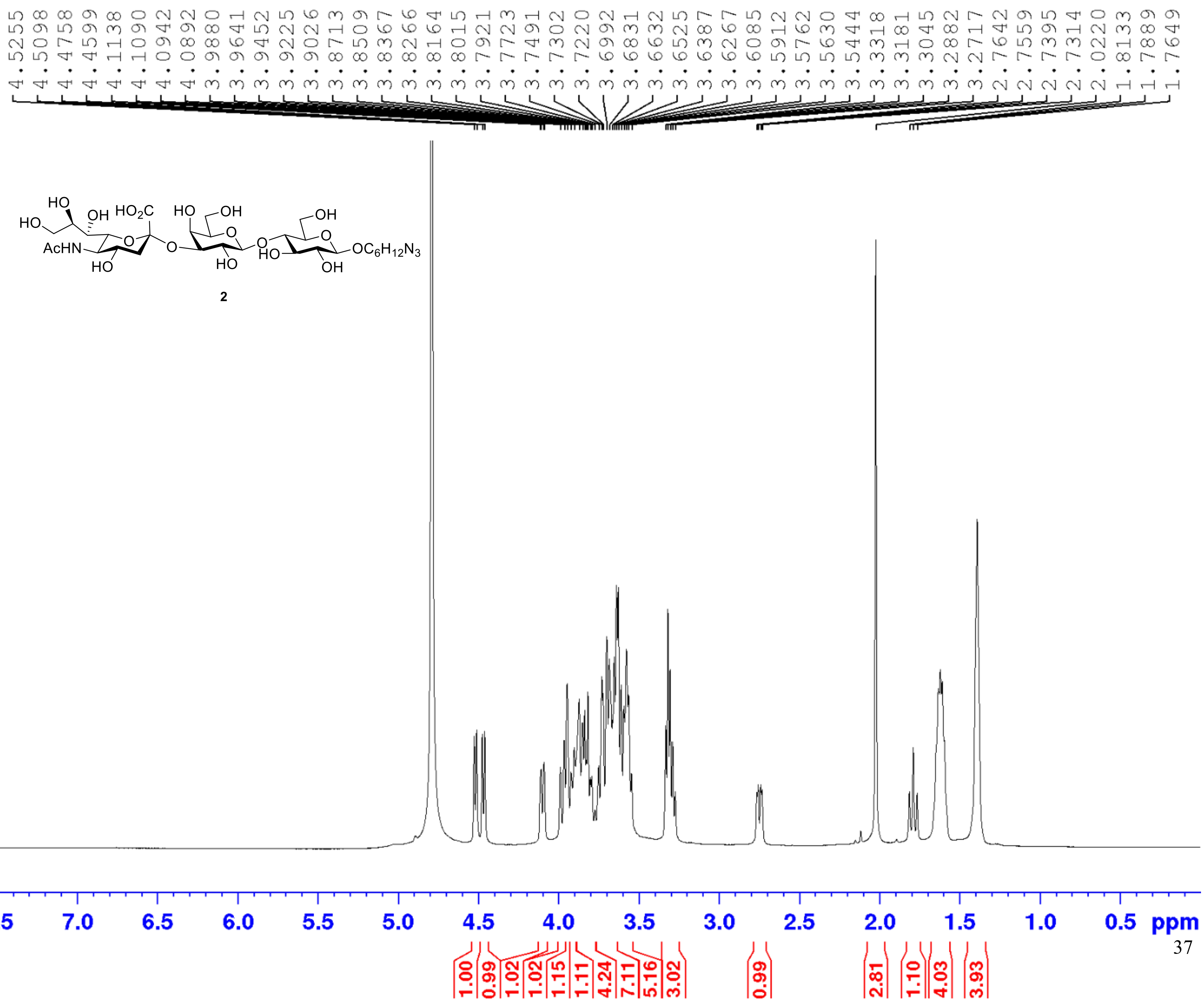

^{13}C NMR spectrum of **2** (**GM3**) (125 MHz D_2O)

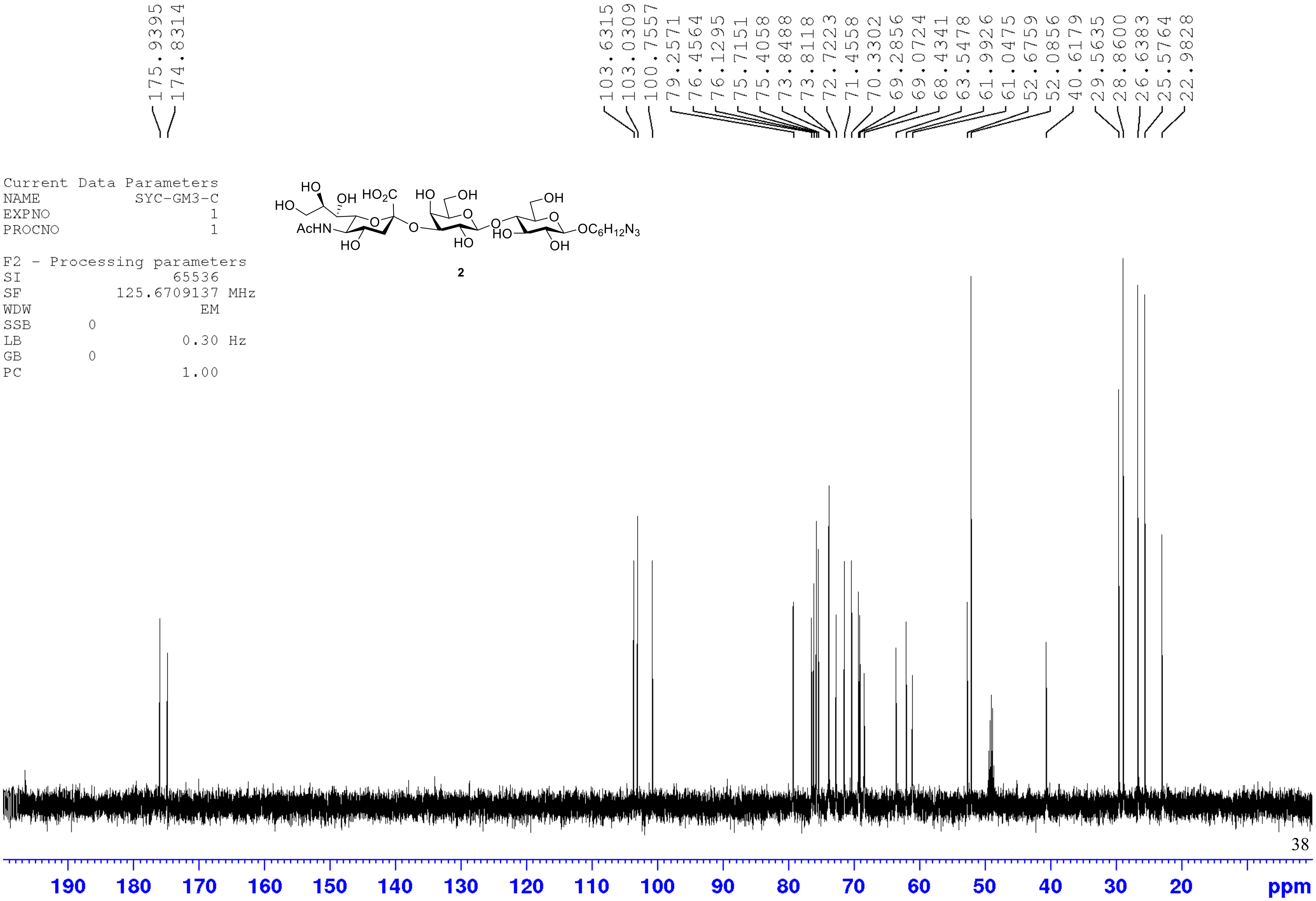

^{13}C NMR spectrum of **2** (**GM3**) (125 MHz D_2O)

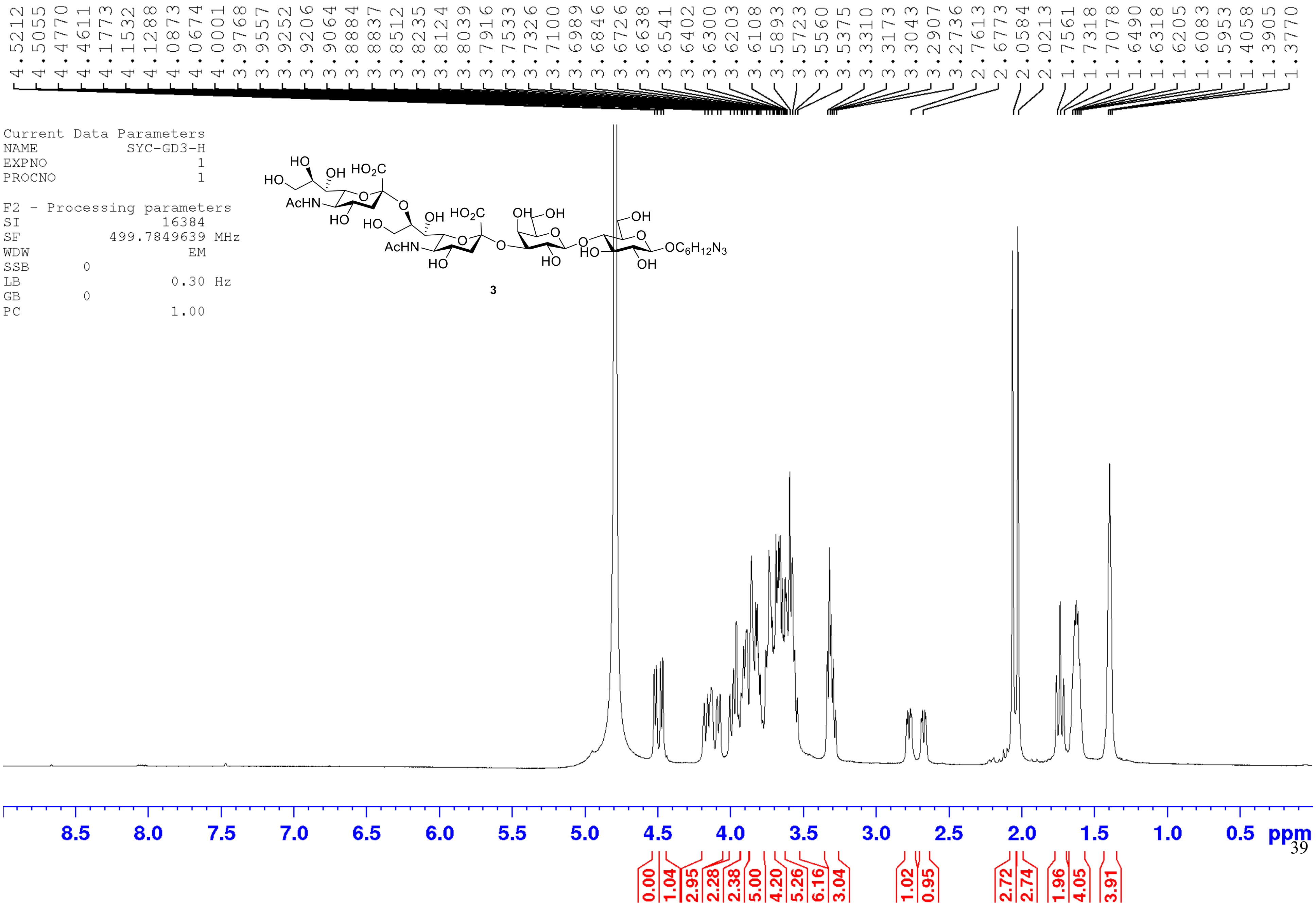

^{1}H NMR spectrum of **3** (**GD3**) (500 MHz D_2O)

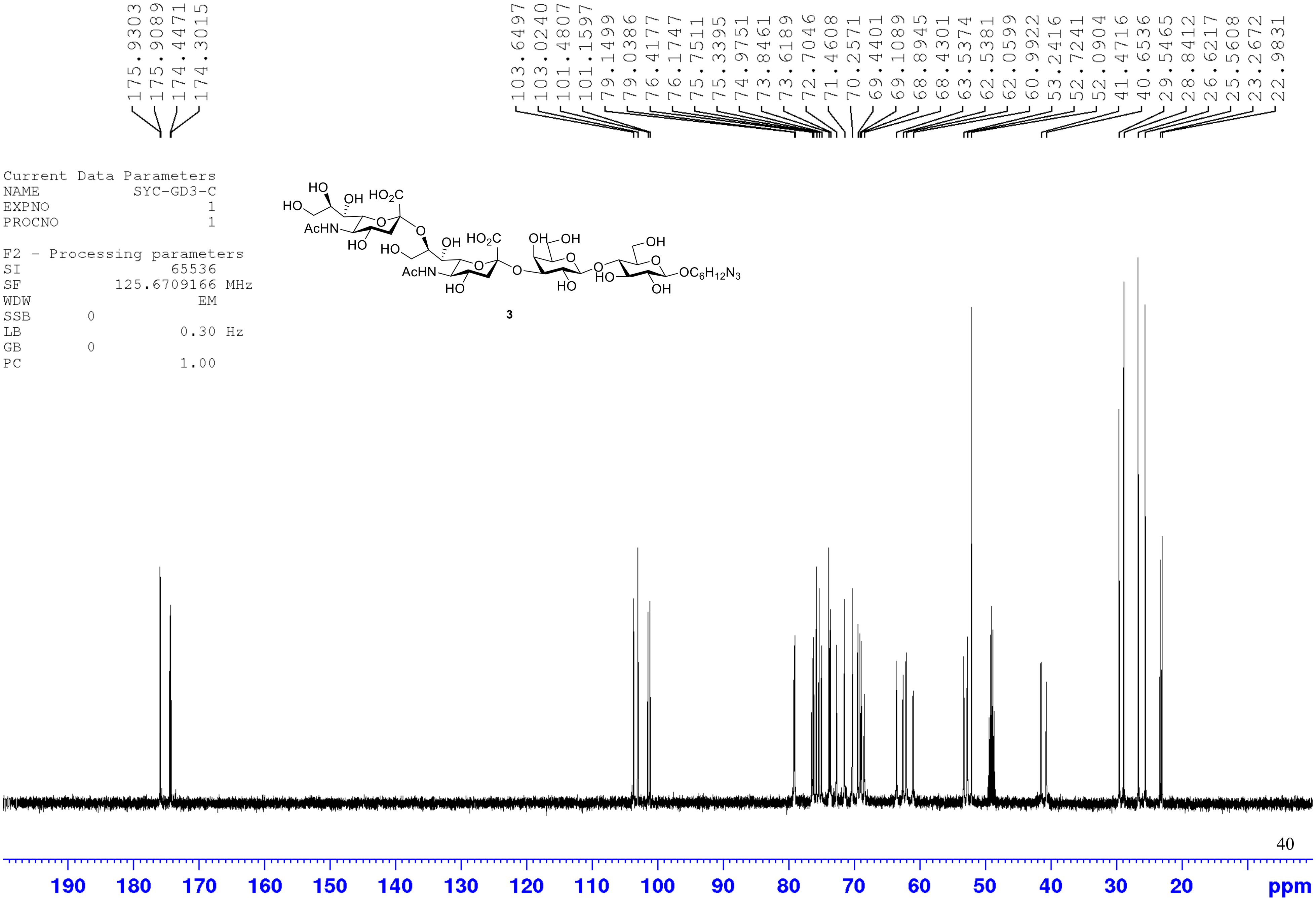

3

^{13}C NMR spectrum of **3** (**GD3**) (125 MHz D_2O)

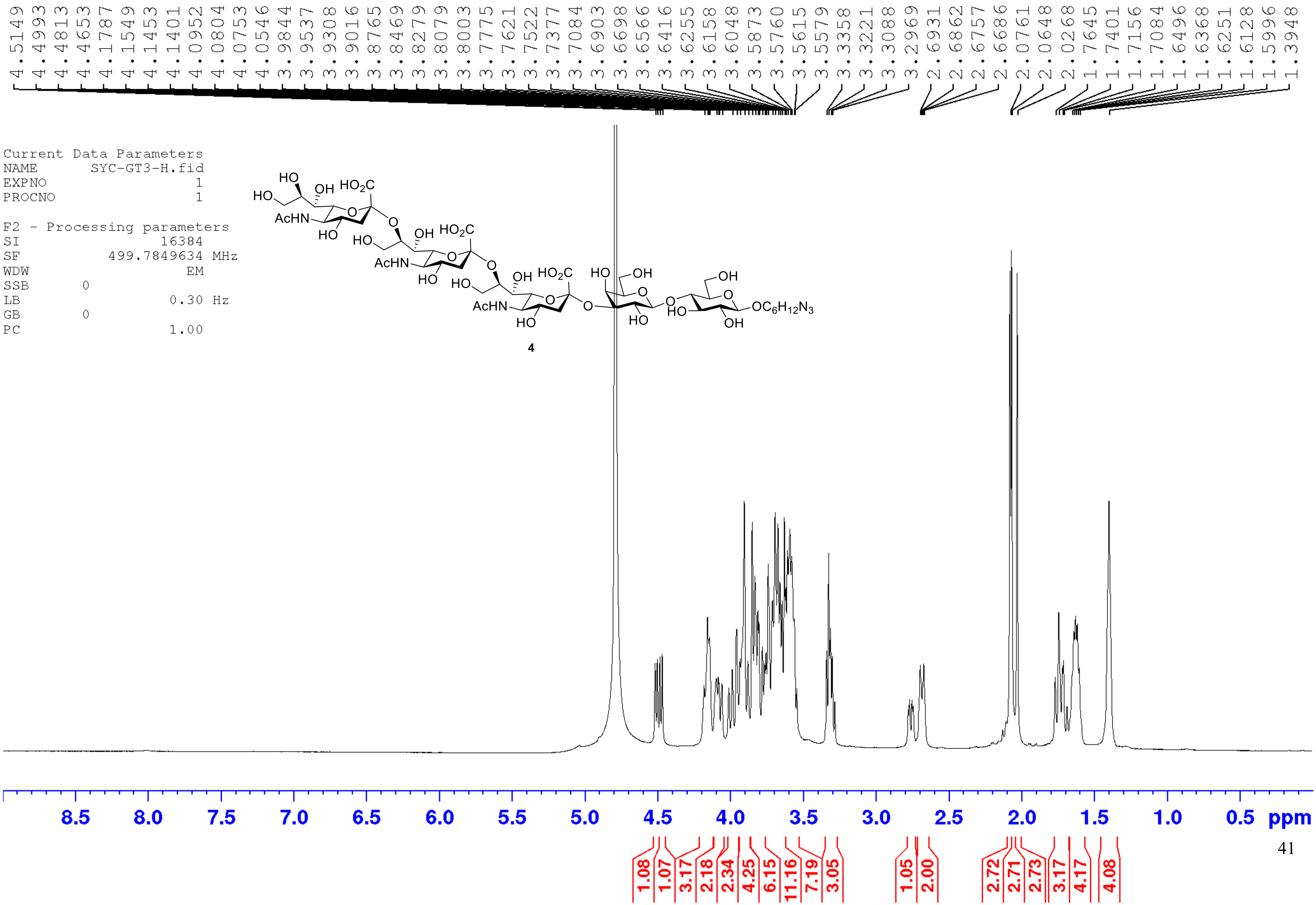

^{1}H NMR spectrum of **4** (**GT3**) (500 MHz D_2O)

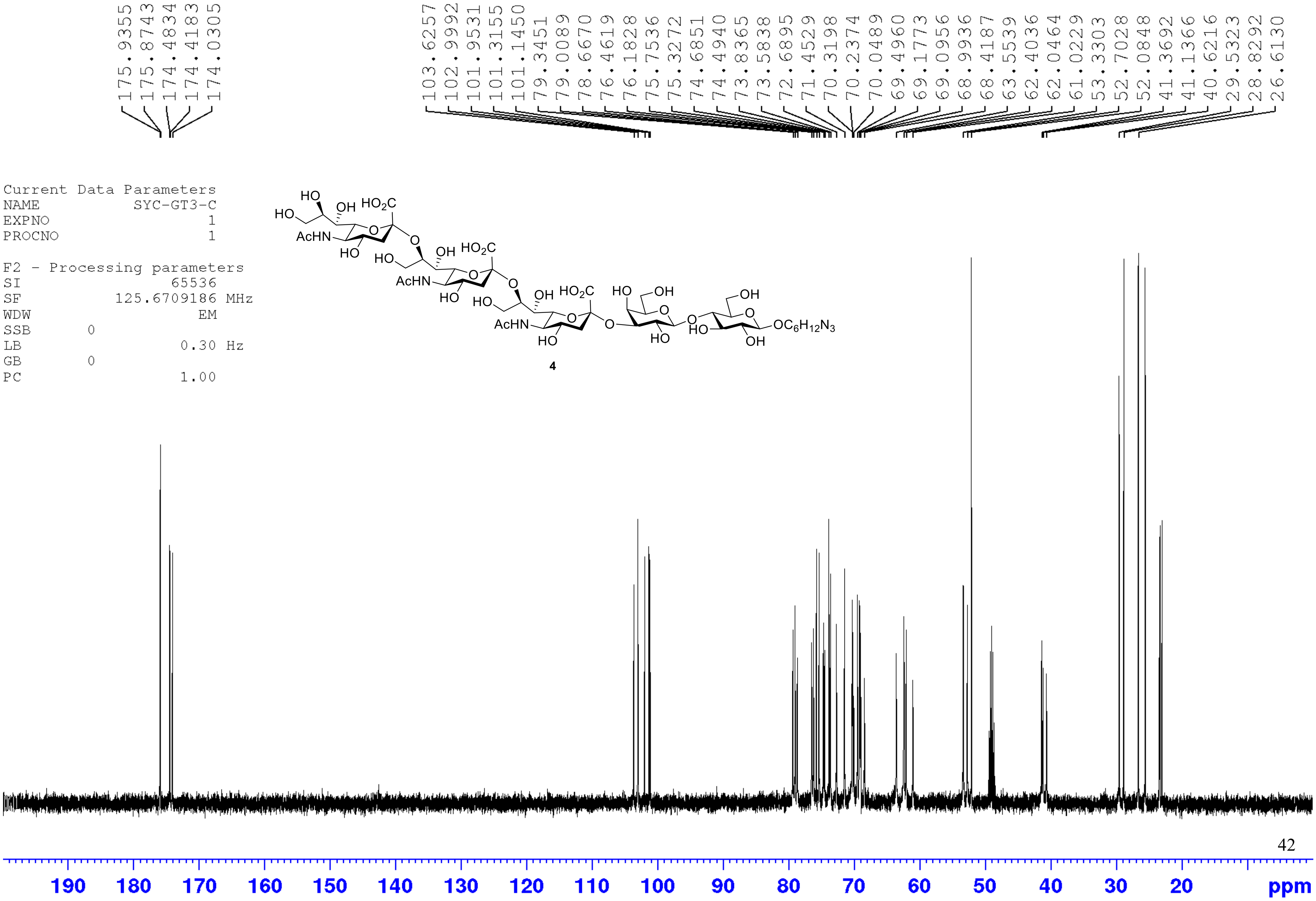

^{13}C NMR spectrum of **4** (**GT3**) (125 MHz D_2O)

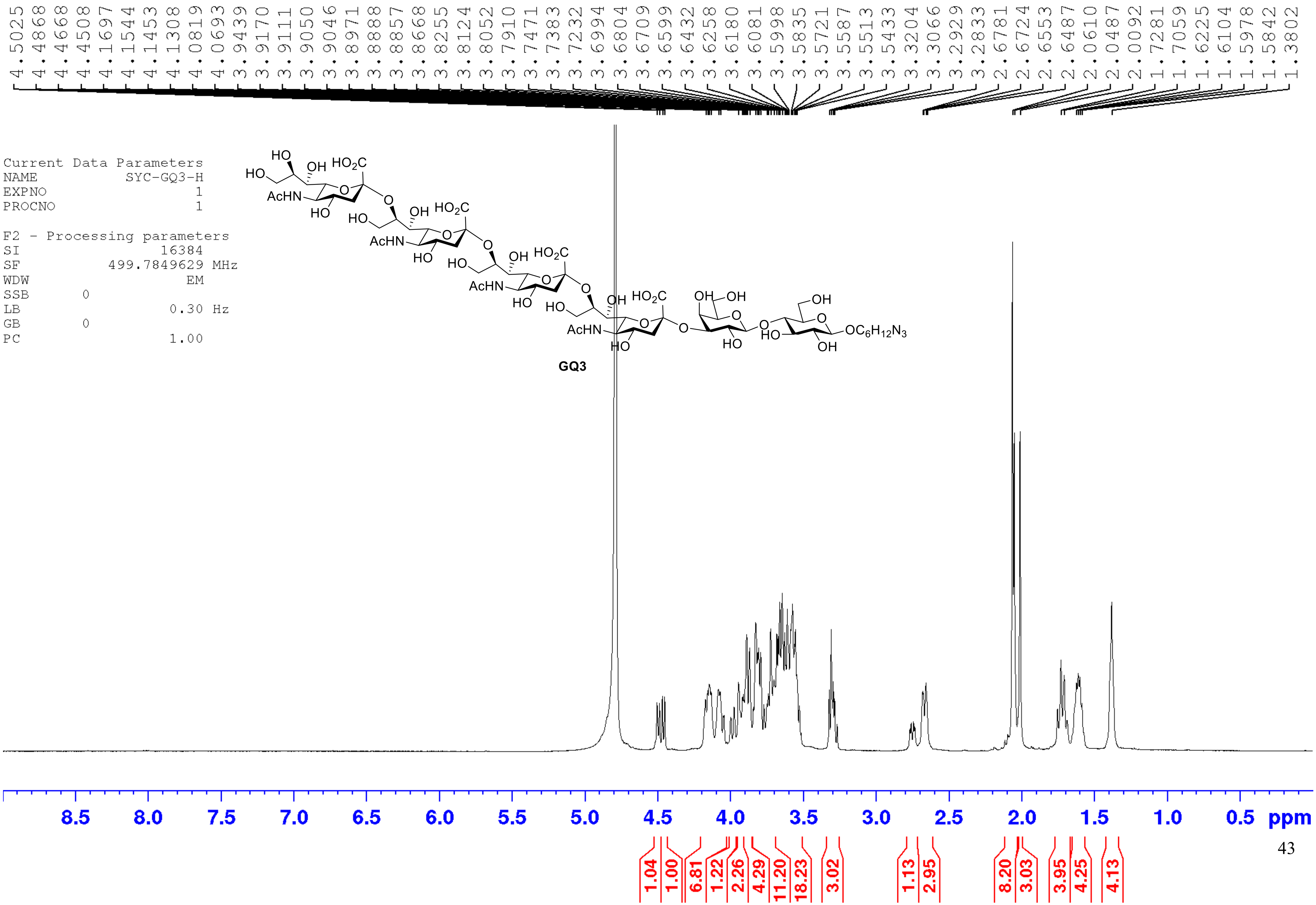

^{1}H NMR spectrum of (**GQ3**) (500 MHz D_2O)

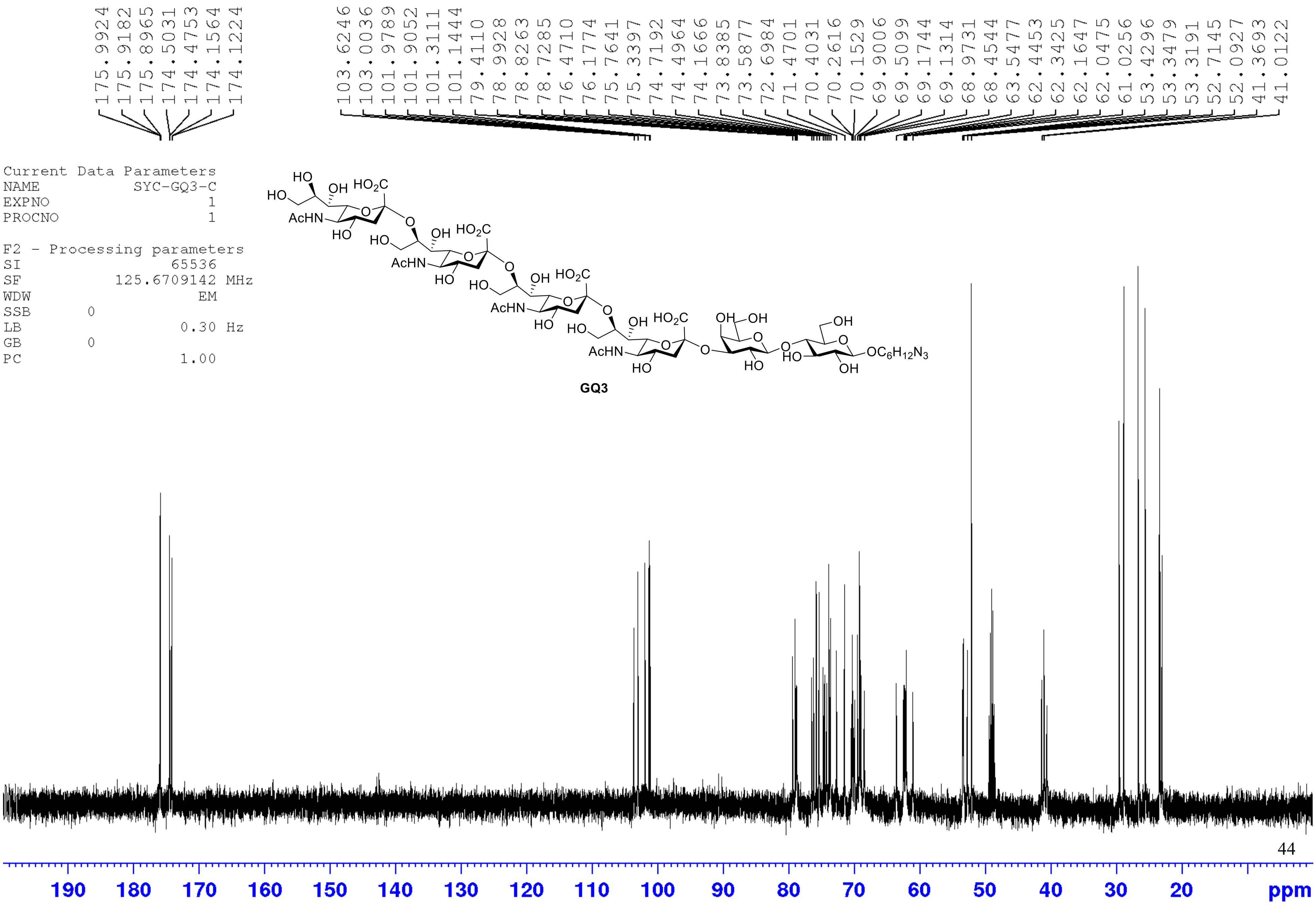

Current Data Parameters
NAME SYC-GQ3-C
EXPNO 1
PROCNO 1

F2 - Processing parameters
SI 65536
SF 125.6709142 MHz
WDW EM
SSB 0
LB 0.30 Hz
GB 0
PC 1.00

GQ3

^{1}H NMR spectrum (**GQ3**) (125 MHz D_2O)

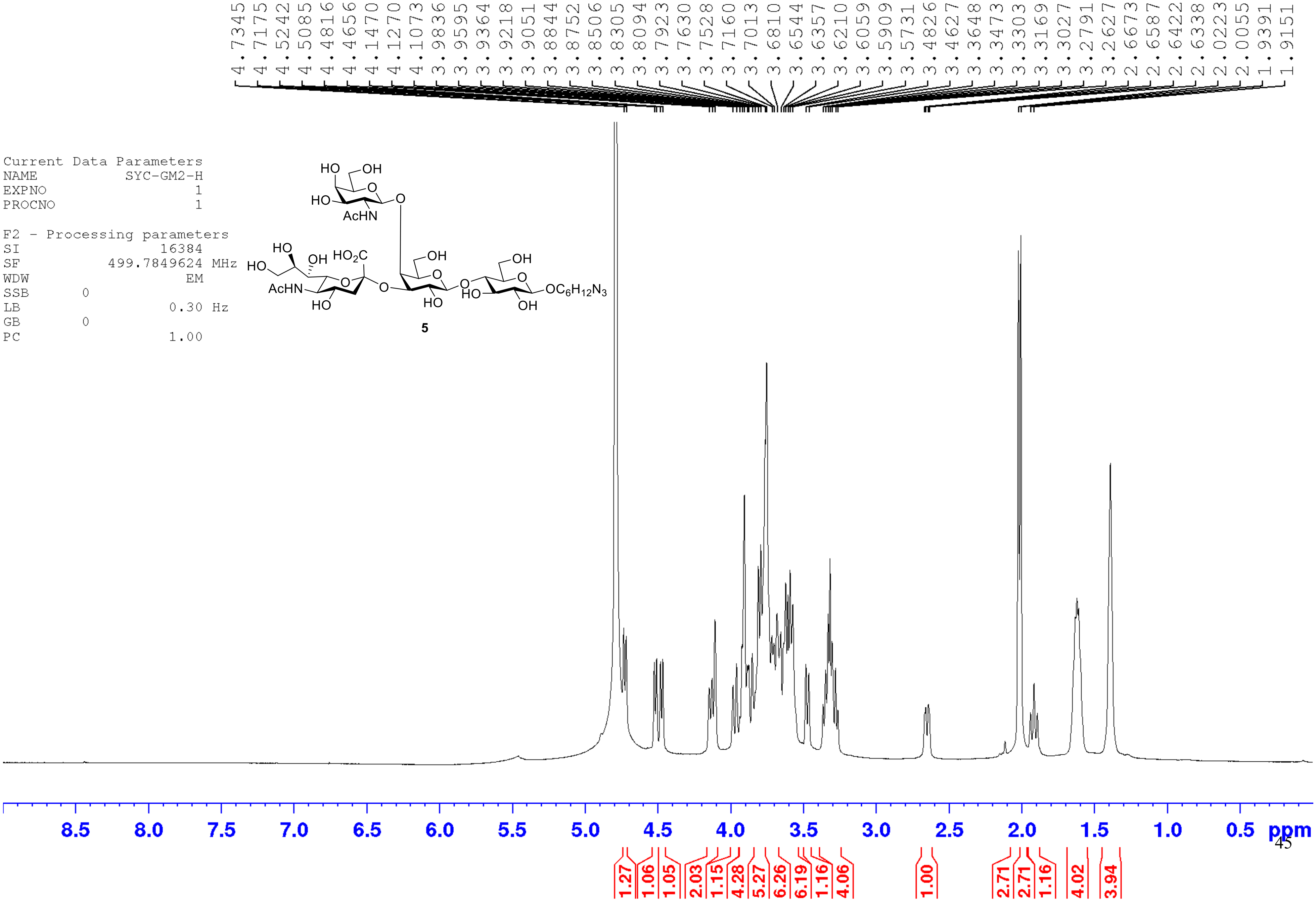

^{1}H NMR spectrum of **5** (**GM2**) (500 MHz D_2O)

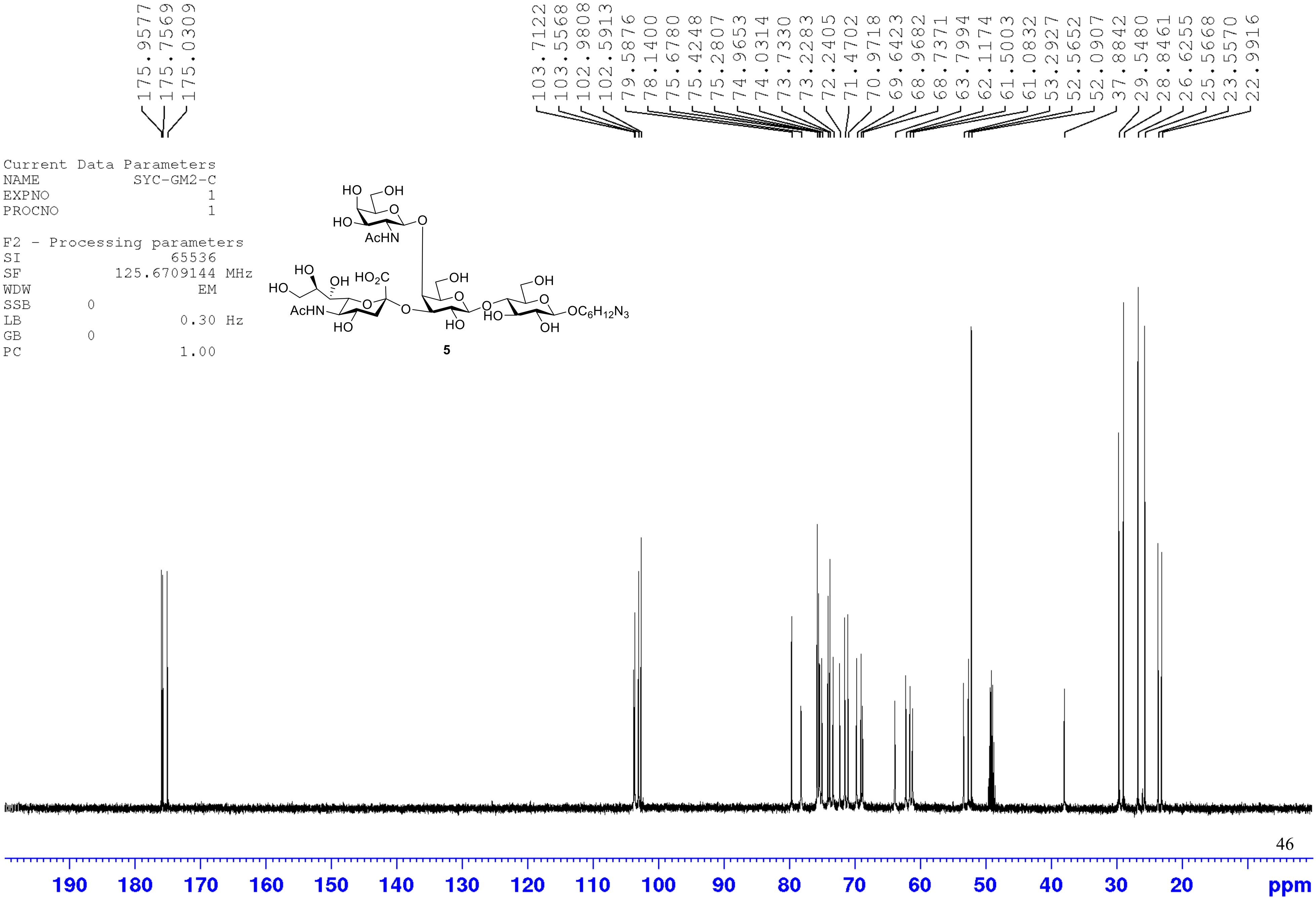

^{13}C NMR spectrum of **5** (**GM2**) (125 MHz D_2O)

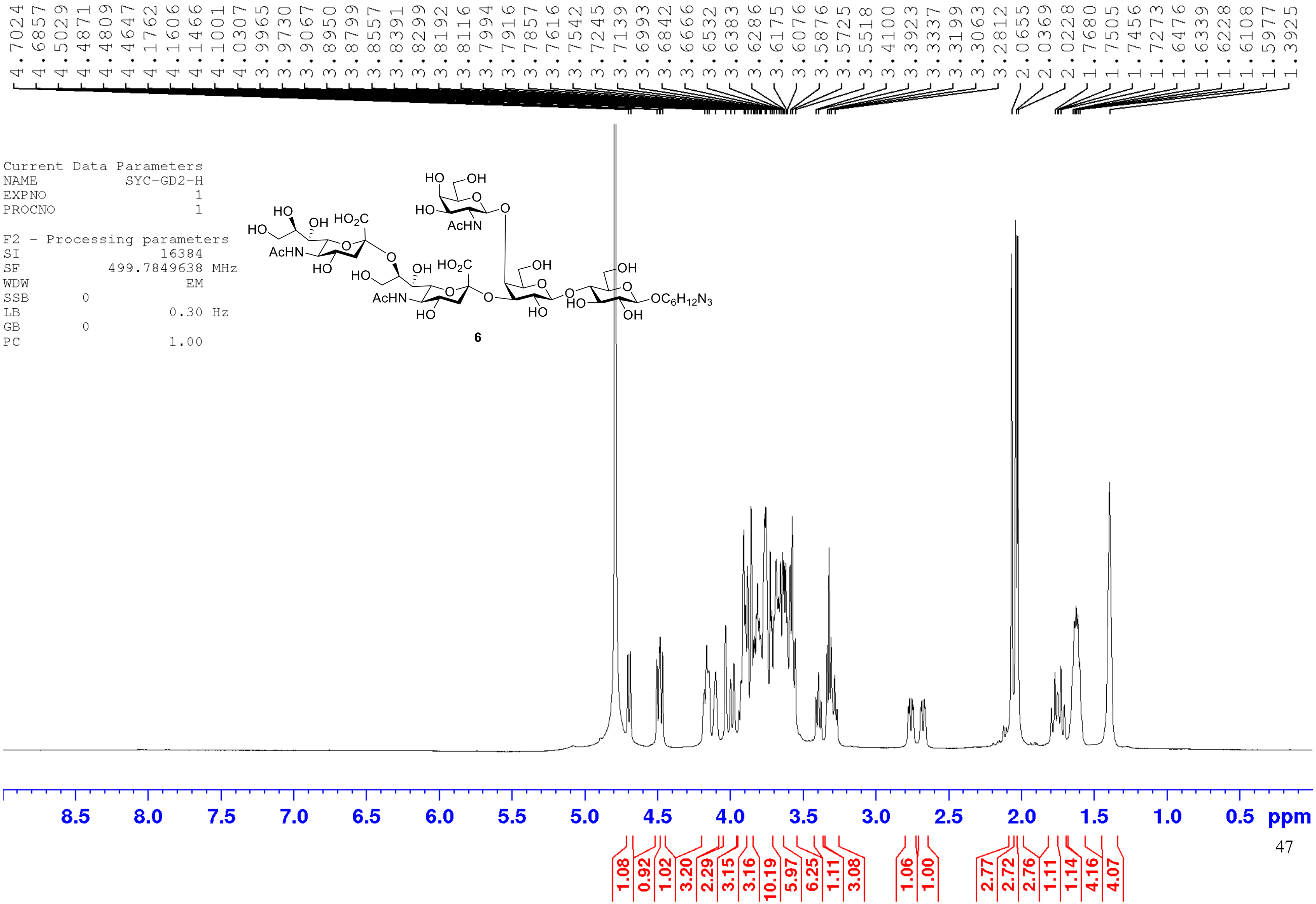

^{1}H NMR spectrum of **6** (**GD2**) (500 MHz D_2O)

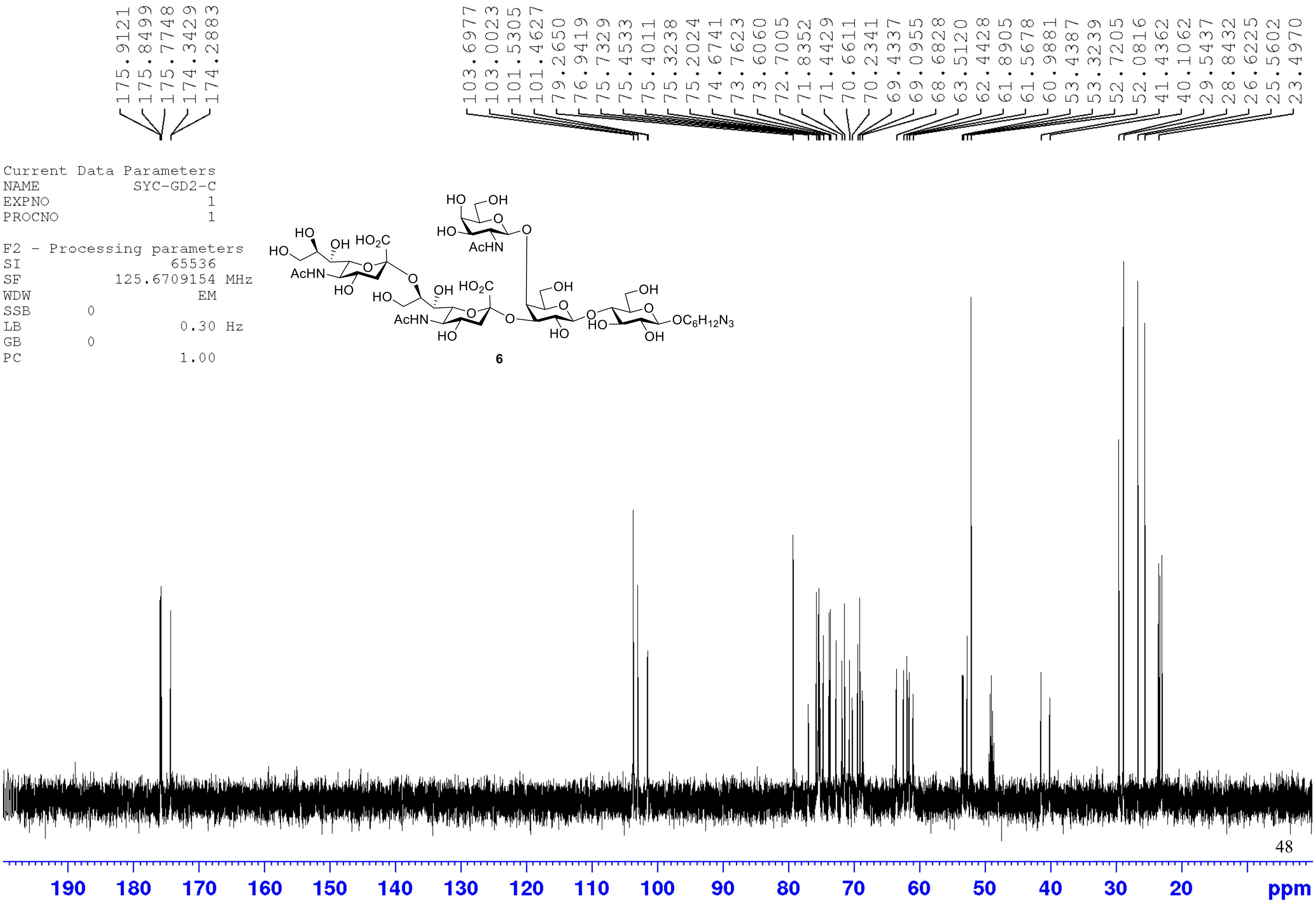

^{13}C NMR spectrum of **6** (**GD2**) (125 MHz D_2O)

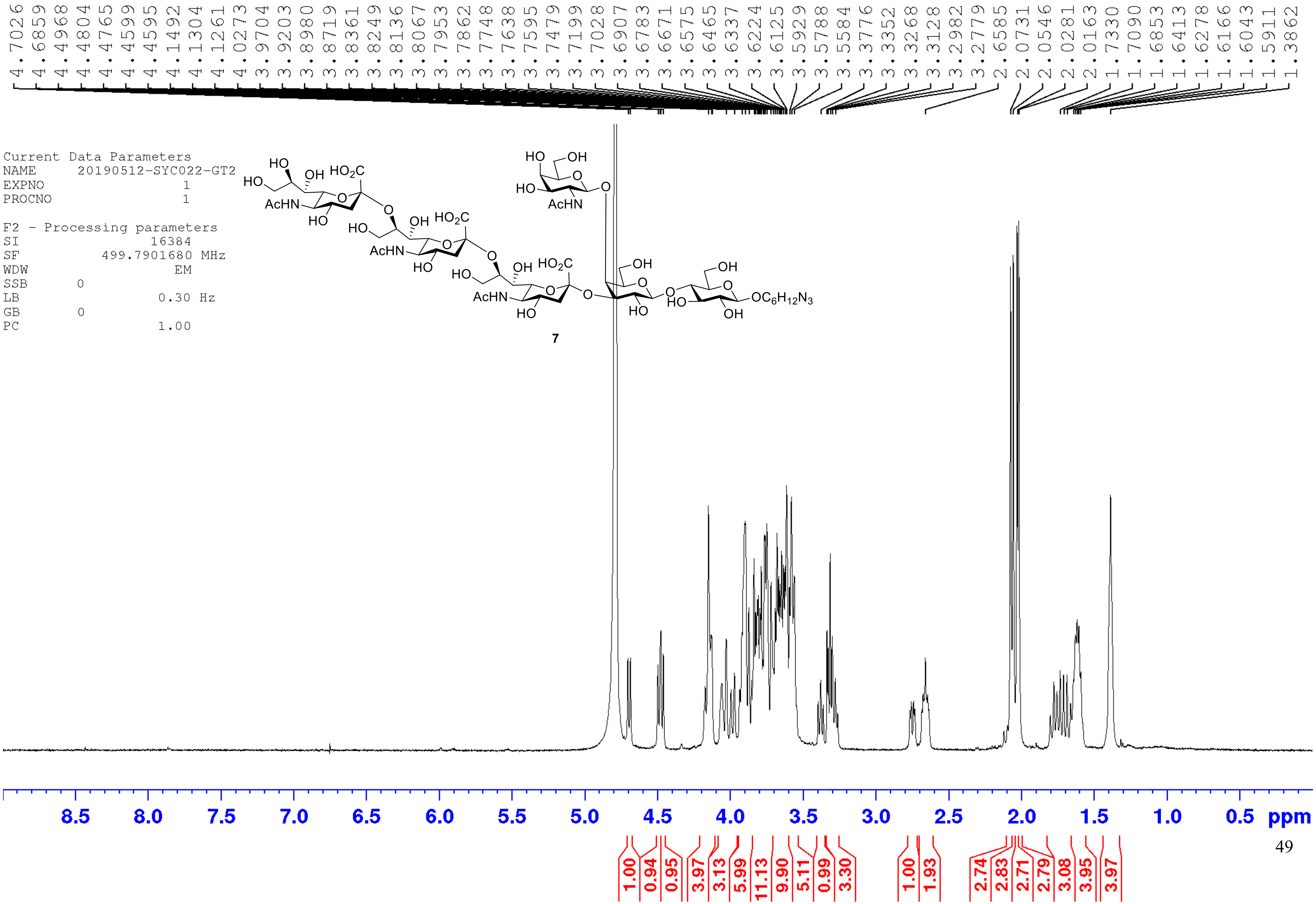

^{1}H NMR spectrum of 7 (**GT2**) (500 MHz D_2O)

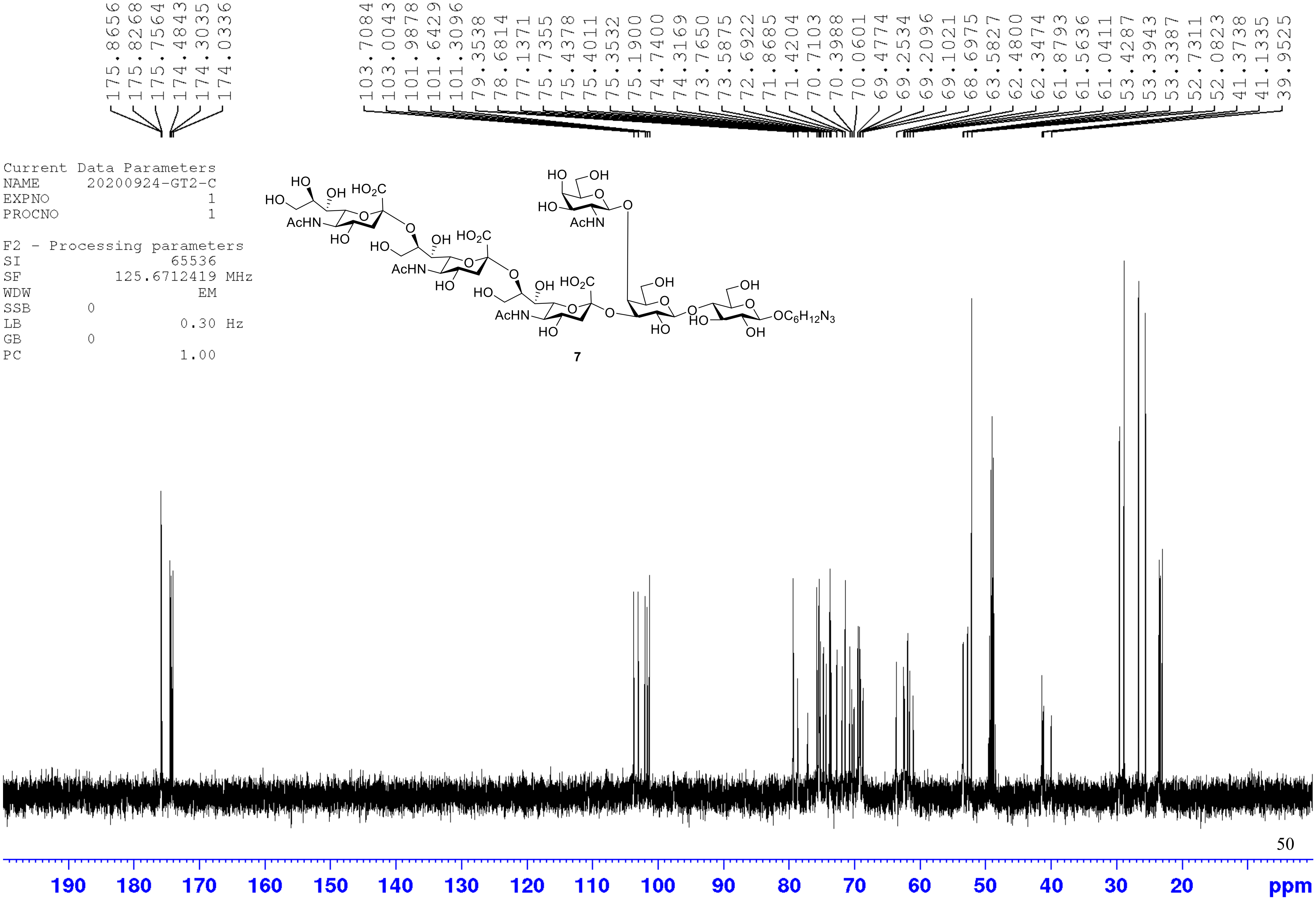

^{13}C NMR spectrum of **7** (**GT2**) (125 MHz D_2O)

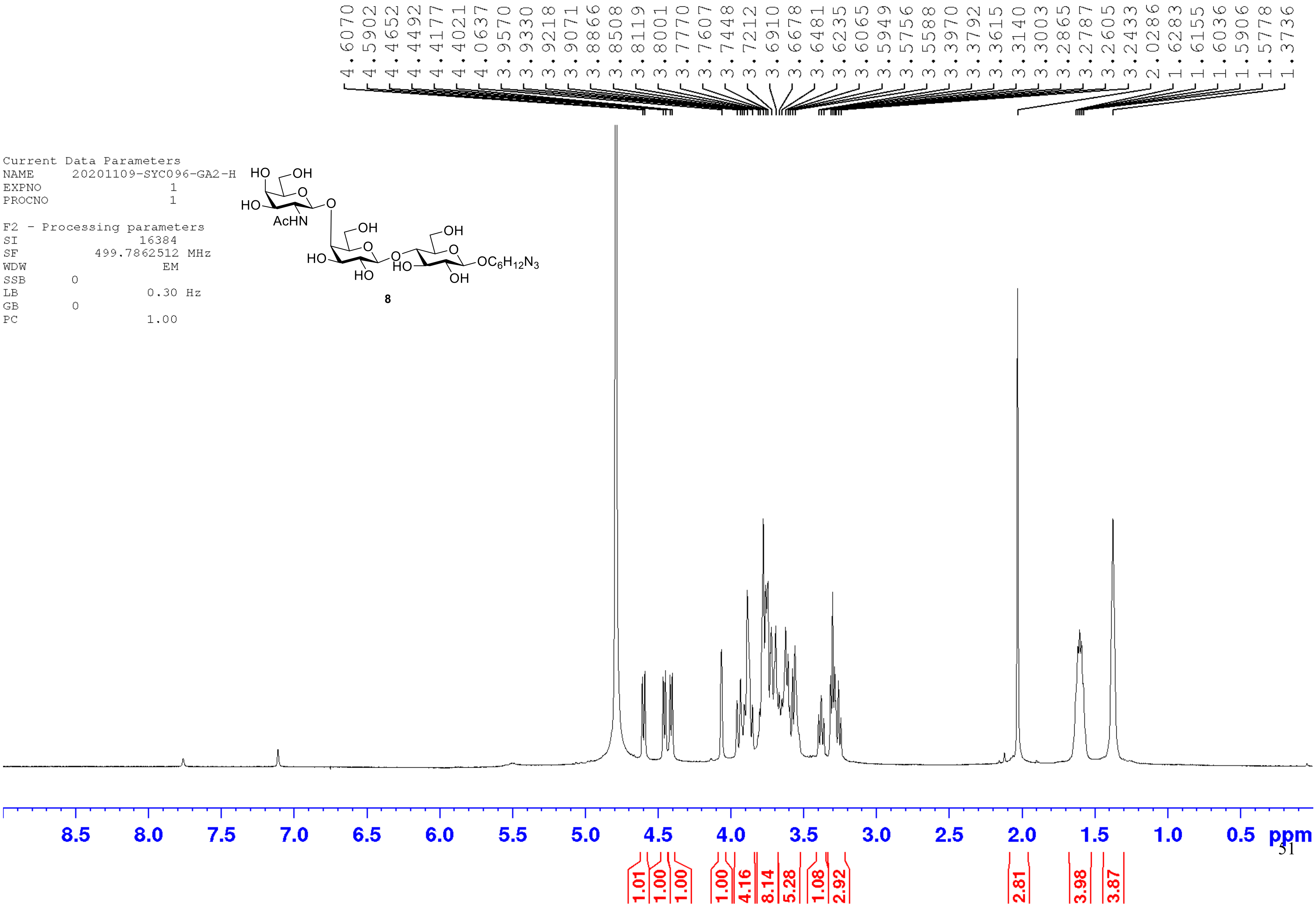

^{1}H NMR spectrum of **8** (**GA2**) (500 MHz D_2O)

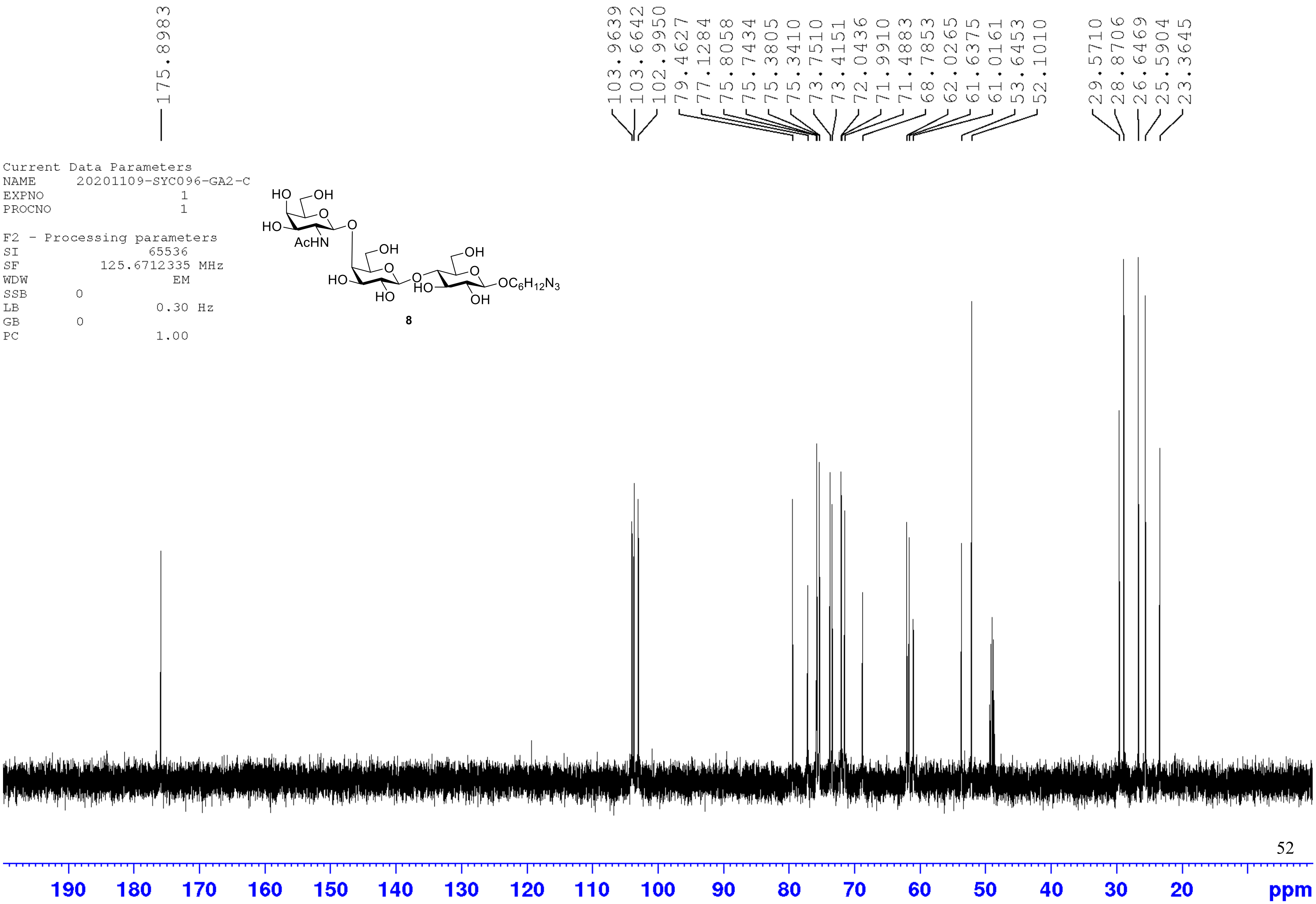

^{13}C NMR spectrum of **8** (**GA2**) (125 MHz D_2O)

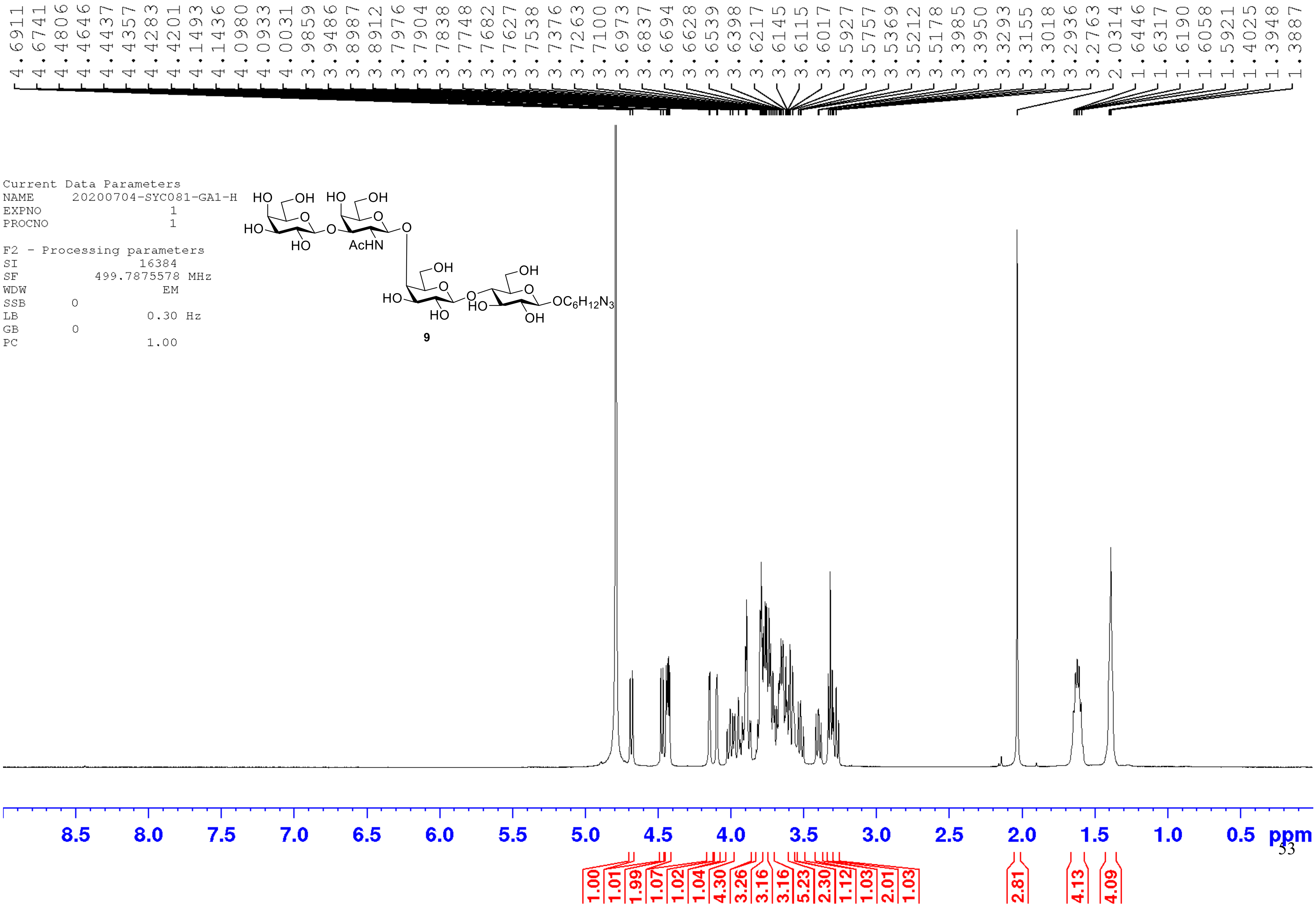

^{1}H NMR spectrum of **9** (**GA1**) (500 MHz D_2O)

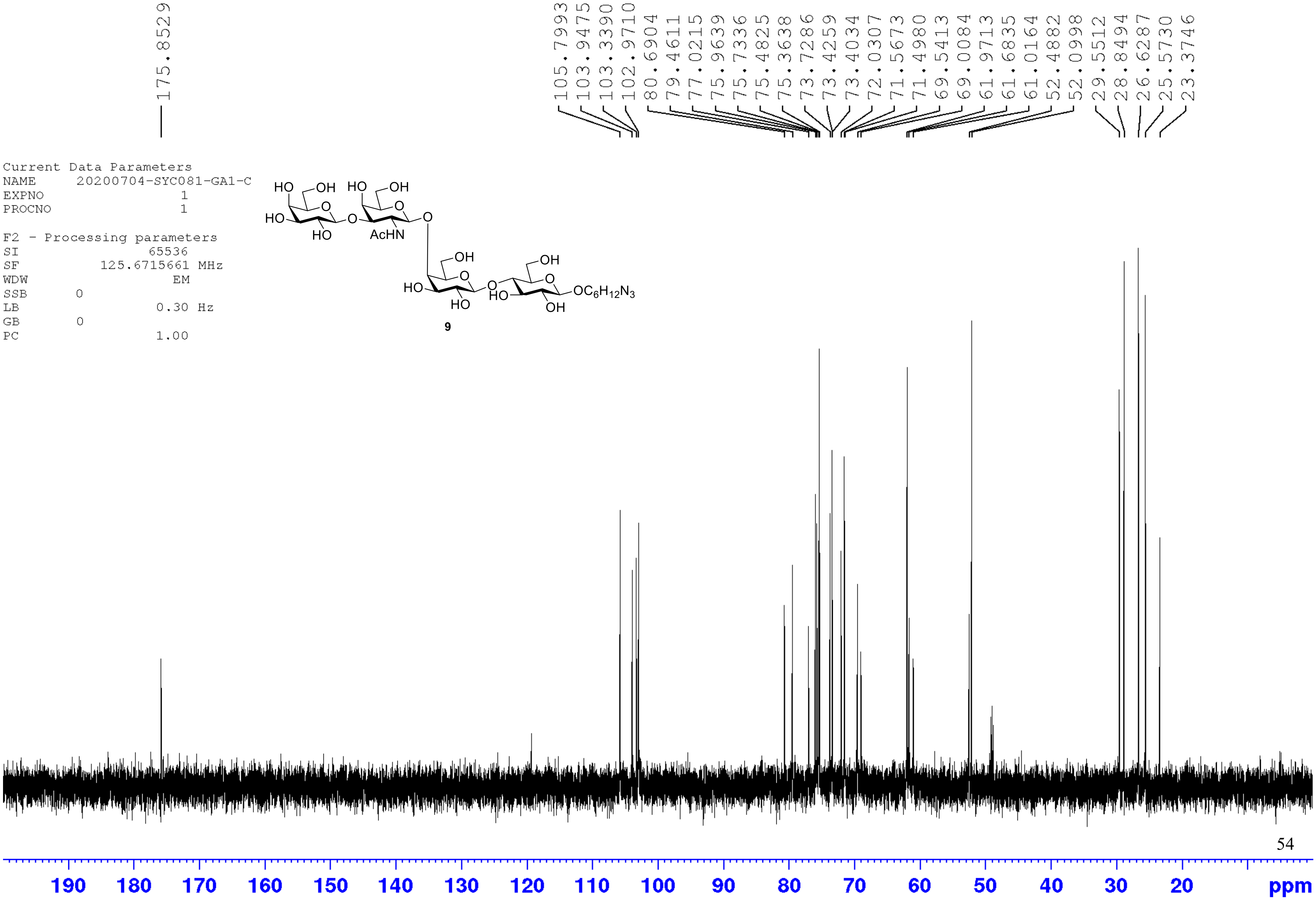

^{13}C NMR spectrum of **9** (**GA1**) (125 MHz D_2O)

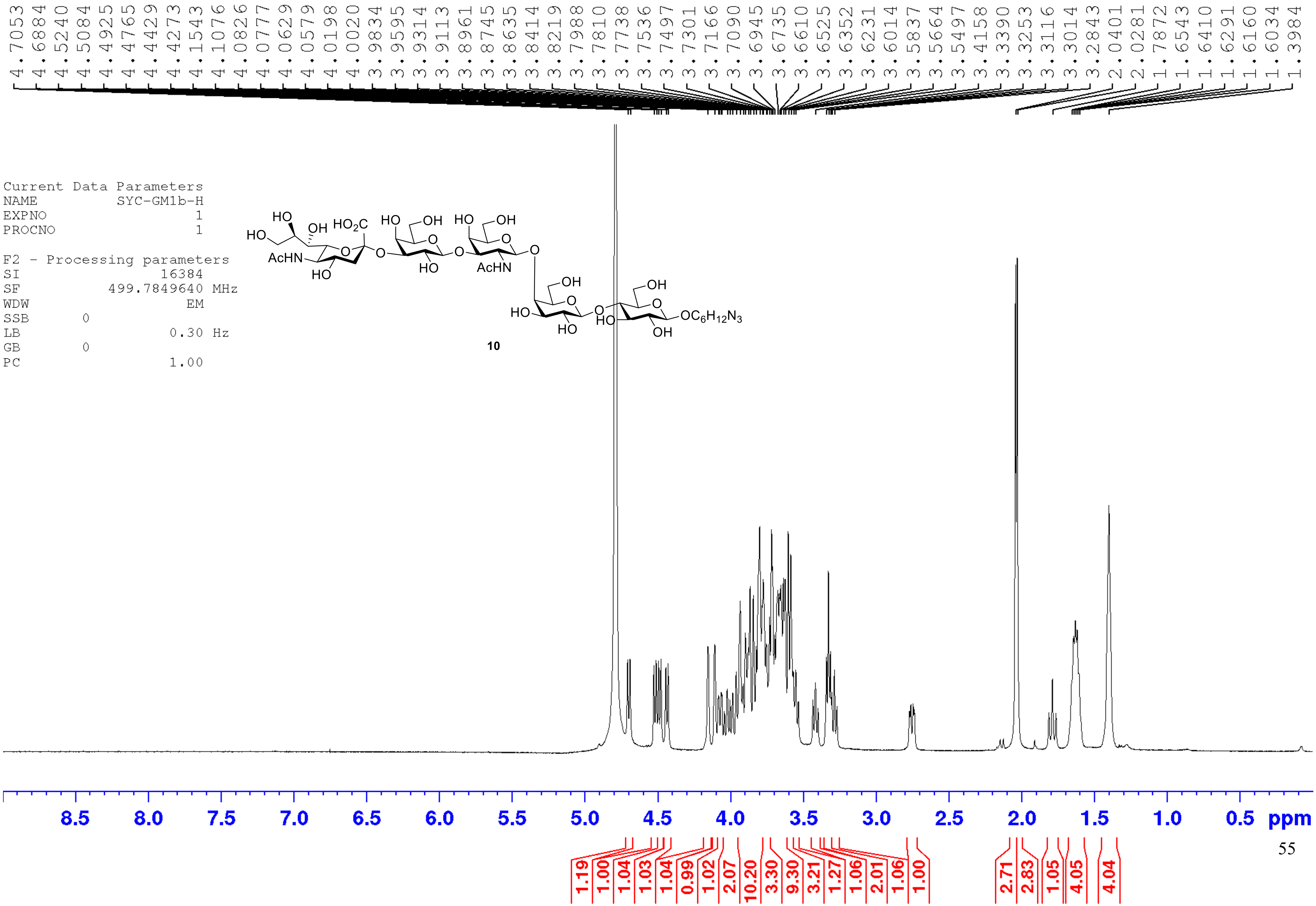

^{1}H NMR spectrum of **10** (**GM1b**) (500 MHz D_2O)

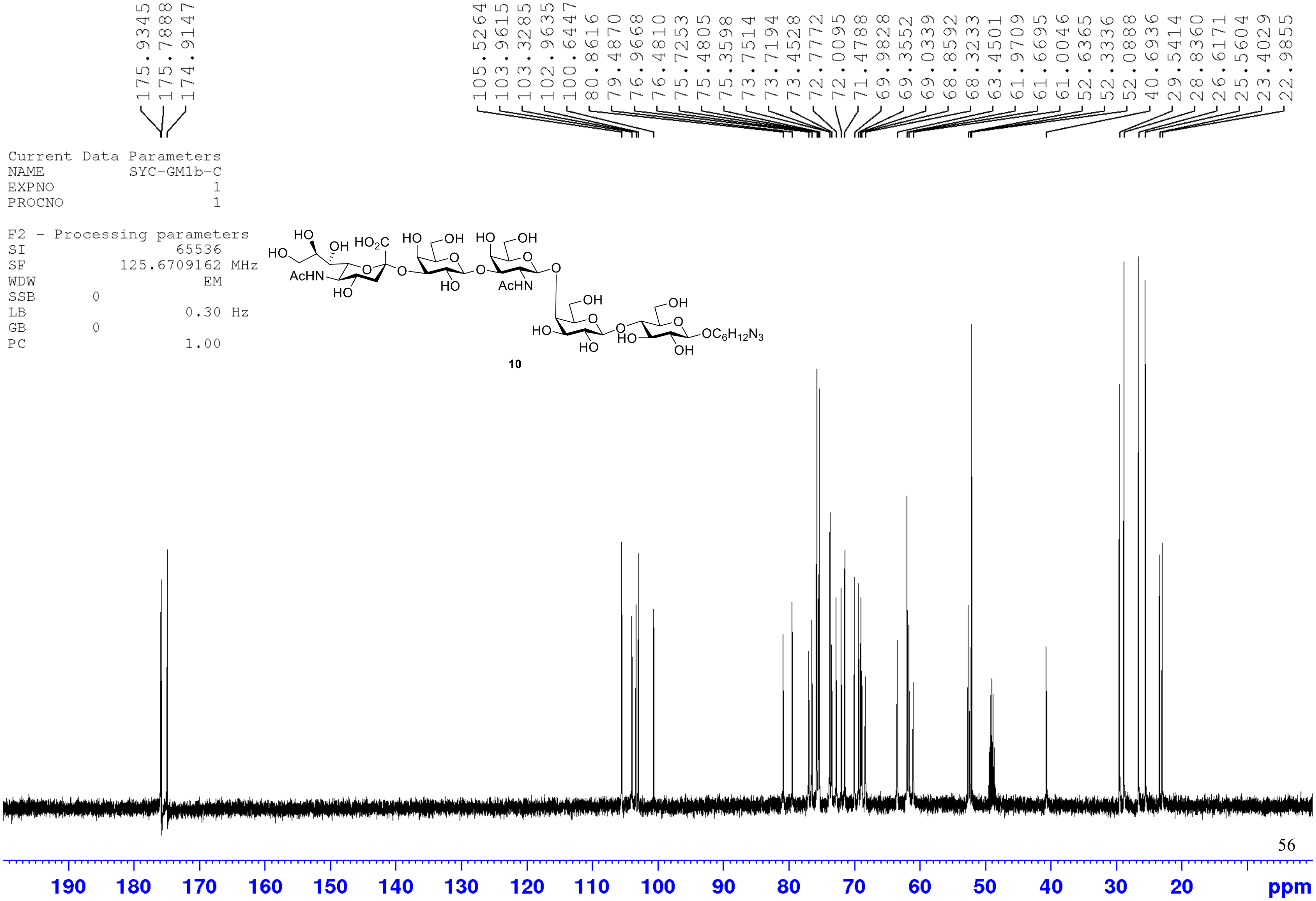

^{13}C NMR spectrum of **10 (GM1b)** (125 MHz D_2O)

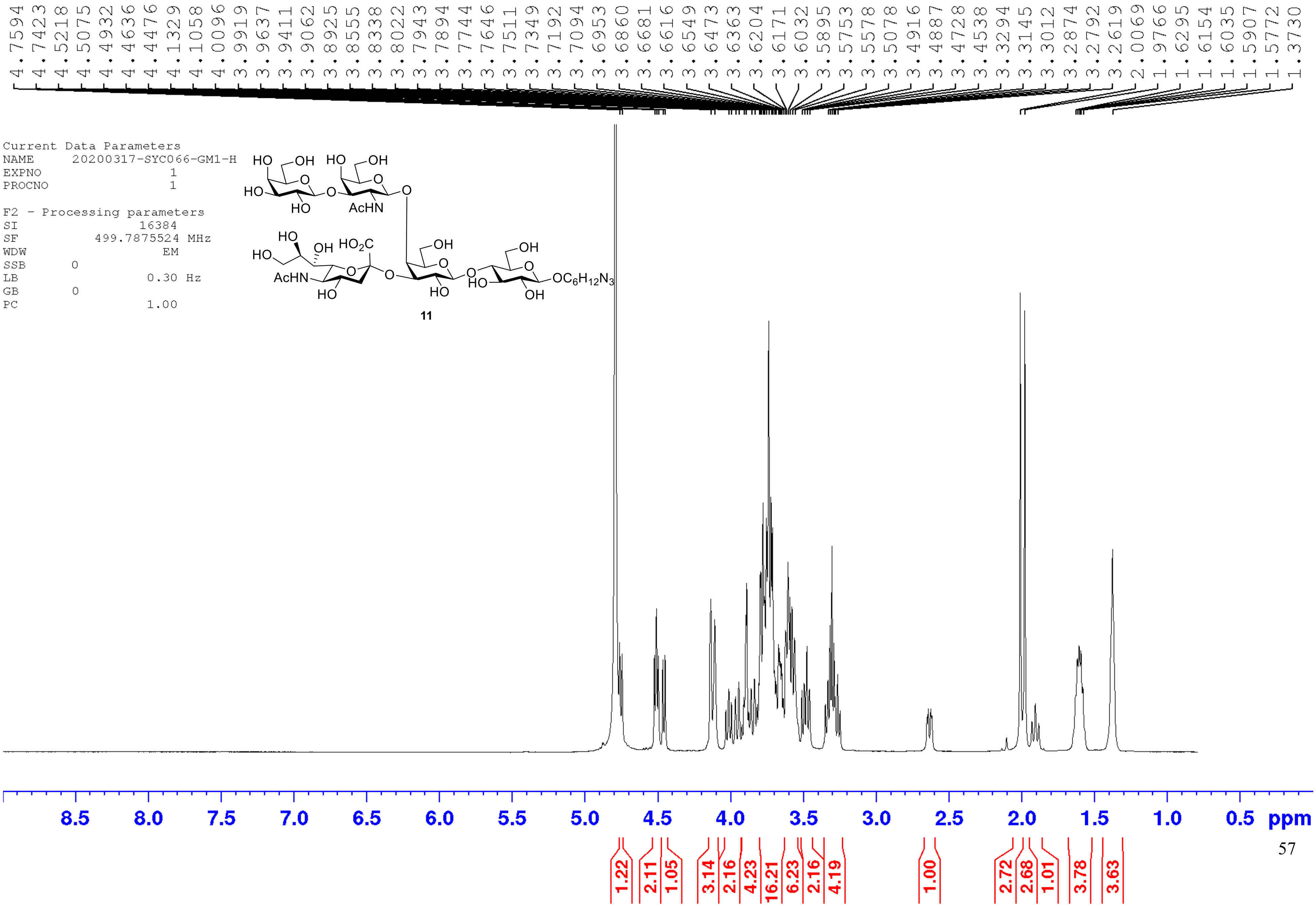

^{1}H NMR spectrum of **11** (**GM1a**) (500 MHz D_2O)

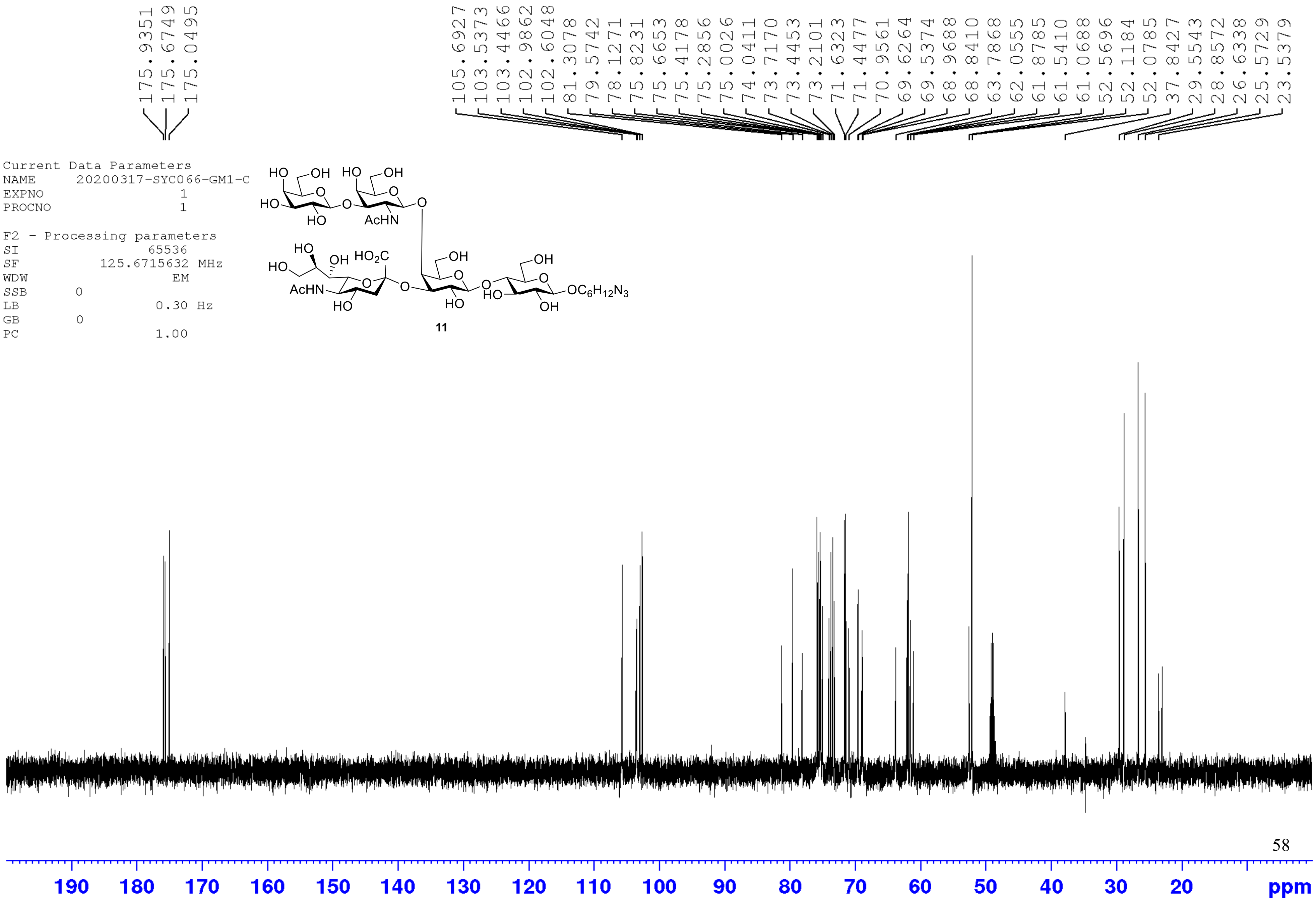

^{13}C NMR spectrum of **11** (**GM1a**) (125 MHz D_2O)

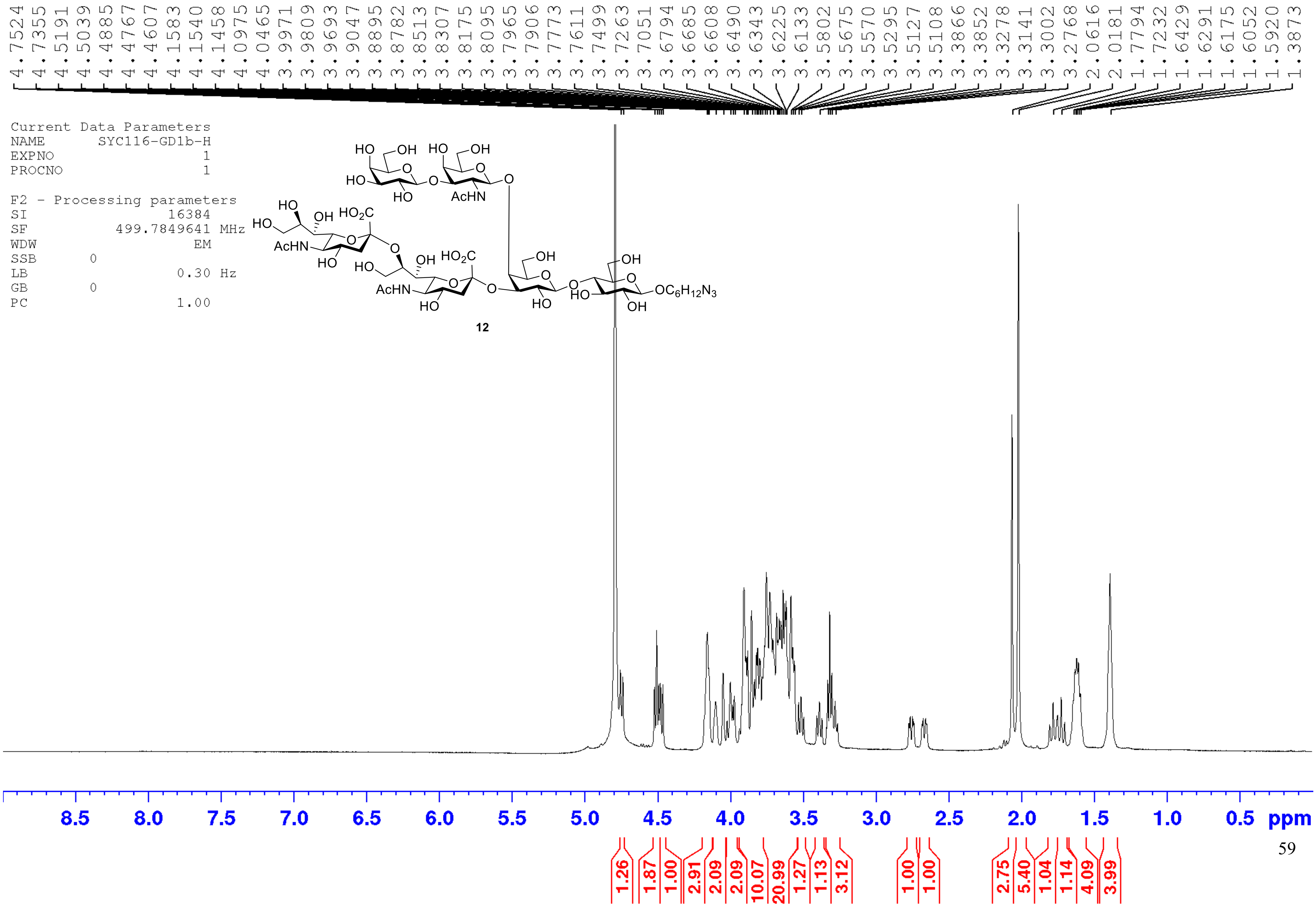

^{1}H NMR spectrum of **12** (**GD1b**) (500 MHz D_2O)

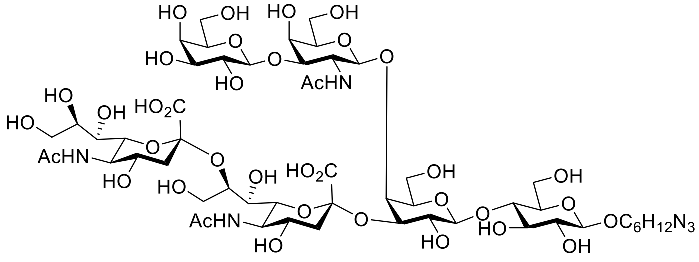

12

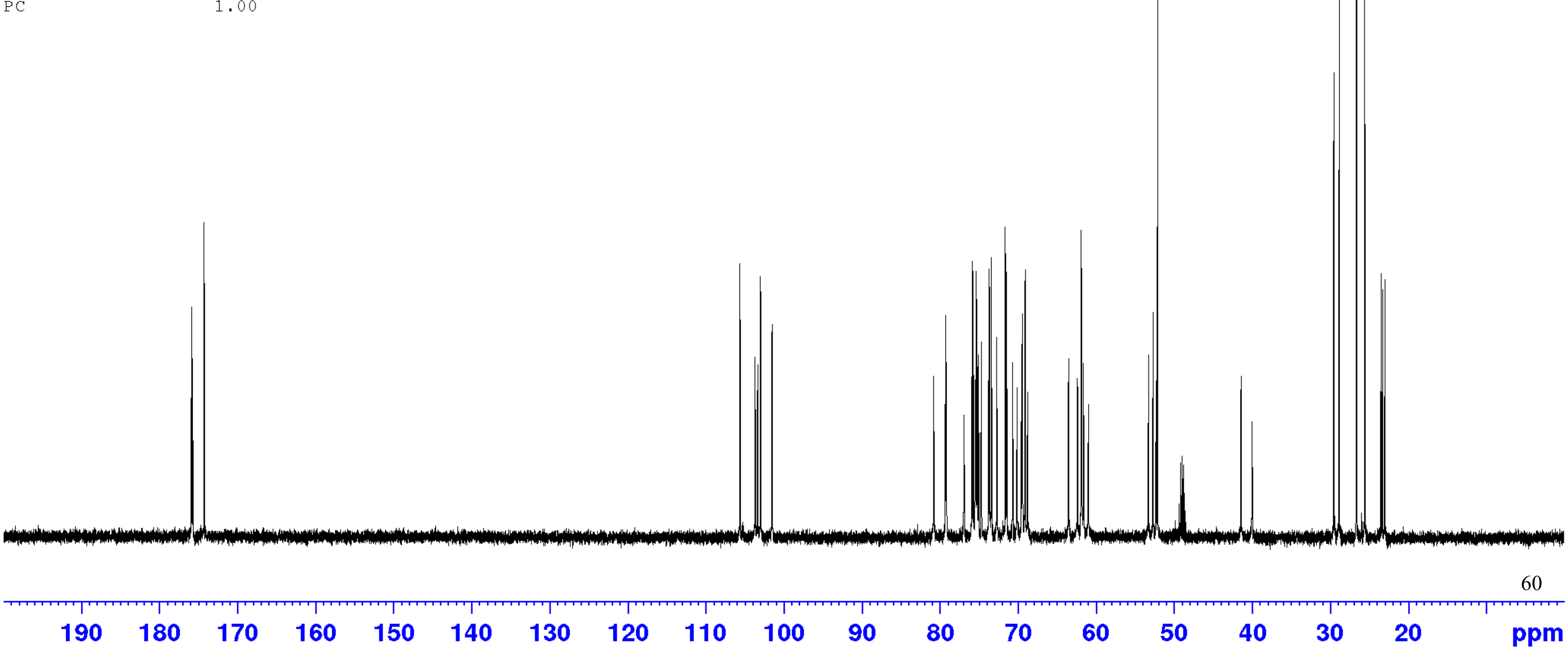

^{13}C NMR spectrum of **12** (**GD1b**) (125 MHz D_2O)

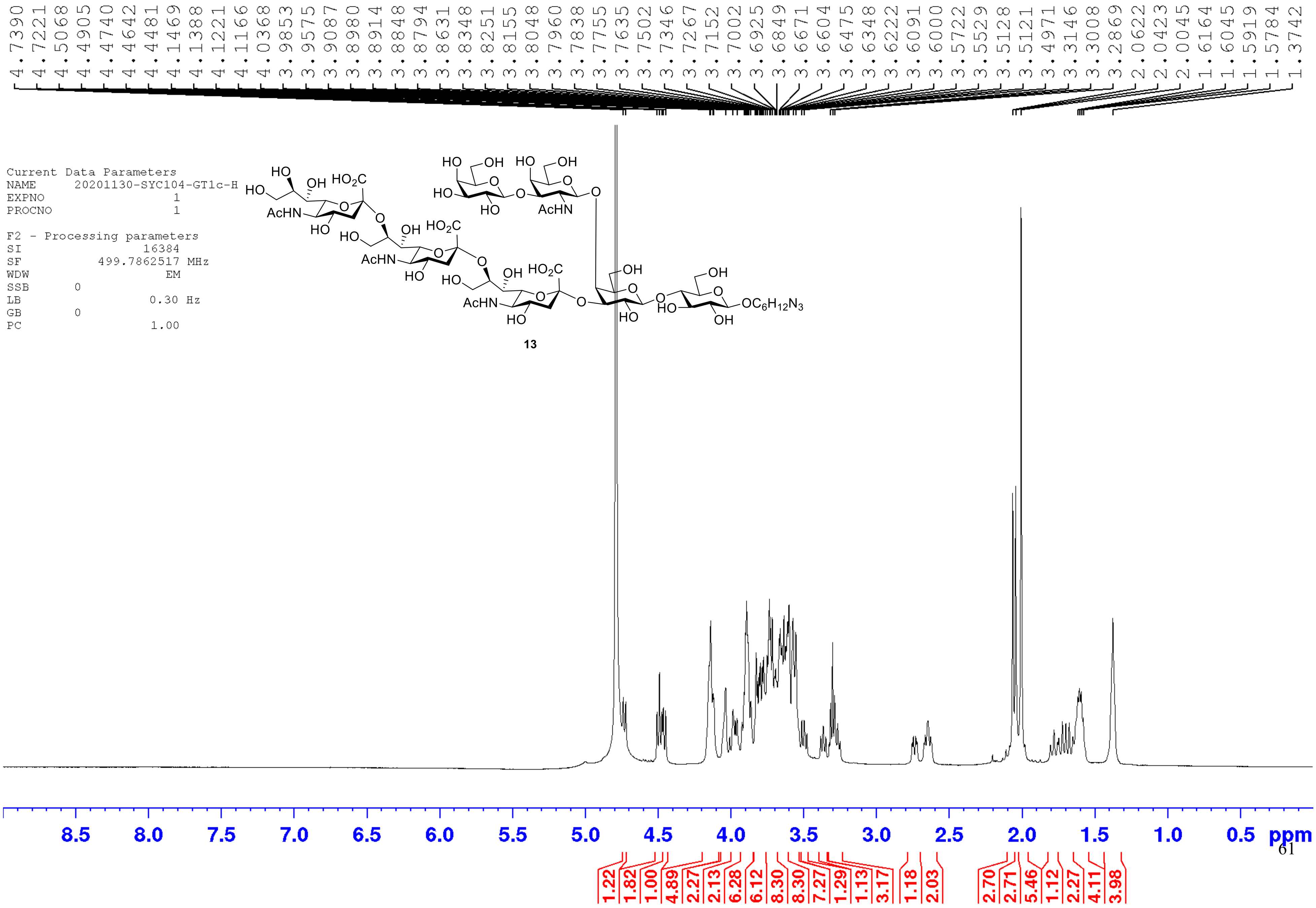

^{1}H NMR spectrum of **13** (**GT1c**) (500 MHz D_2O)

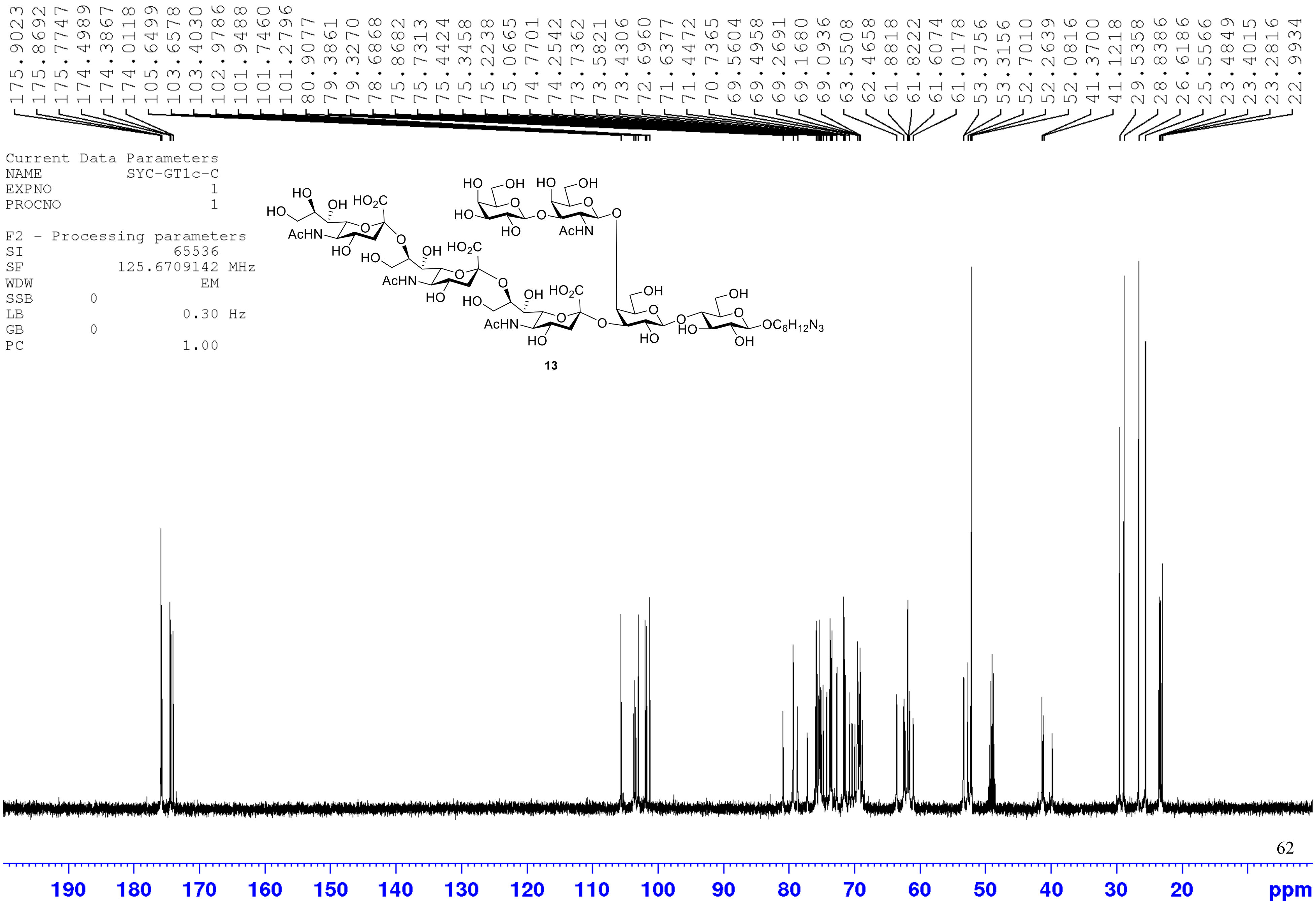

^{13}C NMR spectrum of **13** (**GT1c**) (125 MHz D_2O)

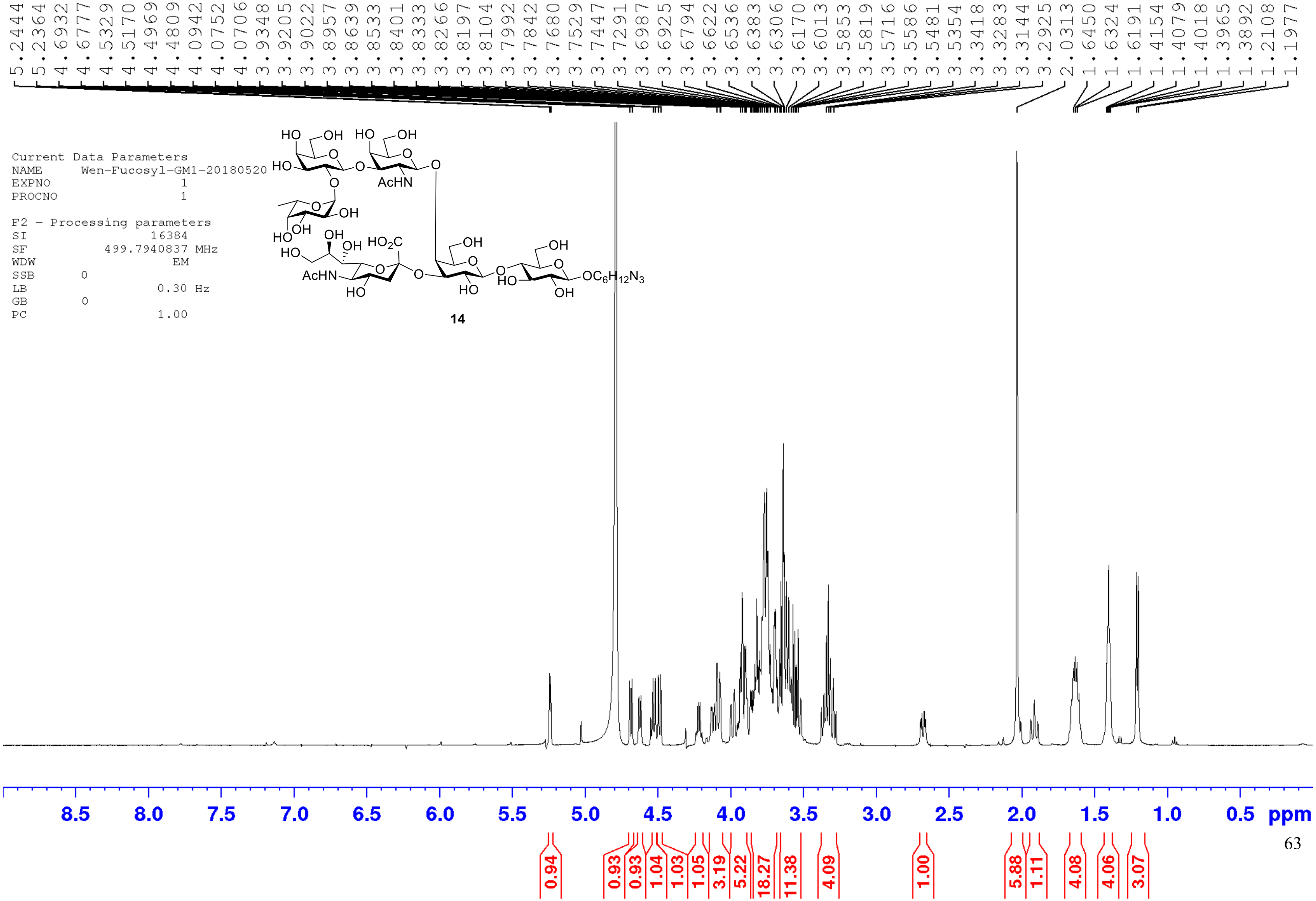

^{1}H NMR spectrum of **14** (**Fucosyl GM1**) (500 MHz D_2O)

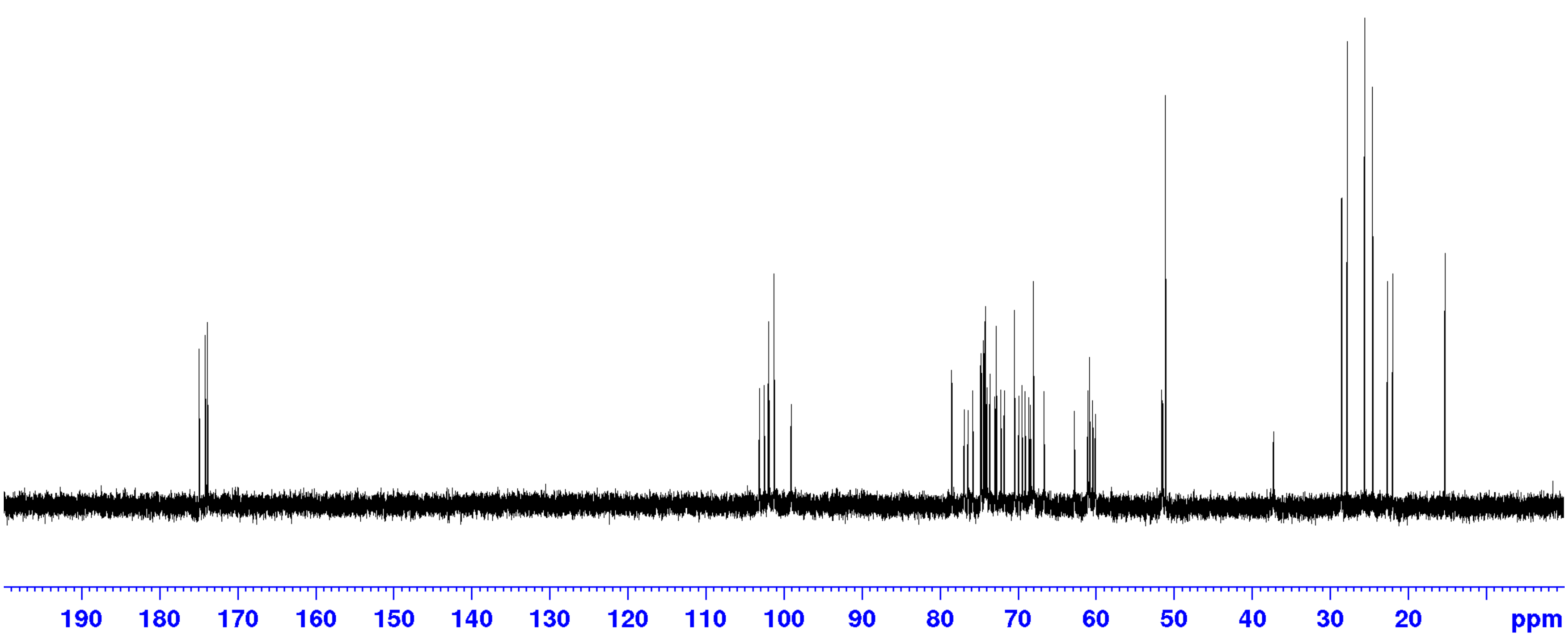


```
Current Data Parameters
NAME     Wen211-Fucosyl-GM1-20171029
EXPNO                      1
PROCNO                     1

F2 - Processing parameters
SI                     65536
SF            100.5201859 MHz
WDW                       EM
SSB       0
LB                   0.30 Hz
GB        0
PC                   1.00
```

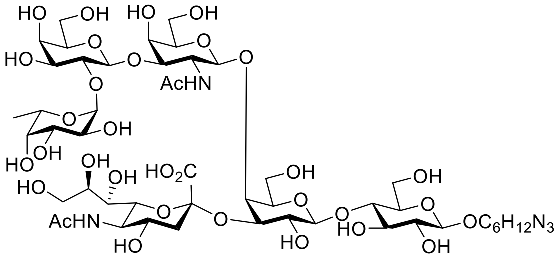

14

^{13}C NMR spectrum of **14** (**Fucosyl GM1**) (125 MHz D_2O)

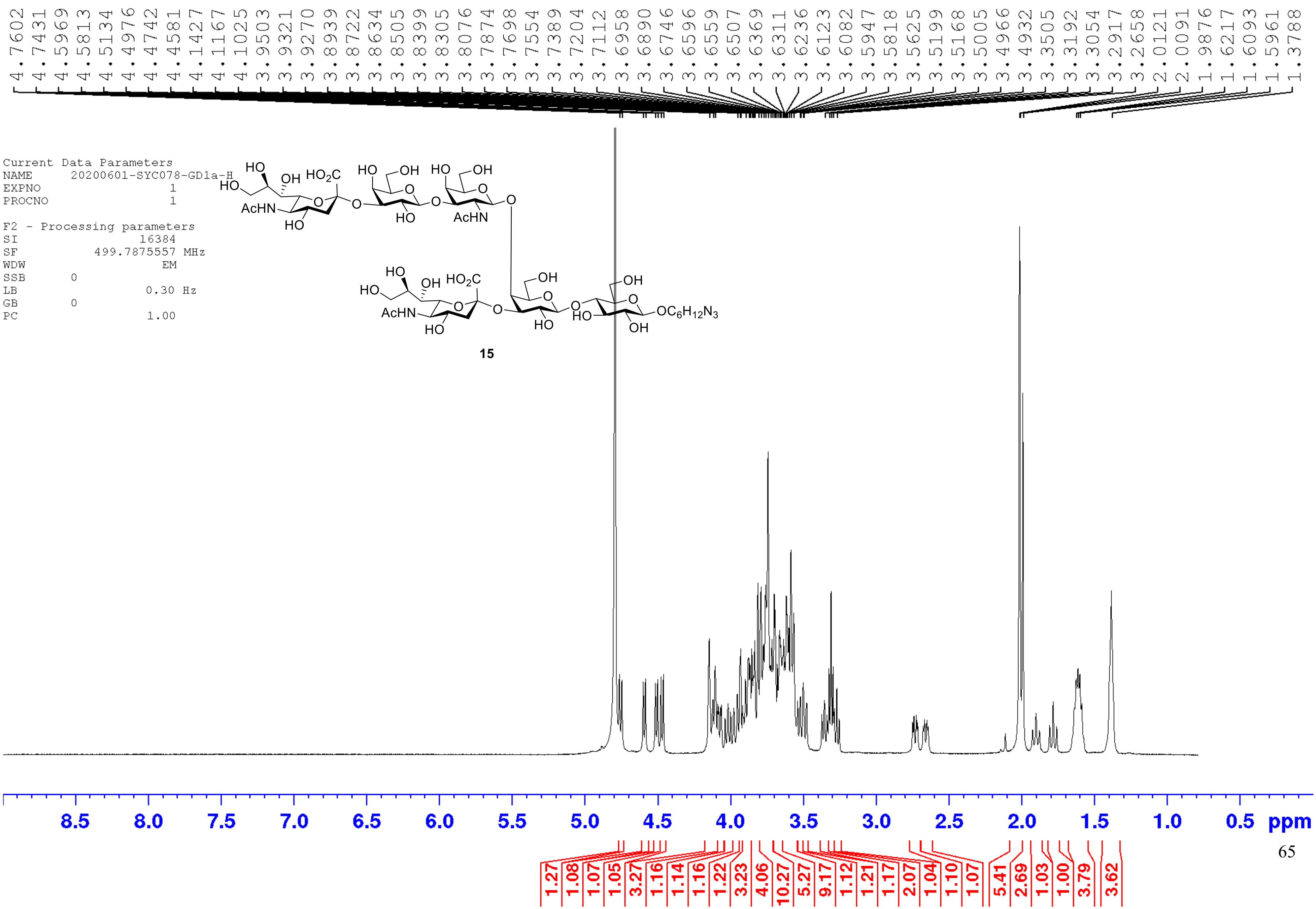

^{1}H NMR spectrum of **15** (**GD1a**) (500 MHz D_2O)

Current Data Parameters
NAME SYC-GD1a-C
EXPNO 1
PROCNO 1

F2 - Processing parameters
SI 65536
SF 125.6709185 MHz
WDW EM
SSB 0
LB 0.30 Hz
GB 0
PC 1.00

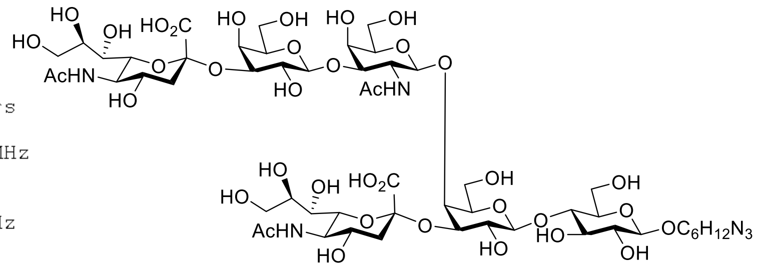

15

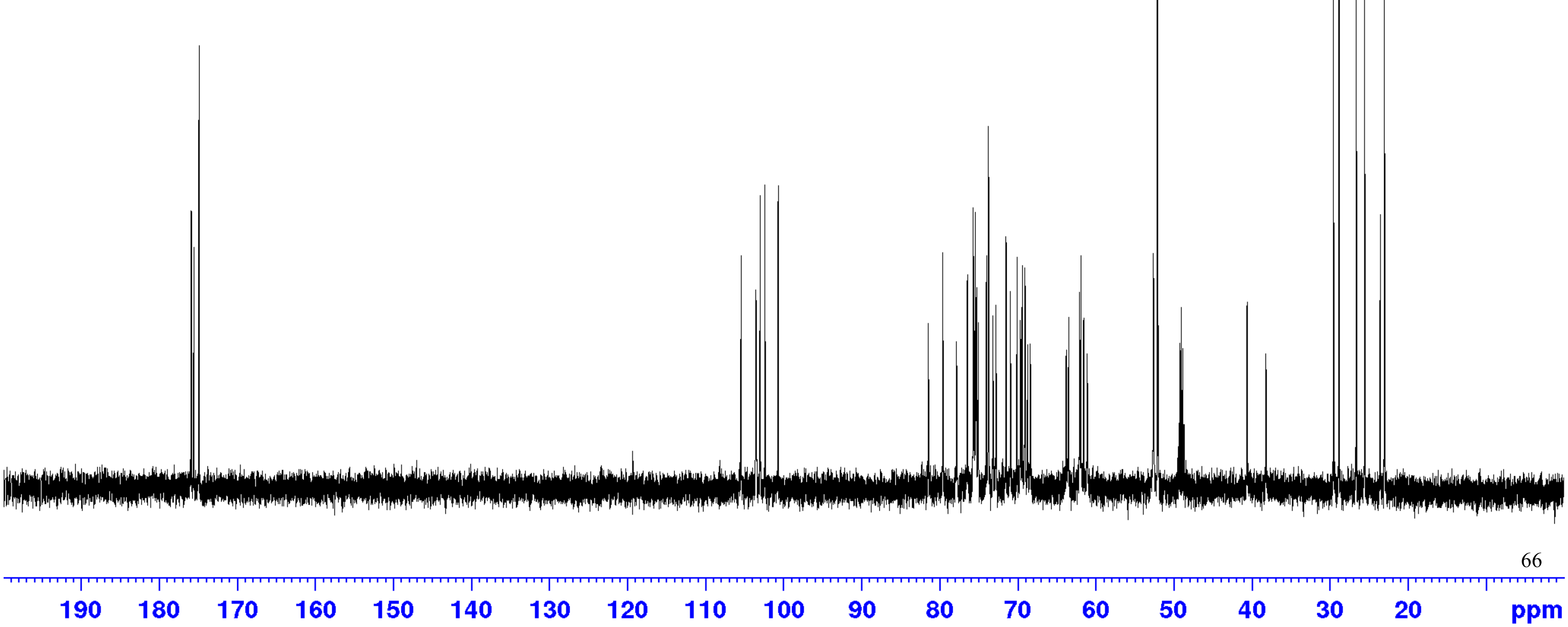

^{13}C NMR spectrum of **15 (GD1a)** (125 MHz D_2O)

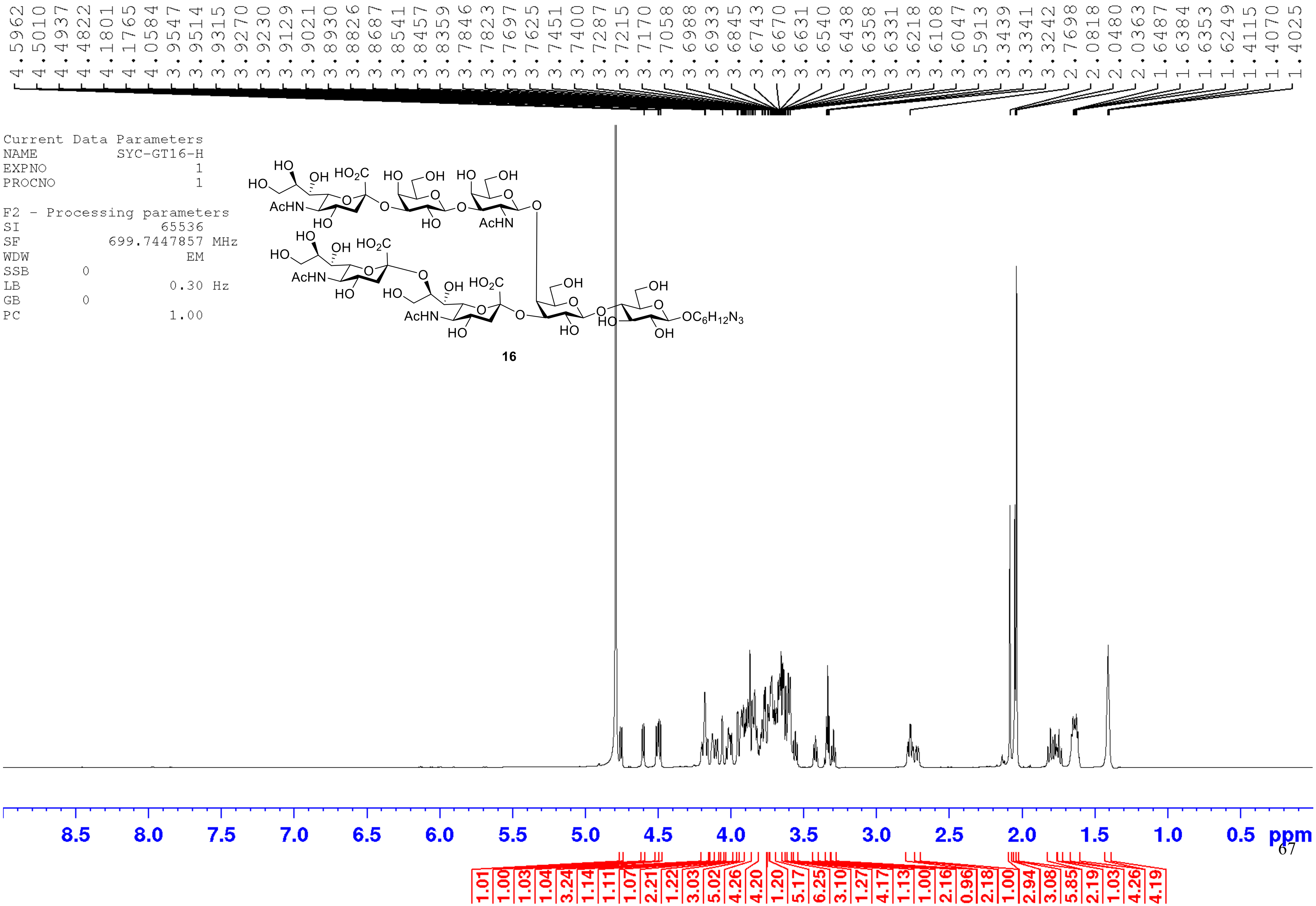

16

^{1}H NMR spectrum of **16** (**GT1b**) (700 MHz D_2O)

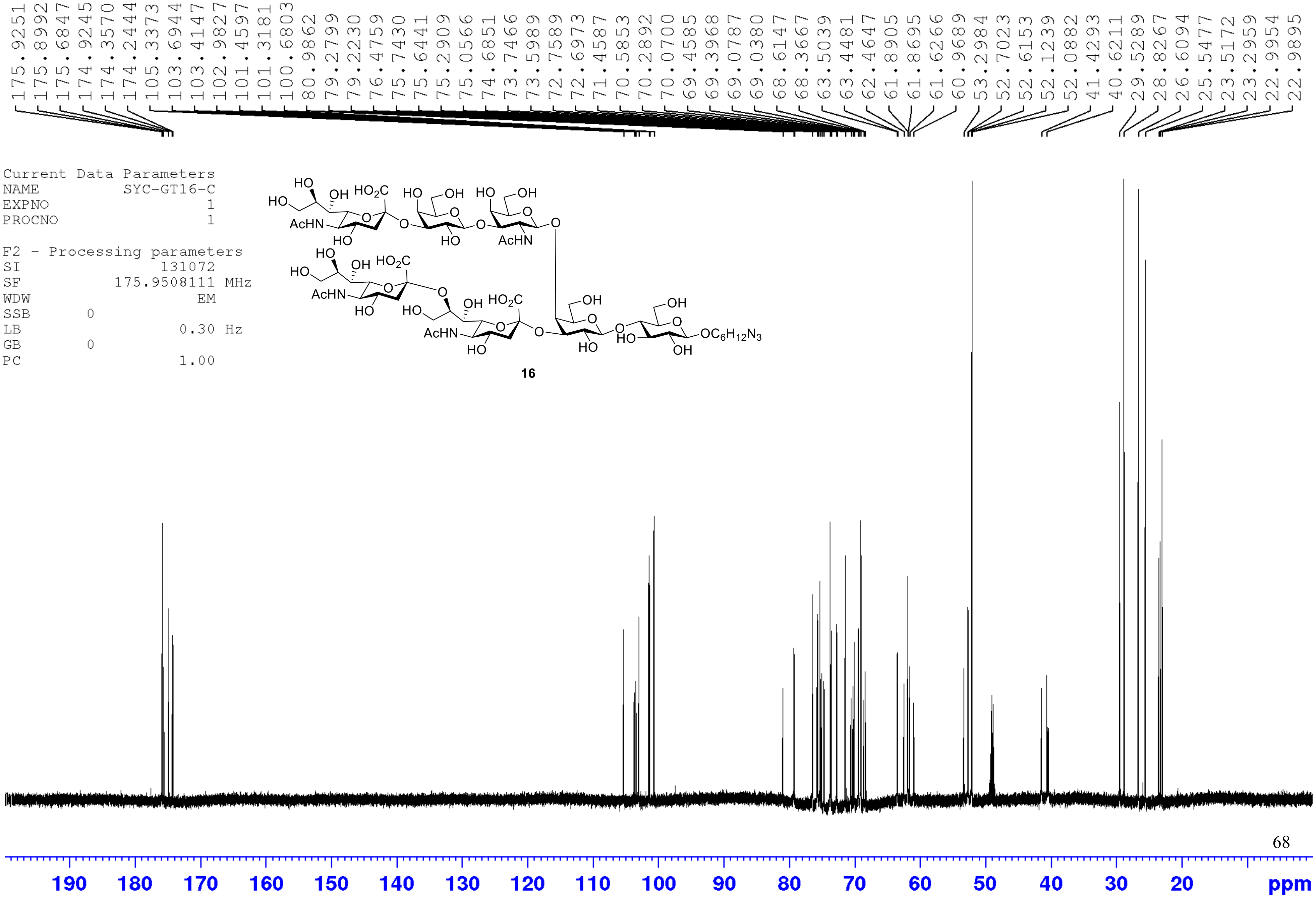

16

^{13}C NMR spectrum of **16 (GT1b)** (175 MHz D_2O)

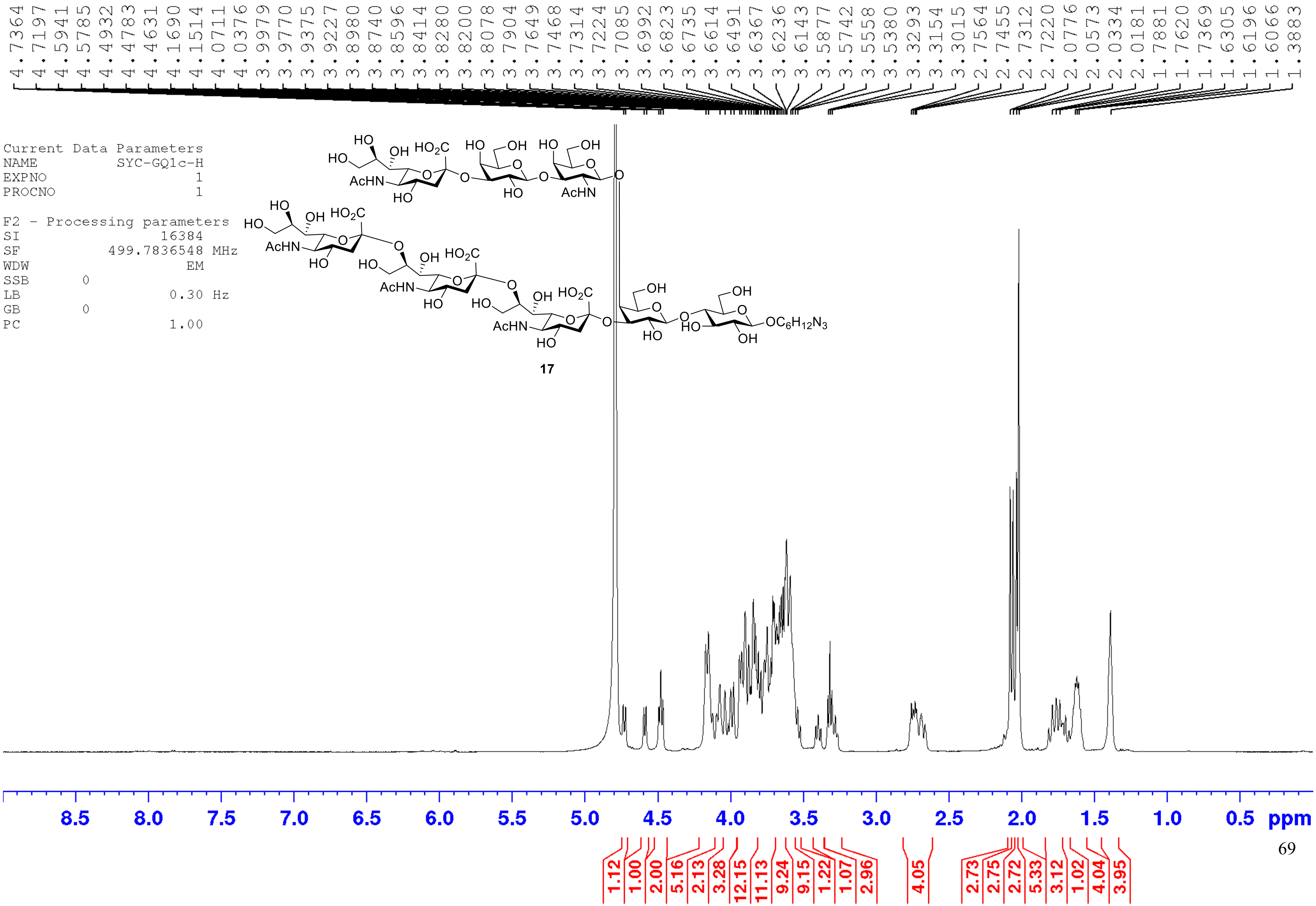

^1H NMR spectrum of **17** (**GQ1c**) (500 MHz D_2O)

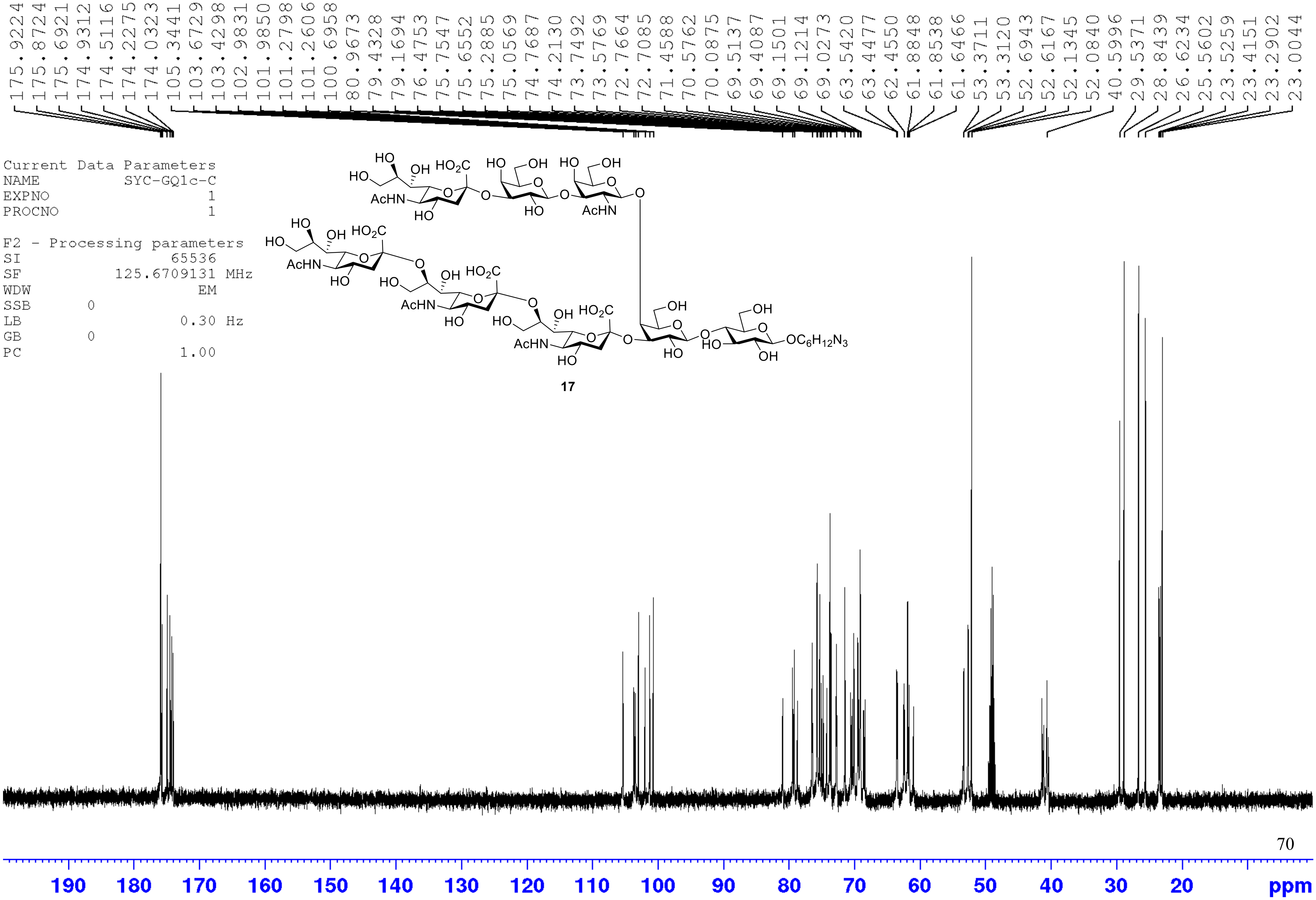

^{13}C NMR spectrum of **17** (**GQ1c**) (125 MHz D_2O)

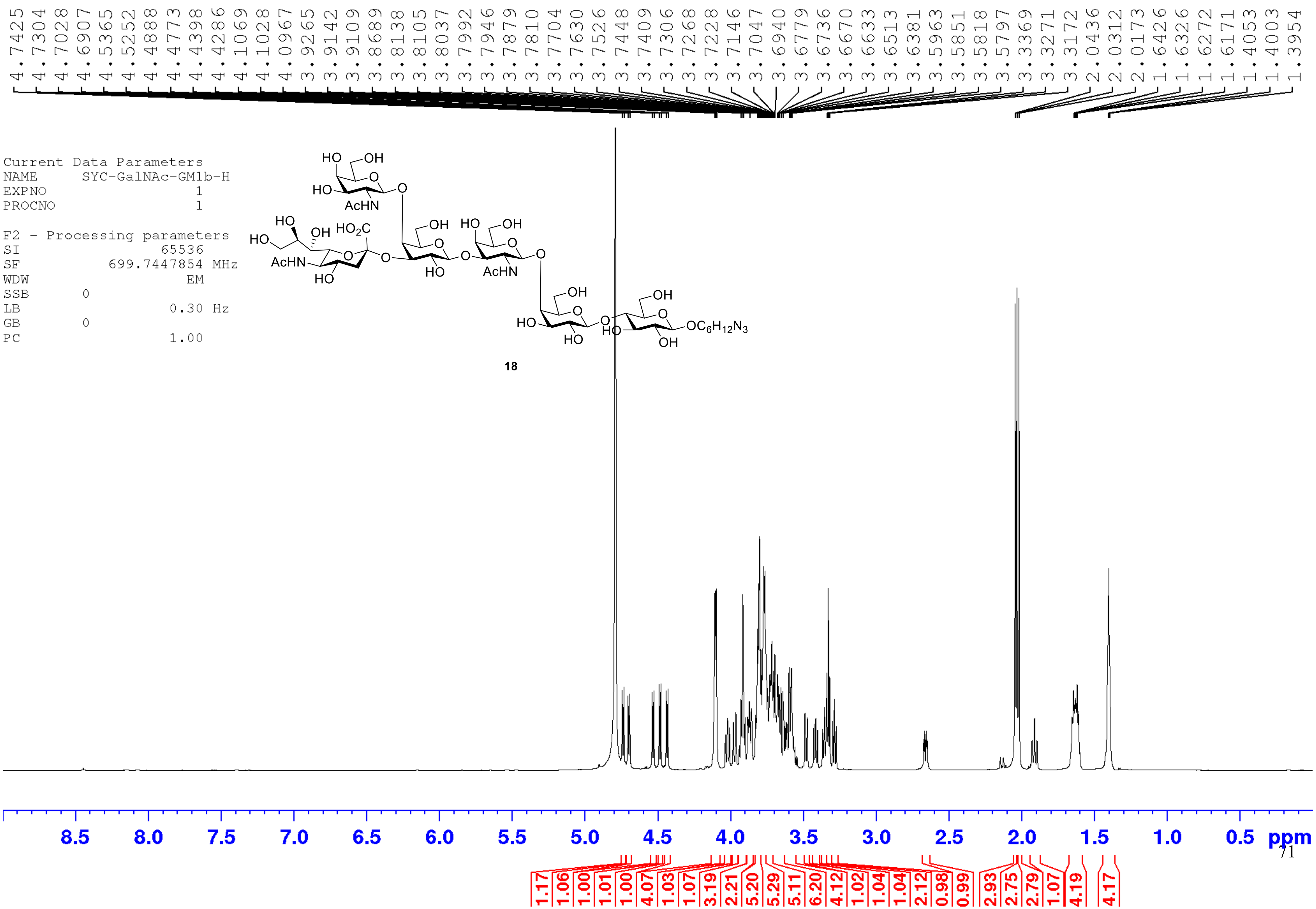

^{1}H NMR spectrum of **18** (**GalNAc-GM1b**) (700 MHz D_2O)

Current Data Parameters
NAME SYC-GalNAc-GM1b-C
EXPNO 1
PROCNO 1

F2 - Processing parameters
SI 131072
SF 175.9508085 MHz
WDW EM
SSB 0
LB 0.30 Hz
GB 0
PC 1.00

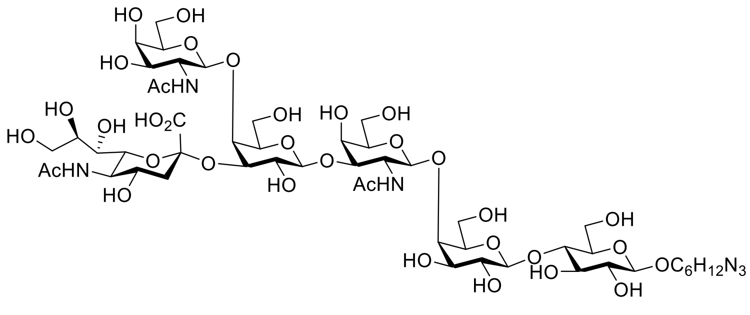

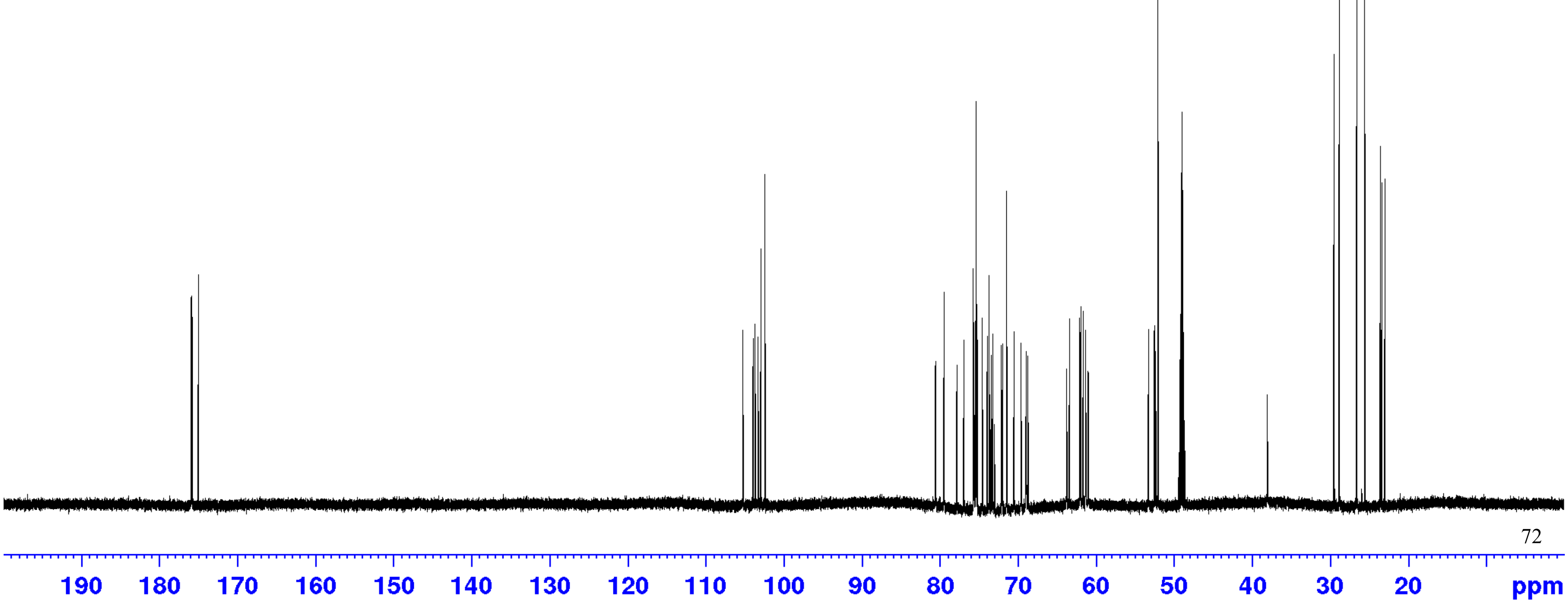

18

^{13}C NMR spectrum of **18** (**GalNAc-GM1b**) (175 MHz D$_2$O)

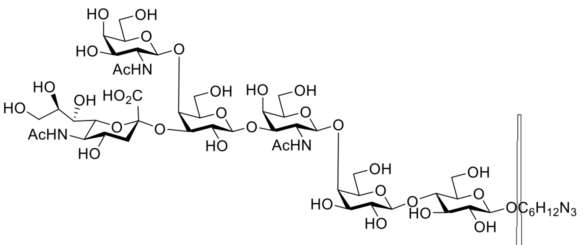

18

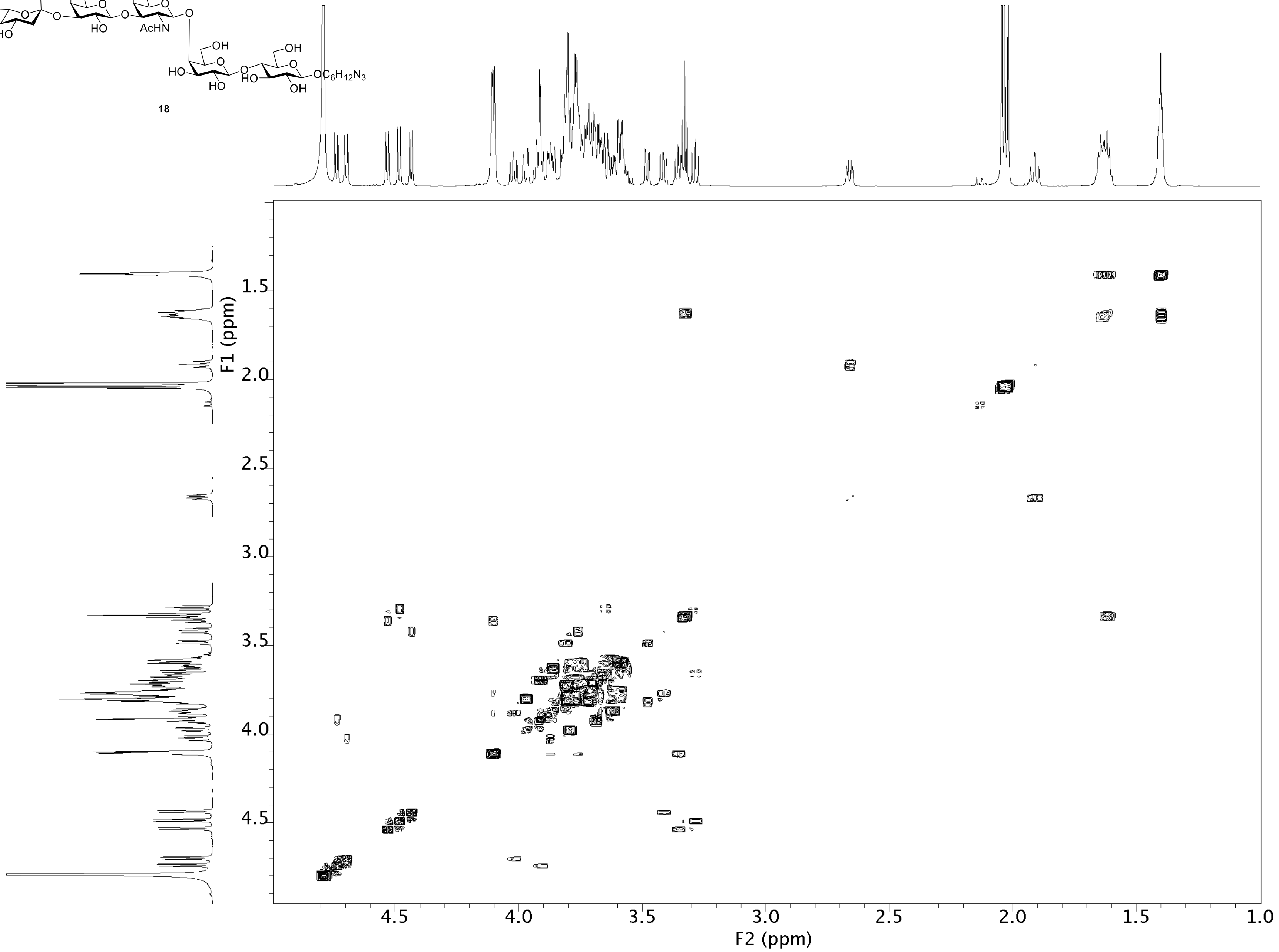

^{1}H-^{1}H COSY spectrum of **18** (**GalNAc-GM1b**) (700 MHz D_2O)

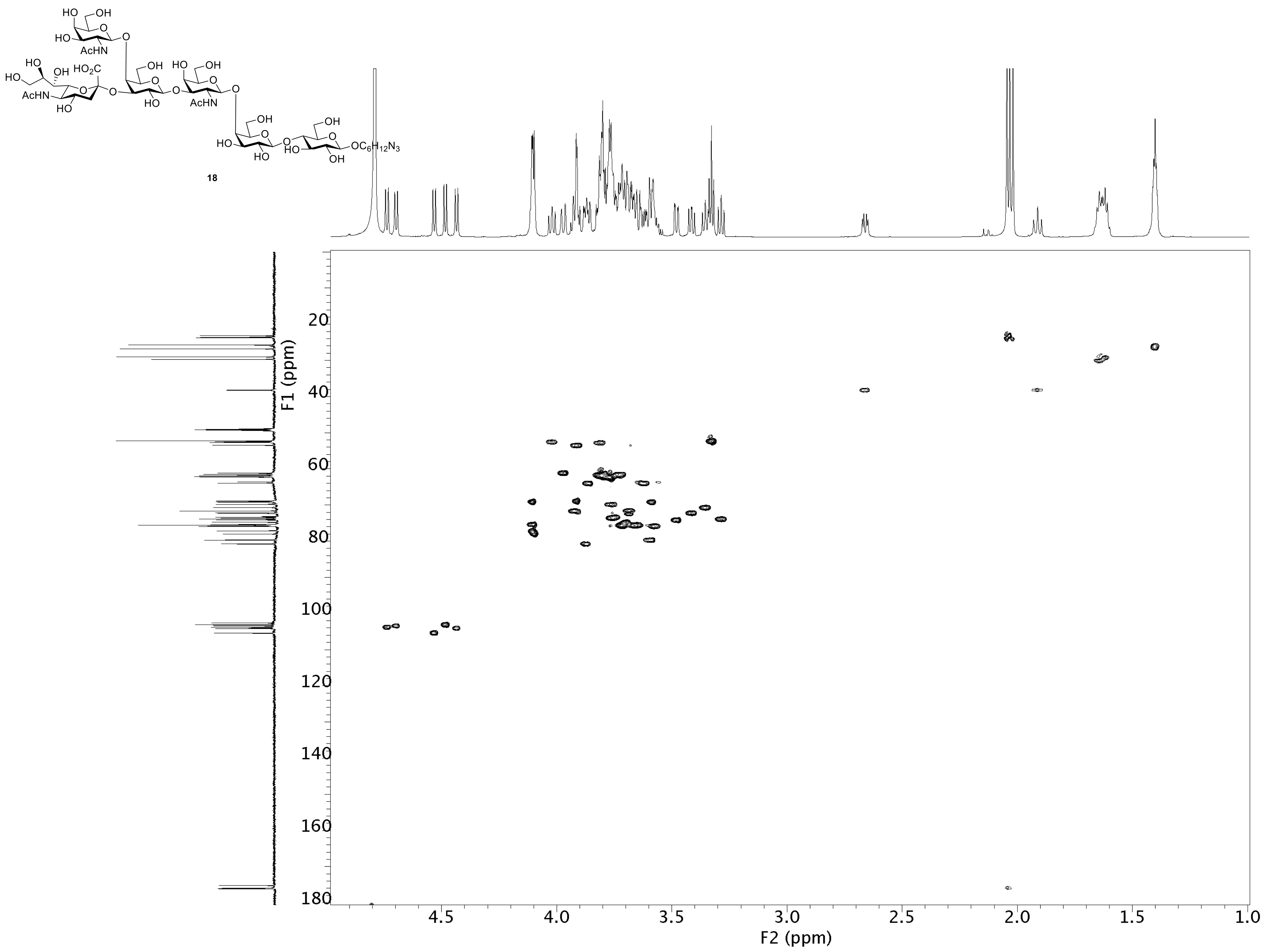

^{1}H-^{13}C HSQC spectrum of **18** (**GalNAc-GM1b**) (700/175 MHz D_2O)

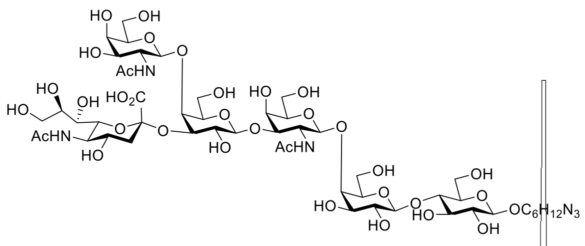

18

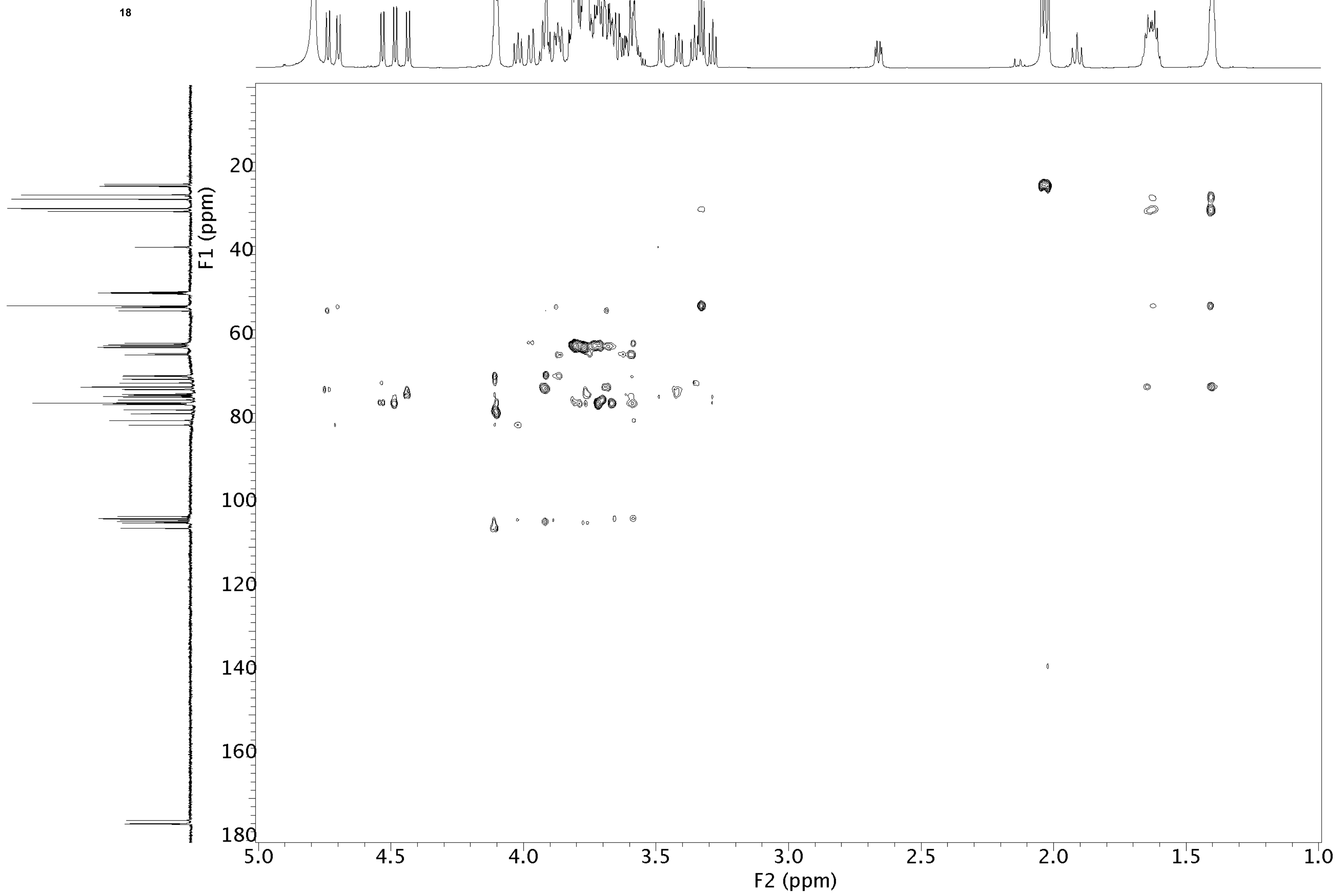

^{1}H-^{13}C HSQC-TOCSY spectrum of **18** (**GalNAc-GM1b**) (700/175 MHz D_2O)

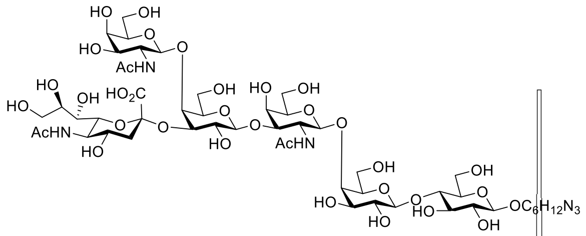

18

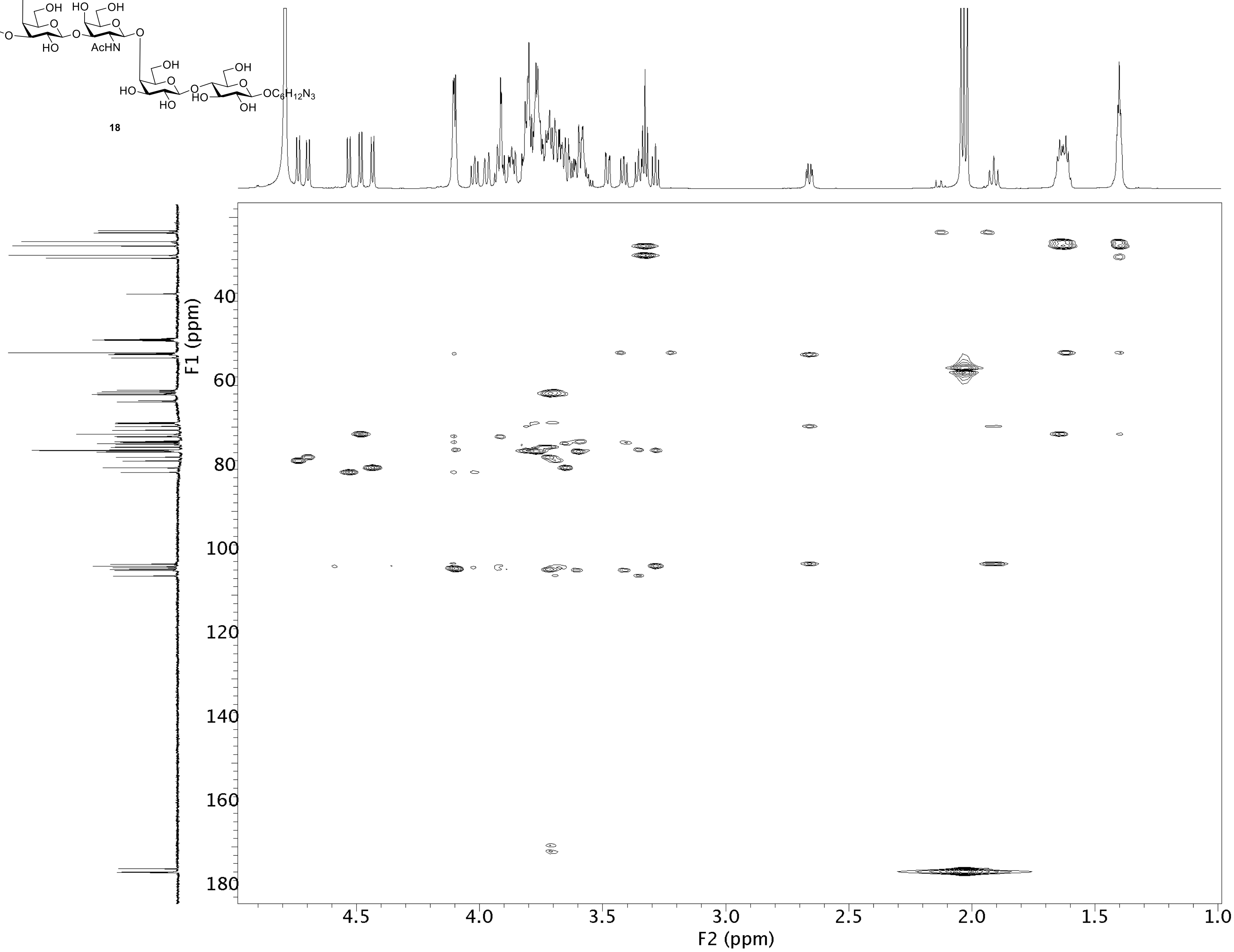

^{1}H-^{13}C HMBC spectrum of **18** (**GalNAc-GM1b**) (700/175 MHz D_2O)

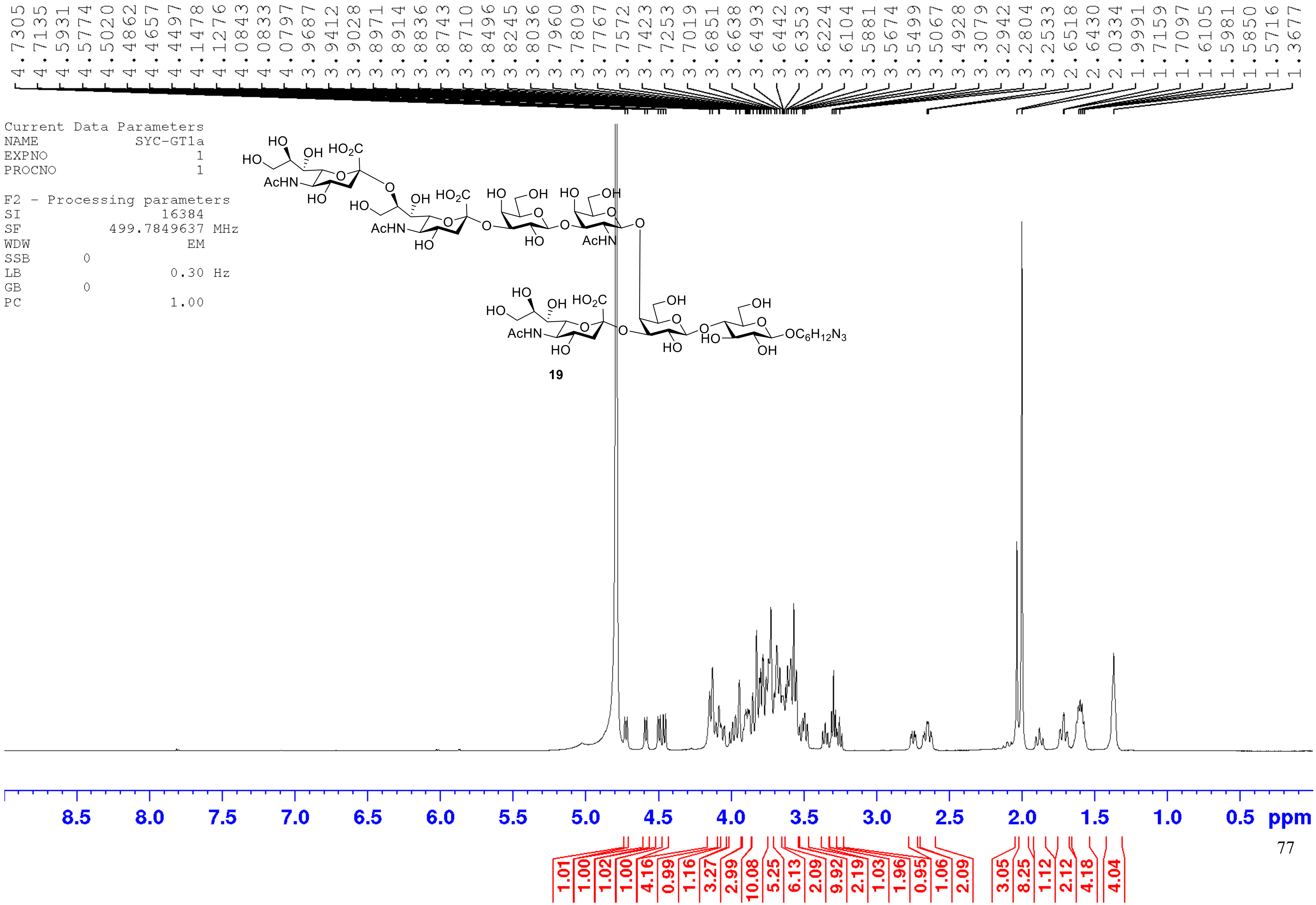

^{1}H NMR spectrum of **19** (**GT1a**) (500 MHz D_2O)

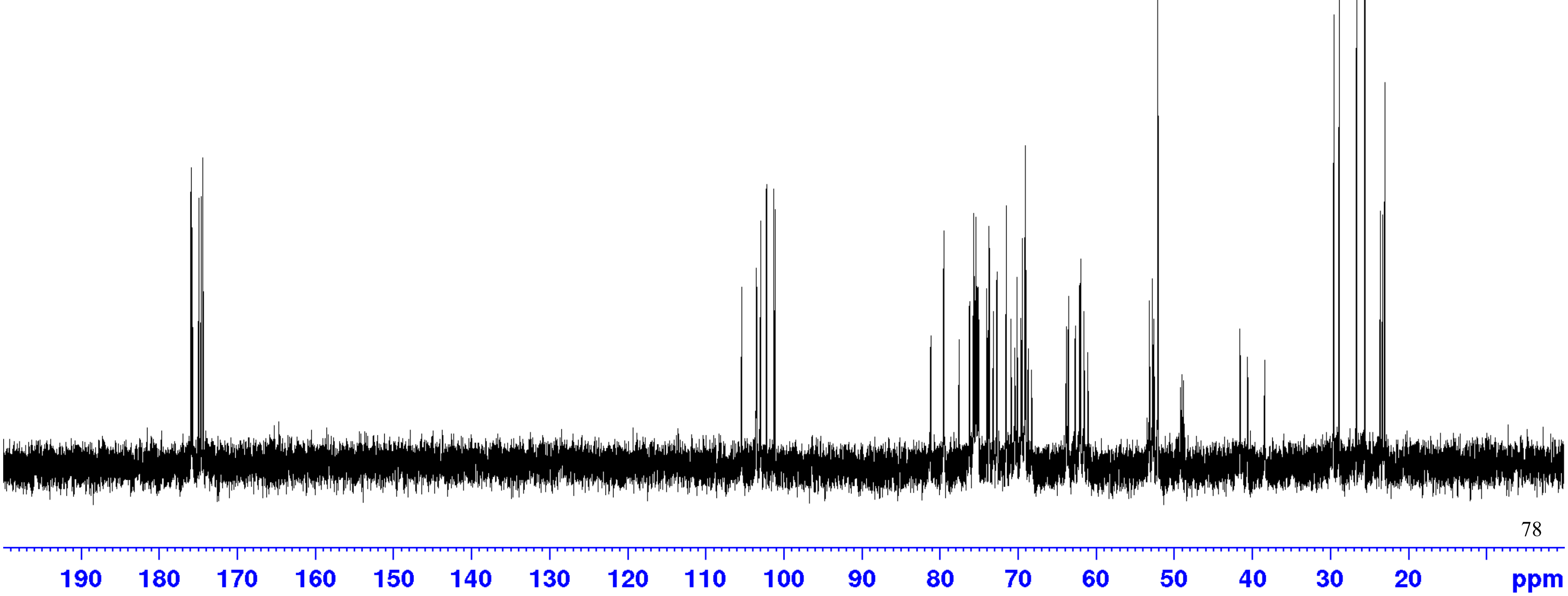


```
Current Data Parameters
NAME          SYC-GT1a-C
EXPNO                  1
PROCNO                 1

F2 - Processing parameters
SI                 65536
SF           125.6709135 MHz
WDW                   EM
SSB       0
LB                  0.30 Hz
GB        0
PC                  1.00
```

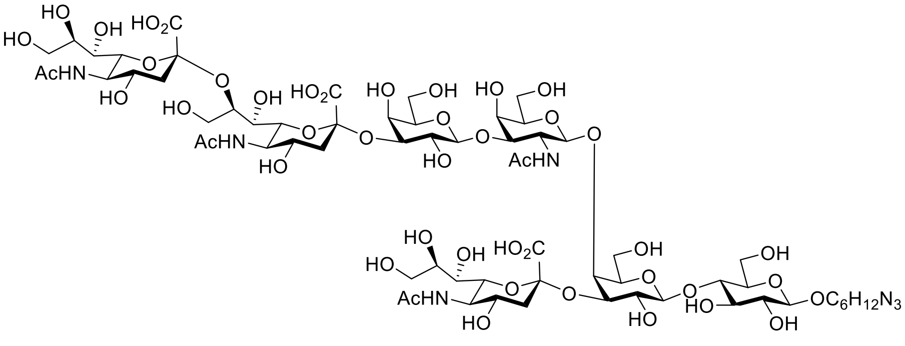

19

^{13}C NMR spectrum of **19** (**GT1a**) (125 MHz D_2O)

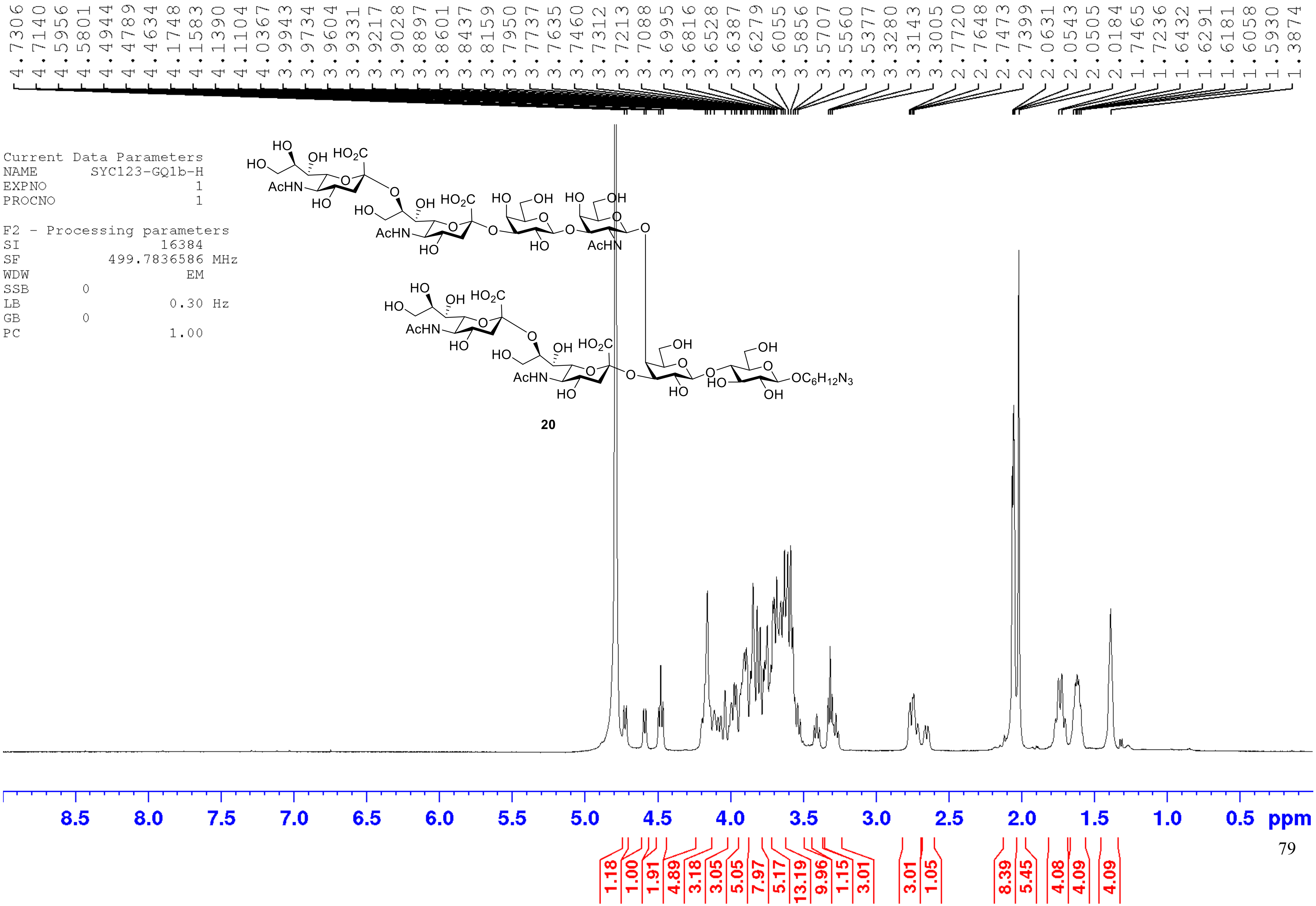

^{1}H NMR spectrum of **20** (**GQ1b**) (500 MHz D_2O)

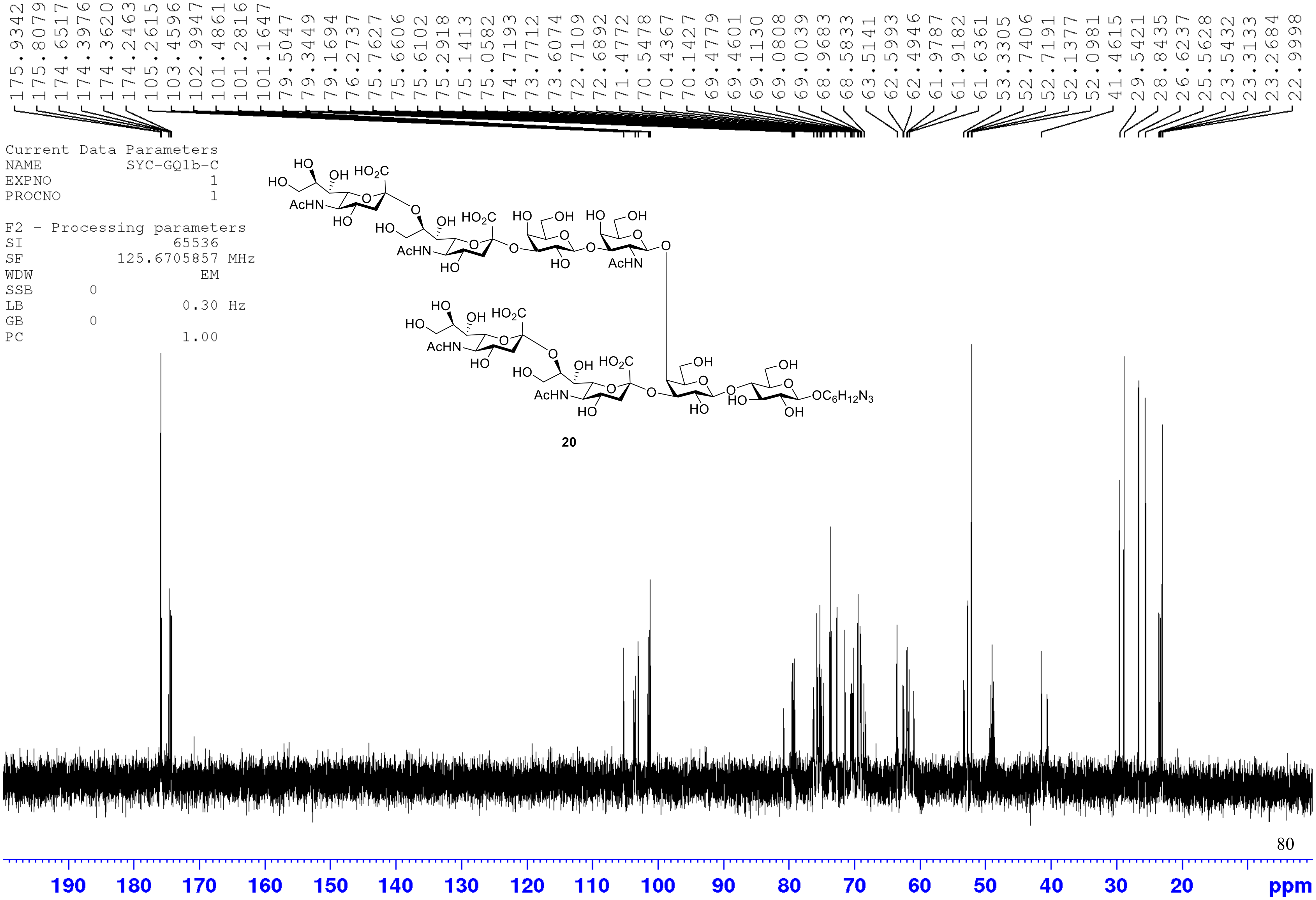

^{13}C NMR spectrum of **20** (**GQ1b**) (125 MHz D_2O)

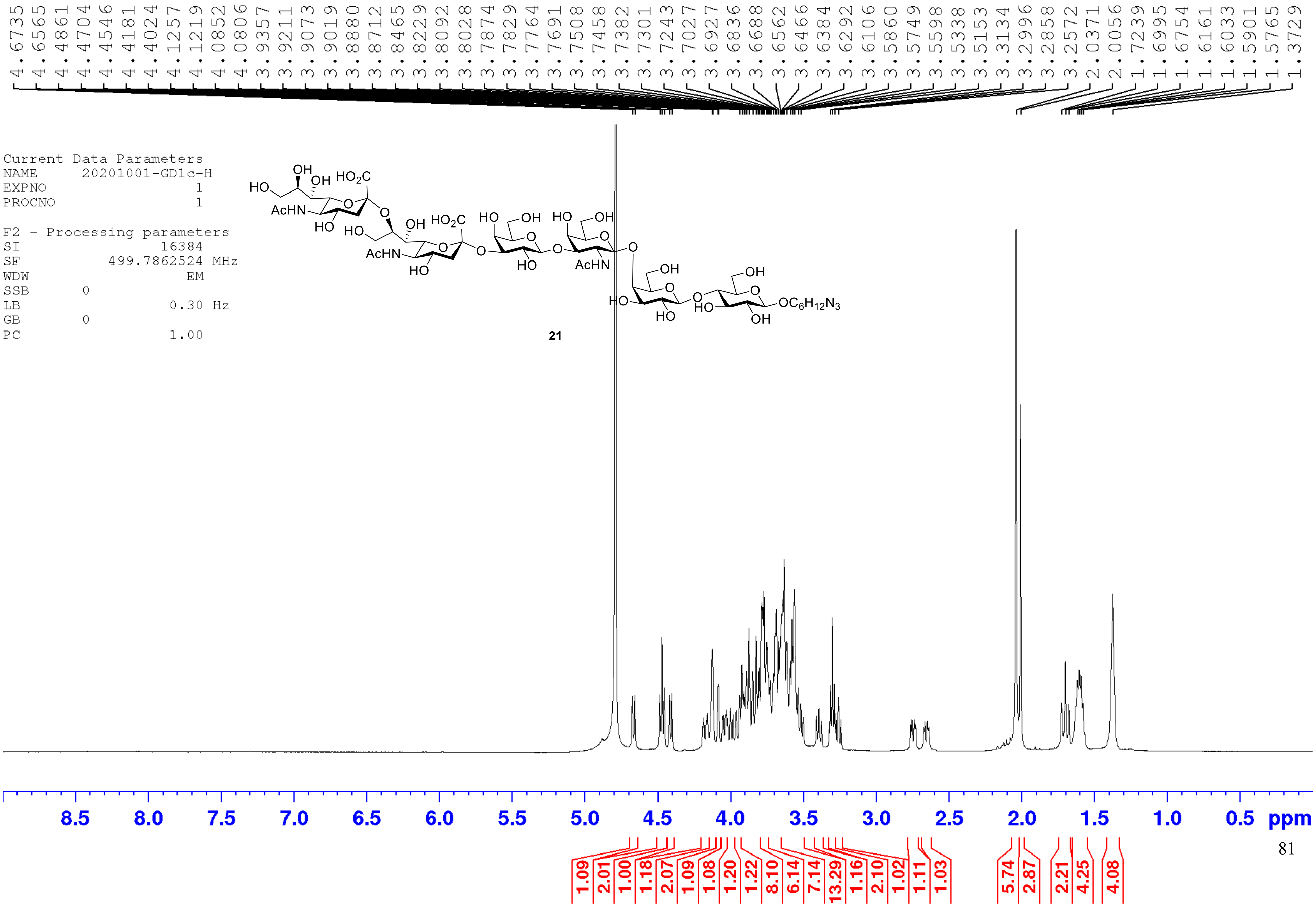

^{1}H NMR spectrum of **21** (**GD1c**) (500 MHz D$_2$O)

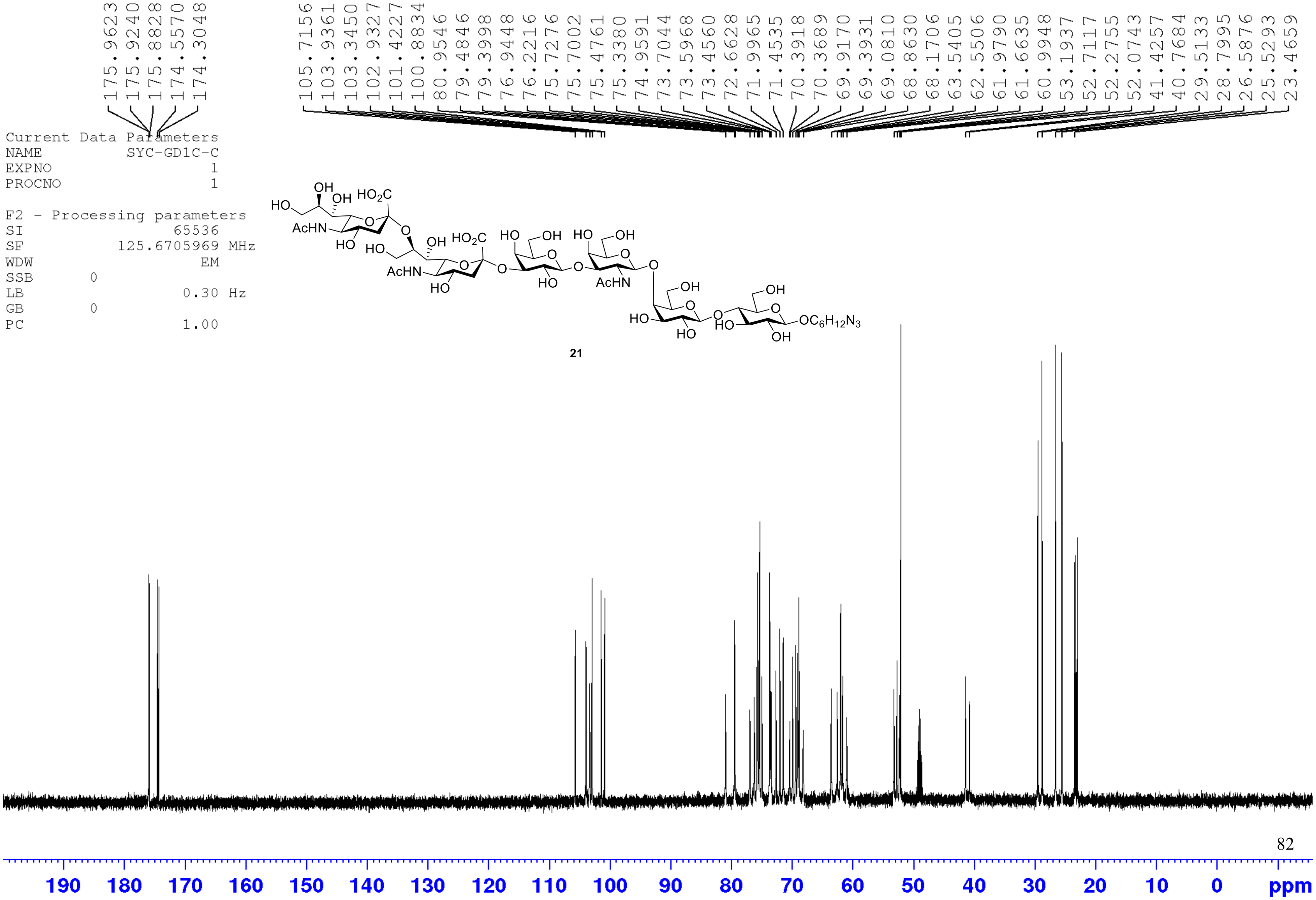

^{13}C NMR spectrum of **21** (**GD1c**) (125 MHz D_2O)

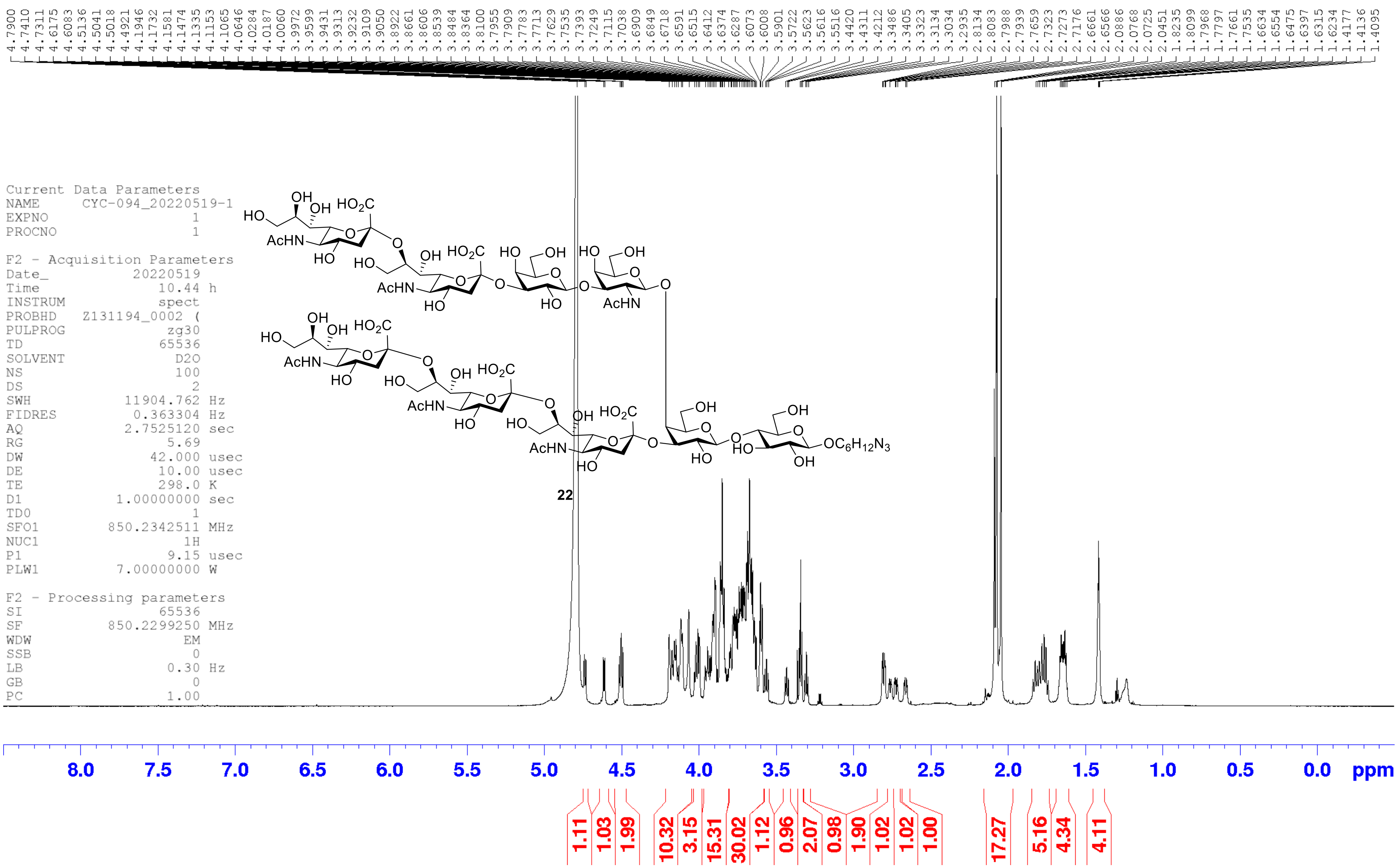

22

^{1}H NMR spectrum of **22** (**GP1c**) (850 MHz D_2O)

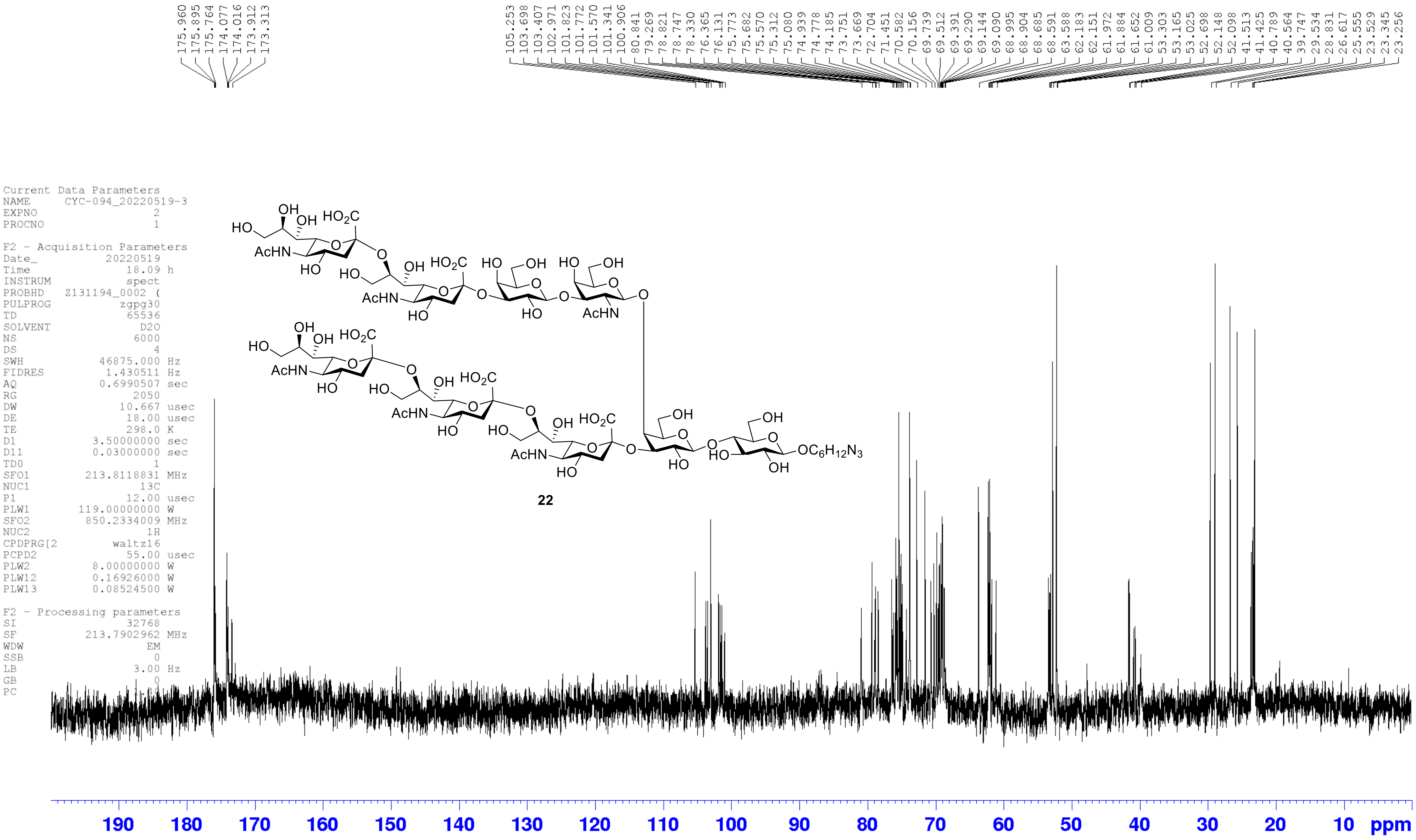

^{13}C NMR spectrum of **22** (**GP1c**) (214 MHz D_2O)

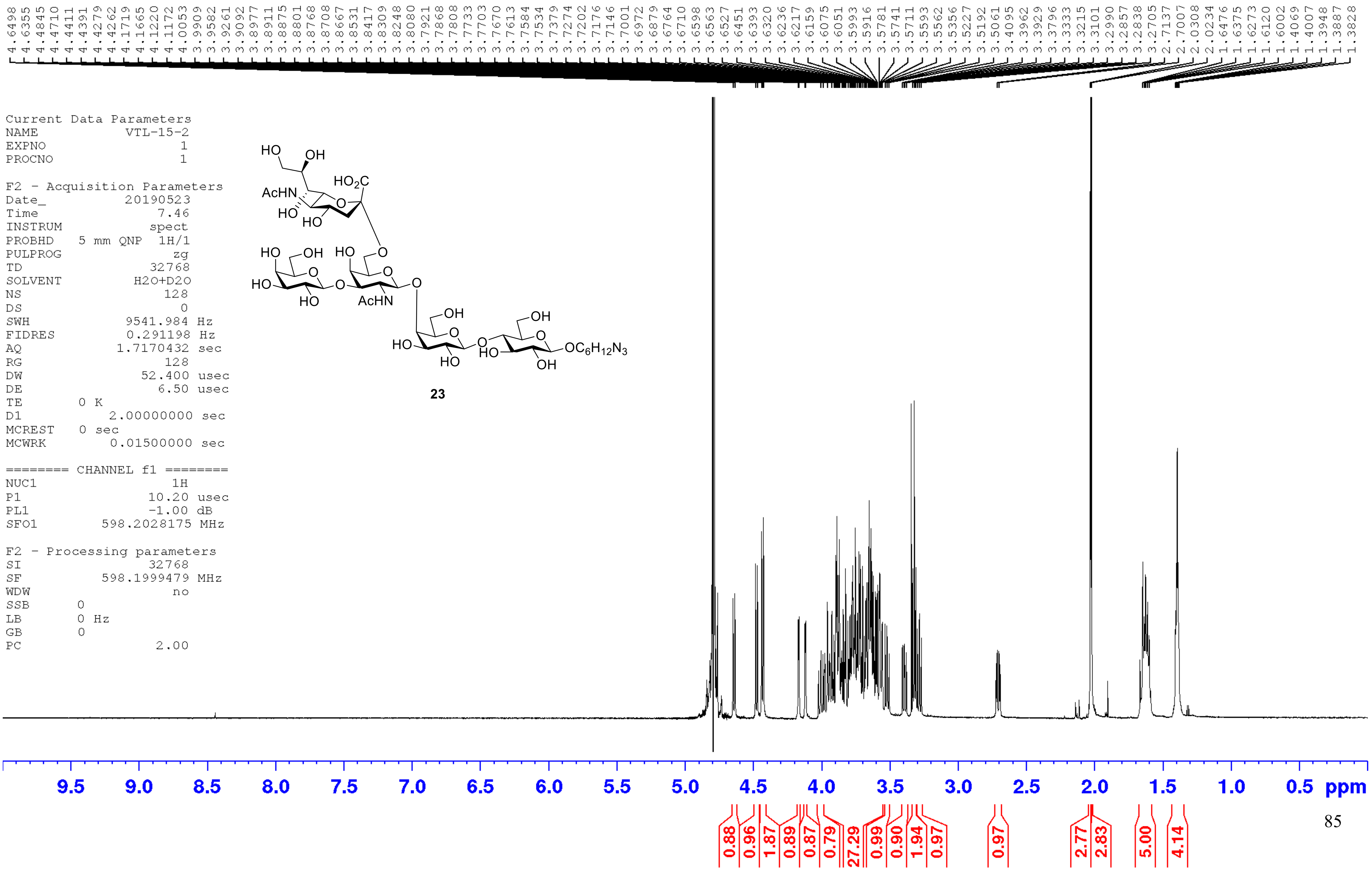

^{1}H NMR spectrum of **23** (**GM1α**) (600 MHz D_2O)

```
Current Data Parameters
NAME            VTL-15-2
EXPNO                  2
PROCNO                 1

F2 - Acquisition Parameters
Date_           20190525
Time               15.10
INSTRUM            spect
PROBHD   5 mm QNP  1H/1
PULPROG             zgpg
TD                 32768
SOLVENT          H2O+D2O
NS                  8259
DS                     0
SWH            45045.047 Hz
FIDRES          1.374666 Hz
AQ             0.3637248 sec
RG                  4096
DW                11.100 usec
DE                  6.50 usec
TE                 298.2 K
D1            3.50000000 sec
d11           0.03000000 sec
DELTA         3.40000010 sec
MCREST   0 sec
MCWRK         0.01500000 sec

======== CHANNEL f1 ========
NUC1                 13C
P1                  6.20 usec
PL1       0 dB
SFO1         150.4340559 MHz

======== CHANNEL f2 ========
CPDPRG[2         waltz16
NUC2                  1H
PCPD2              92.00 usec
PL2               120.00 dB
PL12               12.00 dB
PL13               15.00 dB
SFO2         598.2029910 MHz

F2 - Processing parameters
SI                 65536
SF           150.4173617 MHz
WDW                   EM
SSB      0
LB                  3.00 Hz
GB       0
PC                  1.00
```

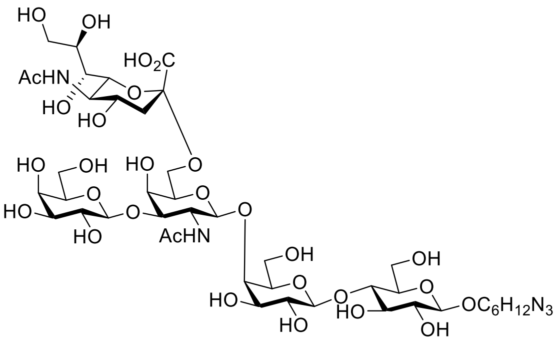

23

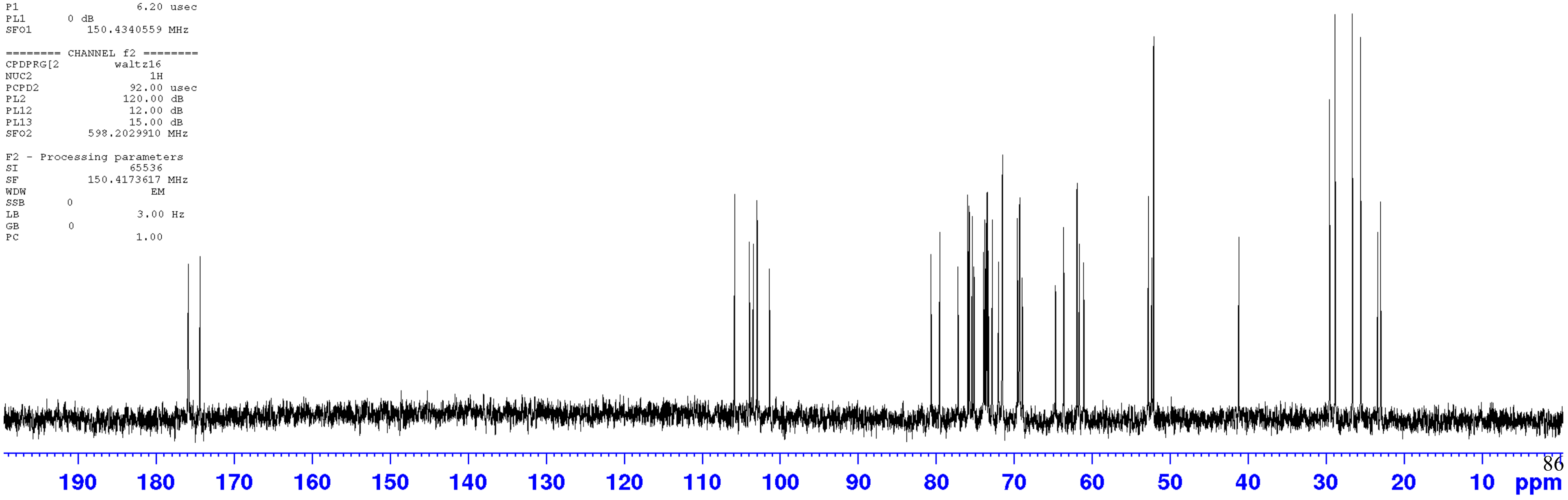

^{13}C NMR spectrum of **23** (**GM1α**) (150 MHz D_2O)

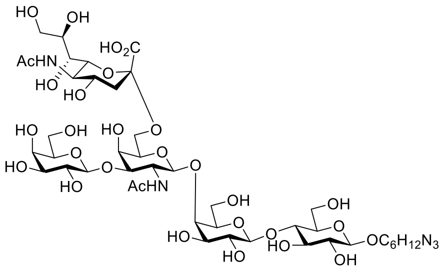

23

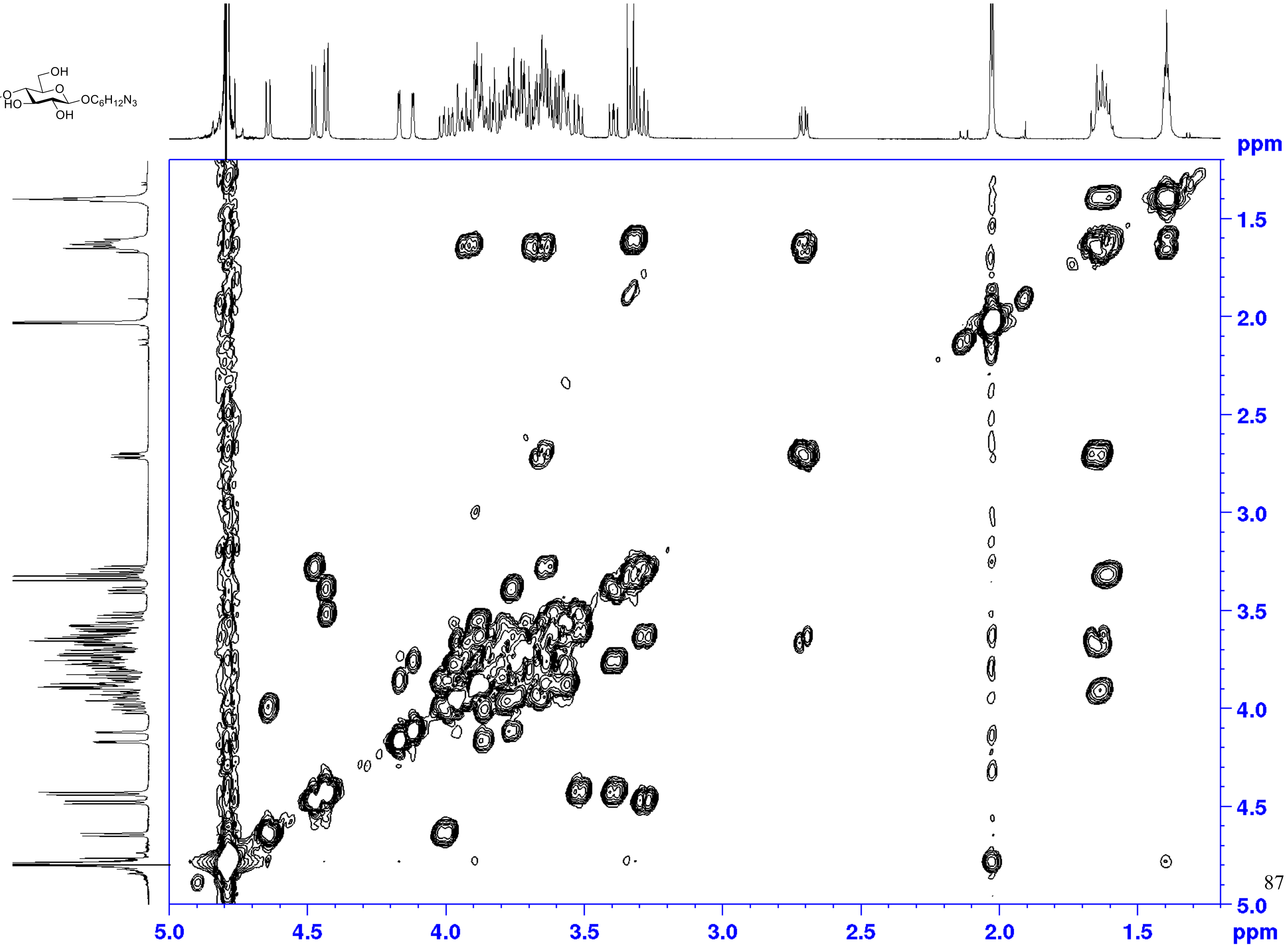


```
Current Data Parameters
NAME            VTL-16-2
EXPNO                 11
PROCNO                 1

F2 - Acquisition Parameters
Date_           20190523
Time                8.19
INSTRUM            spect
PROBHD   5 mm QNP  1H/1
PULPROG           cosygf
TD                  2048
SOLVENT             MeOD
NS                     8
DS                     0
SWH             6009.615 Hz
FIDRES          2.934382 Hz
AQ             0.1703936 sec
RG                   128
DW                83.200 usec
DE                  6.50 usec
TE         0 K
d0            0.00000300 sec
D1            1.50000000 sec
IN0           0.00016640 sec
MCREST   0 sec
MCWRK         1.50000000 sec

======== CHANNEL f1 ========
NUC1                  1H
P0                 14.00 usec
P1                 10.20 usec
PL1                -1.00 dB
SFO1         598.2028080 MHz

F1 - Acquisition parameters
TD                   360
SFO1            598.2028 MHz
FIDRES         33.386753 Hz
SW                10.046 ppm
FnMODE                QF

F2 - Processing parameters
SI                  2048
SF           598.1999420 MHz
WDW                QSINE
SSB        0
LB         0 Hz
GB         0
PC                  1.00

F1 - Processing parameters
SI                   512
MC2                   QF
SF           598.1999520 MHz
WDW                QSINE
SSB        0
LB         0 Hz
GB         0
```

^{1}H-^{1}H COSY spectrum of **23** (**GM1α**) (600 MHz D_2O)

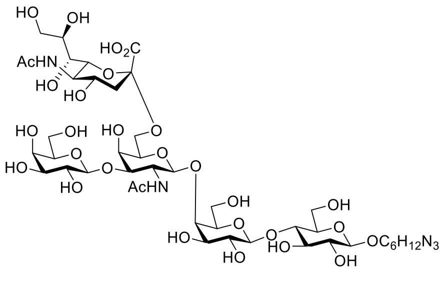

23

Current Data Parameters
NAME VTL-16-2
EXPNO 12
PROCNO 1

F2 - Acquisition Parameters
Date_ 20190523
Time 10.01
INSTRUM spect
PROBHD 5 mm QNP 1H/1
PULPROG hsqcetgpsi
TD 2048
SOLVENT H2O+D2O
NS 8
DS 8
SWH 6009.615 Hz
FIDRES 2.934382 Hz
AQ 0.1703936 sec
RG 32768
DW 83.200 usec
DE 6.50 usec
TE 0 K
CNST2 145.0000000
d0 0.00000300 sec
D1 1.20000005 sec
d4 0.00172414 sec
d11 0.03000000 sec
d13 0.00000400 sec
D16 0.00050000 sec
D24 0.00089000 sec
DELTA 0.00157640 sec
DELTA1 0.00150800 sec
IN0 0.00001279 sec
MCREST 0 sec
MCWRK 0.20000041 sec
ST1CNT 0

======== CHANNEL f1 ========
NUC1 1H
P1 10.20 usec
p2 20.40 usec
P28 1000.00 usec
PL1 -1.00 dB
SFO1 598.2028175 MHz

======== CHANNEL f2 ========
CPDPRG[2 garp
NUC2 13C
P3 10.00 usec
p4 20.00 usec
PCPD2 70.00 usec
PL2 0 dB
PL12 20.00 dB
SFO2 150.4287913 MHz

====== GRADIENT CHANNEL =====
GPNAM[1] SINE.100
GPNAM[2] SINE.100
GPX1 0 %
GPX2 0 %
GPY1 0 %
GPY2 0 %
GPZ1 80.00 %
GPZ2 20.10 %
P16 1000.00 usec

F1 - Acquisition parameters
TD 400
SFO1 150.4288 MHz
FIDRES 195.503418 Hz
SW 259.928 ppm
FnMODE Echo-Antiecho

F2 - Processing parameters
SI 2048
SF 598.1999600 MHz

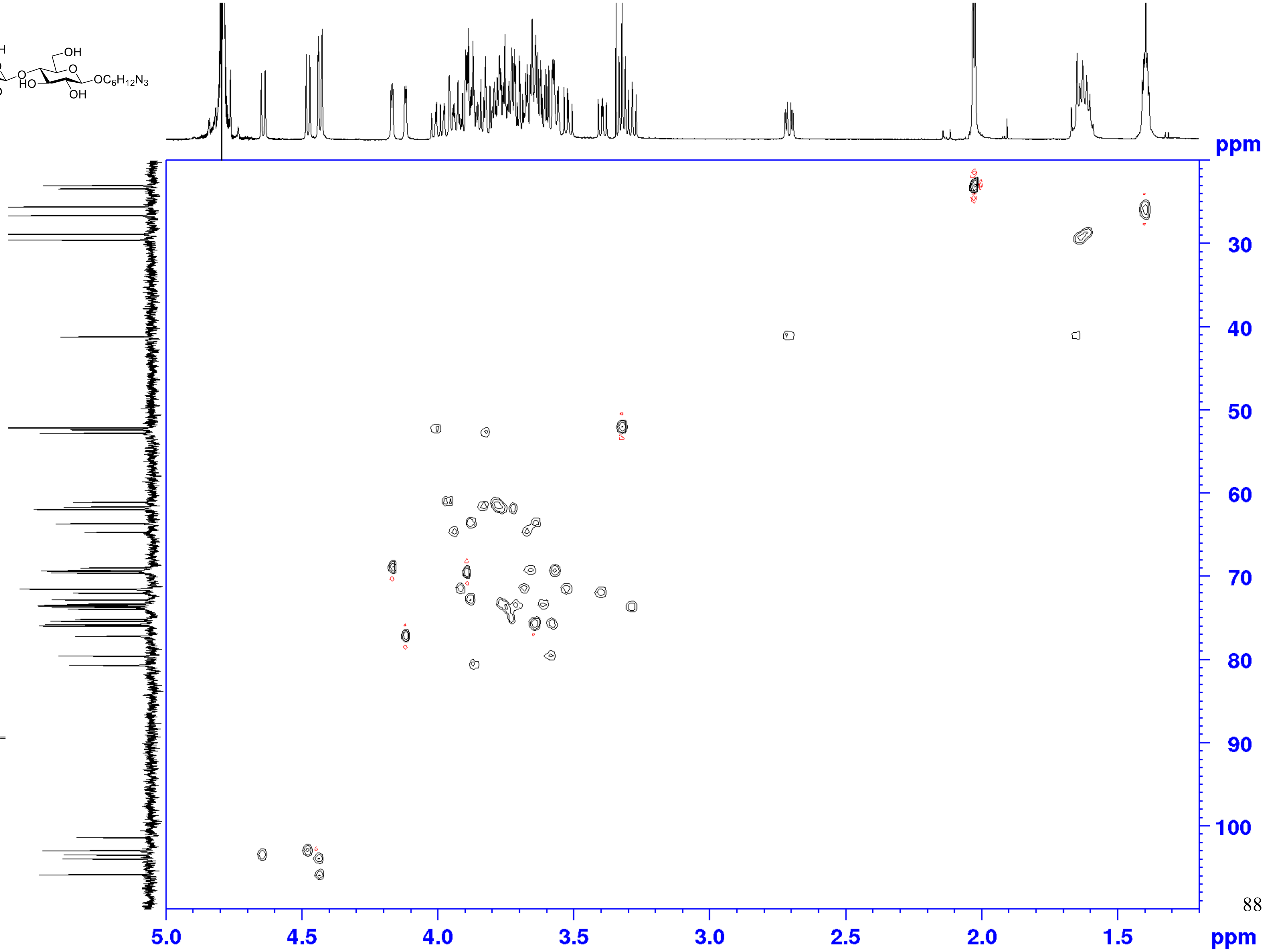

^{1}H-^{13}C HSQC spectrum of **23** (**GM1α**) (600/150 MHz D_2O)

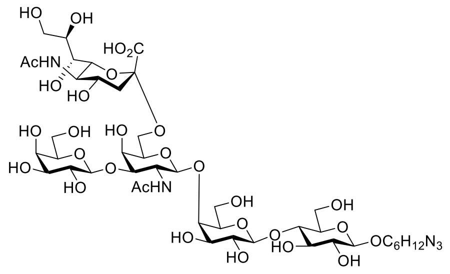

23

```
Current Data Parameters
NAME              VTL-16-2
EXPNO                   13
PROCNO                   1

F2 - Acquisition Parameters
Date_             20190524
Time                  5.08
INSTRUM              spect
PROBHD   5 mm QNP  1H/1
PULPROG     hmbcgplpndqf
TD                    2048
SOLVENT            H2O+D2O
NS                      32
DS                       0
SWH               6009.615 Hz
FIDRES            2.934382 Hz
AQ               0.1703936 sec
RG                   32768
DW                  83.200 usec
DE                    6.00 usec
TE              0 K
CNST2          145.0000000
CNST13           8.0000000
d0              0.00000300 sec
D1              1.20000005 sec
d2              0.00344828 sec
d6              0.06250000 sec
D16             0.00050000 sec
IN0             0.00001661 sec
MCREST   0 sec
MCWRK           1.20000005 sec

======== CHANNEL f1 ========
NUC1                    1H
P1                   10.20 usec
p2                   20.40 usec
PL1                  -1.00 dB
SFO1           598.2028175 MHz

======== CHANNEL f2 ========
NUC2                   13C
P3                   10.50 usec
PL2             0 dB
SFO2           150.4325518 MHz

====== GRADIENT CHANNEL =====
GPNAM[1]          SINE.100
GPNAM[2]          SINE.100
GPNAM[3]          SINE.100
GPX1            0 %
GPX2            0 %
GPX3            0 %
GPY1            0 %
GPY2            0 %
GPY3            0 %
GPZ1                 50.00 %
GPZ2                 30.00 %
GPZ3                 40.10 %
P16                1000.00 usec

F1 - Acquisition parameters
TD                     360
SFO1              150.4326 MHz
FIDRES          167.210098 Hz
SW                 200.075 ppm
FnMODE                  QF

F2 - Processing parameters
SI                    4096
SF             598.1999370 MHz
WDW                  QSINE
SSB                      2
LB              0 Hz
GB              0
PC                    1.00
```

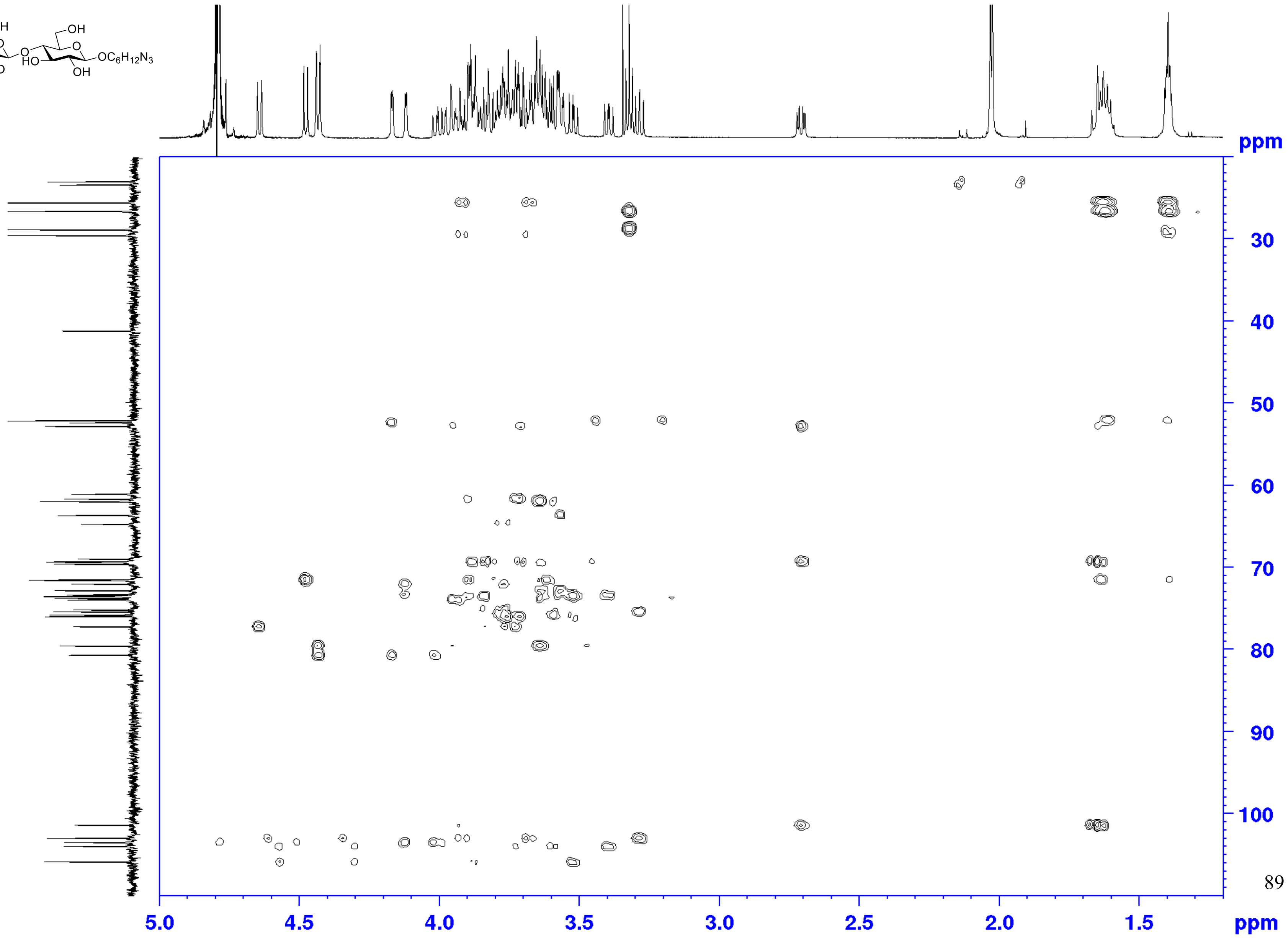

^{1}H-^{13}C HMBC spectrum of **23** (**GM1α**) (600/150 MHz D_2O)

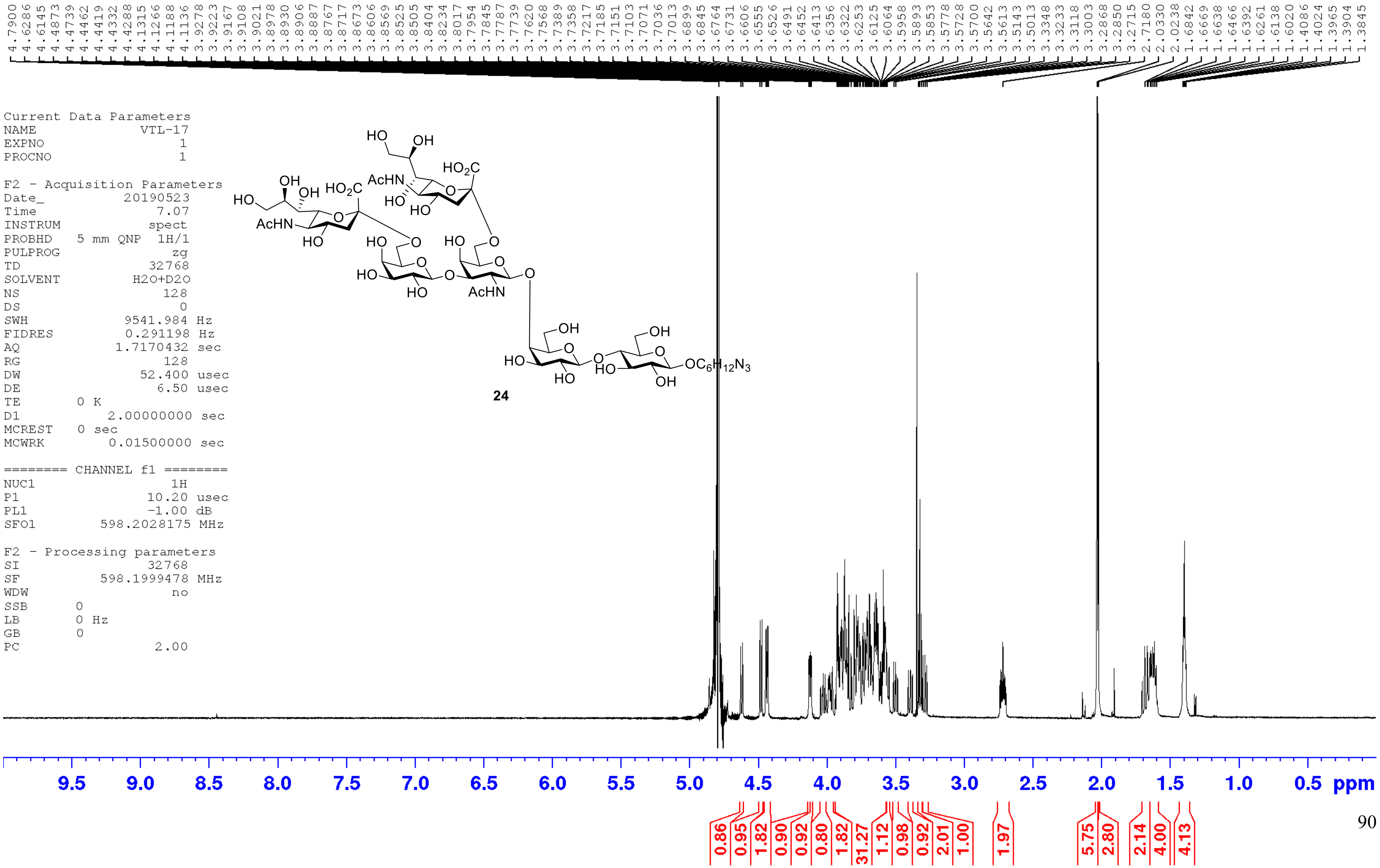

^{1}H NMR spectrum of **24** (**GM1α-S6**) (600 MHz D_2O)

```
Current Data Parameters
NAME     VTL217-di-sialyl-GA1-20190424-600
EXPNO                    2
PROCNO                   1

F2 - Acquisition Parameters
Date_           20190425
Time               12.17
INSTRUM            spect
PROBHD   5 mm QNP  1H/1
PULPROG             zgpg
TD                 32768
SOLVENT          H2O+D2O
NS                 14405
DS                     0
SWH            45045.047 Hz
FIDRES          1.374666 Hz
AQ             0.3637248 sec
RG                  4096
DW                11.100 usec
DE                  6.50 usec
TE         0 K
D1          3.50000000 sec
d11         0.03000000 sec
DELTA       3.40000010 sec
MCREST    0 sec
MCWRK       0.01500000 sec

======== CHANNEL f1 ========
NUC1                 13C
P1                  4.80 usec
PL1        0 dB
SFO1        150.4340559 MHz

======== CHANNEL f2 ========
CPDPRG[2         waltz16
NUC2                  1H
PCPD2              92.00 usec
PL2               120.00 dB
PL12                3.00 dB
PL13                5.00 dB
SFO2         598.2029910 MHz

F2 - Processing parameters
SI                 65536
SF           150.4173785 MHz
WDW                   EM
SSB        0
LB                  3.00 Hz
GB         0
PC                  1.00
```

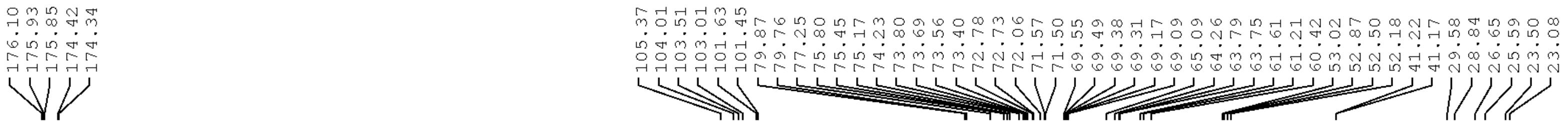

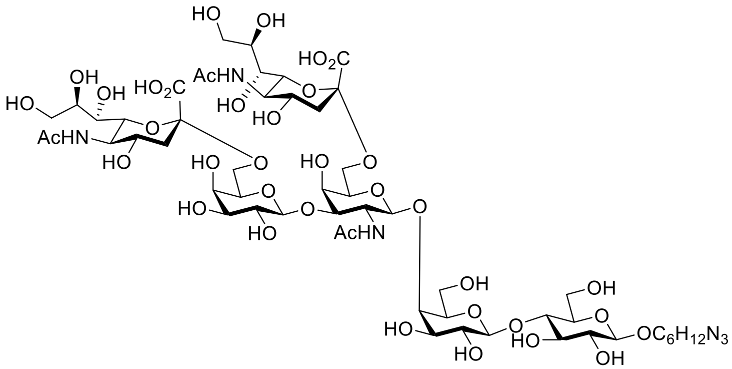

24

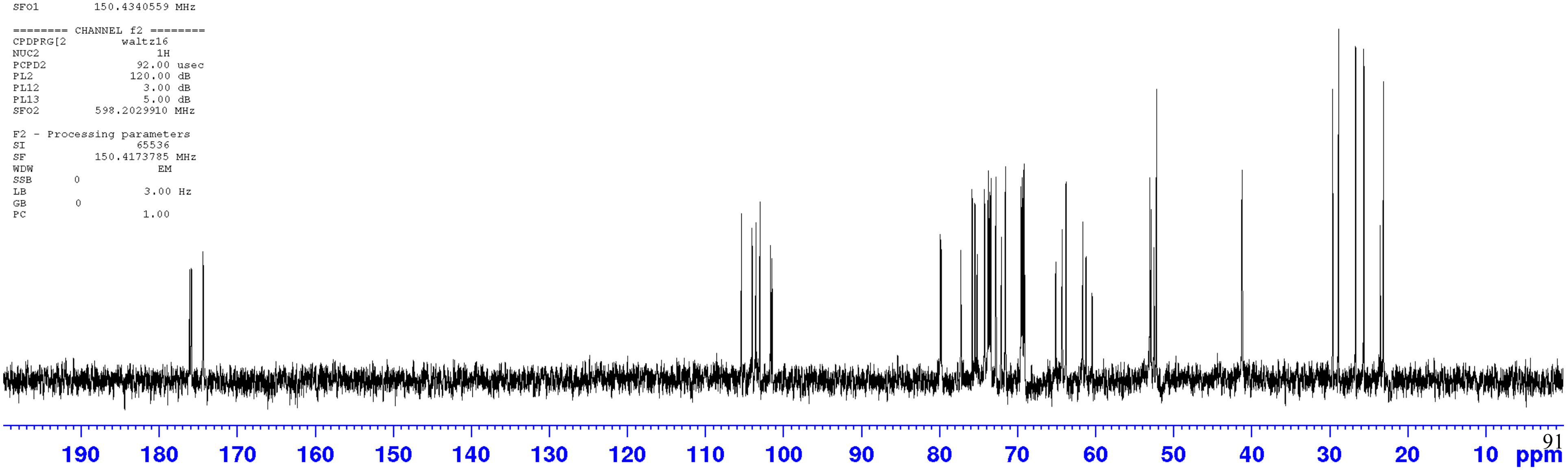

^{13}C NMR spectrum of **24** (**GM1α-S6**) (150 MHz D_2O)

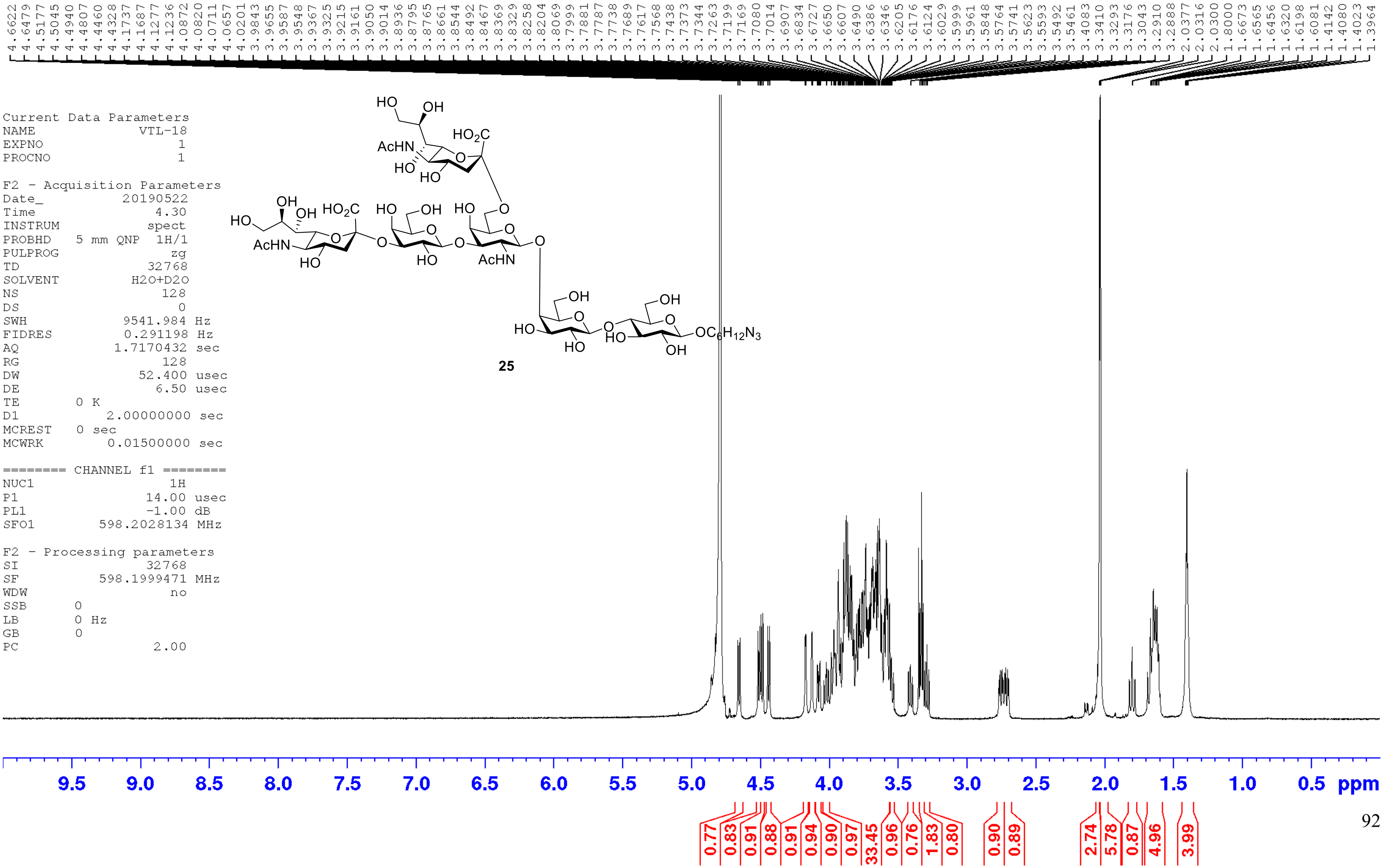

^{1}H NMR spectrum of **25** (**GD1aα**) (600 MHz D_2O)

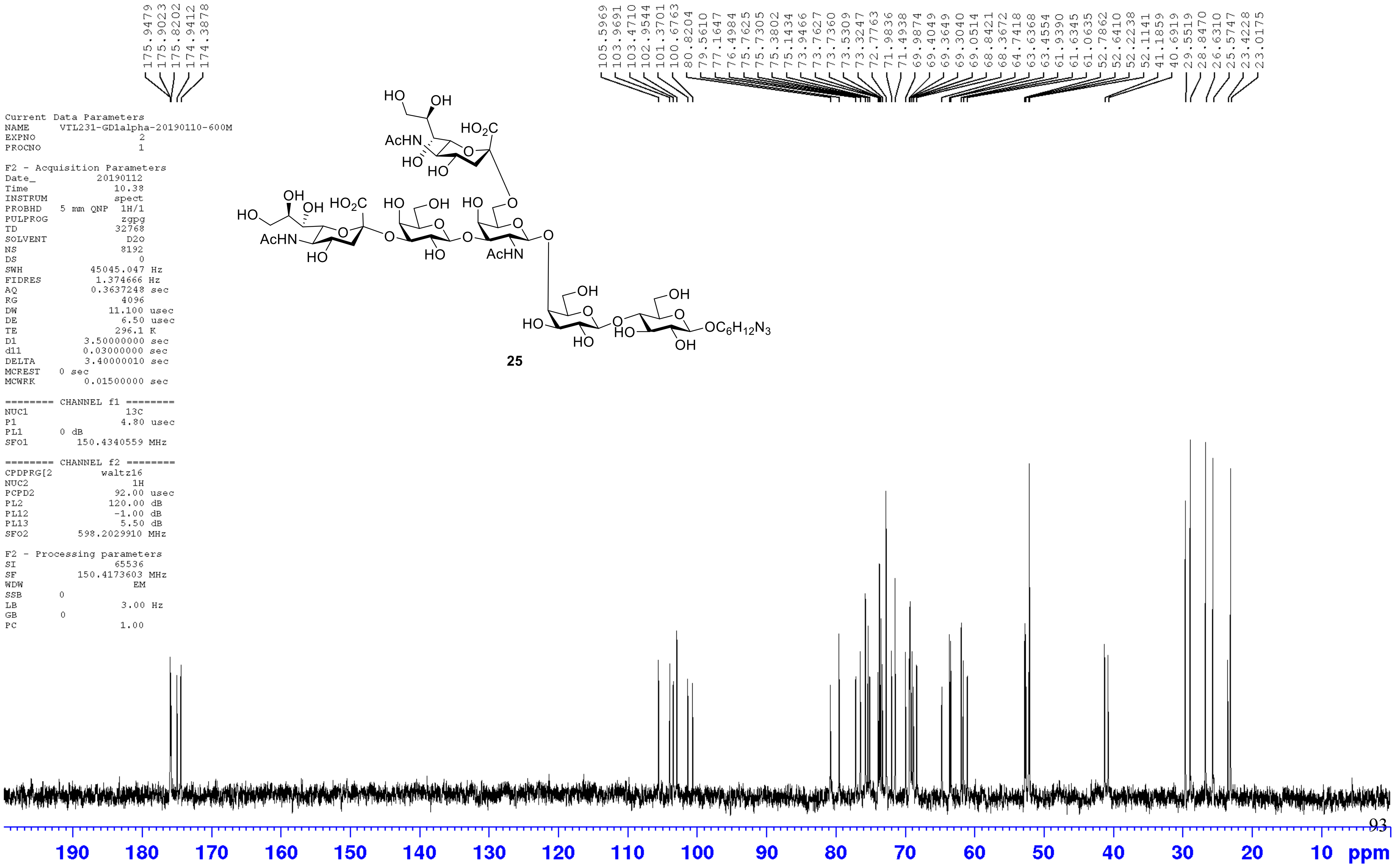

^{13}C NMR spectrum of **25** (**GD1aα**) (150 MHz D_2O)

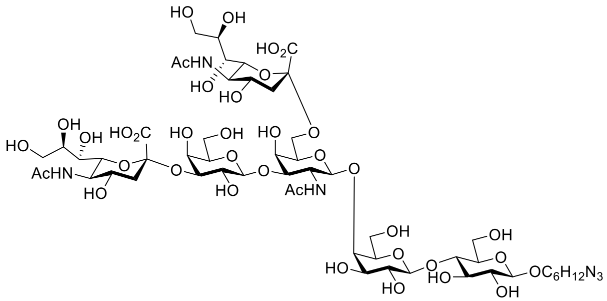

25

```
Current Data Parameters
NAME      VTL231-GD1alpha-20190110-600M
EXPNO                11
PROCNO                1

F2 - Acquisition Parameters
Date_          20190111
Time               5.45
INSTRUM           spect
PROBHD   5 mm QNP  1H/1
PULPROG          cosygf
TD                 2048
SOLVENT             D2O
NS                   16
DS                    0
SWH            6009.615 Hz
FIDRES         2.934382 Hz
AQ            0.1703936 sec
RG                  256
DW               83.200 usec
DE                 6.50 usec
TE                296.5 K
d0           0.00000300 sec
D1           1.50000000 sec
IN0          0.00016640 sec
MCREST   0 sec
MCWRK        1.50000000 sec

======== CHANNEL f1 ========
NUC1                 1H
P0                13.00 usec
P1                13.00 usec
PL1               -3.00 dB
SFO1        598.2028124 MHz

F1 - Acquisition parameters
TD                  400
SFO1           598.2028 MHz
FIDRES        30.048077 Hz
SW               10.046 ppm
FnMODE               QF

F2 - Processing parameters
SI                 2048
SF          598.1999468 MHz
WDW               QSINE
SSB      0
LB       0 Hz
GB       0
PC                 1.00

F1 - Processing parameters
SI                  512
MC2                  QF
SF          598.1999468 MHz
WDW               QSINE
SSB      0
LB       0 Hz
GB       0
```

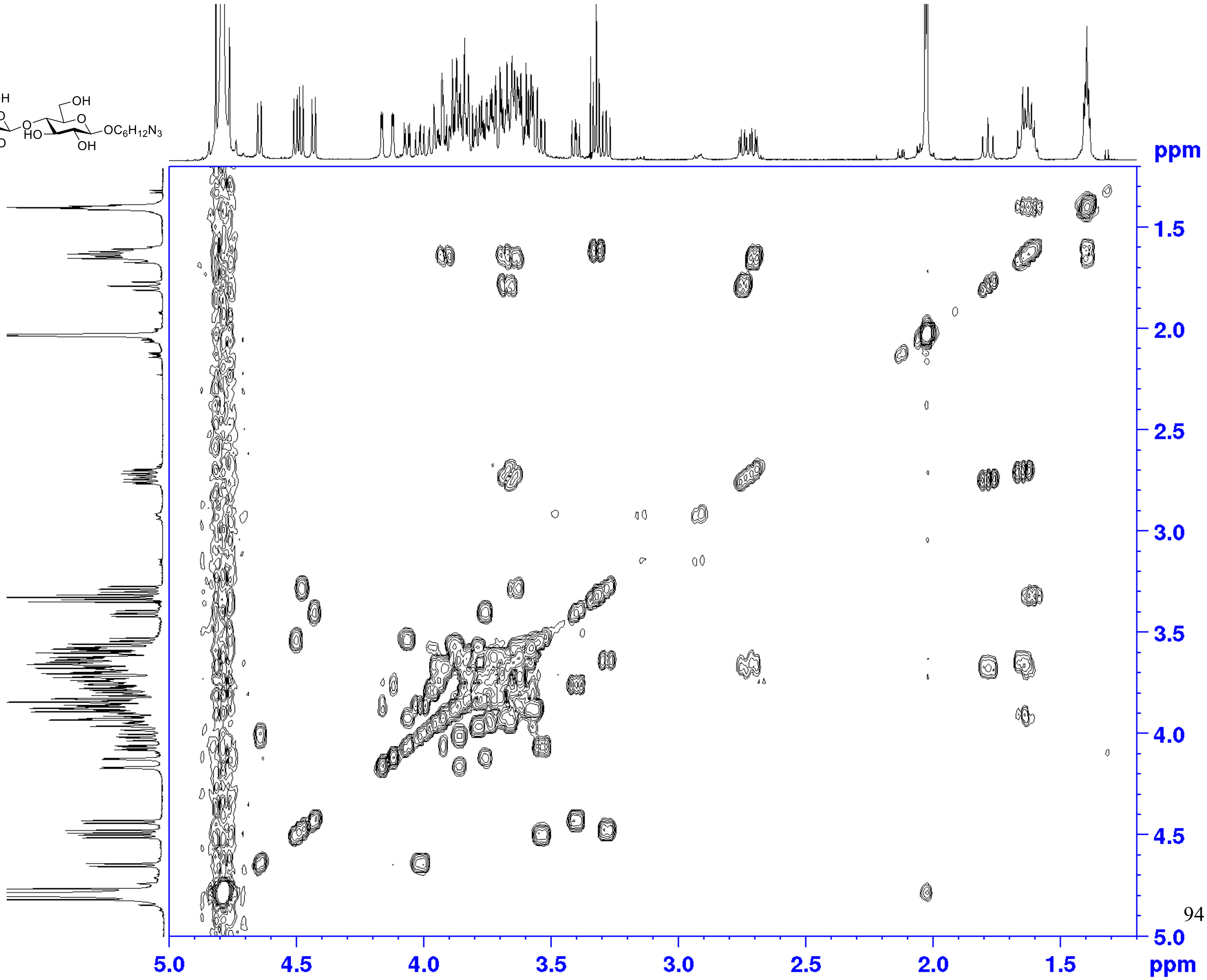

^{1}H-^{1}H COSY spectrum of **25** (**GD1aα**) (600 MHz D_2O)

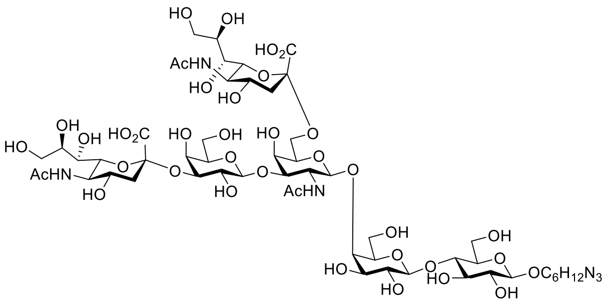

25

```
Current Data Parameters
NAME      VTL231-GD1alpha-20190110-600M
EXPNO                   12
PROCNO                   1

F2 - Acquisition Parameters
Date_            20190113
Time                12.48
INSTRUM             spect
PROBHD   5 mm QNP  1H/1
PULPROG       hsqcetgpsi
TD                   2048
SOLVENT               D2O
NS                     32
DS                      8
SWH              6009.615 Hz
FIDRES           2.934382 Hz
AQ              0.1703936 sec
RG                  32768
DW                 83.200 usec
DE                   6.50 usec
TE                  296.4 K
CNST2         145.0000000
d0             0.00000300 sec
D1             1.50000000 sec
d4             0.00172414 sec
d11            0.03000000 sec
d13            0.00000400 sec
D16            0.00050000 sec
D24            0.00089000 sec
DELTA          0.00158900 sec
DELTA1         0.00150800 sec
IN0            0.00001510 sec
MCREST   0 sec
MCWRK          0.25000051 sec
ST1CNT                  0

======== CHANNEL f1 ========
NUC1                   1H
P1                  16.50 usec
p2                  33.00 usec
P28               1000.00 usec
PL1                 -5.00 dB
SFO1          598.2028124 MHz

======== CHANNEL f2 ========
CPDPRG[2             garp
NUC2                  13C
P3                  10.00 usec
p4                  20.00 usec
PCPD2               70.00 usec
PL2      0 dB
PL12                20.00 dB
SFO2          150.4295434 MHz

====== GRADIENT CHANNEL =====
GPNAM[1]         SINE.100
GPNAM[2]         SINE.100
GPX1      0 %
GPX2      0 %
GPY1      0 %
GPY2      0 %
GPZ1                80.00 %
GPZ2                20.10 %
P16               1000.00 usec

F1 - Acquisition parameters
TD                    400
SFO1             150.4295 MHz
FIDRES         165.562912 Hz
SW                220.120 ppm
FnMODE       Echo-Antiecho

F2 - Processing parameters
SI                   2048
SF            598.1999355 MHz
```

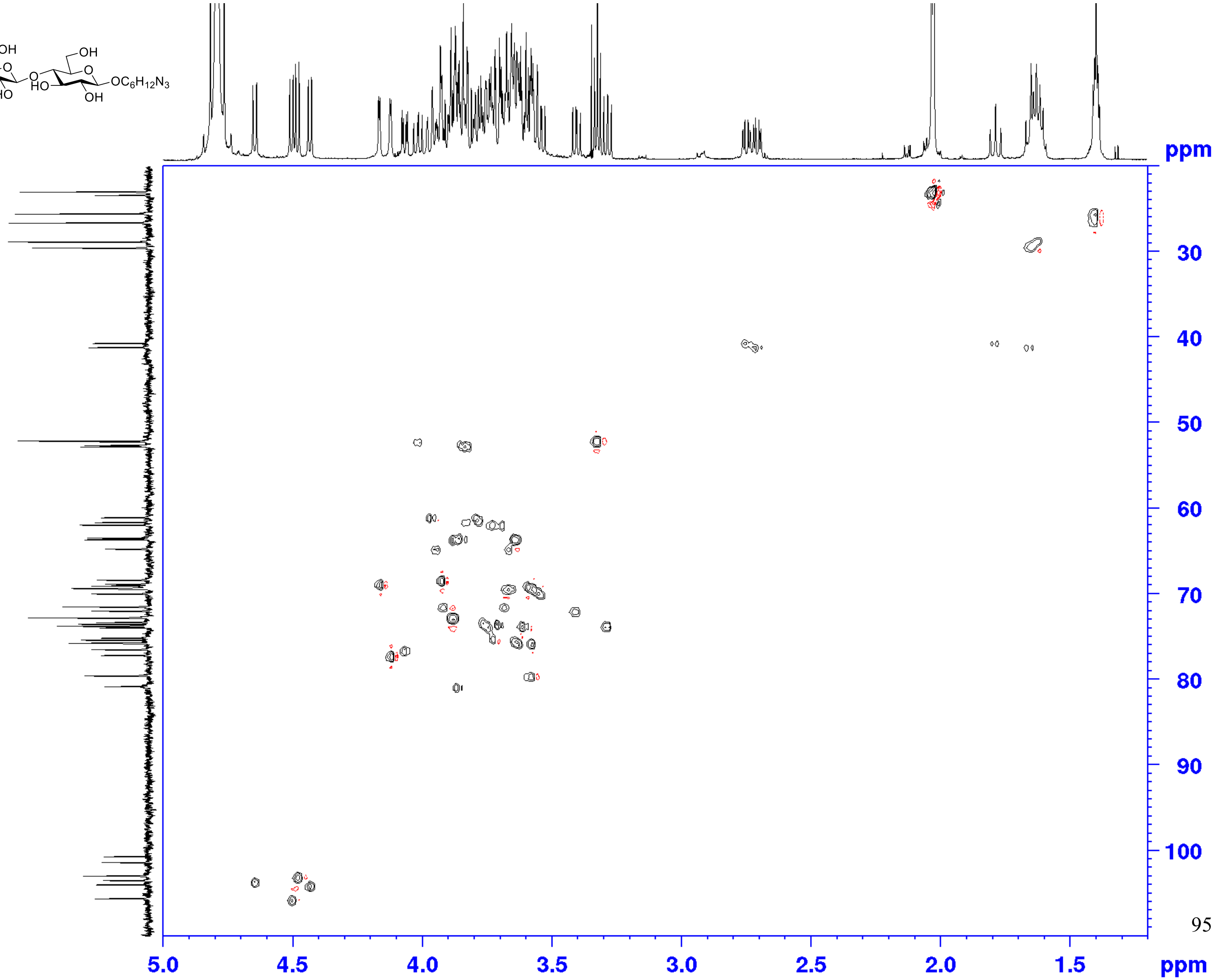

^{1}H-^{13}C HSQC spectrum of **25** (**GD1aα**) (600/150 MHz D_2O)

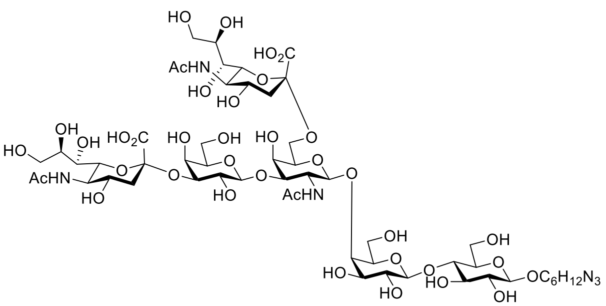

25

```
Current Data Parameters
NAME     VTL231-GD1alpha-20190110-600M
EXPNO                  13
PROCNO                  1

F2 - Acquisition Parameters
Date_           20190113
Time               18.50
INSTRUM            spect
PROBHD   5 mm QNP  1H/1
PULPROG     hmbcgplpndqf
TD                  2048
SOLVENT              D2O
NS                    80
DS                     0
SWH             6009.615 Hz
FIDRES          2.934382 Hz
AQ             0.1703936 sec
RG                 32768
DW                83.200 usec
DE                  6.00 usec
TE                 296.3 K
CNST2        145.0000000
CNST13        12.0000000
d0            0.00000300 sec
D1            1.20000005 sec
d2            0.00344828 sec
d6            0.04166667 sec
D16           0.00050000 sec
IN0           0.00001661 sec
MCREST   0 sec
MCWRK         1.20000005 sec

======== CHANNEL f1 ========
NUC1                  1H
P1                 16.50 usec
p2                 33.00 usec
PL1                -5.00 dB
SFO1         598.2028124 MHz

======== CHANNEL f2 ========
NUC2                 13C
P3                 12.00 usec
PL2       0 dB
SFO2         150.4310476 MHz

====== GRADIENT CHANNEL =====
GPNAM[1]        SINE.100
GPNAM[2]        SINE.100
GPNAM[3]        SINE.100
GPX1      0 %
GPX2      0 %
GPX3      0 %
GPY1      0 %
GPY2      0 %
GPY3      0 %
GPZ1               50.00 %
GPZ2               30.00 %
GPZ3               40.10 %
P16              1000.00 usec

F1 - Acquisition parameters
TD                   342
SFO1             150.431 MHz
FIDRES        176.010635 Hz
SW               200.077 ppm
FnMODE                QF

F2 - Processing parameters
SI                  4096
SF           598.1999100 MHz
WDW                QSINE
SSB                    2
LB        0 Hz
GB        0
PC                  1.00
```

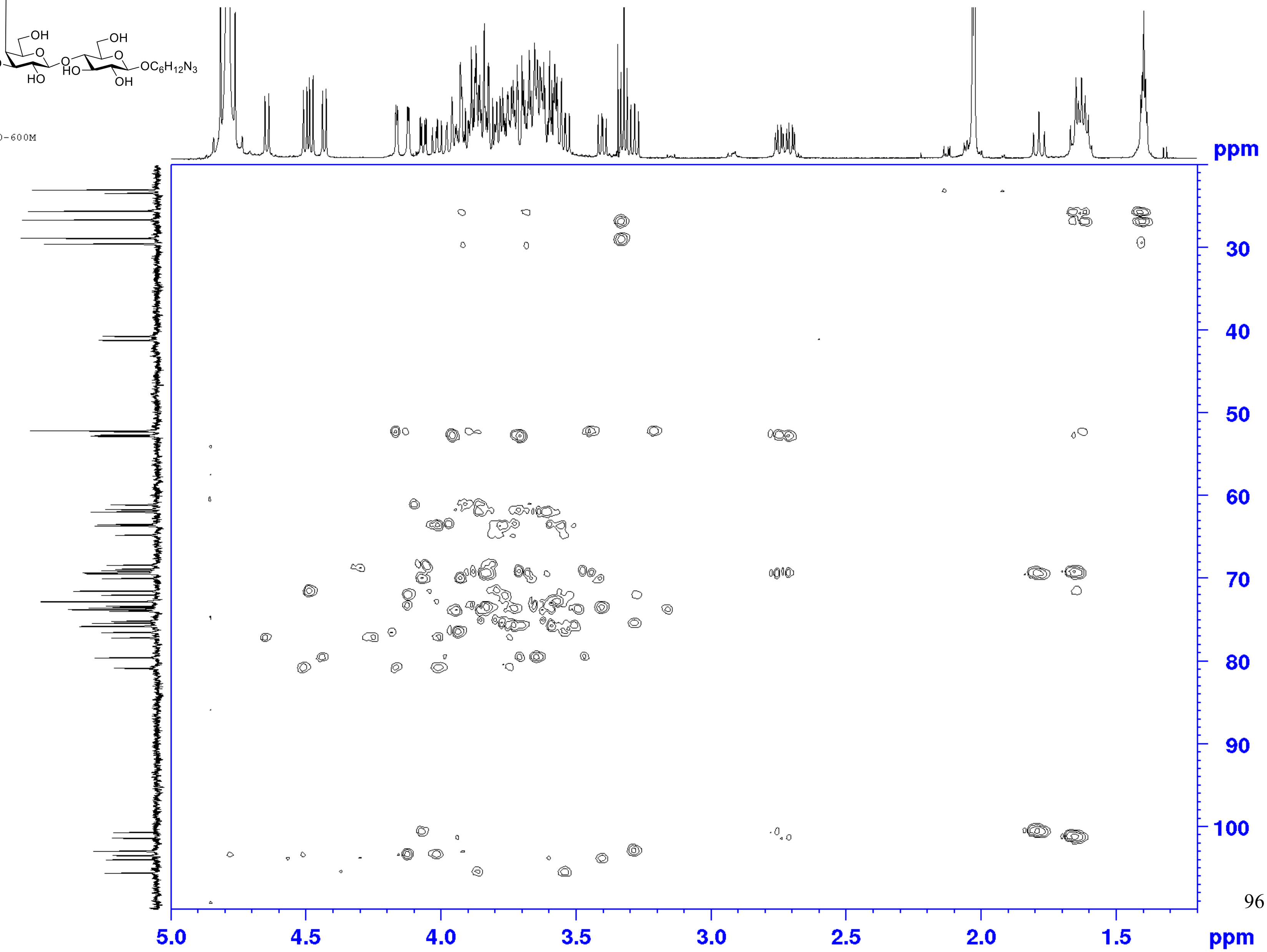

^{1}H-^{13}C HMBC spectrum of **25** (**GD1aα**) (600/150 MHz D_2O)

Current Data Parameters
NAME VTL-268-GD1aalpha
EXPNO 1
PROCNO 1

F2 - Acquisition Parameters
Date_ 20190509
Time 9.41
INSTRUM spect
PROBHD 5 mm QNP 1H/1
PULPROG zg
TD 32768
SOLVENT H2O+D2O
NS 128
DS 0
SWH 9541.984 Hz
FIDRES 0.291198 Hz
AQ 1.7170432 sec
RG 512
DW 52.400 usec
DE 6.50 usec
TE 296.6 K
D1 2.00000000 sec
MCREST 0 sec
MCWRK 0.01500000 sec

======== CHANNEL f1 ========
NUC1 1H
P1 10.00 usec
PL1 -2.00 dB
SFO1 598.2028187 MHz

F2 - Processing parameters
SI 32768
SF 598.1999476 MHz
WDW no
SSB 0
LB 0 Hz
GB 0
PC 2.00

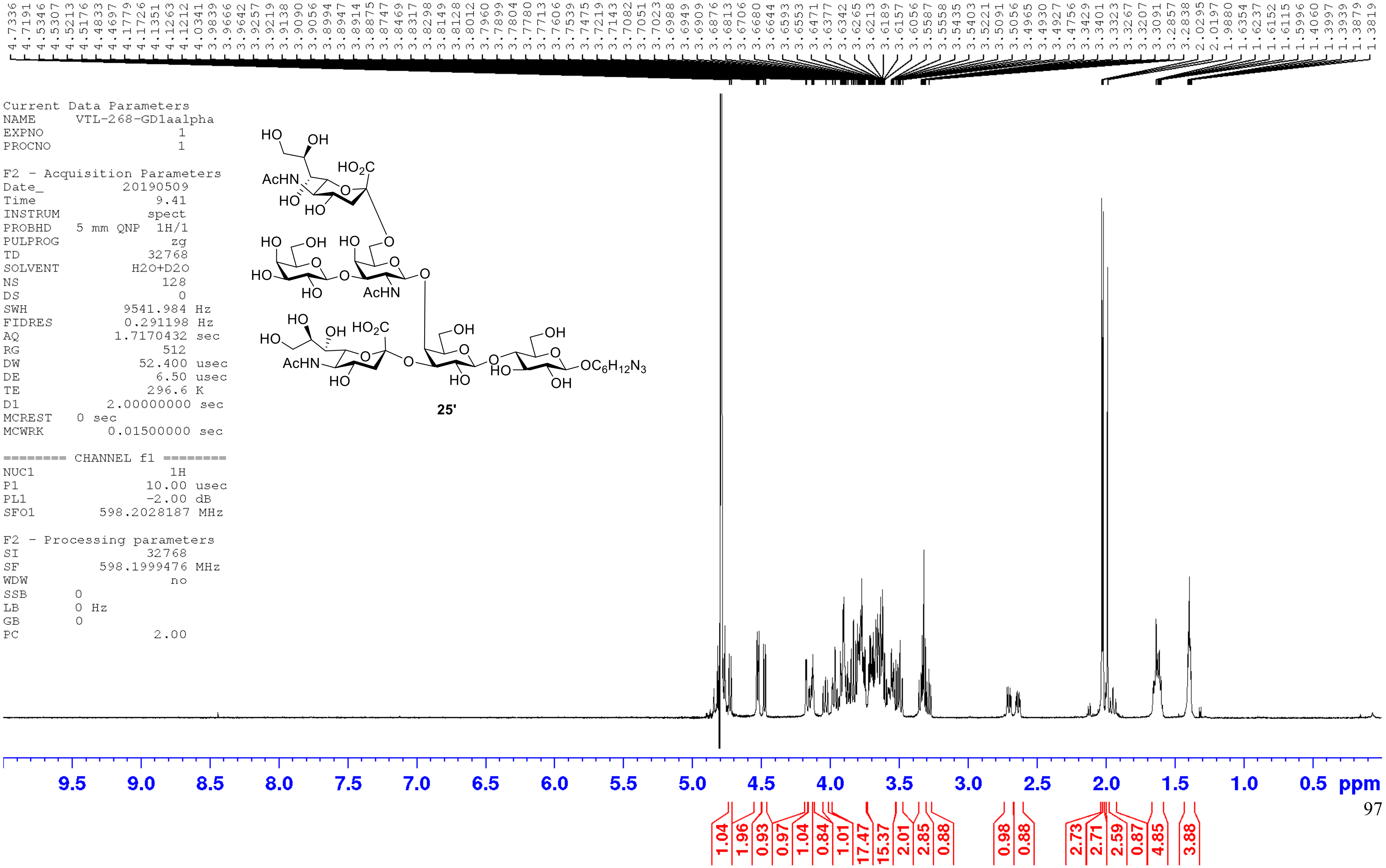

25'

^{1}H NMR spectrum of **25’** (**GD1aα’**) (600 MHz D_2O)

```
Current Data Parameters
NAME      VTL-sia26-GM1-20190423-600M
EXPNO                 2
PROCNO                1

F2 - Acquisition Parameters
Date_          20190424
Time               7.49
INSTRUM           spect
PROBHD   5 mm QNP  1H/1
PULPROG            zgpg
TD                32768
SOLVENT         H2O+D2O
NS                17785
DS                    0
SWH           45045.047 Hz
FIDRES         1.374666 Hz
AQ            0.3637248 sec
RG                 4096
DW               11.100 usec
DE                 6.50 usec
TE         0 K
D1           3.50000000 sec
d11          0.03000000 sec
DELTA        3.40000010 sec
MCREST   0 sec
MCWRK        0.01500000 sec

======== CHANNEL f1 ========
NUC1                13C
P1                 4.80 usec
PL1        0 dB
SFO1        150.4340559 MHz

======== CHANNEL f2 ========
CPDPRG[2          waltz16
NUC2                 1H
PCPD2             92.00 usec
PL2              120.00 dB
PL12               3.00 dB
PL13               5.00 dB
SFO2        598.2029910 MHz

F2 - Processing parameters
SI                65536
SF          150.4173754 MHz
WDW                  EM
SSB        0
LB                 3.00 Hz
GB         0
PC                 1.00
```

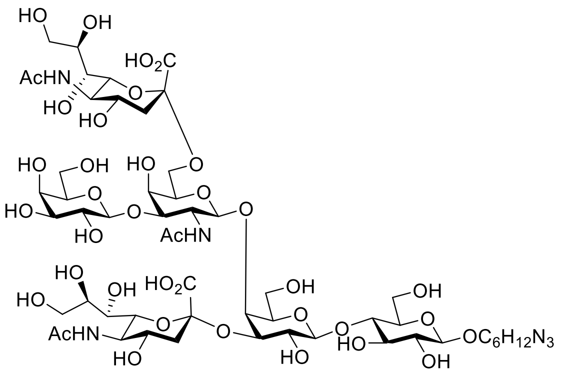

25'

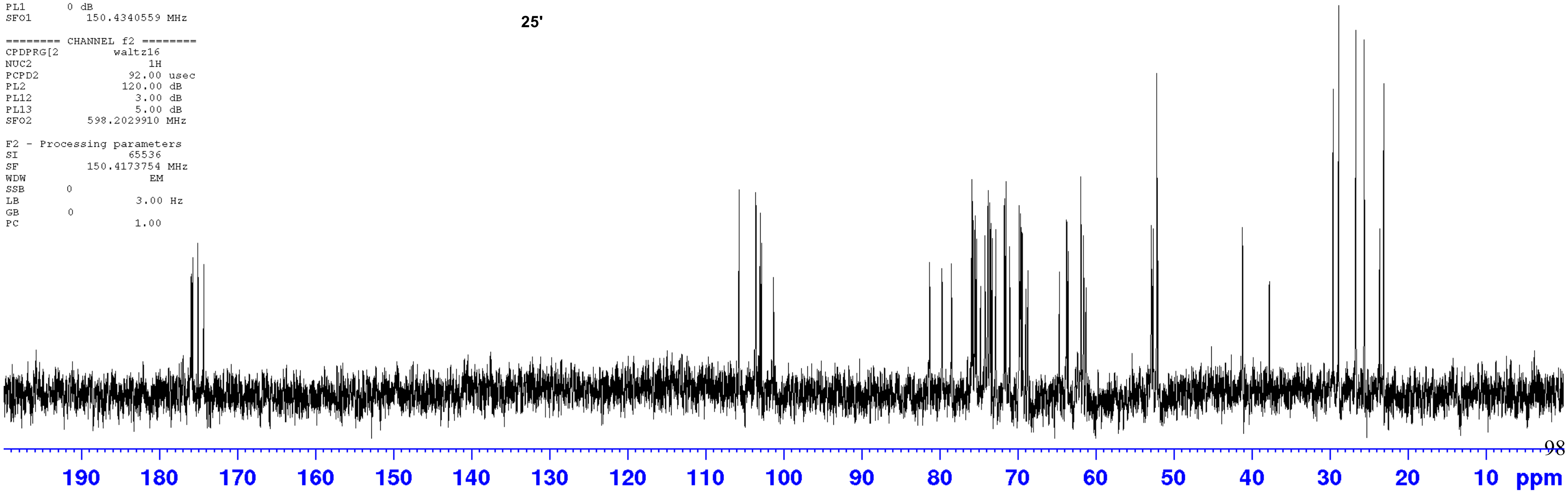

^{13}C NMR spectrum of **25'** (**GD1aα'**) (150 MHz D_2O)

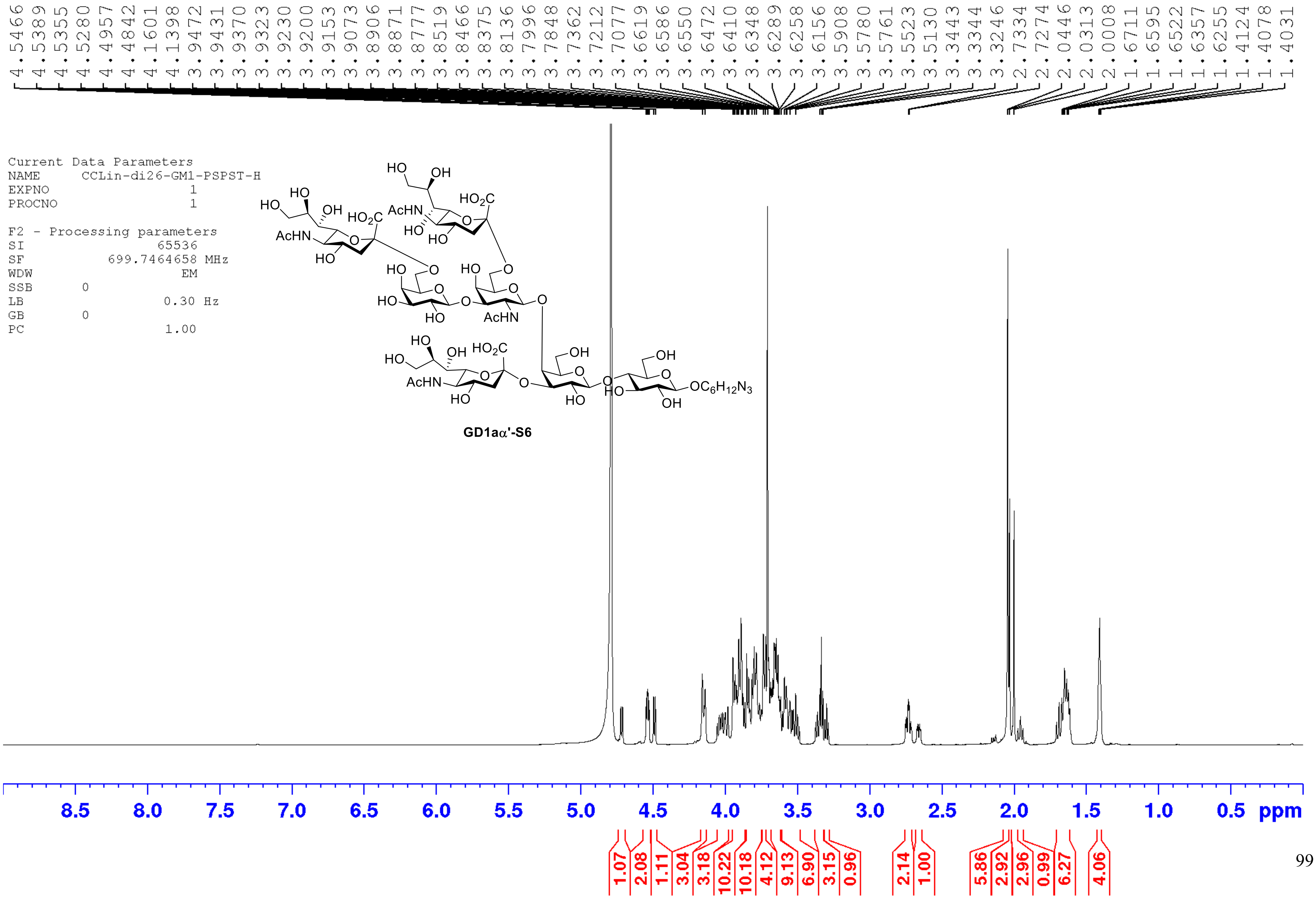

^{1}H NMR spectrum of (**GD1aα'-S6**) (700 MHz D_2O)

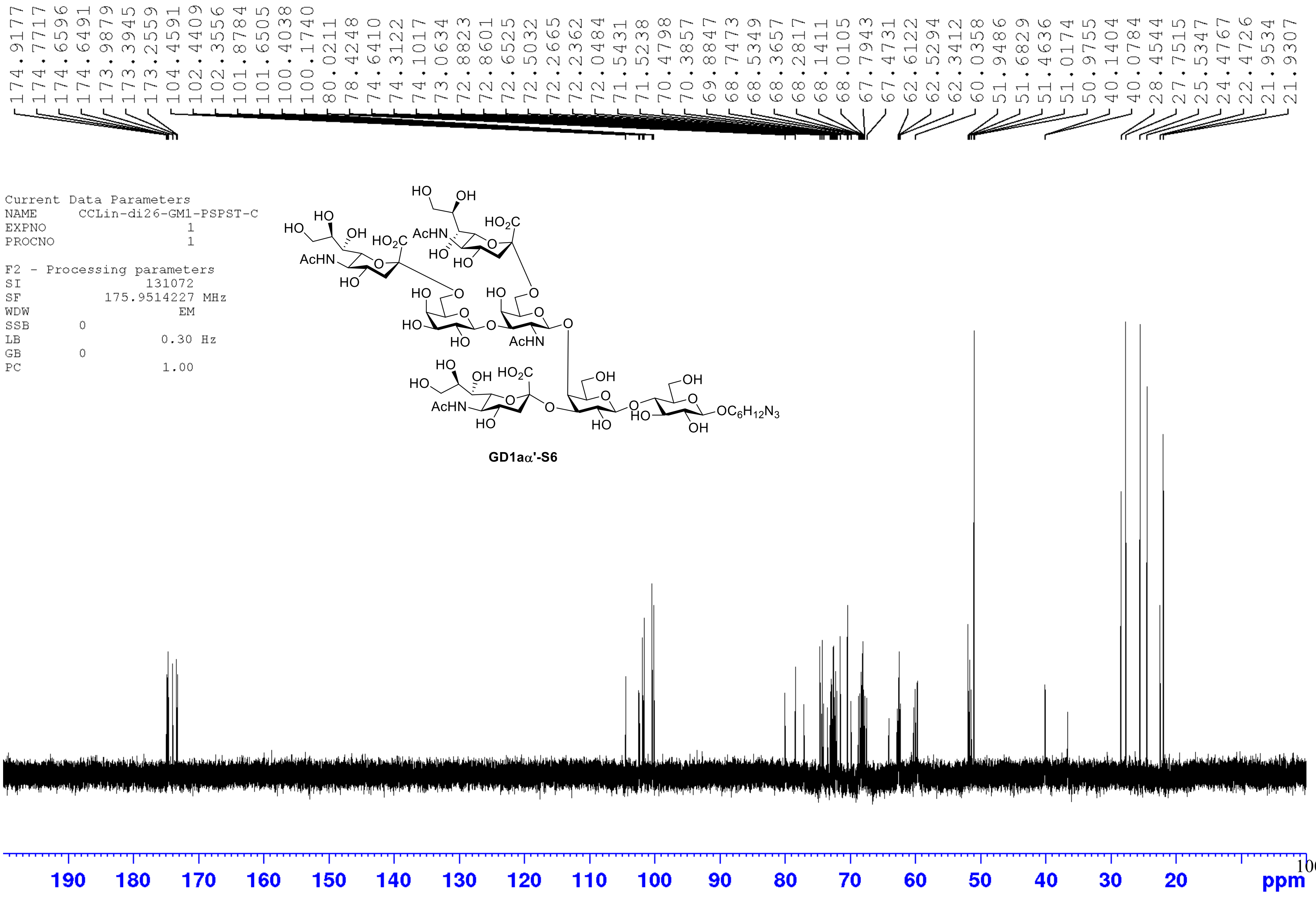

^{13}C NMR spectrum of (**GD1aα'-S6**) (175 MHz D_2O)

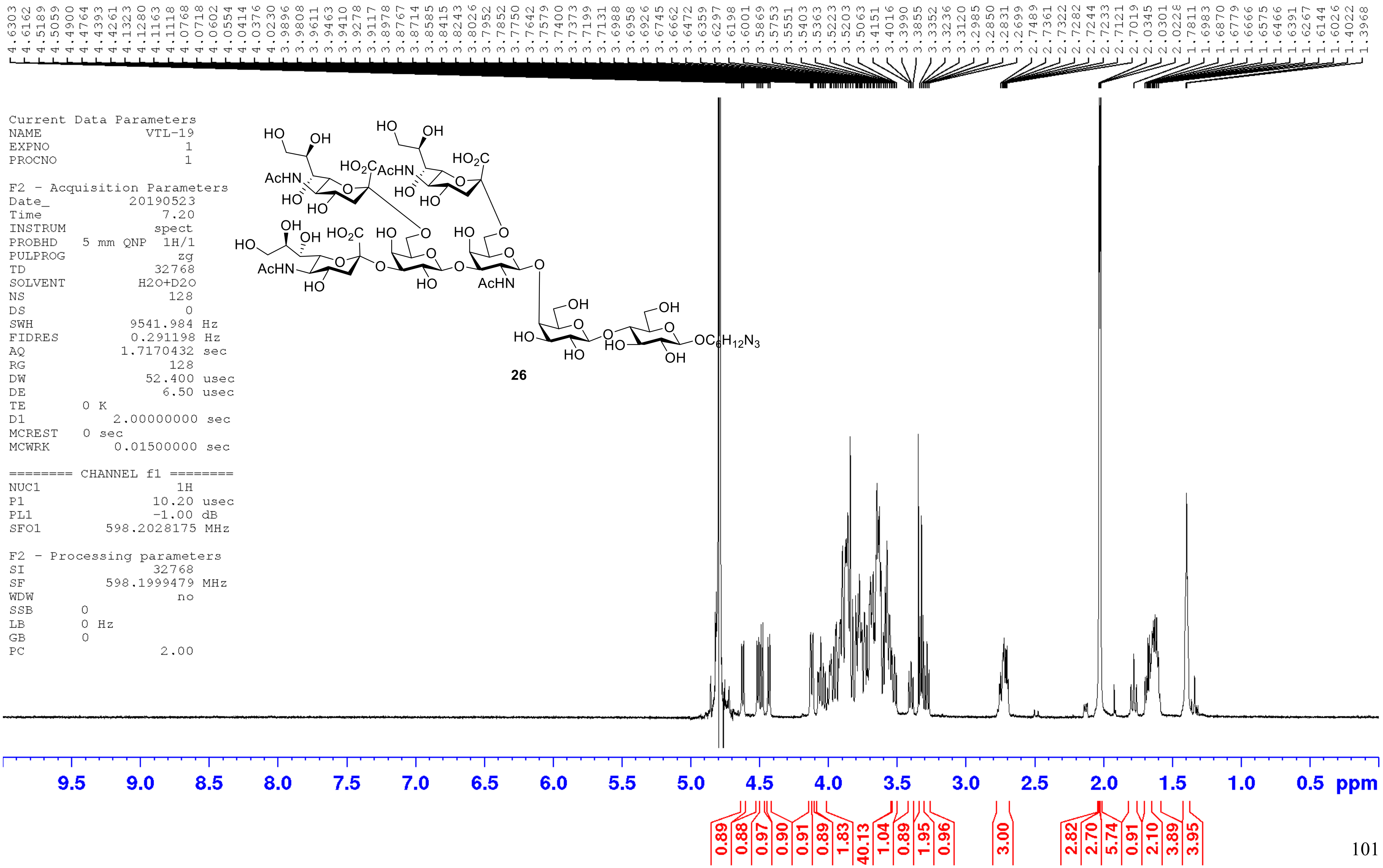

26

^{1}H NMR spectrum of **26** (**GD1aα-S6**) (600 MHz D_2O)

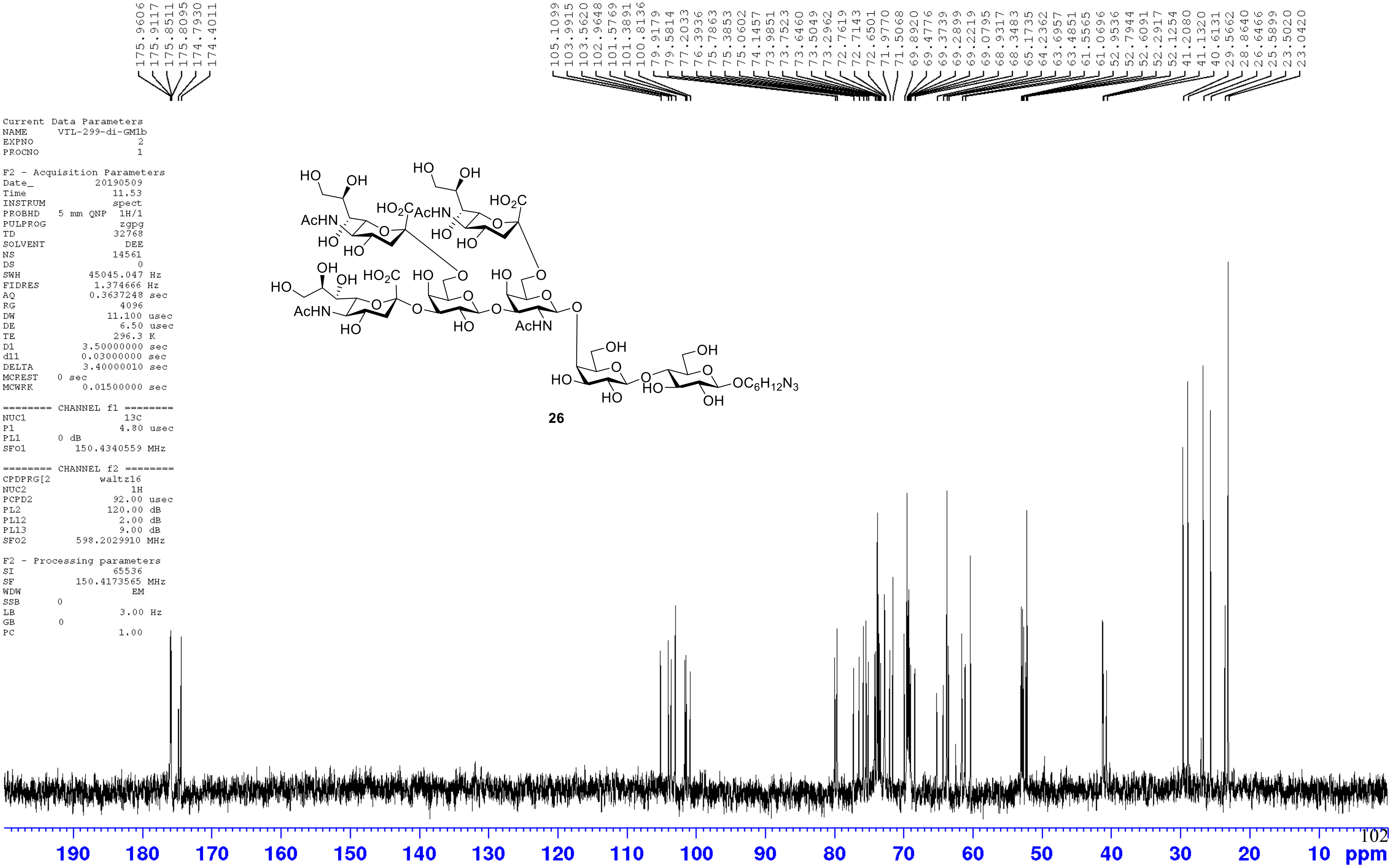

^{13}C NMR spectrum of **26** (**GD1aα-S6**) (150 MHz D_2O)

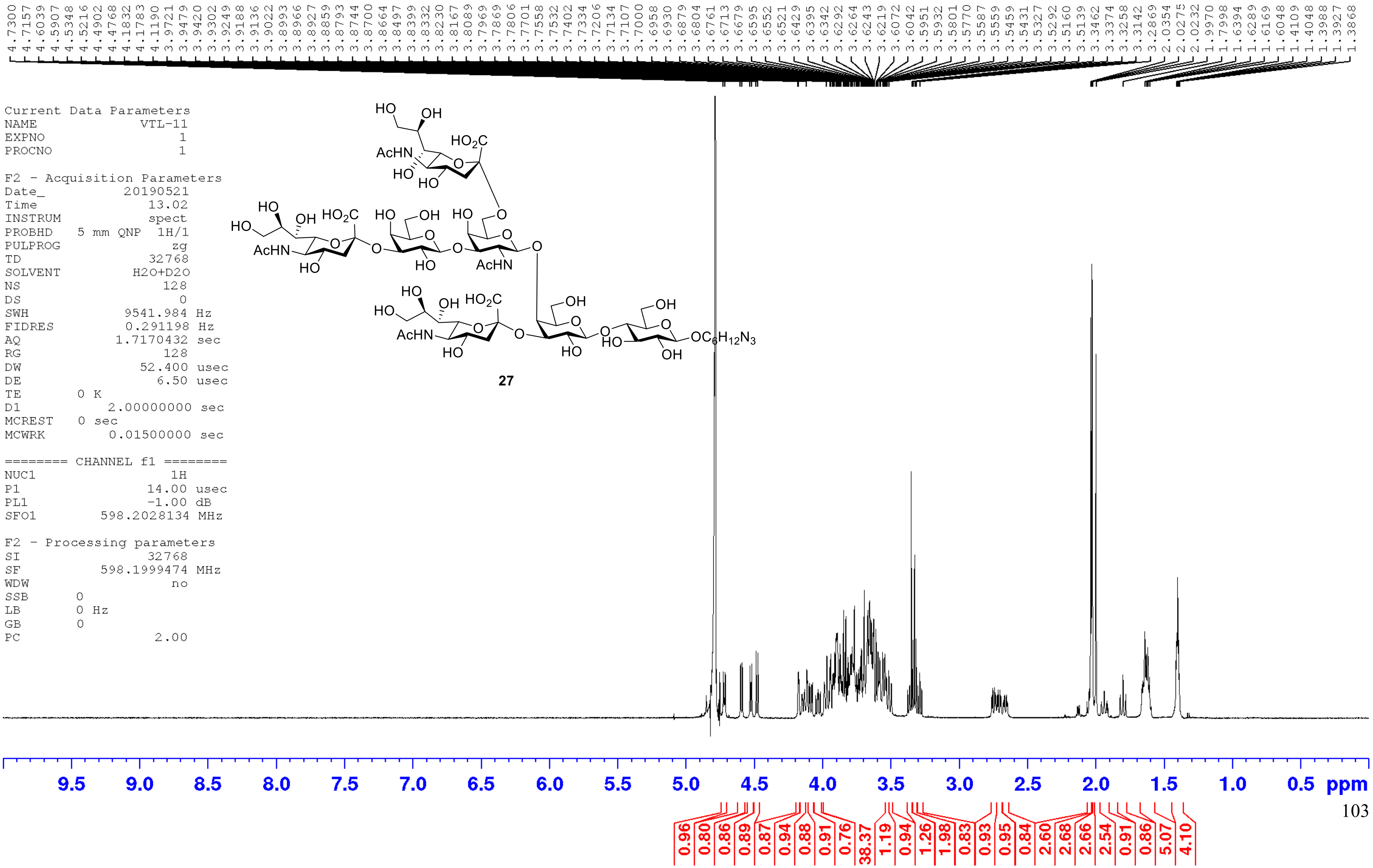

^{1}H NMR spectrum of **27** (**GT1aα**) (600 MHz D_2O)

```
Current Data Parameters
NAME              VTL-11
EXPNO                  2
PROCNO                 1

F2 - Acquisition Parameters
Date_           20190522
Time                2.54
INSTRUM            spect
PROBHD   5 mm QNP  1H/1
PULPROG             zgpg
TD                 32768
SOLVENT          H2O+D2O
NS                 13943
DS                     0
SWH            45045.047 Hz
FIDRES          1.374666 Hz
AQ             0.3637248 sec
RG                  4096
DW                11.100 usec
DE                  6.50 usec
TE        0 K
D1            3.50000000 sec
d11           0.03000000 sec
DELTA         3.40000010 sec
MCREST   0 sec
MCWRK         0.01500000 sec

======== CHANNEL f1 ========
NUC1                 13C
P1                  6.20 usec
PL1       0 dB
SFO1         150.4340559 MHz

======== CHANNEL f2 ========
CPDPRG[2         waltz16
NUC2                  1H
PCPD2              92.00 usec
PL2               120.00 dB
PL12               12.00 dB
PL13               15.00 dB
SFO2         598.2029910 MHz

F2 - Processing parameters
SI                 65536
SF           150.4173617 MHz
WDW                   EM
SSB       0
LB                  4.00 Hz
GB        0
PC                  1.00
```

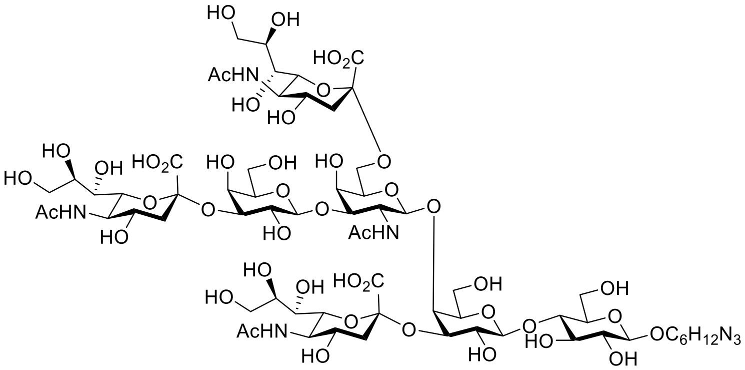

27

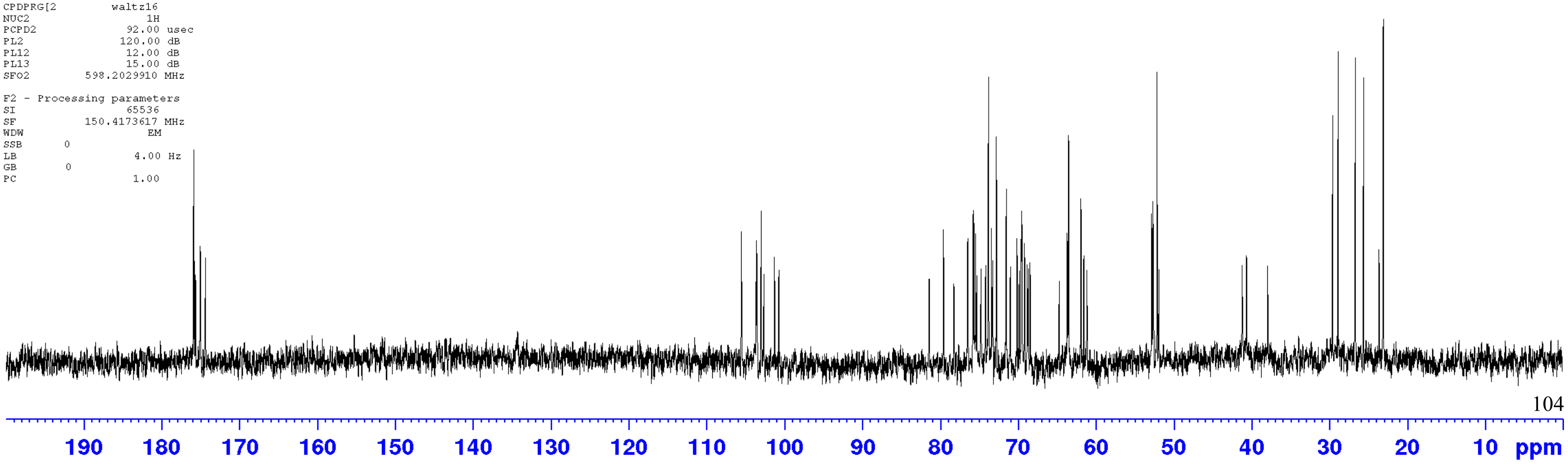

^{13}C NMR spectrum of **27** (**GT1aα**) (150 MHz D_2O)
